# Identification of key components in the energy metabolism of the hyperthermophilic sulfate-reducing archaeon *Archaeoglobus fulgidus* by transcriptome analyses

**DOI:** 10.3389/fmicb.2014.00095

**Published:** 2014-03-11

**Authors:** William P. Hocking, Runar Stokke, Irene Roalkvam, Ida H. Steen

**Affiliations:** Department of Biology, Centre for Geobiology, University of BergenBergen, Norway

**Keywords:** *Archaeoglobus fulgidus*, hydrogenase, heterodisulfide reductase, dissimilatory sulfate reduction, lactate dehydrogenase

## Abstract

Energy conservation via the pathway of dissimilatory sulfate reduction is present in a diverse group of prokaryotes, but is most comprehensively studied in *Deltaproteobacteria*. In this study, whole-genome microarray analyses were used to provide a model of the energy metabolism of the sulfate-reducing archaeon *Archaeoglobus fulgidus*, based on comparative analysis of litoautotrophic growth with H_2_/CO_2_ and thiosulfate, and heterotrophic growth on lactate with sulfate or thiosulfate. Only 72 genes were expressed differentially between the cultures utilizing sulfate or thiosulfate, whereas 269 genes were affected by a shift in energy source. We identified co-located gene cluster encoding putative lactate dehydrogenases (LDHs; *lldD*, *dld*, *lldEFG*), also present in sulfate-reducing bacteria. These enzymes may take part in energy conservation in *A. fulgidus* by specifically linking lactate oxidation with APS reduction via the Qmo complex. High transcriptional levels of Fqo confirm an important role of F_420_H_2_, as well as a menaquinone-mediated electron transport chain, during heterotrophic growth. A putative periplasmic thiosulfate reductase was identified by specific up-regulation. Also, putative genes for transport of sulfate and sulfite are discussed. We present a model for hydrogen metabolism, based on the probable bifurcation reaction of the Mvh:Hdl hydrogenase, which may inhibit the utilization of Fd_red_ for energy conservation. Energy conservation is probably facilitated via menaquinone to multiple membrane-bound heterodisulfide reductase (Hdr) complexes and the DsrC protein—linking periplasmic hydrogenase (Vht) to the cytoplasmic reduction of sulfite. The ambiguous roles of genes corresponding to fatty acid metabolism induced during growth with H_2_ are discussed. Putative co-assimilation of organic acids is favored over a homologous secondary carbon fixation pathway, although both mechanisms may contribute to conserve the amount of Fd_red_ needed during autotrophic growth with H_2_.

## Introduction

The sulfate-reducing prokaryotes (SRP) have played a central role in cycling of carbon and sulfur in anoxic environments throughout long periods of Earth's geological history. Despite early characterization of the cytoplasmic pathway of dissimilatory sulfate reduction (Peck, 1962) it is only in recent years that the mechanisms facilitating energy conservation in SRP have been more comprehensively characterized (Pereira et al., 2011; Grein et al., 2013).

The genus *Archaeoglobus* comprises of archaeal, (hyper)thermophilic, dissimilatory sulfate reducers (Stetter et al., 1987; Stetter, 1988) and is phylogenetically associated with the lineages of *Methanosarcinales*, *Methanomicrobiales*, and uncultured ANME-1 (Brochier-Armanet et al., 2008; Guy and Ettema, 2011). The type species *A. fulgidus* VC16 is a chemolithoautotroph that utilizes H_2_ or formate as electron donors for autotrophic growth. In addition, *A. fulgidus* grows carboxydotrophically on CO/CO_2_ and as a chemoorganoheterotroph utilizing a wide range of substrates including fatty acids, alkenes, complex peptides, and specific amino acids (Stetter et al., 1987; Stetter, 1988; Hartzell and Reed, 2006; Henstra et al., 2007; Khelifi et al., 2010; Parthasarathy et al., 2013). For the complete oxidation of organic substrates to CO_2_, *A. fulgidus* uses a modified acetyl-CoA pathway with similar enzymes and cofactors as in the methanogens (Möller-Zinkhan et al., 1989; Möller-Zinkhan and Thauer, 1990; Vorholt et al., 1995; Estelmann et al., 2011). Reduction of sulfate (SO^2−^_4_) to sulfide (S^2−^) in *A. fulgidus* proceeds via the highly conserved dissimilatory sulfate reduction pathway of the SRP (Peck, 1962; Klenk et al., 1997; Pereira et al., 2011). This was probably acquired by *Archaeoglobales* via multiple lateral gene transfer events from an early ancestor of clostridial SRP (Klein et al., 2001; Zverlov et al., 2005; Meyer and Kuever, 2007).

The energy conservation mechanisms in *A. fulgidus* are incompletely understood. During growth on lactate, the reduced coenzyme F_420_ (F_420_H_2_) is generated from the oxidative acetyl-CoA pathway. The presence of both menaquinone and a homolog of the respiratory NAD(P)H:quinone oxidoreductase complex, the F_420_H_2_:quinone oxidoreductase complex (Fqo), suggest that electrons from F_420_H_2_ are transferred to the membrane-bound respiratory chain by the Fqo complex. Fqo probably couples the reduction of menaquinone and proton translocation. (Tindall et al., 1989; Kunow et al., 1993; Baumer et al., 2000; Brüggemann et al., 2000) A d-lactate dehydrogenase is confirmed to be present (Reed and Hartzell, 1999), but it is unclear how this membrane associated enzyme facilitates energy conservation, as it is shown to interact with a NADH oxidase (Pagala et al., 2002). Also, the cofactor NAD(P)H plays a negligible role in energy conservation (Noll and Barber, 1988; Kunow et al., 1993; Warkentin et al., 2001).

There is also a possible alternative energy conservation pathway in *A. fulgidus*. In *D. vulgaris*, cytochrome c mediated “hydrogen cycling” is suggested as an energy conservation mechanism during growth with lactate (Odom and Peck, 1981; Keller and Wall, 2011). In this reaction, formation of hydrogen is a result of cytoplasmic oxidation of lactate. The subsequent diffusion and periplasmic oxidation of hydrogen contributes to the formation of a proton gradient. In *Methanosarcina barkeri*, the Vht/Vhx dehydrogenase also facilitates a hydrogen cycling mechanism under heterotrophic growth conditions, and sustains growth when Fpo (Fqo) is absent in deletion mutants (Kulkarni et al., 2009). The presence of a cytoplasmic as well as a periplasmic hydrogenase in *A. fulgidus* (Mander et al., 2004) potentially fulfills requirements for a “hydrogen-cycling” mechanism.

Two co-located heterodisulfide reductase (Hdr)-associated hydrogenases are present in the genome of *A. fulgidus*, which are homologous to those involved in energy conservation in the methanogens (Mander et al., 2004). These are the soluble [NiFe]hydrogenase/heterodisulfide-like (MvhABC/HdlABC) complex and the membrane-bound uptake hydrogenase, “F_420_-non-reducing hydrogenase” (Vho/Vht). Reduced ferredoxin (Fd_red_) is essential for fixation of CO_2_ through the acetyl-CoA pathway. In methanogens, the Mvh:Hdl complex homolog, Mvh/Hdr, couples the exergonic reduction of the heterodisulfide, CoM-S-S-CoB, with endergonic reduction of ferredoxin with H_2_, by a flavine-based bifurcation mechanism (Kaster et al., 2011). The periplasmic Vht hydrogenase reduces the quinone-like cofactor methanophenazine coupled to the membrane-bound HdrDE, facilitating energy conservation during growth on H_2_ (Ide et al., 1999; Thauer et al., 2010).

Despite the absence of genes and cofactors for terminal methanogenesis (Stetter et al., 1987; Klenk et al., 1997), several factors suggest that thiol/disulfide conversions catalyzed by Hdr are involved in electron transfer and energy conservation in *A. fulgidus*, as has been proposed for methanogens and more recently for SRP (Mander et al., 2002, 2004; Pereira et al., 2011; Grein et al., 2013). All known SRP, including *A. fulgidus*, encode HdrA and HdrDE related genes, which almost ubiquitously form membrane-bound redox complexes (Pereira et al., 2011; Grein et al., 2013). These complexes may facilitate energy conservation during different steps of sequential dissimilatory sulfate reduction. The quinone-interacting membrane-bound oxidoreductase (QmoABC) complex probably links the electron transfer chain to the first reductive step of sulfate reduction catalyzed by adenosine-5′-phosphosulfate (APS) reductase (AprAB) (Pires et al., 2003; Zane et al., 2010; Grein et al., 2013). In *Desulfovibrio* it has recently been proposed that the Qmo subunit homologous to the bifurcating HdrA, QmoB, may facilitate a “confurcation” mechanism (Ramos et al., 2012). The “confurcating” Qmo complex may catalyze energy conservation by proton translocation via an endergonic periplasmic menaquinol oxidation, driven by an exergonic cytoplasmic oxidation reaction coupled to terminal reduction of APS. The second complex, DsrMK, is a homolog of HdrDE, and is ubiquitous amongst SRP (Pereira et al., 2011). This complex probably facilitates energy conservation and is linked by electron transfer via disulfide/thiol redox reactions, to the terminal step of sulfite reduction by bisulfite reductase/sulfite reductase (DsrAB) (Mander et al., 2002; Pires et al., 2006). Similarly to the HdrDE of methanogens, the DsrMK complex probably couples periplasmic oxidation of reduced menaqinone (instead of reduced methanopenazine) to cytoplasmic cysteine disulfide (Cys-S-S-Cys) reduction, in the enzyme DsrC (in stead of a CoM-S-S-CoB) (Mander et al., 2005). Unusually, *dsrMK* is encoded by multiple homologs in *A. fulgidus*, corresponding to multiple DsrMK and a DsrMK(JOP) complex, which differ in domain composition and among lineages of SRP (Klenk et al., 1997; Pereira et al., 2011). The *dsrC* gene is ubiquitously present in SRP, and DsrC is the probable link between heterodisulfide reductase (DsrK) and DsrAB (Oliveira et al., 2008; Pereira et al., 2011; Grein et al., 2013). However, it should be noted that although it is likely that the DsrMK(JOP) complexes may facilitate proton translocation by MQH_2_ oxidase:DsrC reductase, it is questioned whether this reaction is thermodynamically favorable (Thauer et al., 2007; Grein et al., 2013).

The role of reduced ferredoxin (Fd_red_) in energy conservation in SRP remains unclear, as it has been proposed as an electron donor for both APS and sulfite reduction (Oliveira et al., 2008, 2011; Ramos et al., 2012). In *A. fulgidus*, this offers a potential coupling between ferredoxin and electron transport phosphorylation, but also represents a significant bioenergetic challenge, as fixation of CO_2_ through the acetyl-CoA pathway requires Fd_red_. Interestingly, while chemoorganotrophic and carboxydotrophic growth are coupled to sulfate reduction in *A. fulgidus*, only thiosulfate or sulfite are utilized with H_2_ as energy source (Stetter et al., 1987; Steinsbu et al., 2010). This may potentially be coupled to the role of Fd_red_ in energy and carbon metabolism.

To provide a deeper insight into electron transport and energy conservation mechanisms in *A. fulgidus*, we used whole genome microarrays to identify redox complexes expressed under different growth conditions. Previously, only the heat shock response in *A. fulgidus* has been characterized by global transcriptional profiling (Rohlin et al., 2005). We examined heterotrophic growth with lactate and litoautotrophic growth with H_2_, as well as the differential use of the electron acceptors thiosulfate and sulfate. The results form an overall energy conservation model where the Fqo and membrane-bound electron transport, facilitated by menaquinone, Qmo and multiple DsrMK, are central to energy conservation during growth with lactate. During growth with hydrogen, our model suggests that Fd_red_, generated by Mvh:Hdl, is utilized primarily for carbon assimilation and probably does not contribute to energy conservation. From the data and comparative genomics it seems likely that the inability of *A. fulgidus* to grow with sulfate when hydrogen is an energy source is caused by transcriptional regulation of the gene for pyrophosphatase, resulting in the blocking of APS formation. Overall, the results point to a key role in energy conservation for electron transfer from hydrogen to thiosulfate, facilitated by thiol/disulfide conversions catalyzed by membrane-bound DsrMK in *A. fulgidus*.

## Methods

*Archaeoglobus fulgidus* strain VC16 (DSMZ 4302) obtained from the Deutsche Sammlung von Mikroorganismen und Zellkulturen (Braunschweig, Germany) was cultivated in anoxic, carbonate buffered medium (10 ml medium in 26 ml serum vials) under an atmosphere of N_2_:CO_2_ 80:20 (1 atm), at pH 6.8. The composition of the media was as follows: 0.32 g/l KCl, 1.0 g/l MgCl_2_•6H_2_O, 0.25 g/l NH_4_Cl, 0.14 g/l CaCl_2_•2H_2_O, 0.11 g/l K_2_HPO_4_•3H_2_O, 0.2 g/l KH_2_PO_4_, 18.0 g/l NaCl, and 0.3 g/l yeast extract. Minor constituents were; 0.015 g/l Titriplex I (Nitriloaceticacid), 0.005 g/l MnSO_4_•2H_2_O, 0.001 g/l CoCl_2_•6H_2_O, 0.001 g/l ZnSO_4_•7H_2_O, 0.0001 g/l CuSO_4_•5H_2_O, 0.0001 g/l H_3_BO_3_, 0.0001 g/l Na_2_MoO_4_•2H_2_O, 0.002 g/l NiSO_4_•6H_2_O, 0.039 mM (NH_4_)_2_Fe(SO_4_)_2_•6H_2_O, and 0.5 ml/l 0.2% Resazurin. After autoclaving, sterile anoxic solutions were added to the medium to a final concentration of 30 mM NaHCO_3_ and 0.25 mM Na_2_S. When thiosulfate was used as an electron-acceptor, sulfate; 2.2 g/l Na_2_SO_4_ and 3.7 g/l MgSO_4_•7H_2_O, was exchanged with 3.7 g/l MgCl_2_6H_2_O and 7.45 g/l Na_2_S_2_O_3_•5H_2_O (thiosulfate). All media contain 0.18 mM SO^2−^_4_ attributed to the composition of minor constituents. Filter-sterilized (0.2 μM), anoxic, thiosulfate solution was added after autoclaving. During heterotrophic growth, 35 mM sodium-d,l-lactate (50/50) was added to the medium whereas 250 kPa H_2_:CO_2_ (80:20 ratio) was used during litoautotrophic growth.

Cultivation was performed at 80°C and the tubes were incubated at an approximate angle of 6°ensuring a high surface to volume ratio. The turbidity of samples (absorbance at 600 nm) was used for monitoring of cultures; linearity against direct cell counts for all growth conditions was confirmed throughout the absorbance range, (using a Thoma-chamber; depth 0.02 mm). All cultures were harvested at a pre-determined absorbance, on the basis of growth experiments.

Cultures were flash cooled (20 s) to approximately 0°C in a −80°C, 70% ethanol:water slurry, and harvested by centrifugation in 15 ml falcon tubes at 3000 g for 15 min at 0°C. The pellet was immediately re-suspended in 100 μl RLT buffer of the RNeasy kit (Qiagen) and stored at −80°C for a maximum of 1 week before total RNA extraction.

### Preparation of RNA

Upon RNA extraction, samples were thawed to 37°C and placed on ice. Samples from equivalent growth conditions were pooled in numbers sufficient to obtain the required yield (1 μg). The final volume was adjusted to 600 μl with buffer (RLT, RNeasy) before proceeding. The RNeasy mini kit (Qiagen) was used for the total RNA extraction and with an additional DNase I (Qiagen) step. RNA was concentrated using the RNeasyMinElute kit (Qiagen) to achieve the required concentration of 1 μg/ml total RNA for cDNA synthesis. Total RNA concentration was determined photometrically (Cary 300 UV-Vis, Varian) using a TrayCell cuvette with a 0.2 mm cap (Hellma, Germany), and RNA quality was evaluated using a RNA 6000 Nano kit with a Bioanalyzer 2100 instrument (Agilent).

**Figure 1:**
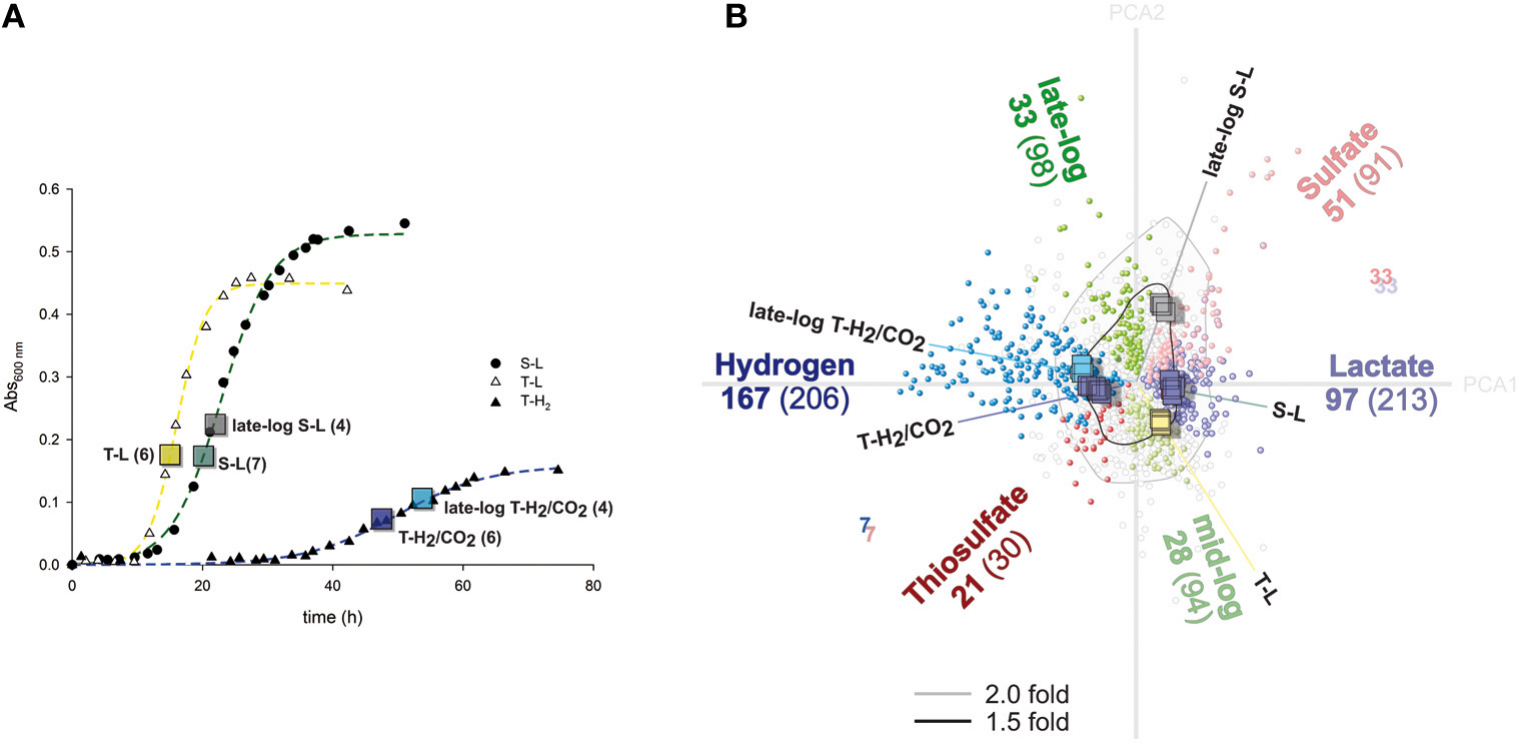
**Growth curves and illustration of differentially regulated genes. (A)** Representative growth curves of cultures grown with lactate and sulfate (S-L), lactate and thiosulfate (T-L), hydrogen/CO_2_ and thiosulfate (T-H_2_). Boxes indicate absorbance values when sampling was performed. The numbers indicate amount of replicate microarray hybridizations for each growth condition. **(B)** Correspondence analysis (CA) provides an overview of variance and differentially regulated genes determined by ANOVA. Genes (circles) and individual hybridizations (squares) are clustered with respect to each other by CA, and are displayed along the two first principle components (Total variance retained 41%; Principle Component Axis—gray lines; PCA1: 27.2%, PCA2: 13.8%). Colored lines indicate medians corresponding to each growth condition. Significantly differentially expressed genes (ANOVA) are colored according to growth condition; black lines denote a fold change greater than 1.5.

Each sample prepared for hybridization was the result of pooling in order to obtain sufficient material for analysis. Samples consist of 5 individual randomly selected tubes for T-H_2_/CO_2_ samples, and 2 individual tubes for all S-L and T-L samples. A total of 27 microarray hybridizations were performed. These correspond to the following growth conditions (outline of design; Figure 1A): S-L, 10 hybridizations (mid-log 6, late log 4); T-L 6 hybridizations (mid-log); T-H_2_/CO_2_ 11 hybridizations (mid-log 7, late log 4). The arrays utilized were the commercially available Roche Nimblegen 080626 Aful DSM4304 design, utilizing the 4-plex array design (4 × 72K format) where 2392 open reading frames (ORF's) are assayed. RNA was prepared according to the guidelines provided for the Nimble Chip arrays (Nimblegen systems, 2007); double stranded cDNA was synthesized by Superscript Double-Stranded cDNA synthesis kit (Invitrogen), with Random Hexamer Primer (Roche Applied Science). Subsequent steps were performed as recommended by the array supplier.

The analysis of the obtained image, and quality control was performed by the NimbleScan software version 2.5, values obtained from robust multiarray average (RMA) normalization (Irizarry et al., 2003) was utilized for further analysis. The data were deposited in the ArrayExpress database (https://www.ebi.ac.uk/arrayexpress/) under the accession code: E-MTAB-2294.

RMA normalized data were further analyzed by the J-Express software 2012 (http://jexpress.bioinfo.no/site/), and quantile normalization was performed on all samples (Bolstad et al., 2003). Due to the quantile normalization procedure, the mean intensity of all assayed genes is practically constant. Therefore, the mean signal intensity was arbitrarily set as 1.0 and values are reported relative to this level in order to convey the level of signal abundance.

Analysis of variance (ANOVA) is the principle method of statistical evaluation in this work. In order to perform ANOVA, a similar sample size is required. The 27 samples correspond to five different growth conditions and were of unequal size. To increase robustness of analysis, the minimum residual sum of squares per gene from the total data was selected for ANOVA. Therefore, 4 values representing each of the corresponding growth conditions were analyzed; S-L, late-log S-L, T-L and T-H_2_/CO_2_, and late-log T-H_2_/CO_2_. We report differential expression as significant when the ANOVA returns a *p*-value of less than 0.00001 (*p* < 0.00001, critical *F* > 37.71). A significant shift between two groups corresponding to 1.5 fold or larger was generally considered as a cut-of for major differential expression. Correspondence analysis was used for clustering of individual samples (Fellenberg et al., 2001) and as a control of the ANOVA (Figure 1B).

Functional annotation was performed using the latest version of archaeal clusters of orthologous genes (arCOG) (Wolf et al., 2012). Enrichment analysis was performed using analysis between selected groups and entire dataset using the Chi-squared test (*p* < 0.05 for groups larger than 5 genes). Association of individual genes to KEGG pathways were retrieved from the KEGG database (http://www.kegg.jp/). In order to evaluate the validity of signal intensity vs. functional genes, enrichment of genes corresponding to KEGG pathways were evaluated by a Kolmogorov–Smirnov statistic equivalent; Gene Set Enrichment Analysis (Subramanian et al., 2005). Enrichment of KEGG pathway associated genes was performed on a list sorted by minimal recorded signal-intensity per gene from any sample.

Homology searches were conducted using either BLASTp or PSI-BLAST using default settings (http://blast.ncbi.nlm.nih.gov/Blast.cgi), while conserved domains were identified using the Conserved Domains Database (CDD) database (http://www.ncbi.nlm.nih.gov/Structure/cdd/cdd.shtml) (Marchler-Bauer et al., 2011). Shared synteny of gene clusters were identified using the STRING database (http://string-db.org/), in combination with the de-novo synteny explorers Absynte and Syntax (http://archaea.u-psud.fr/archaea_software_page.html) (Despalins et al., 2011; Oberto, 2013); which also provide the graphical templates for Figures 3, 4.

## Results

### Growth and general transcriptional shift

Doubling time was more than halved in *A. fulgidus* when thiosulfate (T-L) was added as terminal electron acceptor instead of sulfate (S-L) during growth with lactate (Figure 1A). The specific growth rate (μ) evaluated by 7 replicate cultures, was significantly higher for T-L (μ: 0.28 ± 0.07 h^−1^; doubling time 1.1 ± 0.4 h), than both S-L (0.12 ± 0.004 h^−1^; 2.4 ± 0.1 h) and T-H_2_/CO_2_ (0.13 ± 0.03 h^−1^; 2.3 ± 0.9 h). Turbidity was estimated to increase by 2.6 absorbance units per cell (*A*_600nm_) in cultures grown with lactate (S-L, T-L) compared to the T-H_2_/CO_2_ cultures. Hence, growth rate increased during growth with T-L, vs. indistinguishable rates between S-L and T-H_2_/CO_2_ cultures. The increase in growth yield inferred from absorbance in lactate-grown cultures may be partially explained by an increase in cell size during growth with lactate.

The 27 transcriptional profiles of *A. fulgidus* cells cultivated with S-L, T-L, T-H_2_/CO_2_, and late log-phase cells cultivated with S-L and T-H_2_/CO_2_ were compared by microarray analysis (Figure 1A). Correspondence analysis (Fellenberg et al., 2001) revealed that individual samples clustered together, with samples from similar growth conditions being distinct from other assayed conditions (Figure 1B). Between any of the assayed conditions a total of 1268 genes were differentially expressed (ANOVA *p* < 0.00001); of these 514 genes were differentially expressed over 1.5 fold (53%; and 21% of total assayed genes).

The analysis identified 692 differentially regulated genes (29% of assayed genes, Figure 1B) corresponding to either electron donor/carbon source (S-L/T-L vs. T-H_2_/CO_2_), electron acceptor (S-L vs. T-L, T-H_2_/CO_2_) or growth phase (log; S-L, T-L, T-H_2_/CO_2_ vs. late log; S-L, T-H_2_/CO_2_). Of these, 369 genes were over 1.5 fold differentially expressed (15% of assayed genes, Figure 1B).

The differentially regulated genes were as follows (Figure 1B): hydrogen vs. lactate 419 genes; 264 over 1.5 fold (17.5%; 11%: 167/97 up/down), thiosulfate vs. sulfate 121 genes; 68 over 1.5 fold (5.1%; 2.8%: 21/47), late vs. mid-log; 192 genes; 61 over 1.5 fold (8%; 2.6%: 33/28). Hence, a shift in energy metabolism and carbon source introduced the largest number of differential regulation. Whereas, a shift in electron donor contributes less to the total magnitude of transcriptional regulation, at comparable levels to differential expression related to growth phase. Differential expression corresponding to *either* late log S-L, late log T-H_2_/CO_2_, or T-L were not considered further (21% of assayed genes and 6% regulated above 1.5 fold).

### Differentially expressed genes with hydrogen and CO_2_ vs. lactate

**Figure 2:**
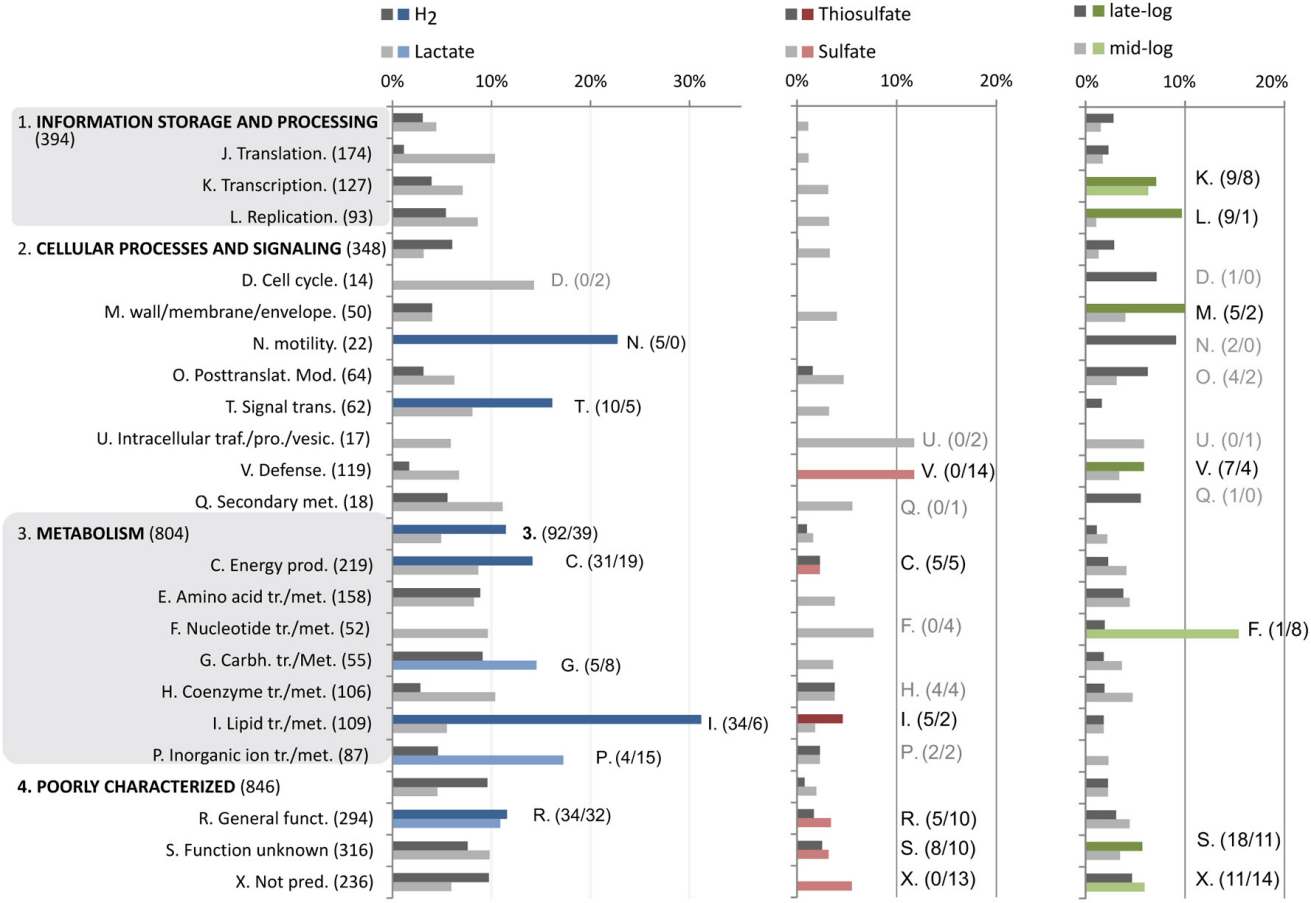
**Distribution and enrichment of COG categories that are differentially regulated corresponding to specific factors (Figure 1B)**. Bar plots display expression as percentage of each COG category. Bars highlighted with specific colors (as in Figure 1B) correspond to significantly enriched COG categories of genes induced by this factor (Chi-squared test; *p* > 0.05, more than 4 genes). The numbers denoted in brackets are the number of differentially regulated genes in each group.

Of the 206 genes up-regulated in cultures grown with T-H_2_/CO_2_ vs. S-L and T-L, 92 were affiliated with the COG's corresponding to metabolic processes: where 31 genes corresponding to energy production (C) and 34 to lipid transport and metabolism (I) were significantly enriched (Figures 1B, 2). There was also a major differential expression involving 5 genes related to cellular motility (N) and signal transduction (T). Specific genes in the energy production category included the 10 co-located hydrogenase genes of two distinct complexes (AF1372-AF1377, AF1379-AF1381, Table 1D; Mander et al., 2004), and a membrane-bound Hdr (AF0755, Table 1C).

**Table 1:**
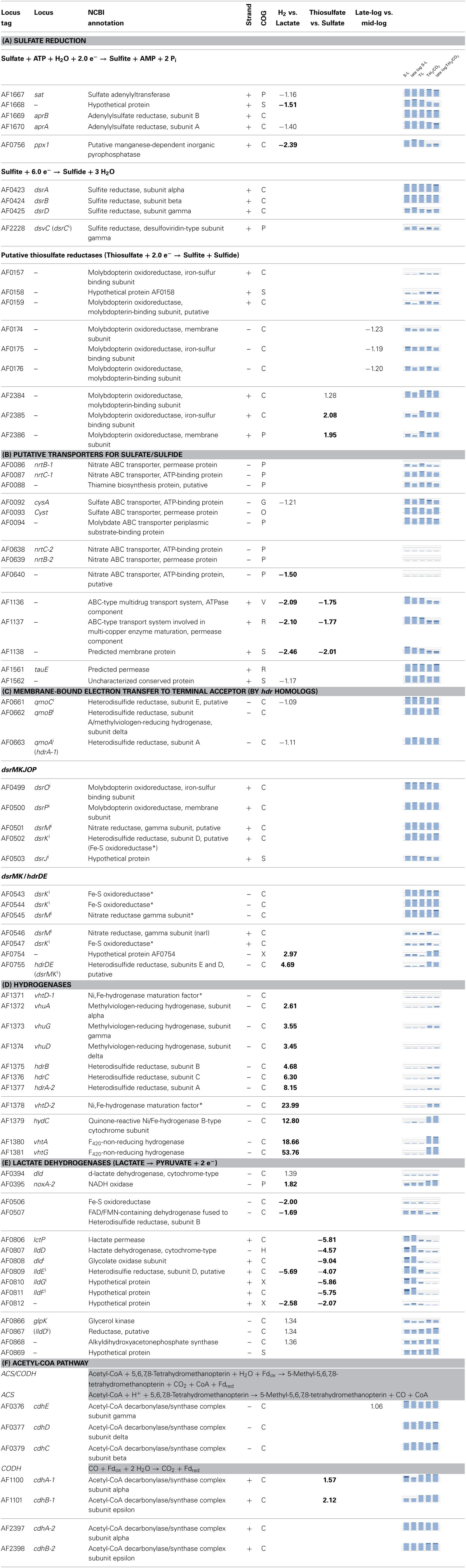
**Differential expression (fold change; above 1.5 in bold, ANOVA; *p*-value 0.00001) of selected genes corresponding to processes in Figure 5, graphs display transcriptional abundance (1–3 fold average expression) and standard deviance around mean (dark area)**.

^*^Annotation from arCOG; (Wolf et al., 2012).

*^i^Inferred locus annotation*.

**Figure 3:**
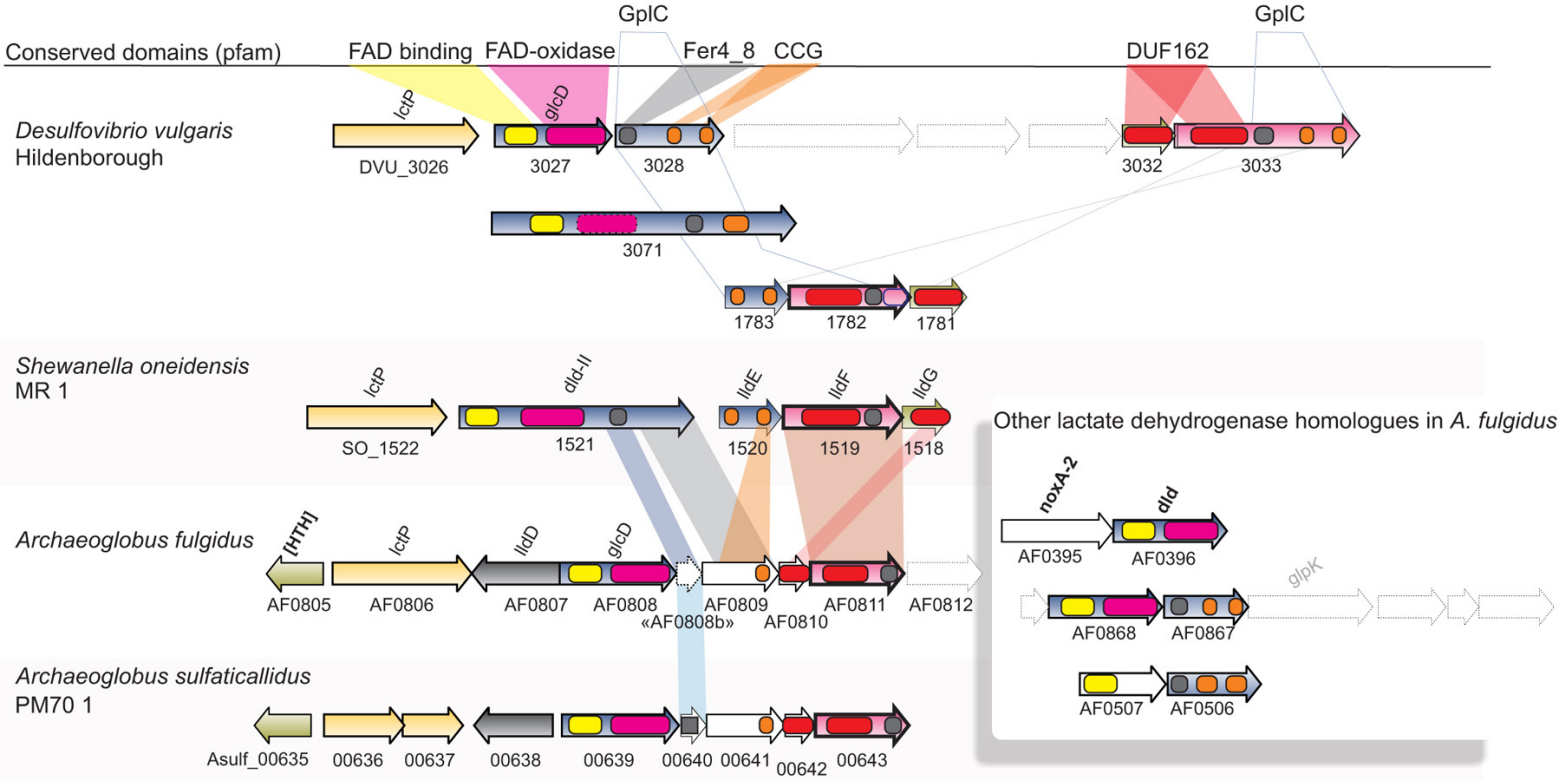
**Synteny and domain composition between lactate dehydrogenases and associated genes**. The genes of *A. fulgidus* correspond to a lactate dehydrogenase (lldEFG), d-lactate dehydrogenase (dld/dld-II), and lactate permease (lctP) which are found co-located in a wide range of bacterial species. Genes with sufficient homology between bacterial and archaeal sequences are colored correspondingly. Although, homology is generally low between genes, the domain composition for the gene clusters is conserved.

Surprisingly, the genes corresponding to d-lactate dehydrogenase (AF0394) and a putative d-lactate dehydrogenase gene (AF0868) (Reed and Hartzell, 1999; Pagala et al., 2002) were induced during growth with T-H_2_/CO_2_. The gene AF0394 was up-regulated by a minor fold (<1.5) and co-regulated (Pearson's *r*-value; 0.89) with the associated NADH oxidase gene (AF0395, >1.5 fold) (Table 1E, Figure 3).

Other highly induced genes classified under energy production belong to the TCA cycle; succinate dehydrogenase (AF0682-AF0683) and malate oxidoreductase (AF1727) which probably has a non-energetic, assimilatory role in *A. fulgidus* (Table S1d). Up-regulated genes; alcohol dehydrogenase (AF0024, AF0339) and acyl-CoA transferase/carnitine dehydratase (AF0973-AF0974), potentially play a role in co-assimilation of organic substrates during growth with T-H_2_/CO_2_ (Table S3). This may also relate to the specific induction of several of the genes of lipid metabolism corresponding to fatty acid activation and beta-oxidation (Table S1a, Figure 7); acyl-CoA synthetase/AMP-acid ligase II (*fadD*/*alkK*; 9/16 induced homologous genes in genome), acyl-CoA dehydrogenase (*acd*; 4/14), enoyl-CoA hydratase (*fad*; 3/5), 3-hydroxyacyl-CoA dehydrogenase (*hdb*; 3/10) and acetyl-CoA acetyltransferase (*acaB*; 3/15). The genes of acyl-coenzyme A synthetase/AMP-(fatty) acid ligase (*acs*; 5/8) may have a role in fatty acid activation or acetate fixation together with a 3-hydroxy-3-methylglutarate CoA synthase homolog (AF0033). The induced genes of putative sterol carrier protein (2/3; AF1174, AF1678) and a short chain fatty acid transporter (AF1538) may play a role in transport of lipids across the membrane. The genes linked to fatty acid oxidation may form part of the 3-hydroxypropionate/4-hydroxybutyrate pathway of CO_2_ assimilation (Figure 7) (Berg et al., 2007). In relation to fixation of CO_2_, the gene of the large-subunit of ribulose bisphosphate carboxylase (RuBisCo; AF1587, Table S3) was also highly up-regulated during growth with T-H_2_/CO_2_. Carboxylase activity has been verified in *A. fulgidus* (Watson et al., 1999). However, the role of RuBisCo in anaerobic Archaea may relate to ribulose 1,5-bisphosphate recycling, or AMP metabolism, rather than a carbon assimilation mechanism (Sato et al., 2007; Estelmann et al., 2011).

Genes related to motility (N) were of flagellin (AF1054, AF1055, Table S3) and archaeal flagellar biosynthesis (AF0338) were induced during growth with T-H_2_/CO_2_, indicating a taxis response to substrate. The induced genes of signal transduction pathways (T) involved genes encoding proteins with potential Per-Arnt-Sim (PAS) domains (AF0277, AF0448, AF1045, AF1472, AF2420). These may correspond to cellular redox sensors that have been linked to chemotaxis in Euryarchaeota, but also hydrogenase expression in Bacteria (Taylor and Zhulin, 1999; Lenz et al., 2002; Shaw et al., 2009). Other up-regulated genes corresponding to intracellular signaling were: histidine kinases (AF0893, AF1483), c-AMP binding (AF0971) and universal stress protein (AF1526). In addition, genes encoding several putative permeases and transporters were up-regulated during growth on hydrogen; permeases (AF0121m, AF0123-AF0124), and putative proline permease/sodium: solute symporters (AF0965-AF0966, AF0969, AF0981-AF0982).

Fewer genes were highly induced (97 genes, >1.5 fold) by utilization of lactate (S-L, T-L) in comparison to utilization of T-H_2_/CO_2_ (Figures 1B, 2). These genes were functionally enriched in COG's corresponding to metabolic processes such as carbohydrate metabolism (G); where carbohydrate kinase (AF1751) and phosphoglycerate mutase (AF1752) were induced above 1.5 fold. During growth with lactate (S-L, T-L) only a gene with low homology to d-lactate dehydrogenase was specifically induced (AF0507, AF0506; Table 1E, Figure 3). In the inorganic ion transport (P) category, up-regulated genes encoding ABC-type multidrug transporter (AF1136-AF1140, Table 1B), and a phosphate ABC transporter (AF1356-AF1360), could play a role in substrate uptake or sulfate transport. A region of unknown, short, DUF2589 related genes (AF0414-AF0417) were also specifically up-regulated during growth with lactate.

The genes encoding enzymes of the dissimilatory sulfate reduction pathway are affiliated with two COG categories (P and C, Table 1A). The genes related to reduction of sulfate, i.e., APS formation and reduction; *sat* and *aprAB* (AF1667-AF1670, Table 1A) and the membrane-bound QmoABC complex (AF0661-AF0663, Table 1C) were highly expressed at all times, but significantly down-regulated by less than 1.5 fold in relation to a shift in energy donor from lactate to hydrogen. The inorganic pyrophosphatase (*ppx*, AF0756, Table 1A) which is perceived to drive the formation of APS (Peck, 1962) was more than 2 fold down-regulated in relation to growth with T-H_2_/CO_2_. Differential regulation of these genes may be related to energy donor (lactate vs. H_2_) and not terminal electron acceptor, as no regulation was observed between S-L and T-L samples.

### Differentially expressed genes with thiosulfate vs. sulfate

**Figure 4:**
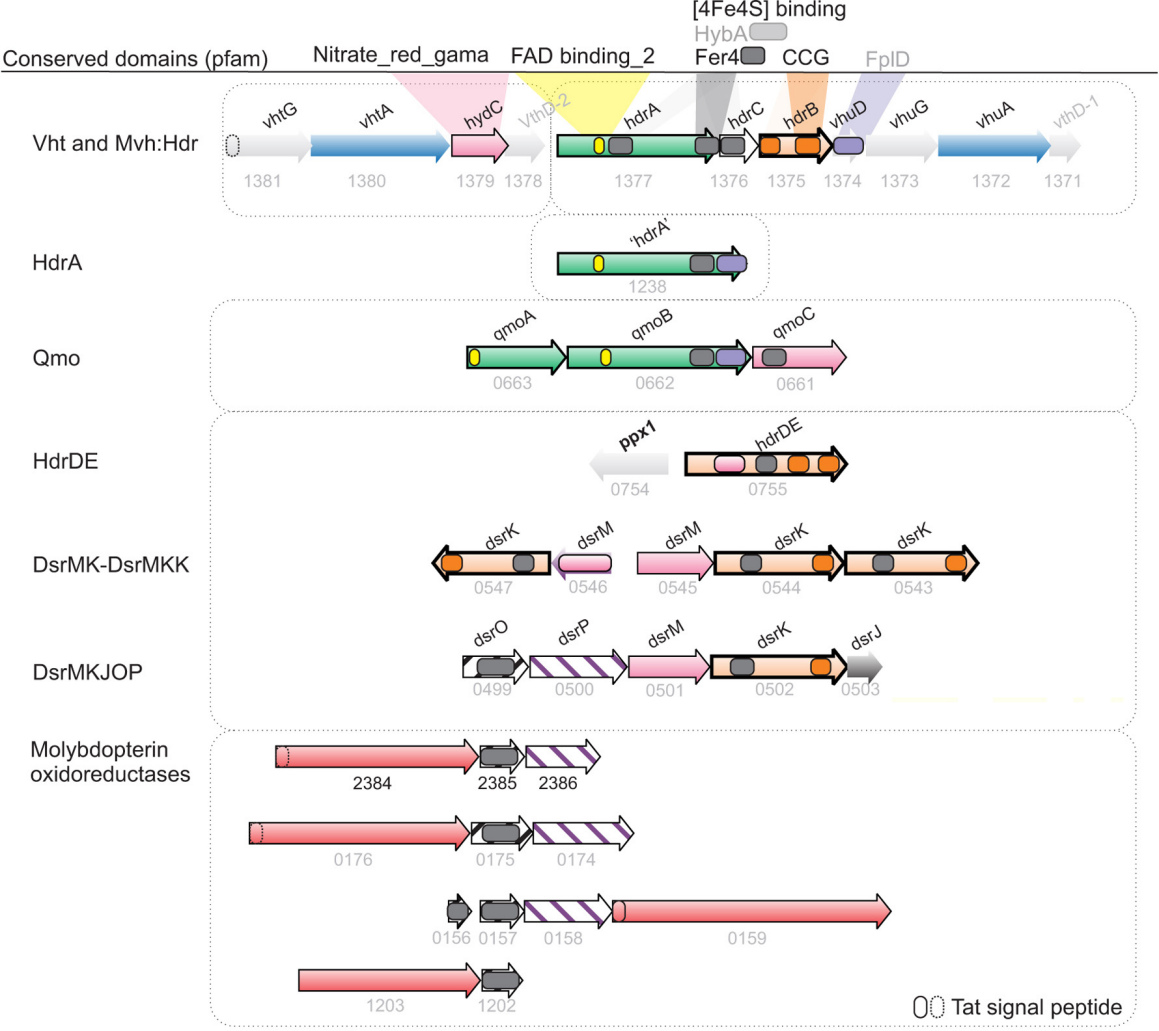
**Synteny and homology between hydrogenases and menaquinol oxidases potentially involved in energy conservation in *A. fulgidus***. Hypothetical bifurcating and ferredoxin interacting genes (green), Menaquinone reductase/Menaquinol oxidase (pink/purple stripes), Heterodisulfide reductase (CCG domain; orange), molybdopterin-binding oxidoreductases; putative thiosulfate/polysulfide/thetrathionate or formate (AF1202-AF1203) dehydrogenase complexes (peach).

Transcriptional up-regulation during growth with thiosulfate (T-L, T-H_2_/CO_2_, Figure 1B) corresponded to significant enrichment of genes in the energy production category (C, Figure 2). Genes regulated more than 1.5 fold during growth with thiosulfate belong to an operon of putative membrane integrated periplasmic thiosulfate reductase (AF2384-AF2386). This cluster is one of three gene clusters (AF0157-AF0160, AF0173-AF0176, AF2384-AF2386, Table 1A, Figure 4) of which protein expression is induced while *A. fulgidus* utilizes (per)chlorate as an electron acceptor (Liebensteiner et al., 2013). A previous study has linked the gene products of AF0157-AF0160 to the twin arginine translocation (Tat) pathway (Coulthurst et al., 2012). The molybdenum-binding subunit, encoded by AF2384, contains a similar Tat signal peptide (BLASTp, TatP 1.0: http://www.cbs.dtu.dk/services/TatP/, Figure 4), indicating a periplasmic location of the thiosulfate reductase.

The second copy of the carbon monoxide dehydrogenase gene *cdhAB-1*; (AF1100-AF1101, Table 1F) (Dai et al., 1998) was unexpectedly highly up-regulated during growth with thiosulfate. Finally, genes of cobalamin/vitamin B_12_ biosynthesis (AF0724-AF0727, AF1843) were specifically up-regulated in cultures utilizing thiosulfate. Vitamin B_12_ is a cofactor in methyl transferases, such as the second subunit of the acetyl-CoA transferase (ACS)/CODH complex (Banerjee and Ragsdale, 2003).

**Figure 5:**
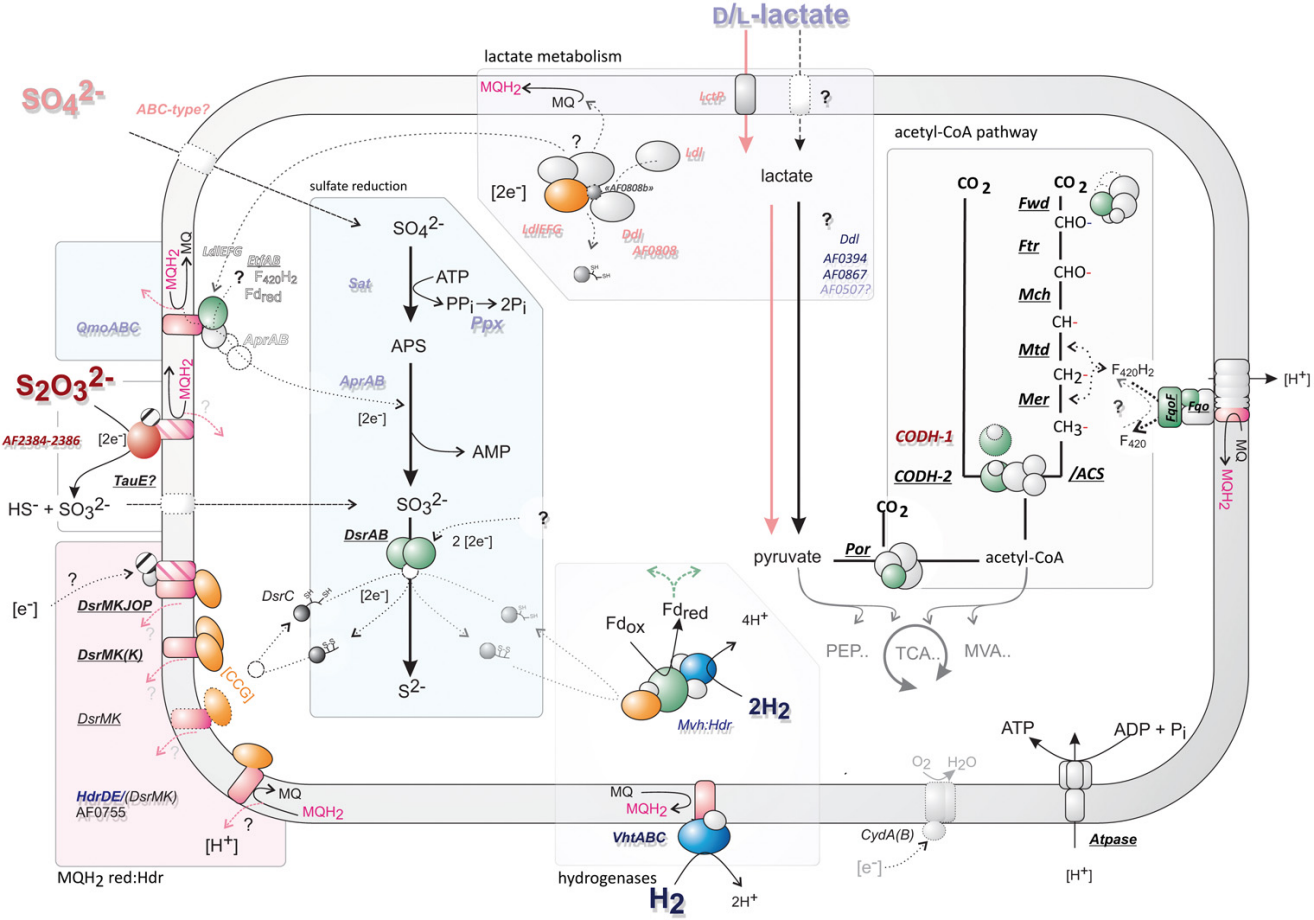
**An overview of the proposed central cellular metabolism of *A. fulgidus***. Gene abbreviations catalyzing reactions are italized; underlined—if constitutively expressed; and bold—if high signal intensity (>3). Gene abbreviations are colored corresponding to regulation of different substrates; H_2_ (dark blue), lactate (light blue), thiosulfate (S_2_O^2−^_3_; dark red), and sulfate (SO^2−^_4_; pink). Cartoons of central enzymes represent a rough outline of structural features, subunits are colored correspondingly: green—represents potential ferredoxin interacting domains; blue—hydrogenases; magenta—menaquinone/quinol interacting subunits, orange; heterodisulfide reductase (with [CCG] domains). Gene names corresponding to this figure can be found in Table 1 and Table S2—for genes of the acetyl-CoA pathway.

Significantly enriched genes up-regulated in the presence of sulfate (S-L, Figure 1B) belong to a specific set of 5 genes categorized under energy production and metabolism (C) corresponding to a region of one lactate permease and 5 putative lactate dehydrogenase (LDH) genes (*lctp*; AF0806, *lldD*; AF0807, *dld*; AF0808 and *lldEFG*; AF0809-AF0811, Table 1E, Figures 3, 5). The genes AF0809-AF0811 may encode an oligomeric LDH (*lldEFG*) based on the presence of conserved domains with bacterial LdlEFG (Figure 3) in *Shewanella oneidensis* MR-1 and *Bacillus subtilis* (Chai et al., 2009; Pinchuk et al., 2009) despite low overall sequence identity (Figure 3). Within the genus *Archaeoglobus*, only *A. fulgidus* and *A. sulfaticallidus* are known to couple lactate oxidation to dissimilatory sulfate reduction (Steinsbu et al., 2010). A genetic comparison of *Archaeoglobales* revealed a corresponding genetic region only in these two species, with a conserved upstream gene encoding a putative regulatory helix turn helix (HTH) motif, and a putative ORF—“AF0808b” homologous to the gene Asulf00640 (Figure 3). The putative ORF, “AF0808b” may encode a protein with a ferredoxin-binding domain.

Other genes up-regulated in the presence of sulfate belong to the COG category defense (V), corresponding to a region CRISPR-associated proteins (AF1861-AF1868, Table S3); the *cmr1–6* genes of RNA guided RNase in *Pyrococcus furiosus* (Hale et al., 2009). The induction of these genes is probably not related to growth rate as T-L cultures had a higher specific growth rate (Figure 1A, Table S3).

### Differentially expressed genes related to growth phase

Up-regulated genes influenced by late log growth (Figure 1B) were distinctly enriched in genes corresponding to information storage and processing. In the categories transcription (K) and replication (L) the genes were differentially expressed below 1.5 fold change (Figure 2, Table S3). Genes for cellular processes and other COG categories: cell wall/membrane/envelope (M), sporulation protein (AF1778. COG - S), histone A1 (AF0337; COG - L), and cell division inhibitor (AF0696; COG - N); were all up-regulated over 1.5 fold in both late-log S-L and late-log T-H_2_/CO_2_ cultures.

Genes corresponding to mid-log belonged to the COG categories of nucleotide transport and metabolism (F), where thymidine phosphorylase (AF1341, AF1342) was highly induced. Also, transcription (K)-related genes were significantly up-regulated, but at minor fold (<1.5). It was therefore evidence of distinct functions related to a shift in log phase, corresponding to transcription, replication and genes for cellular processes. These genes include processes such as replication, histone modification, and metabolism of nucleotides. The functions of genes related to a growth phase specific response, was distinct from that of a shift in energy or electron acceptors, relating to cellular metabolism in general (Figure 2).

### Absolute abundance of transcripts

The genes corresponding to KEGG pathways, such as ribosome (afu03010), oxidative phosphorylation (afu00190), aminoacyl t-RNA synthesis (afu00970), and methane metabolism (afu00680), were enriched amongst genes with high signal abundance. As were genes of general metabolic pathways (afu01100) and biosynthesis of secondary metabolites (afu01110) (Figure S1B).

In the Oxidative phosphorylation pathway (afu00190), genes of the F_420_H_2_: quionone oxidoreductase (Fqo) (AF1823-AF1833) (Kunow et al., 1994; Brüggemann et al., 2000) and the archaeal V-type ATPase (AF1158-AF1168) were continuously expressed above 3 fold average expression (Figure S1A). Genes for dissimilatory sulfate reduction (*sat*, *ppx*, *aps*, and *qmo*), although differentially regulated, were generally expressed at levels above 3 fold average expression levels. The genes related to terminal reduction of sulfite (SO^2−^_3_, Table 1A); *dsrAB* (AF0423-AF0424), membrane-bound heterodisulfide-reductase:quinol-oxidase; *dsrMKJOP* (AF0499-AF0502), and homologous second copy of *dsrMK(K)* (AF0543-AF0545) were constant and highly expressed (Table 1C, Figure S1A-I). The notable exception was that of *dsrD* (AF0425) and *dsrC* transcripts (AF2228), which were expressed at lower levels (Table 1A, Figure S1A) and were both induced in late-log S-L samples. These two genes had a corresponding transcriptional pattern (Pearson's *r*-value; 0.82) despite a large genomic distance (~0.6 Mb).

Genes relating to all steps of the acetyl-CoA pathway were expressed at equivalent and constitutive levels (Table S2): CODH/ACS complex (*cdhAB-2*; AF2397-AF2398/*cdhCDE*;AF0376-AF0379), along with N5,N10-methylenetetrahydromethanopterin reductase (*mtr-1*, AF1066); the F_420_-dependent methylenetetrahydromethanopterin dehydrogenase (*mtd*, AF0714); methenyltetrahydromethanopterin (*mch*; AF1935); formylmethanofuran-tetrahydromethanopterin formyltransferase (*ftr-1*; AF2073 and *ftr-2*; AF2207) and the formylmethanofuran dehydrogenase (*fwdABCD*; AF1928-AF1935). There was therefore no indication of differential regulation relating to the acetyl-CoA pathway of *A. fulgidus* between oxidative (lactate) and reductive (H_2_/CO_2_) processes. Other genes of central metabolic processes related to acetyl-CoA were the constitutively highly expressed pyruvate ferredoxin oxidoreductases (*por*ABDG; AF1669-AF1702, Table S3). This is in line with a bidirectional role of ferredoxin in oxidative and reductive carbon metabolism (Figures 5, 7).

Constitutively highly expressed genes of electron transport flavoproteins (*etfA*; AF0287 and *etfB*; AF0286, Table S1b), may facilitate reactions relating to the homologous ferredoxin reduction by bifurcating butyryl-CoA dehydrogenases/Etf complex in *Clostridium* spp. (Li et al., 2008; Buckel and Thauer, 2013).

The genes AF2378-AF2380 were constitutively highly expressed (Table S3). These genes have previously been linked to syntrophic growth in *D. vulgaris* Hildenborough (Scholten et al., 2007; Fiévet et al., 2011). The proteins encoded by these genes have conserved domains NifX (AF2378) and CbiA superfamilies (AF2380) (NCBI-CDD) (Rubio and Ludden, 2008), indicating a role in the biosynthesis of iron-molybdenum cofactors. These may function in the synthesis of molybdopterin oxidoreductases that are abundant in the genome of *A. fulgidus* (Klenk et al., 1997). Other highly expressed genes of hypothetical proteins are AF1617-AF1619 (Table S3), which contain multiple transmembrane helixes. The adjacent associated PAS domain encoding gene (AF1620) may indicate an unknown regulatory complex.

Finally, the genes of rubrerythrin and desulfoferrodoxin (*rr1*, *rr2*, *dfx*; AF0831-AF0833) were expressed continuously at high abundance (Table S3). These are putative oxidoreductases, and probably function in elimination of superoxides (Rodrigues et al., 2005). Genes of cytochrome bc oxidase (*cydA*; AF2296, AF2297, Table S3) were highly expressed during all conditions. As in *Desulfovibrio* spp. (Ramel et al., 2013), this may allow coupling the menaquinone pool to oxygen reduction in *A. fulgidus* (Figure 5).

## Discussion

In the present work, a model of the energy metabolism in *A. fulgidus* for the utilization of lactate and hydrogen with thiosulfate or sulfate as terminal electron acceptors is presented based on transcriptome profiling.

### Lactate metabolism

Lactate is the “classical” substrate of sulfate reducers, and its link to energy conservation in *Desulfovibrio* has been the subject of intense study (Keller and Wall, 2011). Several transcriptional shifts were observed in *A. fulgidus*, involving expression of LDH and putative LDH genes (Table 1E, Figure 3). Our results indicate that during growth with T-L, activity of multiple LDH isozymes (Figures 3, 5) may occur in *A. fulgidus*, as suggested in *D. vulgaris* (Keller and Wall, 2011). When sulfate is used as an electron acceptor, oligomeric LdlEFG may operate together with monomeric lldD and dld in the oxidation of lactate (Figure 5). The conserved “modular” domain composition of the proteins encoded by the genes dld, the ORF “AF0808” and AF0809, may facilitate a multimeric complex that functions as monomeric homologs encoded in other species (Dvu3071, Figures 3, 5).

The presence of a gene cluster with identical arrangement in the lactate utilizing *A. sulfaticallidus* and *A. fulgidus*, supports a potential role of the LdlEFG in linking lactate oxidation with sulfate reduction in *A. fulgidus*. Acquiring the *lldEFG* gene cluster may have been essential for *Archaeoglobales* in order to perform dissimilatory sulfate reduction with lactate as an energy source, potentially via the QmoABC complex to APS reductase.

Oligomeric lldEFG is widely distributed in Bacteria, including sulfate-reducing *Deltaproteobacteria* (Pinchuk et al., 2009; Pereira et al., 2011), but has previously not been identified in Archaea. Various functions have, however, been suggested for LdlEFG in Bacteria. In *S. oneidensis* MR-1, the LdlEFG is found to stimulate the activity of Dld-II (Figure 3), indicating a functional relationship (Pinchuk et al., 2009). Interestingly, in *D. alaskensis* the LdlEFG is required in syntrophic growth with *Methanococcus* (Meyer et al., 2013). In this model an LdhAB-1 (GplCD) catalyses the primary oxidation of lactate, and transfers electrons, possibly through thiol/disulfide, to an LdlEFG homologous complex. The LdlEFG may transfer electrons to the QmoABC complex, which facilitates menaquinol reduction (Meyer et al., 2013). However, the LdlEFG is also present in species without a QmoABC complex and functions independently as a membrane associated l-LDH capable of reducing quinone (Chai et al., 2009; Pinchuk et al., 2009; Thomas et al., 2011). In order to verify the specific role of the LdlEFG homologs in *A. fulgidus*, biochemical studies are required (enzyme activity and protein-protein interaction) to understand its relation to Qmo and energy conservation. Perhaps, prior to construction of deletion mutants as a genetic system is not yet available for this species.

With the exception of the cdhAB-1 (see next section), genes encoding the acetyl-CoA pathway were constitutively expressed at high levels (Table S2). This was also true for the F_420_H_2_: quinone oxidoreductase (Fqo) complex, which probably catalyzes proton translocation utilizing F_420_H_2_ generated by the oxidative acetyl-CoA pathway (Brüggemann et al., 2000). The hydrogenases in *A. fulgidus* were specifically induced during growth with hydrogen, and low transcriptional expression of hydrogenases was observed during growth on lactate (Table 1). Therefore, it may be questioned whether “hydrogen cycling” (Odom and Peck, 1981; Kulkarni et al., 2009) is used as a mechanism for energy conservation with lactate as the energy source. This would emphasize the role of the respiratory Fqo complex and a menaquinone-based respiratory system (Figure 5) in energy conservation in *A. fulgidus* during growth with lactate.

Several distinct putative menaquinol oxidase:Hdr complexes are present in the genome of *A. fulgidus*. The DsrMKJOP (AF0499-AF503) complex and the DsrMK(K) (AF0543-AF0544) were constitutively highly expressed (Table 1C), whereas a second DsrMK (AF0543-AF0544) was expressed at average expression levels (Table 1C). Multiple membrane-bound DsrMK complexes may therefore oxidize the menaquinol (MQH_2_) generated by the Fqo complex (Figure 5).

The DsrK components may transfer electrons to DsrC by breaking the disulfide bonds between the two C—terminal cysteines of the enzyme (Mander et al., 2005; Oliveira et al., 2008; Grein et al., 2010). The *dsrC* gene (AF2228) is, however, expressed at average transcriptional abundance vs. high transcriptional abundance for *dsrAB* (Table 1C, Figure S1). This is lower than previously estimated in *D. vulgaris*, where the gene of *dsrC* is expressed at high levels (Wall et al., 2008). Although these values are more rigorously estimated in the previous study, our results point toward a lower expression ratio between *dsrAB* and *dsrC* in *A. fulgidus*. This may indicate involvement of additional electron transport components from Hdr to DsrAB. However, other electron carrying proteins, such as ferredoxin, are expressed at similar levels as *dsrC* (*fdx*, <1.4 average expression, Table S3). The true significance of the role of electron flow via DsrC requires further evaluation on translational level.

### Reduction of thiosulfate

The mechanism of thiosulfate reduction and the import of sulfate for cytoplasmatic reduction is uncharacterized in *A. fulgidus*. The specific growth rate of *A. fulgidus* cultivated with lactate was increased by the utilization of thiosulfate, vs. sulfate, as terminal electron acceptor (Figure 1A). The reduction of thiosulfate is thermodynamically favorable (Badziong and Thauer, 1978). However, utilization of thiosulfate vs. sulfate is reported as inhibiting for growth rate in *D. vulgaris* Hildenborough, and has been attributed to the toxicity of increasing intracellular concentrations of sulfite (Badziong and Thauer, 1978; Pereira et al., 2008). The genes corresponding to thiosulfate reductase in *A. fulgidus* are identified by specific up-regulation of a molybdopterin oxidoreductase (AF2384-AF2386, Table 1A). This reductase is active during both lactate and H_2_-oxidation, and is probably a membrane-integrated complex with a periplasmic facing active site (Figure 5). The presence of a Tat signal peptide (Figure 4) indicates that export is facilitated by the twin arginine translocation pathway (Coulthurst et al., 2012).

A periplasmic reduction of thiosulfate may exclude the build-up of toxic intracellular levels of sulfite and may partly explain the high growth rate observed for *A. fulgidus* during cultivation with lactate and thiosulfate. It is unlikely that the reduction of thiosulfate to sulfite (E°′ −402 mV) contributes to energy conservation (Badziong and Thauer, 1978; Stoffels et al., 2012). Rather, final intracellular reduction of SO^2−^_3_ to S^2−^ (E°′ −116 mV) has a redox potential sufficient for energy conservation (Thauer et al., 2007). In most *Desulfovibrio* spp., an indistinguishable ion gradient symport has been found for thiosulfate and sulfate (Cypionka, 1987; Stahlmann et al., 1991). However, such a mechanism has not been identified in *A. fulgidus* (Rabus et al., 2006). The genes previously annotated “sulfate ABC transporter permease” (AF0092-AF0094) are probably a molybdate-specific transporter (Klenk et al., 1997; Hollenstein et al., 2007). The induction of genes corresponding to a ABC-type transport system (AF1136-AF1138) during growth with sulfate (S-L) may ambiguously be assigned as a putative sulfate transporter, as these genes are also induced during growth with lactate (Table 1B, Figure 5). The gene *tauE* is proposed to encode a sulfite exporter in *Cupriavidus necator* (*Ralstonia eutropha*) during sulfoacetaldehyde degradation (Weinitschke et al., 2007). The constitutive highly expressed *tauE* homolog (AF1562) may be assigned a putative function for sulfite import in *A. fulgidus* (Table 1B, Figure 5). The utilization of thiosulfate is a common property of all *Archaeoglobus* spp. and *Ferroglobus placidus*, however, homologous of the putative periplasmic AF2384-AF2386 gene cluster can only be found in the species *A. fulgidus* and *F. placidus* (BLASTp, Absynte, and Syntax-tools Oberto, 2013). The DsrAB of *A. fulgidus* displays a high level of *in vitro* thiosulfate reductase activity (Parey et al., 2010) and may play a role as a parallel process of cytoplasmatic thiosulfate reductase. Although a common trait, different *Archaeoglobus* spp. seem to have diverging enzyme systems for thiosulfate reduction.

Unexpectedly, when thiosulfate was substituted for sulfate as electron-acceptor, a second copy of the cdhAB-1 subunits in the ACS/CODH complex was induced (AF1100-AF1101, Table 1F, Figure 5). The specific regulation corresponding to terminal electron acceptor (thiosulfate) may indicate a preferential utilization of different electron carriers between dissimilatory sulfate and thiosulfate reduction. A similar shift in genes of cobalamin/vitamin B_12_ biosynthesis may also affect the function of the ACS subunit (Banerjee and Ragsdale, 2003). Previous studies have shown that regulation of CODH/ACS complexes in *M. acetivorans* are related to carbon source (Matschiavelli et al., 2012).

### Hydrogen metabolism

*Archaeoglobus fulgidus* possesses only two hydrogenases; the periplasmic Vht hydrogenase and the soluble Mvh:Hdl (Mander et al., 2004). The latter may take part both in energy conservation and in generation of Fd_red_ for CO_2_—fixation through the acetyl-CoA pathway. The reductive acetyl-CoA pathway requires at least 3 mol Fd_red_ for the generation of one mole pyruvate from CO_2_ (Fuchs, 2011). Similar to the methanogens, a bifurcation reaction is obligate in *A. fulgidus* for the generation of Fd_red_ while growing autotrophically with hydrogen. In addition, Fd_red_ can be hypothesized as an electron donor to APS reductase, through the QmoABC (Ramos et al., 2012) and the DsrAB (Oliveira et al., 2008, 2011) offering a potential coupling between ferredoxin and electron transport phosphorylation.

**Figure 6:**
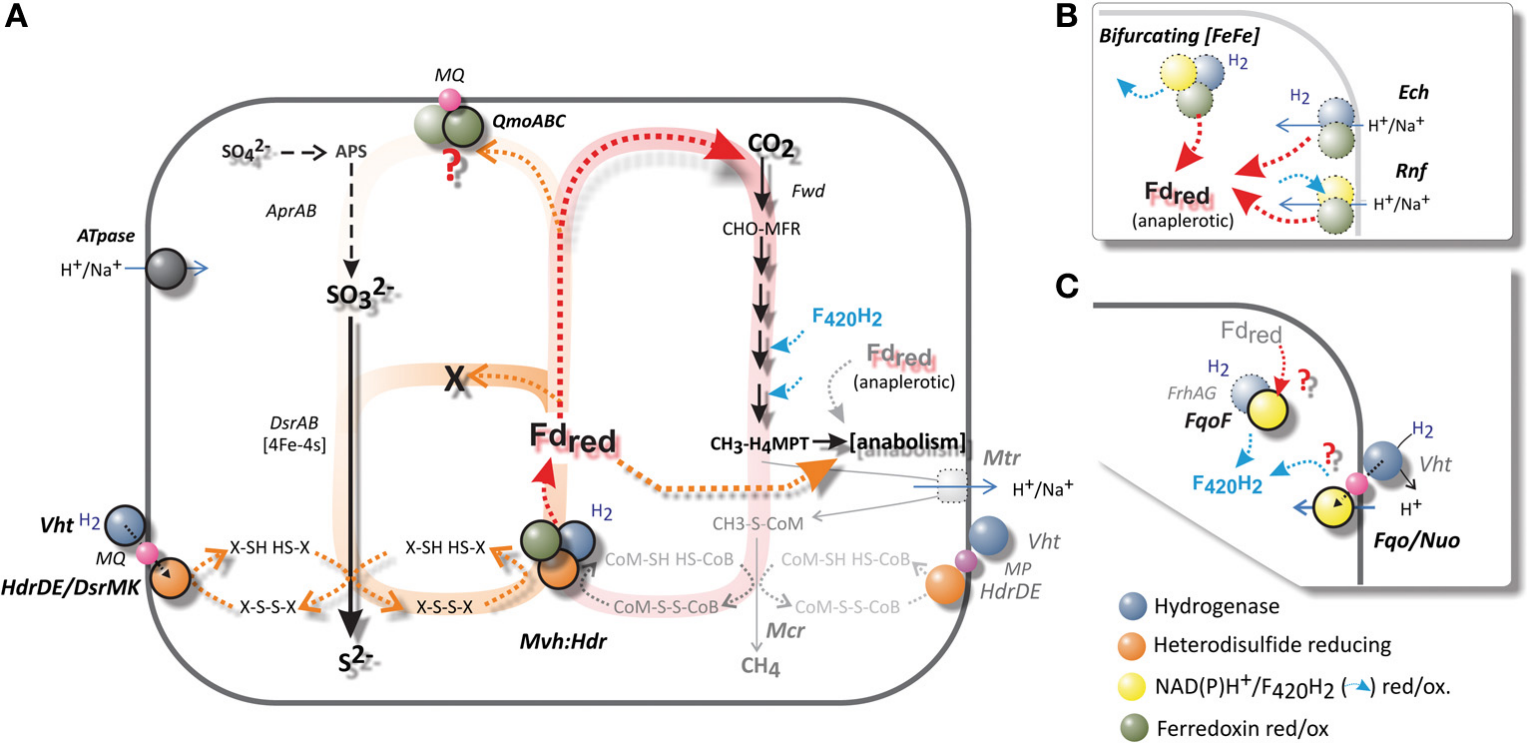
**(A) A schematic comparison of hydrogenotrophic and methanogenic metabolism with that of *A. fulgidus*.** The Wolfe cycle of autotrophic hydrogenotrophic methanogens without cytochromes is shaded in red. The Mvh:Hdr is the only known ferredoxin-reducing complex present in *A. fulgidus*. A pathway analogs to the Wolfe cycle is indicated in orange—if Fd_red_ is required for either APS or SO^2−^_3_ reduction, prior to the reduction of di-thiol (X-SH HS-X), an anaplerotic ferredoxin-reducing hydrogenase is required. **(B)** An overview of alternative complexes for Fd reduction (dotted borders), which are absent in *A. fulgidus*. Most methanogens and SRP maintain several of these complexes. The Wolfe cycle requires an anaplerotic ferredoxin (Fd)-reducing hydrogenase (Ech/Eha), in order to fixate carbon in anabolic processes. **(C)** Putative mechanisms for generation of F_420_H_2_. Complexes absent in *A. fulgidus* outlined in **(B)**: the electron bifurcating [Fe-Fe] hydrogenase (Huang et al., 2012), the Rnf complex (Biegel et al., 2011; Tremblay et al., 2013), the energy converting hydrogenase (Ech), **(C)**: the F_420_-reducing hydrogenase (Frh) (Thauer et al., 2010).

The genes of periplasmic Vht hydrogenase represented the highest transcriptional shift of any genes in relation to growth on T-H_2_/CO_2_ and was expressed at a high level relative to average signal abundance (Table 1D, Figures 4, 5). The resulting two protons from a periplasmic hydrogenase reaction catalyzed by Vht may contribute directly to the formation of *pmf* during growth. In *Methanomicrobiales*, the Vht hydrogenase homolog donates electrons via methanophenazine (MP) to a cytoplasmic-facing, membrane-bound Hdr (HdrDE, Figure 6A) (Deppenmeier et al., 1992; Ide et al., 1999; Thauer et al., 2010). Similarly, the VhtABC complex in *A. fulgidus* may reduce menaquinone (MQ). A subsequent menaquinol (MQH_2_) oxidation, facing the periplasm, by a membrane-bound Hdr may translocate two protons (Figures 5, 6A). The observed co-induction of *vht* hydrogenase genes and a fused *hdrDE* homolog encoding dual [CCG] domains (AF0755, Figure 4) suggests a close physical interaction between the two encoded complexes that may form a distinct path of electron flow to DsrAB. However, the genes of DsrMKJOP or DsrMK(K) were constitutively expressed, and therefore, electron flow is also possible via these complexes (Table 1, Figures 4, 5). The reaction probably represents the major pathway of energy conservation during growth with H_2_.

In contrast to most methanogens and SRP, *A. fulgidus* possesses only one potential mechanism for ferredoxin generation from hydrogen; namely the Mvh:Hdl(Hdr) catalyzed reaction (Klenk et al., 1997; Thauer et al., 2010; Pereira et al., 2011) (Figure 6A). The Mvh:Hdr hydrogenase is one of, so far, 4 perceived reaction mechanisms for the reduction of ferredoxin from H_2_ during autotrophic growth (Fuchs, 2011) (Figures 6A,B). In methanogens the Mvh:Hdr is the key enzyme of the recently named Wolfe cycle (Thauer, 2012), which catalyses the crucial bifurcation reaction that couples the first (Fd_red_ is required for the fixation of CO_2_) and last step of methanogenesis (reduction of heterodisulfide, CoM-S-S-CoB). No net Fd_red_ is generated from this reaction, as generation of Fd_red_ and heterodisulfide reduction are interdependent (Figure 6A). In order to assimilate carbon—an anaplerotic hydrogenase—the energy-conserving membrane-associated hydrogenase (Ech) is required in these methanogens for additional generation of Fd_red_ for anabolic processes (Figure 6B) (Lie et al., 2012; Thauer, 2012).

The presence of ferredoxin-binding sites ([4Fe-4S] clusters) in the structures of DsrAB indicate that soluble Fd_red_ or a ferredoxin reductase complex may facilitate the steps of two-electron transfer to SO^2−^_3_; from redox state +IV, to +II and 0 (Schiffer et al., 2008; Oliveira et al., 2011). However, if the reduction of sulfite required 2 mol Fd_red_ prior to reduction by 1 mol reduced DsrC, the pool of available oxidized DsrC would soon be depleted (Figure 6A). Therefore, disulfide (X-S-S-X) would not be available for recycling Fd_*ox*_ to Fd_red_ by Mvh:Hdl mediated bifurcation.If the reduction of sulfite was dependent on only 1 Fd_red_, an anaplerotic hydrogenase would still be required for the generation of Fd_red_ for subsequent CO_2_ fixation; analogous to the Wolfe cycle (Thauer, 2012). During growth with hydrogen, the absence of an anaplerotic ferredoxin reductase in *A. fulgidus* requires multiple two-electron transfers for the reduction of sulfite by other mechanisms, either by an unknown electron donor or repetitive association, oxidation and dissociation of DsrC.

According to this model Fd_red_ is not a viable electron donor for reduction of sulfite in *A. fulgidus* during growth with H_2_. The electrons for reduction of sulfite must therefore be provided by the Vht hydrogenase (Figure 6A). Hence, Fd_red_ is probably utilized in biosynthesis rather than energy conservation during growth with T-H_2_, and may be a plausible explanation to the low transcriptional levels of *mvh:hdl* (Table 1D, Figure 6A).

Requirement of a Fd_red_-driven “confurcation” mechanism via the QmoABC complex for APS reduction in *A. fulgidus* would according to our model inhibit fixation of CO_2_ (Figure 6). Accordingly, no growth with sulfate and hydrogen (S-H_2_/CO_2_) has been observed for *A. fulgidus* (Stetter et al., 1987; Steinsbu et al., 2010). While growth on sulfate with hydrogen (S-H_2_) does not occur; *A. fulgidus* is capable of utilizing sulfate as terminal electron acceptor with CO or formate as electron donors (Stetter et al., 1987; Henstra et al., 2007). The redox potential of CO/CO_2_ indicates the capacity to reduce ferredoxin directly without the need for a bifurcation reaction (Thauer et al., 2007). The redox potential of formate/CO_2_ is similar to that of hydrogen (Thauer et al., 2007), and requires bifurcation for the generation of Fd_red_. In addition, *A. sulfaticallidus* grows on S-H_2_/CO_2_ (Steinsbu et al., 2010) and genome analysis of *A. sulfaticallidus* (Stokke et al., 2013), did not provide an alternative mechanism of Fd_red_ generation.

An unknown cytoplasmic formate dehydrogenase (AF1203-AF1202, Figure 4; Henstra et al., 2007) probably associates with the HdrA subunit of the Mvh/Hdl complex in order to catalyze the reduction of ferredoxin (Lie et al., 2012). In order to escape the proposed physiological impasse of Fd_red_ as an intermediate of APS reduction, a formate dehydrogenase would also be needed to associate with QmoB (a HdrA homolog, Figure 4) and drive energy conservation by a confurcation mechanism. An unknown mechanism may also be facilitated by the gene product of AF1238 (Figure 4). Similarly, in *A. sulfaticallidus*, the Mvh hydrogenase subunit may, tentatively, form two complexes; one with HdrA and one with a homologous QmoB.

Considering growth with formate and sulfate, and the similar genomic composition of *A. fulgidus* and *A. sulfaticallidus*; the most plausible explanation for the inability of *A. fulgidus* to grow on S-H_2_ is a regulatory link between hydrogen and observed down-regulation of a pyrophosphatase gene (*ppx*, AF0756). Additionally, we observed minor down-regulation of genes corresponding to the Sat-ORF2-AprAB operon (Table 1A, Figure 5). A reduced expression of Ppx would inhibit or limit the formation of APS, as the pyrophosphatase reaction drives the total reaction to completion (Peck, 1962). Uniquely for *A. fulgidus*, the *ppx* gene is close to the inversely induced membrane-bound Hdr gene (*hdrDE*; AF0755) located on the opposite strand (Tables 1A,C, Figure 4), which may suggest a regulatory link. Therefore, the inability of *A. fulgidus* to grow with sulfate and H_2_ may relate to transcriptional regulation rather than a physiological limitation.

Clearly, further biochemical characterization is needed to verify the proposed regulatory mechanism. There is also a need to characterize a mechanism for growth with sulfate and formate for *A. fulgidus*, and S-H_2_ for *A. sulfaticallidus* (Figure 6A).

### Generation of F_420_H_2_ in the absence of a dedicated hydrogenase (Frh)

*Archaeoglobus fulgidus* lacks the cytoplasmic F_420_-reducing hydrogenase (FrhABG) that catalyzes the reduction of F_420_ in most methanogens (Alex et al., 1990; Thauer et al., 2010) (Figure 6C) Therefore, mechanism for generating the reduced F_420_H_2_ required for carbon fixation through the reductive acetyl-CoA is unknown (Figure 5). A negligible role of NAD(P)H is supported by low expression levels of F_420_H_2_:NADP^+^ oxidoreductase genes in our study (AF0892, AF1209; Table S3).

Independently of the Fpo complex and Frh hydrogenase; the FpoF subunit is shown to reduce F_420_ coupled with oxidation of Fd_red_ in *M. mazei* (Welte and Deppenmeier, 2011). It is therefore possible that FqoF in *A. fulgidus* catalyzes a similar mechanism for the generation of reduced F_420_ (Figure 6C, *fqoF*: AF1833, Table S2). The required Fd_red_ must be provided by the bifurcation reaction facilitated by the Mvh:Hdl hydrogenase (Figures 6A,B). As discussed in the previous section, the main route of energy conservation is probably provided by the periplasmic hydrogenase. The low transcriptional levels of *mvh:hdl* may be a reflection of translational levels of Mvh:Hdl hydrogenase, if an alternative pathway of F_420_ reduction is present independently of Fd_red_.

Vorholt et al. (1995) suggested the possibility that reduced F_420_H_2_ may be generated by reverse electron flow through menaquinol oxidation. The Fqo complex, including the FqoF subunit, is also a potential MQH_2_ oxidase (Figures 5, 6C). The constitutive expression of the entire Fqo complex indicates that the complex may function in reverse as a *pmf* (μΔH^+^)-dependent menaquinol—F_420_ oxidoreductase, where the menaquinol (E°′ −75 mV) may donate electrons for the reduction of F_420_ (E°′ −360 mV). The resulting positive redox potential (E°′ +285 mV) may be overcome in a process assisted by the consumption of proton gradient. Further support for such a mechanism can be found in the common ancestry of the respiratory complex I and Ech hydrogenase (Hedderich, 2004; Moparthi and Hägerhäll, 2011). In mitochondria and iron-oxidizing *Thiobacillus ferrooxidans* the NAD(P)H-oxidoreductase (Complex I) has been shown to catalyze this reaction at the expense of ATP hydrolysis, which is perceived to be coupled to generation of a *pmf* by reversal of ATPase (Chance and Hollunger, 1960; Vinogradov, 1998; Elbehti et al., 2000). The Eha/Ech hydrogenase activity is linked to the reduction of ferredoxin (Figure 6B) (Meuer et al., 1999), as the reverse electron flow of the Ech dehydrogenase catalyzes the formation of CO from CO_2_, and H_2_ by consumption of *pmf* (Bott et al., 1986; Bott and Thauer, 1987; Lie et al., 2012). Therefore, the reduction of Fd_red_ (E°′ −500 mV) with H_2_ (E°′ −414 mV; −300 mV at 10 Pa H_2_) results in a positive redox potential (E°′) of at least +86 mV (or +200 mV at 10 Pa H_2_) and is considered possible with the utilization of a proton gradient (Figure 6B) (Thauer et al., 2007).

It remains to be shown in *A. fulgidus* whether it is possible to drive the reduction of F_420_ by MQH_2_ oxidation (E°′ +280 mV) and a proton gradient by e.g., constructing deletion mutants of *fqo* genes encoding MQ interacting components, or biochemical characterization by inverted vesicles (Baumer et al., 2000) coupled to ATP hydrolysis.

### Co-assimilation of organic substrates

**Figure 7:**
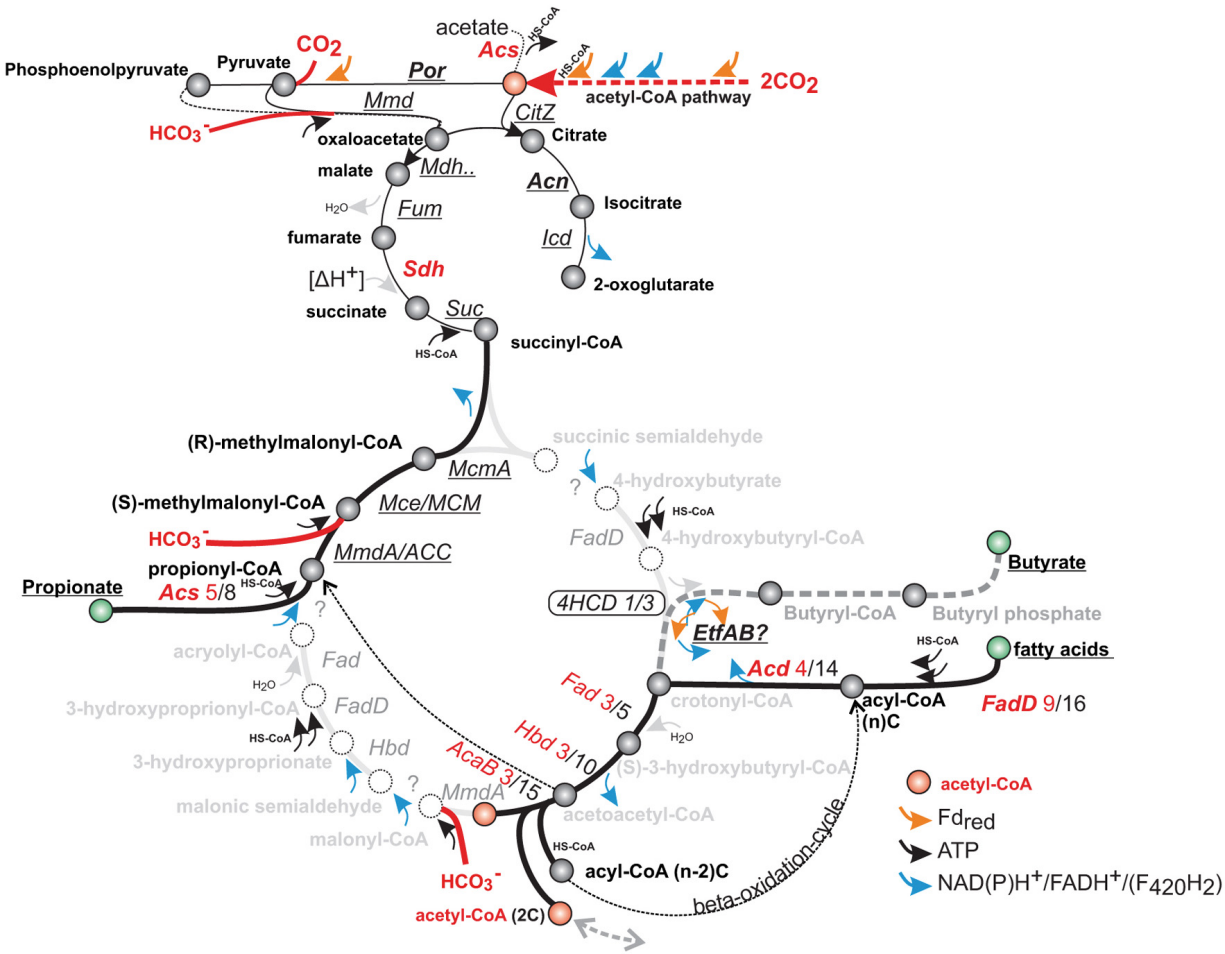
**Genes corresponding to fatty acid and propionate metabolism, compared with putative steps in the 4-hydroxybutyryl (4HB) and 3-hydroxyproprionyl (3HP) pathway of autotrophic Creanarchaea**. Homologous genes correspond to several steps in the 4HCD and 3HP pathway (intermediate names in gray), and may not be easily-distinguished by homology alone. Genes of the acetyl-CoA pathway are expressed at a higher level than the key enzymes of putative 4-hydroxybutyryl-CoA dehydratase (4HCD), which are not uniformly induced. Genes that are not induced by T-H_2_/CO_2_ are underlined, for up-regulated genes—the number of up-regulated vs. total number of homologs is indicated (genes are annotated in Table S1).

The ambiguous roles of genes corresponding to fatty acid metabolism (COG; I, Figures 2, 7) during growth with H_2_ is discussed. Putative co-assimilation of organic acids is considered more likely than a homologous secondary carbon fixation pathway, although, both mechanisms may contribute to conserve Fd_red_ during autotrophic growth with H_2_.

The genes of methylmalonyl metabolism (AF2215-AF2219, Table S1b, Figure 7) were continuously highly expressed. These enzymes may serve as a pathway of propionate degradation (Takaki et al., 2010; Moon et al., 2012). During growth with T-H_2_/CO_2_, several genes related to fatty acid biosynthesis and metabolism were induced (COG; I, Figure 2, Table S1a). Expression of the genes may be affected by trace amounts of fatty acids present in yeast extract (0.03% weight) and points to a potential for fatty acid scavenging/co-assimilation of organic substrates during autotrophic growth (Klenk et al., 1997; Zarzycki and Fuchs, 2011).

Recently, Parthasarathy et al. (2013) demonstrated induced activity of phenylalanine degradation in the presence of yeast extract. Despite amino acids being a major component of yeast extract, none of the putative genes reported in the previous study (Table 2 in Parthasarathy et al., 2013) were induced during T-H_2_/CO_2_ growth in this study. Thus, there is no uniform induction of putative scavenging mechanisms for organic carbon during autotrophic growth.

The genes related to fatty acid biosynthesis also encode enzymes in the 3-hydroxypropionate/4-hydroxybutyrate (3HP/4HB) cycle identified in *Metallosphaera sedula*, and could potentially represent a secondary carbon fixation pathway in *A. fulgidus* (Berg et al., 2007; Estelmann et al., 2011). The genome of *A. fulgidus* includes 3 homologs of the 4-hydroxybutyryl-CoA dehydratase (*4hcd*), (AF0333, AF0885 and AF1027, Table S1c), which is a key enzyme of the 4-hydroxybutyrate carbon dioxide assimilation pathway (Figure 7) (Berg et al., 2007). During growth with T-H_2_/CO_2_, one of the *4hcd* homologs (AF0885) was induced (1.5 fold). The induced gene displayed average signal intensity (1.3), and was expressed at a similar level as the two other unregulated homologs (AF0333 and AF1027). The differential transcriptional expression of *4hcd* (Msed1321) in *M. sedula* was related to autotrophic vs. heterotrophic growth and resulted in a more than 7 fold up-regulation (Auernik and Kelly, 2010). The presence of a 3HP/4HB cycle was refuted by Estelmann et al. (2011) by a subsequent study on the obligate autotroph “*A. litotrophicus*,” where enzyme activity of key processes could not be detected. The presence of 5 homologs of *A. fulgidus 4hcd* in the genome of *Desulfatibacillum alkenivorans* suggest that this enzyme is involved in alkene degradation in these species (Estelmann et al., 2011). Analogously to *A. fulgidus*, the facultative autotroph and fatty-acid and alkene degrading *D. alkenivorans* utilizes the bacterial acetyl-CoA/Wood-Ljungdal pathway (So and Young, 1999; Callaghan et al., 2012), indicating physiological similarities between the distantly related species.

Alternative mechanisms for up-regulation of homologous of propionate and beta-oxidation may be co-assimilation of organic substrates that may supplement the reductive acetyl-CoA pathway. In the light of the considerations of the role of Fd_red_ during growth with T-H_2_/CO_2_, this may provide a significant advantage by supplementing reduction of CO_2_ with the uptake of reduced organic acids. The constitutively highly expressed EtfAB may also provide a source of Fd_red_ by an unknown bifurcation reaction from ambient fatty or amino acids (Buckel and Thauer, 2013; Parthasarathy et al., 2013). In summary, our data may add support to the theory that the 3HB/4HP cycle may have originated from a heterotrophic pathway; or as a co-assimilatory pathway in Archaea (Fuchs, 2011; Zarzycki and Fuchs, 2011).

### Conflict of interest statement

The authors declare that the research was conducted in the absence of any commercial or financial relationships that could be construed as a potential conflict of interest.

## Supplementary material

Figure S1 **Distribution of array abundance and correlation with and central metabolic processes. (A)** Distribution of intensity of quantile-normalized data; (**A-I**) distribution of values corresponding to minimum expression per gene and (**A-II**) maximum expression per gene. **(B)** Gene set enrichment analysis (GSEA) (Subramanian et al., 2005) on the distribution of selected genes on a list of genes sorted by minimum intensity values (i, corresponding to pie chart **A-I**), i.e., deviation from a random distribution, is displayed for identified peptides and genes corresponding to KEGG pathways. The maximum enrichment score (ES) corresponds to the largest deviance from random distribution, the region above the maximum enrichment score corresponds to the leading edge (LE), this region is colored corresponding to the intensity at point of ES. The numbers denote—[values below LE/above LE].
