# Supplementary material for: Identification of key components in the energy metabolism of the hyperthermophilic sulfate-reducing archaeon Archaeoglobus fulgidus by transcriptome analyses
Source: Front Microbiol. 2014 Mar 11;5:95. doi: 10.3389/fmicb.2014.00095 (PMC3949148; doi:10.3389/fmicb.2014.00095)
Supplement: Figure S1 — Distribution of array abundance and correlation with and central metabolic processes. (A) Distribution of intensity of quantile-normalized data; (A-I) distribution of values corresponding to minimum expression per gene and (A-II) maximum expression per gene. (B) Gene set enrichment analysis (GSEA) (Subramanian et al., 2005) on the distribution of selected genes on a list of genes sorted by minimum intensity values (i, corresponding to pie chart A-I), i.e., deviation from a random distribution, is displayed for identified peptides and genes corresponding to KEGG pathways. The maximum enrichment score (ES) corresponds to the largest deviance from random distribution, the region above the maximum enrichment score corresponds to the leading edge (LE), this region is colored corresponding to the intensity at point of ES. The numbers denote—[values below LE/above LE]. [file Presentation1.ZIP › 58387_Steen_Data_Sheet_1.PDF]

Table S1a. Differential expression (fold change) of genes induced by T-H_2/CO_2, corresponding to fatty acid metabolism and transport (COG: I), graphs display transcriptional abundance, as expression relative to mean

					Differential expression (ANOVA)			Expression level relative to mean				
Locus tag	Locus	NCBI annotation (*arCOG; Wolf et al., 2012)	COG	strand	H_2 vs. Lactate	Thiosulfate vs. Sulfate	late-log vs. mid-log	S-L	late-log S-L	T-L	T-H_2/CO_2	late-log T-H_2/CO_2
AF0033	acaA-1	acyl carrier protein synthase	I	+	**2.87**			0.1	0.1	0.1	0.4 ± 0.1	0.4
AF0089	fadD-1	long-chain-fatty-acid--CoA ligase	I	+	**4.71**			0.1	0.8 ± 0.1	0.1	1.5 ± 0.1	1.8
AF0134	acaB-4	3-ketoacyl-CoA thiolase	I	-	1.31			0.2	0.2	0.2	0.2	0.3
AF0196	alkK-1	medium-chain acyl-CoA ligase	I	+	1.43			0.1	0.1	0.1	0.1	0.1
AF0262	alkK-2	medium-chain acyl-CoA ligase	I	-	**6.78**			0.1	0.2	0.1	0.7 ± 0.1	0.9 ± 0.1
AF0366	acs-2	acetyl-CoA synthetase	I	+	**2.08**			0.2	0.2	0.1	0.4 ± 0.1	0.4
AF0677	acs-3	acetyl-CoA synthetase	I	-	**2.84**			0.5	0.6	0.5	1.4 ± 0.1	1.8 ± 0.3
AF0685	fad-2	enoyl-CoA hydratase	I	+	**1.57**			0.1	0.2	0.2	0.3	0.3
AF0840	fadD-4	long-chain-fatty-acid--CoA ligase	I	+	**3.51**			0.1	0.9	0.1	1.2 ± 0.1	1.5
AF0845	acd-5	acyl-CoA dehydrogenase	I	-	1.47			0.1	0.1	0.1	0.2	0.2
AF0963	fad-3	enoyl-CoA hydratase	I	-	**2.00**	**2.04**		1.2	1.1 ± 0.1	1.7 ± 0.1	2.7	2.8 ± 0.2
AF0964	acd-6	acyl-CoA dehydrogenase	I	-	**1.57**	**1.60**		1.9 ± 0.1	1.6 ± 0.2	2.3	**3.1**	**3.2 ± 0.2**
AF0968	acaB-10	acetyl-CoA acetyltransferase	I	-	**1.60**	**1.73**		1.6 ± 0.2	1.3 ± 0.1	2.2	**3 ± 0.2**	2.7 ± 0.2
AF0975	acs-4	acetyl-CoA synthetase	I	+	**3.28**			0.7 ± 0.1	1.4	0.8	**3.3**	**3.3**
AF0976	acs-5	acetyl-CoA synthetase	I	+	**2.46**			1.1 ± 0.1	1.9 ± 0.1	1.1	**3.4**	**3.4**
AF0991	gcdH	glutaryl-CoA dehydrogenase	I	-	**1.77**			1.1 ± 0.1	1.8 ± 0.2	1.1 ± 0.1	2.7 ± 0.2	2.4
AF1029	fadD-5	long-chain-fatty-acid--CoA ligase	I	-	1.46			0.5 ± 0.1	0.6 ± 0.1	0.5 ± 0.1	1 ± 0.2	0.8
AF1122	hbd-5	3-hydroxyacyl-CoA dehydrogenase	I	+	**2.43**			0.5	0.8 ± 0.1	0.4	1.6 ± 0.2	1.4 ± 0.1
AF1123		Poly(3-hydroxyalkanoate) synthetase*	I	+	1.29			0	0	0	0.1	0.1
AF1174		Putative sterol carrier protein*	I	+	**11.03**			0.1	0.5 ± 0.1	0.1	2.7	2.9
AF1175	acdS	acyl-CoA dehydrogenase, short chain-specific	I	+	**11.06**			0.1	0.4	0.1	2.5	2.5
AF1177	hbd-6	3-hydroxyacyl-CoA dehydrogenase	I	+	**6.48**			0.2	0.2	0.2	1.2 ± 0.1	1.3 ± 0.1
AF1190	hbd-7	3-hydroxyacyl-CoA dehydrogenase	I	-	1.48			1.2 ± 0.1	1.6 ± 0.2	1.2 ± 0.1	2	2
AF1207	kduD	2-deoxy-D-gluconate 3-dehydrogenase	I	-	1.40			0.8 ± 0.1	0.7 ± 0.1	0.8 ± 0.1	1.4 ± 0.2	1.1 ± 0.1
AF1287	acs-6	acetyl-CoA synthetase	I	-	1.39	1.36		2.1	2 ± 0.1	2.3	**3**	**3**
AF1538		Short chain fatty acids transporter*	I	-	**8.01**			0.1	0.1	0.1	0.6 ± 0.1	0.7 ± 0.1
AF1641	fad-4	enoyl-CoA hydratase	I	-	**4.36**			0.1	0.1	0.1	0.4	0.4
AF1678		Putative sterol carrier protein*	I	-	**1.99**			0.3	0.4	0.3	0.5	0.7
AF1772	fadD-7	long-chain-fatty-acid--CoA ligase	I	+	**2.66**			0.8 ± 0.1	1.4 ± 0.1	0.9 ± 0.1	**3**	**3**
AF1855	entE	2,3-dihydrosybenzoate-AMP ligase	I	+	**17.87**			0	0	0	0.8 ± 0.1	0.9
AF1932	fadD-8	long-chain-fatty-acid--CoA ligase	I	+	**2.12**			0.4	0.5	0.3	0.7	1
AF2368	fadD-9	long-chain-fatty-acid--CoA ligase	I	-	1.43			0.8 ± 0.1	0.9 ± 0.1	0.6 ± 0.1	1.3 ± 0.1	1.1 ± 0.1
AF2415	acaA-2	3-hydroxy-3-methylglutaryl CoA synthase*	I	+	1.31			2 ± 0.2	1.8	1.8 ± 0.1	2.6 ± 0.2	2.7
AF2416	acaB-12	acetyl-CoA acetyltransferase	I	+	**1.58**			1.7 ± 0.1	1.5	1.4 ± 0.1	2.3 ± 0.2	2.8

Table S1b. Constitutively higly expressed genes corresponding to fatty acid metabolism and transport (COG: I)

Locus tag	Locus	NCBI annotation (*arCOG; Wolf et al., 2012)	COG	strand	H_2 vs. Lactate	Thiosulfate vs. Sulfate	late-log vs. mid-log	S-L	late-log S-L	T-L	T-H_2/CO_2	late-log T-H_2/CO_2
AF0283	acaB-7	acetyl-CoA acetyltransferase	I	+				**3.3**	**3.2**	**3.4**	**3.2**	**3**
AF0285	hbd-2	3-hydroxyacyl-CoA dehydrogenase	I	+				**3.1**	**3.2**	**3.2**	**3**	2.9 ± 0.3
AF0286	etfB	electron transfer flavoprotein, subunit beta	C	+				**3.5**	**3.5**	**3.5**	**3.5**	**3.5**
AF0287	etfA	electron transfer flavoprotein, subunit alpha	C	+				2.8	**3.2**	2.8	**3**	**3.1**
AF0967	acaB-9	acetyl-CoA acetyltransferase	I	-		1.21		**3**	2.6	**3.3**	**3.5**	**3.5**
AF1288a		methylmalonyl-CoA mutase N-terminal domain-containing protein	I	+				2.9	2.2	**3.1**	2.6	2.8
AF1288b		methylmalonyl-CoA mutase C-terminal domain-containing protein	I	+				2.9	1.8 ± 0.2	**3.2**	2.5 ± 0.3	2.6 ± 0.3
AF1291	acaB-11	3-ketoacyl-CoA thiolase	I	+				2.6	1.8	2.7	2.1	2.1
AF1794	ino1	myo-inositol-1-phosphate synthase	I	-				2.5	2.4	2.6	2.2	1.8 ± 0.1
AF2033	alkK-5	acyl-CoA synthetase	I	-				**3.1**	**3.1**	**3.3**	**3.1**	**3.2**
AF2057	acd-10	acyl-CoA dehydrogenase	I	+				2.5	2.5	2.3	2.2 ± 0.2	1.9 ± 0.1
AF2085		oxaloacetate decarboxylase, biotin carboxyl carrier subunit, putative	I	-				2.5	2.4	2.4	2.3	2.2
AF2215	mcmA1	methylmalonyl-CoA mutase, subunit alpha, N-terminus	I	-				2.7	2.4	2.7	2.8	2.8
AF2216	mmdC	methylmalonyl-CoA decarboxylase, biotin carboxyl carrier subunit	I	-			-1.26	2.7	1.9 ± 0.1	2.7	2.6 ± 0.2	2.2
AF2217	mmdA	methylmalonyl-CoA decarboxylase, subunit alpha	I	-				2.6	2.1	2.7	2.6	2.5

Table S1c. Homologues of 4-Hydroxybutyryl-CoA dehydratase

Locus tag	Locus	NCBI annotation (*arCOG; Wolf et al., 2012)	COG	strand	H_2 vs. Lactate	Thiosulfate vs. Sulfate	late-log vs. mid-log	S-L	late-log S-L	T-L	T-H_2/CO_2	late-log T-H_2/CO_2
AF0333	hpaA-1	4-hydroxyphenylacetate-3-hydroxylase	Q	+				0.9 ± 0.1	1.3 ± 0.1	0.7 ± 0.1	0.6 ± 0.1	0.5
AF0885	hpaA-2	4-hydroxyphenylacetate-3-hydroxylase	Q	-	**1.54**			0.8 ± 0.1	0.6	0.9 ± 0.1	1.3 ± 0.1	1.3
AF1027	hpaA-3	4-hydroxyphenylacetate-3-hydroxylase	Q	-				0.2	0.5	0.2	0.5 ± 0.1	0.5

Table S1d. Genes of the TCA cycle

Locus tag	Locus	NCBI annotation (*arCOG; Wolf et al., 2012)	COG	strand	H_2 vs. Lactate	Thiosulfate vs. Sulfate	late-log vs. mid-log	S-L	late-log S-L	T-L	T-H_2/CO_2	late-log T-H_2/CO_2
Acetyl-CoA + Oxaloacetate + H2O → Citrate + CoA												
AF1340	citZ	citrate synthase	C	-			-1.12	2.6	2.2	2.6	2.4	2.3
Citrate → Isocitrate												
AF1963	acn	aconitase	E	+				3	2.8	3	2.7	2.1
Isocitrate + NADP → 2-Oxoglutarate + NADPH + CO2												
AF0647	icd	isocitrate dehydrogenase, NADP	C	+				2.4	2.7	2.2	2.1	2
Pyruvate (or Pospoenolpyruvate) + CO_2(aq) ↔ Oxaloacetate												
AF0220	acc	pyruvate carboxylase subunit A	I	+	**-2.39**			1 ± 0.1	1	1.4 ± 0.1	0.5	0.4
AF1252m	oadA	oxaloacetate decarboxylase	C	-				1.8 ± 0.2	2	1.8 ± 0.1	1.5 ± 0.1	1.5 ± 0.1
AF1486		phosphoenolpyruvate carboxylase	G	-	-1.41			0.1	0.1	0.1	0.1	0.1
AF2085		oxaloacetate decarboxylase, biotin carboxyl carrier subunit, putative	I	-				2.5	2.4	2.4	2.3	2.2
Oxaloacetate + NADPH ↔ Malate + $NADP^+$												
AF0855	mdhA	L-malate dehydrogenase, NAD+-dependent	C	+				1.9 ± 0.1	2	2	1.9 ± 0.1	2
AF0628	leuB	3-isopropylmalate dehydrogenase	C	-			1.15	2.2	2.8	2.3	2.2	2.5
AF0629	leuD	3-isopropylmalate dehydratase small subunit	E	-				1.2 ± 0.1	1.6 ± 0.1	1.2 ± 0.1	1.2 ± 0.1	1.4 ± 0.1
AF1727	mae	malate oxidoreductase	C	+	**2.62**			0.7 ± 0.1	0.9 ± 0.1	0.3	1.6 ± 0.2	1.9 ± 0.1
Malate ↔ Fumarate + H_2O												
AF1098	fum-1	fumarate hydratase	C	-				0.4 ± 0.1	1.5 ± 0.1	0.3	1.4 ± 0.1	1.9 ± 0.1
AF1099	fum-2	fumarate hydratase	C	-				0.4 ± 0.1	1.3	0.4	1.5 ± 0.1	1.6 ± 0.1
Fumarate + [ΔH^+]↔ Succinate												
AF0681	sdhA	succinate dehydrogenase flavoprotein subunit	C	+				2.3 ± 0.2	1.6 ± 0.2	2.3 ± 0.2	3.1	3
AF0682	sdhB	succinate dehydrogenase iron-sulfur subunit	C	+	**2.03**			0.9 ± 0.1	0.8 ± 0.1	0.9 ± 0.1	1.7 ± 0.1	2.1
AF0683	sdhC	succinate dehydrogenase, subunit C	C	+	**1.88**			1.4 ± 0.1	1.2 ± 0.1	1.3 ± 0.2	2.3	2.8
AF0684	sdhD	succinate dehydrogenase, subunit D	C	+	**2.47**			0.9 ± 0.1	0.8 ± 0.1	0.8 ± 0.1	1.7 ± 0.1	2.6 ± 0.2
Succinate + CoA + (G/A)TP ↔ Succinyl CoA + Pi + (G/A)DP												
AF1539	sucD-1	succinyl-CoA synthetase, alpha subunit	C	-				0.4 ± 0.1	0.6	0.5 ± 0.1	0.5 ± 0.1	0.3
AF1540	sucC	succinyl-CoA synthetase, beta subunit	C	-				0.6 ± 0.1	1.1	0.9 ± 0.1	0.9 ± 0.1	0.7
AF2185	sucD-2	succinyl-CoA synthetase, alpha subunit	C	-				2 ± 0.1	2.7	2.2	2.9	2.4
AF2186	sucC	succinyl-CoA synthetase, beta subunit	C	-				1.4 ± 0.1	2.5	1.6 ± 0.1	2.2	2.1

Table S2. Genes of the acetyl-CoA pathway and Fqo complex

Locus tag	Locus	NCBI annotation (*arCOG; Wolf et al., 2012)	COG	strand	H_2 vs. Lactate	Thiosulfate vs. Sulfate	late-log vs. mid-log		S-L	late-log S-L	T-L	$T-H_2/CO_2$	late-log $T-H_2/CO_2$
ACS/CODH													
Acetyl-CoA + H^+ + 5,6,7,8-Tetrahydromethanopterin → 5-Methyl-5,6,7,8-tetrahydromethanopterin + CO + CoA													
AF0376	cdhE	acetyl-CoA decarbonylase/synthase complex subunit gamma	C	-			1.06		3.2	3.4	3.2	3.1	3.4
AF0377	cdhD	acetyl-CoA decarbonylase/synthase complex subunit delta	C	-					3.4	3.4	3.4	3.4	3.2
AF0379	cdhC	acetyl-CoA decarbonylase/synthase complex subunit beta	C	-					3.4	3.4	3.5	3.4	3.4
CO + Fd_{ox} + 2 H2O ↔ CO2 + Fd_{red}													
AF1100	cdhA-1	acetyl-CoA decarbonylase/synthase complex subunit alpha	C	+		**1.57**			2.3 ± 0.3	1.9 ± 0.1	3.4	3.5	3.5
AF1101	cdhB-1	acetyl-CoA decarbonylase/synthase complex subunit epsilon	C	+		**2.12**			1.5 ± 0.2	1.4 ± 0.1	3	3	3.4
AF2397	cdhA-2	acetyl-CoA decarbonylase/synthase complex subunit alpha	C	+					3.6	3.5	3.5	3.6	3.6
AF2398	cdhB-2	acetyl-CoA decarbonylase/synthase complex subunit epsilon	C	+					3.4	3.4	3.3	3.3	3.3
5-Methyl-5,6,7,8-tetrahydromethanopterin + Coenzyme F_{420} ↔ 5,10-Methylenetetrahydromethanopterin + Reduced coenzyme F_{420}													
AF1066	mer-1	methylenetetrahydromethanopterin reductase	C	+	-1.10				2.7	2.8	2.8	2.5	2.4
AF1196	mer-2	N5,N10-methylenetetrahydromethanopterin reductase	C	-					0.8 ± 0.1	1.9 ± 0.1	0.9 ± 0.1	2.1	1.8
5,10-Methylenetetrahydromethanopterin + Coenzyme F_{420} ↔ 5,10-Methenyltetrahydromethanopterin + Reduced coenzyme F_{420}													
AF0714	mtd	F420-dependent methylenetetrahydromethanopterin dehydrogenase	C	+					3.1	3.1	3	3	2.9 ± 0.2
5,10-Methenyltetrahydromethanopterin + H_2O ↔ 5-Formyl-5,6,7,8-tetrahydromethanopterin + H^+													
AF1935	mch	N(5),N(10)-methenyltetrahydromethanopterin cyclohydrolase	H	-					3.4	3.4	3.5	3.4	3.3
5,10-Methenyltetrahydromethanopterin + Methanofuran + H2O ↔ Formylmethanofuran +Tetrahydromethanopterin													
AF2073	ftr-1	tetrahydromethanopterin formyltransferase	C	-					2.4	2.7	2.5	2.4	2.3
AF2207	ftr-2	tetrahydromethanopterin formyltransferase	C	+					3.1	3.3	3.2	3	3
Formylmethanofuran + H_2O + Fd_{ox} ↔ CO2 + Methanofuran + Fd_{red}													
AF0177	fwdE	tungsten formylmethanofuran dehydrogenase, subunit E	C	-					0.2	0.1	0.3	0.2	0.1
AF0433		uncharacterized conserved protein*	S	-					0.9 ± 0.3	0.8 ± 0.1	1.5 ± 0.2	1.4 ± 0.3	1.3 ± 0.3
AF1644	fwdF	tungsten formylmethanofuran dehydrogenase, subunit F	C	+					2.1	1.7 ± 0.1	2	2	2 ± 0.1
AF1649	fwdG	tungsten formylmethanofuran dehydrogenase, subunit G	C	+					1.2 ± 0.2	1.4 ± 0.2	2.1	1.6 ± 0.1	2.1 ± 0.2
AF1650	fwdB-1	tungsten formylmethanofuran dehydrogenase, subunit B	C	+					1.6 ± 0.2	2 ± 0.2	2.5	2.1 ± 0.2	2.7
AF1928	fwdD-2	tungsten formylmethanofuran dehydrogenase, subunit D	C	+					3.4	3.4	3.5	3.3	3.3
AF1929	fwdB-2	tungsten formylmethanofuran dehydrogenase, subunit B	C	+					3.5	3.5	3.5	3.5	3.5
AF1930	fwdA	tungsten formylmethanofuran dehydrogenase, subunit A	C	+					3.5	3.5	3.5	3.5	3.5
AF1931	fwdC	tungsten formylmethanofuran dehydrogenase, subunit C	C	+					3.3	3.4	3.2	3.3	3.4
**Fqo complex**													
AF1823		F420H2:quinone oxidoreductase, 16.5 kDa subunit, putative	S	+					3.3	2.6	2.7	2.9 ± 0.3	2.7
AF1824		F420H2:quinone oxidoreductase, 11.2 kDa subunit, putative	C	+					3.6	3.4	3.5	3.5	3.4
AF1825	nuoM	F420H2:quinone oxidoreductase, 53.9 kDa subunit	C	+					3.6	3.4	3.5	3.5	3.5
AF1826	nuoL	F420H2:quinone oxidoreductase, 72.4 kDa subunit.	C	+					3.3	2.9	3	3.1	3
AF1827		F420H2:quinone oxidoreductase, 43.2 kDa subunit, putative	C	+					3.6	3.4	3.5	3.5	3.5
AF1828		NADH dehydrogenase subunit A	C	+					3.5	3.4	3.4	3.4	3.4
AF1829		F420H2:quinone oxidoreductase, 39.7 kDa subunit, putative	C	+					3.3	3	3.3	3.2	3.2
AF1830	nuoD	NADH dehydrogenase subunit D	C	+					3.4	3.2	3.3	3.3	3.2
AF1831		NADH dehydrogenase subunit H	C	+					3.6	3.4	3.5	3.5	3.3
AF1832a		NADH dehydrogenase subunit I	C	+					3.5	3.4	3.4	3.4	3.2
AF1833	FqoF[i]	F420H2:quinone oxidoreductase, 39 kDa subunit, putative	C	+					3.2	3	3	3	2.9
