# Supplementary material for: Identification of key components in the energy metabolism of the hyperthermophilic sulfate-reducing archaeon Archaeoglobus fulgidus by transcriptome analyses
Source: Front Microbiol. 2014 Mar 11;5:95. doi: 10.3389/fmicb.2014.00095 (PMC3949148; doi:10.3389/fmicb.2014.00095)
Supplement: Figure S1 — Distribution of array abundance and correlation with and central metabolic processes. (A) Distribution of intensity of quantile-normalized data; (A-I) distribution of values corresponding to minimum expression per gene and (A-II) maximum expression per gene. (B) Gene set enrichment analysis (GSEA) (Subramanian et al., 2005) on the distribution of selected genes on a list of genes sorted by minimum intensity values (i, corresponding to pie chart A-I), i.e., deviation from a random distribution, is displayed for identified peptides and genes corresponding to KEGG pathways. The maximum enrichment score (ES) corresponds to the largest deviance from random distribution, the region above the maximum enrichment score corresponds to the leading edge (LE), this region is colored corresponding to the intensity at point of ES. The numbers denote—[values below LE/above LE]. [file Presentation1.ZIP › 58387_Steen_Data_Sheet_2.PDF]

Table S3. Differential expression (fold change) and expression (relative to mean) for all assayed genes

Locus tag	Locus	NCBI annotation (*arCOG; Wolf et al., 2012)	COG	strand	Differential expression (ANOVA): H_2 vs. Lactate	Thiosulfate vs. Sulfate	late-log vs. mid-log	Expression level relative to mean: S-L	late-log S-L	T-L	$T-H_2/CO_2$	late-log $T-H_2/CO_2$
AF0001		hypothetical protein	S	-				0.1	0.1	0.1	0.1	0.3
AF0002		hypothetical protein	S	-				0	0	0	0	0
AF0003		uncharacterized protein with SCP/PR1 domains*	S	+	-1.49			0.2	0.2	0.2	0.1	0.1
AF0004		putative ATPase RIL	R	+		-1.36		1.6	1.9 ± 0.1	1.3 ± 0.1	1.3 ± 0.1	1.2 ± 0.1
AF0005		Predicted antitoxin, copG family*	V	+				0.4	0.3	0.3	0.3	0.3
AF0006	mtaC-1	corrinoid methyltransferase protein	R	+				1.3	1 ± 0.1	1.1 ± 0.1	1.5 ± 0.1	1 ± 0.1
AF0007		uroporphyrinogen-III decarboxylase*	H	+				1.1	0.7	0.7	1.3 ± 0.1	0.8
AF0008	oxlT-1	oxalate/formate antiporter	G	+				0.2	0.2	0.2	0.2	0.2
AF0009	mtr	tetrahydromethanopterin S-methyltransferase subunit H	H	+				0.8	0.7	0.7 ± 0.1	0.5	0.3
AF0010		uncharacterized metal-binding protein*	R	+	**-1.57**			0.8	0.7	0.6	0.5	0.3
AF0011	mtaC-2	corrinoid methyltransferase protein	R	+				0.6	0.5 ± 0.1	0.5	0.5	0.3
AF0012		uroporphyrinogen-III decarboxylase*	H	+				0.8 ± 0.1	0.9 ± 0.1	0.7 ± 0.1	0.7 ± 0.1	0.5
AF0013	exuT	hexuronate transporter	G	+				0.5 ± 0.1	0.6 ± 0.1	0.4 ± 0.1	0.4 ± 0.1	0.3
AF0014		uncharacterized conserved protein*	S	+				0.1	0	0.1	0	0
AF0015	putP-1	proline permease	E	+				0.1	0.1	0.2	0.1	0.1
AF0016		uncharacterized conserved protein*	S	+				0.1	0.1	0.1	0.1	0.1
AF0017	hbd-1	3-hydroxyacyl-CoA dehydrogenase	I	+				0.3	0.3	0.3	0.4	0.3
AF0018	acaB-1	3-ketoacyl-CoA thiolase	I	+				0.4	0.5	0.4	0.4	0.4
AF0019		Predicted nucleic-acid-binding protein containing a Zn-ribbon*	R	+				0.2	0.3	0.2	0.2	0.2
AF0020	caiB-1	L-carnitine dehydratase	C	+				0.1	0.2	0.1	0.1	0.1
AF0021		signal-transducing histidine kinase	T	+				0.5	0.1	0.3	0.2	0.2
AF0022		Molybdopterin converting factor, small subunit*	H	-				0.1	0.1	0.1	0.1	0.1
AF0023	aor-1	aldehyde ferredoxin oxidoreductase	C	-	1.46			0.5 ± 0.1	0.6	0.5	0.8 ± 0.1	0.9
AF0024		alcohol dehydrogenase, iron-containing	C	-	**2.89**			0.2	0.1	0.2	0.7 ± 0.1	0.5
AF0025	cynX	cyanate transport protein	G	-	**3.33**			0.1	0.1	0.1	0.4 ± 0.1	0.4
AF0026		Sugar-specific transcriptional regulator TrmB*	K	+				0.1	0.1	0.1	0.2	0.2
AF0027		GYD domain, alpha/beta barrel superfamily*	S	+			1.41	0.1	0.2	0.1	0.2	0.2
AF0028		Sep-tRNA:Cys-tRNA synthetase	E	-		**-1.50**		0.6	0.6 ± 0.1	0.4	0.4	0.3
AF0029		uncharacterized conserved protein*	S	-	**-1.64**			0.2	0.3	0.2	0.1	0.1
AF0030		hypothetical protein	X	+				0	0	0	0	0
AF0031		tRNA m(1)G methyltransferase*	J	+				0.5	0.9 ± 0.1	0.5	0.4	0.6 ± 0.1
AF0032		Predicted hydrolase (HD superfamily)*	R	-				0.4	0.6	0.5	0.3	0.3
AF0033	acaA-1	acyl carrier protein synthase	I	+	**2.87**			0.1	0.1	0.1	0.4 ± 0.1	0.4
AF0034	acaB-2	acetyl-CoA acetyltransferase	I	+				0.2	0.2	0.2	0.2	0.2
AF0035		mannosephosphate isomerase, putative	S	-				1 ± 0.1	1.2 ± 0.1	1.1 ± 0.1	1.1 ± 0.1	0.9
AF0036	pcm-1	L-isoaspartyl protein carboxyl methyltransferase	O	+				0	0	0	0	0
AF0037	cobS-1	cobalamin (5'-phosphate) synthase	H	+				0.1	0.1	0.1	0	0
AF0038		Glycosyltransferase*	M	+			1.28	1.1 ± 0.1	1.4 ± 0.1	1 ± 0.1	1	1.4
AF0039		dolichol-P-glucose synthetase, putative	M	+				2.1 ± 0.2	2 ± 0.1	2	1.8 ± 0.1	1.8 ± 0.1
AF0040		uncharacterized membrane protein, required for N-linked glycosylation*	R	+				0.7 ± 0.1	0.5	0.5	0.4	0.5
AF0041	rfbB-1	polysaccharide ABC transporter, ATP-binding protein	G	-				0.1	0.1	0.1	0	0.1
AF0042	rfbA-1	polysaccharide ABC transporter, permease protein	G	-				0.1	0.1	0.1	0.1	0.1
AF0043	wbaZ-1	first mannosyl transferase	M	+				0.1	0.1	0.1	0.1	0.1
AF0044m	gmd-1	GDP-D-mannose dehydratase	M	+				0.8 ± 0.1	0.8	0.7 ± 0.1	0.6 ± 0.1	0.6
AF0045	mtfA	mannosyltransferase A	M	+				0.1	0.1	0.1	0.1	0.1
AF0046		SAM-dependent methyltransferase*	Q	-				0.3 ± 0.1	0.3	0.3	0.4 ± 0.1	0.3
AF0047		hypothetical protein	X	+				0	0	0	0	0
AF0048		Peptidase family C25*	E	-				0.1	0.2	0.1	0.4 ± 0.2	0.5 ± 0.1
AF0049		uncharacterized conserved protein*	S	-	**3.91**			0.1	0.3	0.1	0.6 ± 0.1	0.7
AF0050		S-adenosylmethionine synthetase	E	+				1.7 ± 0.1	1.8 ± 0.1	1.8 ± 0.1	1.5 ± 0.1	1 ± 0.1
AF0051	dapE-1	succinyl-diaminopimelate desuccinylase	E	-				0.5	0.6	0.5	0.4	0.3
AF0052		hypothetical protein	X	+	**2.73**			0	0.1	0	0.1	0.2
AF0053		Predicted membrane-associated Zn-dependent protease*	M	-				0.7 ± 0.1	0.6 ± 0.1	0.6	0.6 ± 0.1	0.7
AF0054		hypothetical protein	X	-				1 ± 0.1	1.1 ± 0.1	1 ± 0.1	1 ± 0.1	1.2 ± 0.1
AF0055		Swiveling domain associated with predicted aconitase*	C	-				1.3 ± 0.2	1.4 ± 0.1	1.3 ± 0.1	1.2 ± 0.1	1.5 ± 0.1
AF0056		DNA-directed RNA polymerase, subunit RPC12/RpoP (contains C4-type Zn-finger)*	K	-				1.4 ± 0.2	1.3 ± 0.2	1.4 ± 0.1	1.2 ± 0.2	1.1 ± 0.1
AF0057	rpl37ae	50S ribosomal protein L37Ae	J	-				1.2 ± 0.2	1.2 ± 0.1	1.1 ± 0.1	1.3 ± 0.1	1.4 ± 0.1
AF0058		Protein involved in ribosomal biogenesis, contains PuA domain*	J	-	**2.79**			0.3 ± 0.1	0.7	0.5	1.3 ± 0.1	1.6 ± 0.1
AF0059		uncharacterized conserved protein*	S	-	**2.57**			0.3	0.9	0.5	1.3 ± 0.1	1.7 ± 0.1
AF0060		hypothetical protein	X	+				0.1	0.1	0.1	0.1	0.1
AF0061	tmk	thymidylate kinase	F	+				0.1	0.1	0.1	0.1	0.1
AF0062		CBS domain*	R	-	1.38			0.4	0.3	0.4	0.5	0.5
AF0063		PIN domain*	V	-				0.3 ± 0.1	0.1	0.2	0.1	0.1
AF0064		hypothetical protein	X	+				0.5 ± 0.1	0.3 ± 0.1	0.4 ± 0.1	0.2 ± 0.1	0.3 ± 0.1
AF0065		PIN domain containing protein*	V	-				0.4 ± 0.1	0.4 ± 0.1	0.4 ± 0.1	0.2	0.4 ± 0.1
AF0066		hypothetical protein	X	-				0.7 ± 0.2	0.7 ± 0.1	0.5 ± 0.1	0.3	0.6 ± 0.1
AF0067		CRISPR system related protein Cas5, RAMP superfamily*	V	-				1.1 ± 0.1	1 ± 0.1	0.7 ± 0.1	0.5 ± 0.1	0.7 ± 0.2
AF0068		CRISPR system related protein Cas7, RAMP superfamily*	V	-		-1.40		2.3	2.1	1.7 ± 0.3	1.4 ± 0.1	1.5 ± 0.3
AF0069		uncharacterized conserved protein*	S	-				2.1	2.4	1.6 ± 0.3	1.2 ± 0.1	1.8 ± 0.2
AF0070		uncharacterized conserved protein*	S	-				0.7 ± 0.1	0.8 ± 0.1	0.5 ± 0.2	0.5 ± 0.1	0.9 ± 0.2
AF0071		ATP-dependent RNA helicase, putative	V	-				1.1 ± 0.1	0.8 ± 0.1	0.8 ± 0.2	0.8 ± 0.1	1 ± 0.1
AF0072		CRISPR system related protein, RAMP superfamily*	V	-				2	1.5 ± 0.1	1.5 ± 0.1	1.6 ± 0.2	1.6
AF0073		hypothetical protein	X	-				0	0	0	0	0
AF0074	birA	biotin operon repressor/biotin--	H	+				0.2	0.3	0.2	0.2	0.2
AF0075		Single-stranded DNA-specific exonuclease RecJ*	L	+				2.3 ± 0.2	2.6	2.4	2.3	2.5
AF0076		iron-sulfur cluster binding protein	C	+				2.2	2.5	2.3	2.3	2.4
AF0077	aor-2	aldehyde ferredoxin oxidoreductase	C	+			1.20	1.6 ± 0.1	2.1	1.9 ± 0.1	1.7 ± 0.1	2
AF0078		hypothetical protein	X	-				0.2	0.4	0.3	0.2	0.2
AF0079		uncharacterized conserved protein*	S	+	**3.15**			0.1	0.1	0.1	0.3	0.2
AF0080	argD-1	acetylornithine aminotransferase	E	+				0.8 ± 0.1	1.2	0.7	0.6	0.7
AF0081		Predicted membrane protein, DuF2068*	S	-				0.2	0.1	0.2	0.1	0.1
AF0082		Integrase*	L	+				0	0	0	0	0
AF0083		hypothetical protein	X	-				0.2	0.2	0.2	0.2	0.1
AF0084		uncharacterized conserved protein*	S	-				0.2	0.2	0.2	0.2	0.1
AF0085		hypothetical protein	X	+				0.2	0.1	0.2	0.1	0.1
AF0086	nrtB-1	nitrate ABC transporter, permease protein	P	-				1.1 ± 0.1	0.9	1.1 ± 0.1	0.9 ± 0.1	0.7
AF0087	nrtC-1	nitrate ABC transporter, ATP-binding protein	P	-				2.3	2.3	2.4	2.2	2
AF0088		thiamine biosynthesis protein, putative	P	-				1.9 ± 0.1	1.9	1.9 ± 0.1	1.9 ± 0.1	1.5 ± 0.1
AF0089	fadD-1	long-chain-fatty-acid--CoA ligase	I	+	**4.71**			0.1	0.8 ± 0.1	0.1	1.5 ± 0.1	1.8
AF0090		dehydrase	R	+				1.2 ± 0.1	1.3	1 ± 0.1	1.4 ± 0.1	1.3 ± 0.1
AF0091	hpcE-1	2-hydroxyhepta-2,4-diene-1,7-dioate isomerase	Q	+	-1.34			0.8	1	0.8 ± 0.1	0.6	0.7
AF0092	cysA	sulfate ABC transporter, ATP-binding protein	G	-	-1.21			2.1	2.2	2.4 ± 0.2	1.8 ± 0.2	1.7 ± 0.1
AF0093	cysT	sulfate ABC transporter, permease protein	O	-				2.8	**3.1**	2.8	2.5	**3**
AF0094		ABC-type molybdate transport system, periplasmic component*	P	-				**3**	2.8	2.9	2.7	2.3
AF0096		uncharacterized conserved protein*	S	-				0.1	0.1	0	0.1	0
AF0097		hypothetical protein	X	-			-1.13	0	0	0	0	0
AF0098		hypothetical protein	X	-			-1.22	0	0	0	0	0
AF0099		PIN domain containing protein*	V	-			**1.52**	0	0.1	0	0	0.1

AF0100		Predicted antitoxins containing the HTH domain*	V	-				0.1	0.1	0.1	0.1	0.1
AF0101		hypothetical protein	X	-		**-2.61**		0.3	0.2	0.1	0.1	0.1
AF0102		Predicted transcriptional regulator*	K	-		**-2.31**		0.8 ± 0.1	0.6	0.3	0.3	0.3 ± 0.1
AF0103		uncharacterized conserved protein*	S	+				1.4 ± 0.1	1.1	1.4 ± 0.1	1 ± 0.1	0.6
AF0104		Predicted DNA-binding protein with PD1-like DNA-binding motif*	R	-				0.9 ± 0.1	0.8	1.3 ± 0.1	1	1
AF0105		Predicted membrane protein*	S	-	**1.80**	**2.03**		0.3	0.2	0.5	0.6	0.6
AF0106	pyrB	aspartate carbamoyltransferase, catalytic subunit	F	+				2.6 ± 0.2	2.3	2.6	2.6	2 ± 0.1
AF0107	pyrI	aspartate carbamoyltransferase regulatory subunit	F	+				1.6 ± 0.2	1.8 ± 0.1	2	1.7 ± 0.1	1.5 ± 0.1
AF0108		fructose-bisphosphate aldolase	E	-				2.5	2.9	2.2	2.5	2.8
AF0109		RNase P subunit RPR2*	J	-				0.5	0.4	0.5	0.4	0.3
AF0110		O-phosphoseryl-tRNA synthetase	J	+				1.9	1.9	1.6 ± 0.1	1.7 ± 0.1	1.3 ± 0.1
AF0111		Predicted transcriptional regulator, contains C-terminal CBS domains*	K	-				1.5 ± 0.2	2.8	1.3 ± 0.1	2.4 ± 0.2	2.5
AF0112		Sir2 family transcriptional regulator	K	-				0.2	0.4	0.2	0.3	0.3
AF0113	purP	5-formaminoimidazole-4-carboxamide-1-(beta)-D-ribofuranosyl 5'-monophosphate synthetase	F	-	**-1.97**	**-1.71**		1.1 ± 0.1	1.1 ± 0.1	0.9	0.6	0.4
AF0114		transcriptional regulatory protein, putative	K	+			1.27	0.7	0.9	0.7 ± 0.1	0.7	0.9 ± 0.1
AF0115		Predicted amidohydrolase*	R	+			1.16	1.1	1.3 ± 0.1	1.1	1.1 ± 0.1	1.3 ± 0.1
AF0116	hemD	uroporphyrinogen III synthase	H	+				0.8 ± 0.1	0.8 ± 0.1	0.8 ± 0.1	0.7 ± 0.1	0.8
AF0117	act-1	pyruvate formate-lyase activating enzyme	O	-			**-1.64**	0.6 ± 0.1	0.4	0.6	0.5 ± 0.1	0.3
AF0118		hypothetical protein	X	-			**-1.80**	1 ± 0.2	0.5 ± 0.1	0.8	0.8 ± 0.1	0.4
AF0119		uncharacterized conserved protein*	S	+				1.4 ± 0.2	1.9 ± 0.1	1.5 ± 0.2	1.3 ± 0.1	1.2 ± 0.1
AF0121m		uncharacterized conserved protein*	S	+	**4.01**			0.3	1 ± 0.1	0.3	2.1	2.3
AF0123		hypothetical protein	X	-	**3.45**			0.4 ± 0.1	1 ± 0.1	0.4	2.2 ± 0.2	2.1 ± 0.2
AF0124		Predicted permease*	R	-	**3.43**			0.1	0.6 ± 0.1	0.1	1.1 ± 0.1	1.1 ± 0.1
AF0125		Nucleotide-binding protein, uspA family*	T	+				0.7 ± 0.1	0.7	0.8 ± 0.1	0.5 ± 0.1	0.7 ± 0.1
AF0126		hypothetical protein	X	-				1.5 ± 0.1	1.3 ± 0.1	1.6 ± 0.2	1.1 ± 0.1	1.4 ± 0.1
AF0127		Predicted permease*	R	-				1.2 ± 0.1	1.2 ± 0.1	1.1 ± 0.1	0.9	1 ± 0.1
AF0128		Predicted antitoxin, copG family*	V	+			1.48	0.6 ± 0.1	1.2 ± 0.1	0.6 ± 0.1	0.8 ± 0.1	0.9
AF0129		Deoxyinosine 3'endonuclease (endonuclease V)*	L	-	**1.91**			0.5	0.6	0.5 ± 0.1	1.1 ± 0.1	1 ± 0.1
AF0130	aphA	acetylpolyamine aminohydrolase	R	-	**1.74**			1 ± 0.1	1.6	1 ± 0.1	2.1	2.2
AF0131		NAD(P)H-flavin oxidoreductase, putative	C	+	**3.51**			0.1	0.3	0.1	0.6 ± 0.1	0.8 ± 0.1
AF0132		Predicted nucleic-acid-binding protein containing a Zn-ribbon*	R	-				0.2	0.2	0.2	0.3	0.2
AF0133	acaB-3	acetyl-CoA acetyltransferase	I	-				0.3	0.3	0.3	0.3	0.3
AF0134	acaB-4	3-ketoacyl-CoA thiolase	I	-	1.31			0.2	0.2	0.2	0.2	0.3
AF0135		OB-fold domain and Zn-ribbon containing protein, possible acyl-CoA-binding protein*	R	-	**1.68**			0	0	0	0.1	0.1
AF0136		uncharacterized conserved protein*	S	-	**3.08**			0	0.1	0	0.1	0.2
AF0137		transposase, putative	L	+	-1.11	-1.11		0	0	0	0	0
AF0138		transposase IS240-A	L	+	-1.11			0	0	0	0	0
AF0139		Predicted transcriptional regulator*	K	-				0.2	0.2	0.1	0.1	0.1
AF0140	ubiE	ubiquinone/menaquinone biosynthesis methyltransferase	Q	+				0	0	0	0	0
AF0141		hypothetical protein	X	+				0.1	0.1	0.1	0.1	0.1
AF0142		cytochrome C oxidase, subunit II, putative	C	+				0.2	0.1	0.2	0.1	0.1
AF0143		Polyferredoxin*	C	+				0.1	0.1	0.1	0.1	0.1
AF0144	cbaB	cytochrome C oxidase, subunit II	C	+			-1.24	0.1	0	0.1	0	0
AF0145		hypothetical protein	X	-			-1.42	0.1	0	0.1	0.1	0
AF0146		hypothetical protein	X	-				0	0	0	0	0
AF0147		uncharacterized conserved protein*	S	-				0.1	0.1	0.1	0.1	0.1
AF0148		hypothetical protein	X	+		**-2.38**		0.2	0.1	0.1	0	0.1
AF0149		hypothetical protein	X	+				0.1	0	0	0	0
AF0150		hypothetical protein	X	-				0.1	0.1	0.1	0.1	0.1
AF0151		hypothetical protein	X	-	**1.67**			0.1	0.1	0.1	0.2	0.2
AF0152	copB	copper-transporting ATPase, P-type	P	+	**1.56**			0.1	0.1	0.2	0.2	0.3
AF0153		Predicted membrane protein*	S	-		**2.55**		0.1	0.1	0.3	0.3 ± 0.1	0.4 ± 0.1
AF0154		High-affinity Fe2+/Pb2+ permease*	P	+				0.8 ± 0.1	0.8 ± 0.1	1.3 ± 0.2	0.7 ± 0.1	0.7 ± 0.1
AF0155		hypothetical protein	X	+				0.6 ± 0.1	0.6 ± 0.1	0.9 ± 0.1	0.4 ± 0.1	0.4 ± 0.1
AF0156	fdx-1	ferredoxin	C	+				0.4	0.3	0.6 ± 0.1	0.2	0.2
AF0157		molybdopterin oxidoreductase, iron-sulfur binding subunit	C	+				0.3	0.2	0.5 ± 0.1	0.3	0.3
AF0158		Predicted membrane protein*	S	+				0.5 ± 0.1	0.4	1 ± 0.1	0.8 ± 0.2	0.7 ± 0.1
AF0159		molybdopterin oxidoreductase, molybdopterin binding subunit, putative	C	+				0.9 ± 0.1	0.7	1.2 ± 0.1	1.2 ± 0.1	1.1 ± 0.1
AF0160		uncharacterized conserved protein*	S	+				0.2	0.2	0.2	0.2	0.2
AF0161	moeA-3	molybdenum cofactor biosynthesis protein	H	+				0.4	0.3	0.5	0.4	0.4
AF0162		hypothetical protein	X	+				0.3	0.2	0.3	0.1	0.1
AF0163		uncharacterized conserved protein*	S	+				0.2	0.1	0.1	0.1	0.1
AF0164	nirA	ferredoxin-nitrite reductase	P	+				0.1	0	0.1	0	0
AF0165		Predicted redox protein, regulator of disulfide bond formation*	O	+				0.1	0.1	0.1	0.1	0.1
AF0166	fdx-2	ferredoxin	C	-				0.1	0.1	0.2	0.1	0.1
AF0167	fprA-1	flavoprotein	C	-				1.2 ± 0.1	1.7 ± 0.1	1.4	1.8 ± 0.1	2
AF0168		arsenical resistance operon repressor, putative	K	-	1.44			0.5 ± 0.1	0.7	0.7	1 ± 0.1	0.9 ± 0.1
AF0169		uncharacterized conserved protein*	S	-				0.2	0.3	0.2	0.3	0.4
AF0170		Hemerythrin HHE cation binding domain containing protein*	S	+	**1.98**			0.5	0.7 ± 0.1	0.5	1.1	1.2
AF0171		Sulfite oxidase or related enzyme*	R	+	**2.21**			0.1	0.2	0.2	0.4 ± 0.1	0.5
AF0172		hypothetical protein	X	+	**1.75**			0.1	0.1	0.2	0.2	0.3
AF0173		reductase, assembly protein	C	-			-1.43	0.2	0.1	0.2	0.2	0.1
AF0174		molybdopterin oxidoreductase, membrane subunit	C	-			-1.23	1.3 ± 0.1	1.1 ± 0.1	1.3 ± 0.1	1.4 ± 0.1	1
AF0175		molybdopterin oxidoreductase, iron-sulfur binding subunit	C	-			-1.19	1.8 ± 0.2	1.6 ± 0.1	2	2 ± 0.1	1.6 ± 0.1
AF0176		molybdopterin oxidoreductase, molybdopterin binding subunit	C	-			-1.20	1.9 ± 0.2	1.6 ± 0.1	2 ± 0.1	1.9 ± 0.1	1.6 ± 0.1
AF0177	fwdE	tungsten formylmethanofuran dehydrogenase, subunit E	C	-				0.2	0.1	0.3	0.2	0.1
AF0178		uncharacterized conserved protein*	S	+				0.1	0.2	0.1	0.1	0.1
AF0179		hypothetical protein	X	+				0.2	0.3	0.2	0.2	0.1
AF0180		3-hydroxy-3-methylglutaryl CoA synthase family enzyme*	R	-				0.2	0.1	0.2	0.1	0.1
AF0181		Sep-tRNA:Cys-tRNA synthetase	E	-				0.4	0.2	0.4 ± 0.1	0.2	0.2
AF0182		hypothetical protein	X	-				0.8 ± 0.1	0.5 ± 0.1	1 ± 0.2	0.5 ± 0.1	0.5
AF0183		Peroxiredoxin family protein*	R	-				0.8 ± 0.1	0.5	1 ± 0.1	0.6 ± 0.1	0.5
AF0184		Predicted transcriptional regulator*	R	-				2	1.8 ± 0.1	2.1	1.8 ± 0.1	1.8 ± 0.1
AF0185	nifU-1	nifu protein	C	-				1.4 ± 0.2	1.2	1.6 ± 0.1	1.6 ± 0.1	1.6 ± 0.1
AF0186	nifS-1	nifS protein, class-V aminotransferase	E	-				1 ± 0.2	0.6	1.4 ± 0.1	1.3 ± 0.1	1.1 ± 0.1
AF0187		Predicted peroxiredoxin*	R	-				0.3 ± 0.1	0.3	0.6	0.4	0.3
AF0188		Predicted redox protein, regulator of disulfide bond formation*	O	-				0	0	0.1	0	0
AF0189		hypothetical protein	X	-				0.1	0.2	0.2	0.2	0.4
AF0190		cytochrome C oxidase, subunit II, putative	C	-	**1.54**			0.1	0.1	0.1	0.2	0.2
AF0191		Cation transport ATPase*	P	-		**-1.77**		0.1	0.1	0.1	0.1	0.1
AF0192		Cation transport ATPase*	P	-		**-2.35**		0.2	0.2	0.1	0.1	0.1
AF0194		hypothetical protein	X	+				0.1	0.1	0.1	0.1	0.1
AF0195		Ferritin-like domain containing protein*	S	+				0	0	0	0	0
AF0196	alkK-1	medium-chain acyl-CoA ligase	I	+	1.43			0.1	0.1	0.1	0.1	0.1
AF0197	acs-1	acetoacetyl-CoA synthetase	I	+				0.2	0.2	0.2	0.2	0.2
AF0198		monoamine oxidase regulatory protein, putative	I	+				0.6 ± 0.1	0.5	0.5	0.6 ± 0.1	0.4
AF0199	acd-1	acyl-CoA dehydrogenase	I	+			-1.45	0.4	0.3	0.4	0.4	0.2
AF0200	fadD-2	long-chain-fatty-acid--CoA ligase	I	+				0.2	0.2	0.2	0.2	0.2
AF0201	acaB-5	3-ketoacyl-CoA thiolase	I	+		-1.26		0.5	0.4	0.4	0.3	0.3
AF0202	acaB-6	3-ketoacyl-CoA thiolase	I	+				0.1	0.1	0.1	0.1	0.1
AF0203		Predicted nucleic-acid-binding protein containing a Zn-ribbon*	R	+	-1.32			0.1	0.1	0.1	0.1	0.1
AF0204		uncharacterized conserved protein*	S	+				0.7 ± 0.1	0.5	0.8	0.8	0.9
AF0205		Predicted RNA methylase*	J	+				0.8 ± 0.1	0.7	0.9	1 ± 0.1	1.3 ± 0.1

AF0206		exosome complex RNA-binding protein Csl4	J	+	1.19				1.3 ± 0.1	1.4	1.4 ± 0.1	1.6 ± 0.1	1.7 ± 0.1
AF0207	rpoL	DNA-directed RNA polymerase, subunit L	K	+			**-1.77**		0.9 ± 0.1	0.5	1 ± 0.1	1.1 ± 0.1	0.6 ± 0.1
AF0208		signal-transducing histidine kinase	T	-					0.4 ± 0.1	0.5	0.5	0.3	0.5 ± 0.1
AF0209		3-polyprenyl-4-hydroxybenzoate decarboxylase or related decarboxylase*	H	+					2.3	1.8	1.9	2.1	1.8 ± 0.1
AF0210		Predicted membrane protein*	S	+					1.4 ± 0.1	1.1 ± 0.1	1.1 ± 0.1	1.1	1 ± 0.1
AF0211		Protein containing two CBS domains (some fused to C-terminal double-stranded RNA-binding domain of RaiA family)*	R	+					1.6 ± 0.1	1.9 ± 0.1	1.4 ± 0.1	1.3	1.2 ± 0.1
AF0212	hisD	histidinol dehydrogenase	E	-					0.4	0.7	0.4	0.3	0.3
AF0213		Sugar-specific transcriptional regulator TrmB*	K	-					0.3	0.2	0.3	0.2	0.2
AF0214		S-layer domain*	M	+					1.4 ± 0.2	1.1 ± 0.1	1.4 ± 0.1	1.3 ± 0.2	1.2 ± 0.1
AF0215	nac	nascent polypeptide-associated complex protein	K	+					1.1 ± 0.1	1.1 ± 0.1	1.2	1	1
AF0216		L-isoaspartyl protein carboxyl methyltransferase PimT, putative	J	+					1 ± 0.1	0.9 ± 0.1	1.2 ± 0.1	0.9 ± 0.1	0.9
AF0217	napA-1	Na+/H+ antiporter	P	-					0.2	0.2	0.3	0.2	0.2
AF0218	trkA-1	TRK potassium uptake system protein	P	-					0.8 ± 0.1	0.8 ± 0.1	0.8 ± 0.1	0.7 ± 0.1	0.7 ± 0.1
AF0219	leuA-2	2-isopropylmalate synthase	E	-					2	1.9 ± 0.1	1.9 ± 0.1	1.9 ± 0.1	1.6 ± 0.1
AF0220	acc	pyruvate carboxylase subunit A	I	+	**-2.39**				1 ± 0.1	1	1.4 ± 0.1	0.5	0.4
AF0221	braG-1	branched-chain amino acid ABC transporter, ATP-binding protein	E	-					1.8 ± 0.1	1.4	1.8 ± 0.1	2 ± 0.1	1.5 ± 0.1
AF0222	braF-1	branched-chain amino acid ABC transporter, ATP-binding protein	E	-					1.6	1.5	1.4	1.6 ± 0.1	1.3
AF0223	braC-1	branched-chain amino acid ABC transporter, periplasmic binding protein	E	+					**3.4**	**3.3**	**3.3**	**3.4**	**3.3**
AF0224	braD-1	branched-chain amino acid ABC transporter, permease protein	E	+					**3.3**	**3.3**	**3.2**	**3.3**	**3.4**
AF0225	braE-1	branched-chain amino acid ABC transporter, permease protein	E	+					2.8 ± 0.2	2.5	2.6 ± 0.2	2.8	**3 ± 0.2**
AF0226	noxC	NADH oxidase	C	+					1.6 ± 0.1	1.4 ± 0.1	1.5 ± 0.1	1.7 ± 0.1	1.9 ± 0.3
AF0227	pheA	chorismate mutase/prephenate dehydratase	E	-					2.2 ± 0.2	1.9 ± 0.1	2.1 ± 0.2	2.3 ± 0.2	2.4 ± 0.2
AF0228	aroD	3-dehydroquinate dehydratase	E	-					2.5 ± 0.2	1.8 ± 0.1	2.5 ± 0.2	2.7 ± 0.3	2.8
AF0229		3-dehydroquinate synthase	E	-					2.5	2.2	2.5	2.8 ± 0.2	2.9
AF0230		fructose-bisphosphate aldolase	E	-					2.3 ± 0.2	2 ± 0.1	2.1	2.7 ± 0.3	**3**
AF0231	glnH	glutamine ABC transporter, periplasmic glutamine-binding protein	E	+					2.3 ± 0.2	2.2	2.3 ± 0.2	2.2	1.9 ± 0.1
AF0232	glnP	glutamine ABC transporter, permease protein	E	+					0.5	0.5	0.4	0.4	0.3
AF0233		hypothetical protein	X	+					0.1	0	0.1	0.1	0
AF0234		Metal-dependent hydrolase of the beta-lactamase superfamily II*	R	-			**-1.84**		0.3 ± 0.1	0.2	0.4 ± 0.1	0.3	0.2
AF0235	htpX	heat shock protein	O	-					2.1	1.8 ± 0.1	2.1 ± 0.2	2.2 ± 0.2	1.4 ± 0.1
AF0236		hypothetical protein	X	+			**-1.94**		0.2	0.1	0.3	0.2	0.1
AF0237	panF-3	pantothenate permease	E	+					0.5 ± 0.1	0.3	0.6	0.3	0.2
AF0238	cbf5	H/ACA RNA-protein complex component Cbf5p	J	-					0.8	0.9	0.8 ± 0.1	0.7 ± 0.1	0.6
AF0239	gptA-1	xanthine-guanine phosphoribosyltransferase	R	+					1.5 ± 0.1	1.5 ± 0.1	1.4 ± 0.1	1.4 ± 0.2	1.2 ± 0.1
AF0240	adeC	adenine deaminase	F	+					2 ± 0.2	2.2	1.9 ± 0.1	1.9 ± 0.2	2 ± 0.1
AF0241		uncharacterized conserved protein*	S	-			-1.46		0.2	0.1	0.2	0.2	0.1
AF0242	graD-1	glucose-1-phosphate thymidylyltransferase	M	+					0.8 ± 0.1	0.8	0.9	0.8 ± 0.1	0.6
AF0243		Radical SAM superfamily enzyme*	C	+					0.7 ± 0.1	0.7 ± 0.1	0.8 ± 0.1	0.7 ± 0.1	0.6 ± 0.1
AF0244		cell division control protein 6, putative	L	+					1.4 ± 0.2	1.2	1.7 ± 0.1	1.2 ± 0.1	1 ± 0.1
AF0245	desR	iron-dependent repressor	K	-					1.1 ± 0.2	0.8 ± 0.2	1.5 ± 0.1	1.2 ± 0.2	1
AF0246	feoB-1	iron transporter	P	-					2.4	2 ± 0.3	2.7	2.6 ± 0.2	2.8
AF0247		uncharacterized protein of the AP superfamily*	R	+					0.3	0.4	0.3	0.4	0.4
AF0248		NADH-dependent flavin oxidoreductase	C	-					0.4 ± 0.1	0.7	0.3	0.3	0.3
AF0249		hypothetical protein	X	+					0.4	0.6	0.4	0.5	0.9
AF0250		hypothetical protein	X	+					0.3 ± 0.1	0.3	0.3	0.3	0.3
AF0251		Ca2+/Na+ antiporter*	P	-	**-1.83**				0.3	0.2	0.3	0.1	0.1
AF0252	pyrG	CTP synthetase	F	+					2.7	2.9	2.7	2.8	2.5
AF0253	guaA-1	GMP synthase subunit B	F	+					2.7	2.9	2.6	2.8	2.6
AF0254	noxA-1	NADH oxidase	P	-					1	0.8	0.9 ± 0.1	1 ± 0.1	1
AF0255		Transcriptional regulator, ArsR family*	K	+					0.3 ± 0.1	0.4	0.5 ± 0.1	0.3	0.6 ± 0.1
AF0256	purP	5-formaminoimidazole-4-carboxamide-1-(beta)-D-ribofuranosyl 5'-monophosphate synthetase-like protein	F	-					1.3	1.3	1.1 ± 0.1	1 ± 0.1	0.7
AF0257		hypothetical protein	X	-					0	0	0.1	0.1	0
AF0258		hypothetical protein	X	-					0	0	0.1	0.1	0
AF0259		Minimal nucleotidyltransferase*	V	+					0.4	0.2	0.3	0.2	0.2
AF0260	gltX	glutamyl-tRNA synthetase	J	-	-1.14				2.5	2.4	2.3	2.1	2
AF0261		uncharacterized conserved protein*	S	+					0.3	0.2	0.2	0.2	0.2
AF0262	alkK-2	medium-chain acyl-CoA ligase	I	-	**6.78**				0.1	0.2	0.1	0.7 ± 0.1	0.9 ± 0.1
AF0263		Predicted sugar nucleotidyltransferase*	M	-					0.3 ± 0.1	0.3	0.3 ± 0.1	0.2	0.2
AF0264	rad2	flap endonuclease-1	L	+					0.6 ± 0.1	0.8 ± 0.1	0.6 ± 0.1	0.8 ± 0.1	0.8 ± 0.1
AF0265	moaB	molybdenum cofactor biosynthesis protein	H	+					0.5 ± 0.1	0.6 ± 0.1	0.5	0.6 ± 0.1	0.6 ± 0.1
AF0266		Permease of the drug/metabolite transporter (DMT) superfamily*	G	-					0.3	0.2	0.3	0.2	0.1
AF0267		Acyl-coenzyme A:6-aminopenicillanic acid acyl-transferase related enzyme*	R	-					0.1	0.1	0.1	0.1	0.1
AF0268		hypothetical protein	X	-					0	0	0	0	0
AF0269		hypothetical protein	X	-					0	0	0	0	0
AF0270		alkyl hydroperoxide reductase	O	-					0	0	0	0	0
AF0271		heterodisulfide reductase, subunit B, putative	C	-					0	0	0	0	0
AF0272		uncharacterized conserved protein*	S	-			**1.92**		0.3 ± 0.1	0.7	0.3	0.2	0.5
AF0273	soxA	sarcosine oxidase, subunit alpha	R	+					**3.5**	**3.5**	**3.4**	**3.5**	**3.3 ± 0.2**
AF0274	soxB	sarcosine oxidase, subunit beta	E	+			1.09		**3**	**3.3**	2.9	2.8	**3.1**
AF0275	slgB-1	surface layer protein B	S	+		**1.75**			1.3 ± 0.1	1.1 ± 0.1	2.2 ± 0.2	2.1	2.4
AF0276		NaMN:DMB phosphoribosyltransferase*	H	+					1.8 ± 0.1	2	2.3	2.1	2.2
AF0277		signal-transducing histidine kinase, putative	T	+	**1.93**				0.4 ± 0.1	0.5	0.5	0.8 ± 0.1	1 ± 0.1
AF0278		ISA1214-1 transposase	S	-					0.1	0.1	0.1	0.1	0.1
AF0279		ISA1214-1 transposase	L	+					0.1	0.2	0.1	0.1	0.1
AF0280		Predicted RNA-binding protein, contains TRAM domain*	R	+					0.5 ± 0.2	0.6	0.3 ± 0.1	0.4 ± 0.1	1 ± 0.2
AF0281		Predicted transcriptional regulator*	K	-					0.6 ± 0.1	0.6	0.7 ± 0.1	0.8 ± 0.1	0.9 ± 0.1
AF0282		uncharacterized conserved protein*	S	-					0.6	0.5	0.5	0.5 ± 0.1	0.3
AF0283	acaB-7	acetyl-CoA acetyltransferase	I	+					**3.3**	**3.2**	**3.4**	**3.2**	**3**
AF0284		Predicted nucleic-acid-binding protein containing a Zn-ribbon*	R	+					**3.2**	**3.1**	**3.2**	**3.1**	2.9
AF0285	hbd-2	3-hydroxyacyl-CoA dehydrogenase	I	+					**3.1**	**3.2**	**3.2**	**3**	2.9 ± 0.3
AF0286	etfB	electron transfer flavoprotein, subunit beta	C	+					**3.5**	**3.5**	**3.5**	**3.5**	**3.5**
AF0287	etfA	electron transfer flavoprotein, subunit alpha	C	+					2.8	**3.2**	2.8	**3**	**3.1**
AF0288	cysC	adenylylsulfate kinase	P	+					0.5	0.6	0.4	0.3	0.4
AF0289	rfbA-2	polysaccharide ABC transporter, permease protein	G	+					0.4 ± 0.1	0.4 ± 0.1	0.4 ± 0.1	0.3	0.3
AF0290	rfbB-2	polysaccharide ABC transporter, ATP-binding protein	G	+					0.2	0.2	0.2	0.1	0.2
AF0291		DHH superfamily phosphohydrolase/exonuclease*	L	+					0.3	0.4	0.3	0.2	0.2
AF0292		hypothetical protein	X	-					0.1	0.1	0.2	0.1	0.1
AF0293		PIN domain containing protein*	V	-					0.4	0.5	0.5	0.4 ± 0.1	0.7 ± 0.1
AF0295		hypothetical protein	X	+					0.4	0.3	0.4	0.5	0.5 ± 0.1
AF0296		PIN domain containing protein*	V	+					0.1	0.1	0.1	0.1	0.2
AF0298		HEPN domain containing protein*	V	-					0.1	0.1	0.1	0.1	0.1
AF0299		Minimal nucleotidyltransferase*	V	-			1.43		0.1	0.2	0.1	0.1	0.2
AF0300		PIN domain containing protein*	V	-					0	0	0	0	0
AF0301		uncharacterized conserved small protein*	S	-					0.1	0.1	0.1	0.1	0.1
AF0302	ugd-1	uDP-glucose dehydrogenase	M	+					0.1	0.1	0.1	0.1	0.1
AF0303		uncharacterized conserved protein*	S	+					0	0	0	0	0
AF0304		hypothetical protein	X	-					0	0	0	0	0
AF0307		hypothetical protein	X	-			1.17		0.1	0.1	0.1	0.1	0.1
AF0308		hypothetical protein	X	-			-1.14		0	0	0	0	0
AF0309		ISA0963-2 transposase	L	+					0.1	0.1	0.1	0.1	0.1
AF0310		hypothetical protein	X	-					0.1	0.1	0.1	0	0.1
AF0312		Predicted O-methyltransferase*	R	-	**1.72**				0	0	0	0.1	0.1
AF0313		PIN domain containing protein*	V	+					0.1	0.2	0.2	0.2	0.2

AF0315		PIN domain containing protein*	V	+				0.2	0.2	0.2 ± 0.1	0.2	0.3
AF0317		PIN domain containing protein*	V	+				0.2	0.2	0.2	0.2	0.2
AF0318		PIN domain containing protein*	V	+			-1.39	0.3	0.2	0.4 ± 0.1	0.4	0.3
AF0319		DNA-binding protein, potential antitoxin AbrB/MazE fold*	K	+				0.1 ± 0.1	0.1	0.2	0.2	0.2
AF0320		PIN domain containing protein*	V	+				0.1	0.1	0.1	0.1	0.1
AF0321		glycosyl transferase	R	-				0.3	0.2	0.3	0.2	0.4
AF0322	rfbQ	rhamnosyl transferase	R	-				0.9 ± 0.1	0.8 ± 0.1	0.9 ± 0.1	0.9 ± 0.1	1.3 ± 0.1
AF0323a	AF0323b	dTDP-4-dehydrorhamnose 3,5-epimerase and related enzyme	M	-				1.8 ± 0.1	1.3 ± 0.1	1.7 ± 0.1	1.7 ± 0.1	1.8
AF0323b	AF0323a	dTDP-4-dehydrorhamnose 3,5-epimerase and related enzyme	M	-				1.5 ± 0.2	1.2 ± 0.1	1.4 ± 0.1	1.4 ± 0.2	1.9 ± 0.1
AF0324	rfbB	dTDP-glucose 4,6-dehydratase	M	-				2.8	1.9 ± 0.1	2.6	2.7	2.7
AF0325	graD-2	glucose-1-phosphate thymidylyltransferase	M	-				2.1	1.4 ± 0.1	2	2.1	2.4 ± 0.2
AF0327		Glycosyltransferase*	M	+				0.3	0.3	0.3	0.3	0.3
AF0328		dolichol-P-glucose synthetase, putative	M	+		-1.27		0.5	0.6	0.5	0.4	0.4
AF0329		transmembrane oligosaccharyl transferase, putative	R	+				0.7 ± 0.1	0.5	0.6 ± 0.1	0.5 ± 0.1	0.4
AF0330		Predicted Zn-dependent protease*	R	-				0.3 ± 0.1	0.3	0.3	0.3	0.2
AF0331	traB	pheromone shutdown protein	S	-				1 ± 0.1	1	0.9	1 ± 0.1	0.9
AF0332		Zn-dependent protease fused to CBS domain*	R	-				0.5	0.6	0.4	0.5	0.6
AF0333	hpaA-1	4-hydroxyphenylacetate-3-hydroxylase	Q	+				0.9 ± 0.1	1.3 ± 0.1	0.7 ± 0.1	0.6 ± 0.1	0.5
AF0334		hypothetical protein	X	+				0.1	0.2	0.1	0.1	0.1
AF0335	pol30	DNA polymerase sliding clamp	L	+				1.8 ± 0.1	1.9 ± 0.1	1.8	1.9 ± 0.1	2.2
AF0336		DNA primase large subunit	L	+				0.5 ± 0.1	0.6 ± 0.1	0.5 ± 0.1	0.5 ± 0.1	0.7
AF0337	hpyA1-1	histone A1	L	-			**1.98**	0.8 ± 0.1	1.7 ± 0.1	0.8 ± 0.1	1.6 ± 0.1	2.5
AF0338	gspE-1	type II secretion system protein	N	-	**1.68**			0.7 ± 0.1	0.5	0.7	1.2	1.1 ± 0.1
AF0339		alcohol dehydrogenase, iron-containing	C	+	**2.34**			0.6 ± 0.1	1.7 ± 0.2	0.8 ± 0.1	2.7	2.4
AF0340	aor-3	aldehyde ferredoxin oxidoreductase	C	+	**2.42**			0.5	1.3 ± 0.1	0.6 ± 0.1	2 ± 0.1	2 ± 0.1
AF0341		Ferredoxin-thioredoxin reductase, catalytic subunit fused to rubrerythrin*	C	-				0.4	0.2	0.4 ± 0.1	0.1	0.1
AF0342		nigerythrin, putative	C	-	**-2.04**			0.6	0.4	0.7 ± 0.1	0.2	0.3
AF0343	wrbA	tryptophan repressor binding protein	R	-				1.6 ± 0.1	1.7	1.5 ± 0.1	1.5 ± 0.1	1.7 ± 0.2
AF0344		desulfoferrodoxin, putative	C	-				1.6 ± 0.1	1.6 ± 0.2	1.5 ± 0.1	1.5 ± 0.1	1.7 ± 0.2
AF0345		Acetyltransferase (GNAT) family*	R	-				0.4	0.5	0.4	0.3	0.4
AF0346	merP	mercuric transport protein periplasmic component	P	-	-1.19			1 ± 0.1	1	1	0.8	0.8
AF0347	mae1	C4-dicarboxylate transporter	P	+				0.3 ± 0.1	0.2	1.2 ± 0.1	0.4 ± 0.1	0.3 ± 0.1
AF0348		uncharacterized homolog of gamma-carboxymuconolactone decarboxylase subunit*	S	+				0.1	0.1	0.1	0.1	0.1
AF0349		Nucleotide-binding protein, uspA family*	T	+				0.2	0.2	0.2	0.1	0.1
AF0350		Predicted permease*	R	+	-1.19			0.1	0.1	0.1	0.1	0.1
AF0351		RecA-superfamily ATPase implicated in signal transduction*	T	-				1.1 ± 0.1	0.8 ± 0.1	1.1 ± 0.1	1.4 ± 0.2	1.3 ± 0.1
AF0352		uncharacterized conserved protein*	S	-				1 ± 0.1	0.9 ± 0.1	1.1 ± 0.1	1.4 ± 0.2	1.8
AF0353		KaiC-like protein*	R	-				0.6 ± 0.1	0.5	0.7 ± 0.1	0.9 ± 0.2	1
AF0354		RecA-superfamily ATPase implicated in signal transduction*	T	-				0.8 ± 0.1	0.6	0.9 ± 0.1	1.1 ± 0.2	1.3 ± 0.1
AF0355	fdx-3	ferredoxin	C	+				0.9 ± 0.1	0.8 ± 0.1	0.7 ± 0.1	0.8 ± 0.1	0.5
AF0356		carbohydrate kinase	G	+				1.3	1.6 ± 0.1	1.3 ± 0.1	1.3 ± 0.1	1.3
AF0357		Nuclease of restriction endonuclease-like fold, implicated in nucleotide excision repair*	L	-	1.39			0.5	0.6	0.6	0.8	0.9 ± 0.1
AF0358		DNA repair protein RAD25	K	-	1.43			0.3	0.4	0.4	0.6	0.6 ± 0.1
AF0359		Protein implicated in RNA metabolism, contains PRC-barrel domain*	R	+				0.3	0.1	0.2	0.3	0.2
AF0360		Predicted membrane protein*	S	+				0.5	0.3	0.6	0.5	0.5
AF0361	galE-1	uDP-glucose 4-epimerase	M	-				0.8	1	0.8 ± 0.1	0.7 ± 0.1	0.7
AF0362		snRNP, putative	K	+				**3.1**	**3.2**	**3.1**	**3.2**	**3 ± 0.2**
AF0363	cinR	transcriptional repressor	K	+				**3.3**	**3.3**	**3.3**	**3.3**	**3.3**
AF0364	lon	ATP-dependent protease Lon	O	+				2.8	2.6	2.6	2.6	2.5
AF0365		Predicted antitoxin, copG family*	V	-	**1.63**			0.1	0.1	0.1	0.2	0.2
AF0366	acs-2	acetyl-CoA synthetase	I	+	**2.08**			0.2	0.2	0.1	0.4 ± 0.1	0.4
AF0367	oxlT-2	oxalate/formate antiporter	G	+				0.2	0.1	0.1	0.2	0.2
AF0368		uncharacterized conserved small protein*	S	+		1.28		0.1	0	0.1	0.1	0.1
AF0369		hypothetical protein	X	-				0.1	0.1	0.1	0.1	0.1
AF0370	eif2BD	translation initiation factor IF-2B subunit alpha	J	-				1 ± 0.2	1	0.8 ± 0.1	0.7	0.6 ± 0.1
AF0371		N-glycosylase/DNA lyase	L	-				1.2 ± 0.1	1.2 ± 0.1	1 ± 0.1	0.9 ± 0.1	0.7
AF0372		uncharacterized protein required for formate dehydrogenase activity*	C	-				0.9 ± 0.1	1	0.8	0.7 ± 0.1	0.5
AF0373		transcription factor	K	+				2.2 ± 0.2	1.9 ± 0.1	2.1	2.2	2.2
AF0374	pho2	p-nitrophenyl phosphatase	G	+				2.3	2.1	2.2	2.3	2.3
AF0375		Predicted RNA-binding protein containing a C-terminal EMAP domain*	R	+				1.9 ± 0.1	1.5 ± 0.1	1.8 ± 0.1	1.9 ± 0.1	1.8
AF0376	cdhE	acetyl-CoA decarbonylase/synthase complex subunit gamma	C	-			1.06	**3.2**	**3.4**	**3.2**	**3.1**	**3.4**
AF0377	cdhD	acetyl-CoA decarbonylase/synthase complex subunit delta	C	-				**3.4**	**3.4**	**3.4**	**3.4**	**3.2**
AF0378	cooC-1	CODH nickel-insertion accessory protein	D	-				**3.3**	**3.3**	**3.3**	**3.3**	**3.2**
AF0379	cdhC	acetyl-CoA decarbonylase/synthase complex subunit beta	C	-				**3.4**	**3.4**	**3.5**	**3.4**	**3.4**
AF0380		transmembrane oligosaccharyl transferase, putative	R	-				1.7 ± 0.1	1.6 ± 0.1	1.6	2 ± 0.1	1.5 ± 0.1
AF0381	dph5	diphthine synthase	J	+				1	1.3	1 ± 0.1	1.3 ± 0.1	1.4 ± 0.1
AF0382		Single-stranded DNA-binding replication protein A (RPA), large (70 kD) subunit or related ssDNA-binding protein*	L	+				0.5	0.6	0.5 ± 0.1	0.6 ± 0.1	0.5
AF0383		endonuclease III, putative	L	-				0.2	0.2	0.2	0.2	0.1
AF0384		Predicted transglutaminase-like protease*	R	+	**-1.79**			0.6	0.4	0.4	0.3	0.2
AF0385		Endonuclease Nob1, consists of a PIN domain and a Zn-ribbon module*	V	+				2.1 ± 0.2	2.1	2.1	2.1	1.8
AF0386		orotate phosphoribosyltransferase	F	+				2.8	2.9	2.9	2.9	2.9
AF0387		glycosyltransferase, putative	M	+				2.1	1.8 ± 0.1	2.1 ± 0.2	2.1	2.3 ± 0.2
AF0388		Thiamine biosynthesis enzyme ThiH, FO synthase or related uncharacterized enzyme*	H	+				2.3 ± 0.2	2.5 ± 0.2	2.4 ± 0.2	2.3 ± 0.2	2.6 ± 0.2
AF0389		Predicted periplasmic solute-binding protein*	R	+				2 ± 0.3	2.1 ± 0.2	2 ± 0.2	1.9 ± 0.2	2.2
AF0390		Thiamine biosynthesis enzyme ThiH, FO synthase or related uncharacterized enzyme*	H	+				1.4 ± 0.2	1.6 ± 0.2	1.4 ± 0.2	1.2 ± 0.2	1.6 ± 0.2
AF0391		Predicted metal-binding protein*	S	+				0.5 ± 0.1	0.4	0.4 ± 0.1	0.3 ± 0.1	0.3
AF0392		Predicted permease*	R	-				1.6 ± 0.1	1 ± 0.1	1	0.8 ± 0.1	0.9 ± 0.1
AF0393		ABC transporter, ATP-binding protein	V	-				1.2 ± 0.1	0.8 ± 0.1	0.6 ± 0.1	0.6 ± 0.1	0.6 ± 0.1
AF0394	dld	D-lactate dehydrogenase, cytochrome-type	C	-	1.39			0.4	0.4	0.4	0.5	0.6
AF0395	noxA-2	NADH oxidase	P	-	**1.82**			1 ± 0.1	1.5 ± 0.1	1.4 ± 0.1	2.3	2.6 ± 0.2
AF0396		Predicted transcriptional regulator*	K	-				0.2	0.1	0.1	0.1	0.1
AF0397		hypothetical protein	X	+		**-1.65**		0.6	0.7	0.4	0.3 ± 0.1	0.3
AF0398		hypothetical protein	X	+		**-1.54**		0.9 ± 0.1	1.1	0.8	0.6 ± 0.1	0.5 ± 0.1
AF0399		hypothetical protein	X	+	**-1.80**			0.1	0.1	0.1	0.1	0.1
AF0400	noxA-3	NADH oxidase	P	-	-1.30			**3**	**3.3**	2.9	2.4	2.2
AF0401		carbohydrate kinase	G	+				0.3	0.3	0.3	0.3 ± 0.1	0.2
AF0402		uncharacterized conserved protein*	S	+				0.5	0.6	0.6 ± 0.1	0.7	0.6
AF0403		geranylgeranylglyceryl phosphate synthase-like protein	R	+				1.8 ± 0.2	2.1	1.8 ± 0.1	2 ± 0.2	2
AF0404	ubiA	prenyltransferase	H	+				1.3 ± 0.2	1.4 ± 0.1	1.1	1.2 ± 0.1	1.2 ± 0.1
AF0405		lysine biosynthesis protein LysW*	E	+				0.2	0.2	0.1	0.1	0.2
AF0406		RNA 2'-phosphotransferase-like protein	J	-				2.2	1.6 ± 0.1	2	1.9 ± 0.1	1 ± 0.1
AF0407		uncharacterized conserved protein*	S	-				1.5 ± 0.1	1.2 ± 0.1	1.4 ± 0.1	1.3 ± 0.1	0.8 ± 0.1
AF0408		uncharacterized conserved protein*	S	-	-1.34			1	1.1	1	0.8	0.7
AF0409	aspB-4	aspartate aminotransferase	E	+				1.5 ± 0.1	1.9	1.4 ± 0.1	1.4 ± 0.1	1.2 ± 0.1
AF0410		signal-transducing histidine kinase, putative	T	+				2.1 ± 0.2	2	2.1	1.9 ± 0.1	1.7 ± 0.1
AF0411	cysS	cysteinyl-tRNA synthetase	J	+				1.1	1	1	0.9	0.8
AF0412		Membrane-bound metal-dependent hydrolase*	R	+				1 ± 0.1	0.9	0.8 ± 0.1	0.7 ± 0.1	0.9 ± 0.1
AF0413		uncharacterized conserved protein*	S	+	**-2.14**			0.8 ± 0.1	0.6	0.8 ± 0.1	0.3	0.3
AF0414		hypothetical protein	X	-	**-2.58**			2.6	2.5	2.5 ± 0.2	1.1 ± 0.2	0.7 ± 0.1
AF0415		hypothetical protein	X	-	**-1.80**			**3.1**	**3**	**3**	1.8 ± 0.3	1.3 ± 0.2
AF0416		hypothetical protein	X	-	**-1.81**			**3.2**	**3.2**	**3**	1.7 ± 0.3	1.4 ± 0.3
AF0417		hypothetical protein	X	-	**-2.06**			2.9	2.7	2.6	1.4 ± 0.2	1 ± 0.1
AF0418		Predicted Ser/Thr protein kinase*	T	-				0.1	0.1	0.1	0.1	0.1

AF0419		Predicted TIM-barrel enzyme*	R	-				0.2	0.2	0.2	0.2	0.2
AF0420		uncharacterized conserved protein*	S	-				0.3	0.3	0.4	0.3	0.3
AF0421		ArsR family transcriptional regulator*	K	-				0.5	0.6	0.6 ± 0.1	0.5	0.5
AF0422	cysG-1	uroporphyrin-III C-methyltransferase	H	-	**-1.58**			0.3	0.4	0.3	0.2	0.2
AF0423	dsrA	sulfite reductase, subunit alpha	C	+				**3.5**	**3.5**	**3.5**	**3.4**	**3.2 ± 0.2**
AF0424	dsrB	sulfite reductase, subunit beta	C	+				**3.5**	**3.5**	**3.5**	**3.5**	**3.4**
AF0425	dsrD	sulfite reductase, subunit gamma	C	+				1.8 ± 0.1	2.4	1.7	1.3 ± 0.1	1.7 ± 0.1
AF0426		uncharacterized conserved protein*	S	+				0.5 ± 0.1	0.4 ± 0.1	0.4 ± 0.1	0.2 ± 0.1	0.3
AF0427	fdx-4	ferredoxin	C	+				0.5 ± 0.1	0.8	0.5 ± 0.1	0.3 ± 0.1	0.5 ± 0.1
AF0428		GTP1/OBGfamily GTP-binding protein	R	-				0.7	0.6	0.7	0.7	0.5 ± 0.1
AF0429		methyltransferase	Q	-				0.5 ± 0.1	0.4	0.9 ± 0.1	0.7 ± 0.2	0.6 ± 0.2
AF0430	hemV-1	iron ABC transporter, ATP-binding protein	P	-				0.6 ± 0.1	0.4	1 ± 0.2	1 ± 0.3	0.8 ± 0.2
AF0431	hemU-1	iron ABC transporter, permease protein	P	-				0.9 ± 0.2	0.6	1.4 ± 0.2	1.4 ± 0.3	1.2 ± 0.3
AF0432	hemV-2	iron ABC transporter, ATP-binding protein	P	-				2.2 ± 0.2	1.7 ± 0.1	2.9	2.9 ± 0.2	2.4 ± 0.4
AF0433		uncharacterized conserved protein*	S	-				0.9 ± 0.3	0.8 ± 0.1	1.5 ± 0.2	1.4 ± 0.3	1.3 ± 0.3
AF0434	hbd-3	3-hydroxyacyl-CoA dehydrogenase	I	-				0.7 ± 0.1	1	0.6	0.4	0.5
AF0435	fad-1	enoyl-CoA hydratase	I	-				1.1 ± 0.1	1.4 ± 0.1	1 ± 0.1	0.8 ± 0.1	0.8
AF0436	acd-2	acyl-CoA dehydrogenase	I	-				1 ± 0.1	1.2	0.9	0.7 ± 0.1	0.7
AF0437		Predicted nucleic-acid-binding protein containing a Zn-ribbon*	R	-	**-1.62**			0.8	0.9 ± 0.1	0.7 ± 0.1	0.5 ± 0.1	0.4
AF0438	acaB-8	acetyl-CoA acetyltransferase	I	-				0.9	1.3	0.9	0.6 ± 0.1	0.8
AF0439		AsnC family transcriptional regulator	K	-				0.7 ± 0.1	0.4	0.7	0.7 ± 0.1	0.7
AF0440		6-pyruvoyl-tetrahydropterin synthase*	H	-		-1.47		0.3	0.3	0.2	0.2	0.2
AF0441		Organic radical activating enzyme*	O	-				0.6	0.5	0.5 ± 0.1	0.4	0.3
AF0442	exsB	succinoglycan biosynthesis regulator	R	-				0.9 ± 0.1	0.8	0.9 ± 0.1	0.7 ± 0.1	0.5
AF0443		hypothetical protein	S	+				0.1	0.1	0.1	0.1	0.3
AF0444		phosphoglycolate phosphatase	R	+	**-1.53**			0.2	0.2	0.2	0.1	0.1
AF0445		Predicted Fe-S-cluster oxidoreductase*	R	-				0.1	0.1	0.1	0.1	0.1
AF0446		LEA14-like dessication related protein*	V	-				0.1	0.2	0.1	0.1	0.1
AF0447		LEA14-like dessication related protein*	V	-				0.2	0.2	0.2 ± 0.1	0.2 ± 0.1	0.2
AF0448		signal-transducing histidine kinase, putative	T	+	**2.34**			0.6 ± 0.1	0.6	0.8 ± 0.1	1.4 ± 0.1	1.9 ± 0.1
AF0449		response regulator	T	+				1 ± 0.1	1.1	1.2 ± 0.1	1.4 ± 0.1	1.9 ± 0.1
AF0450		signal-transducing histidine kinase	T	+				1.3 ± 0.2	1.5	1.4 ± 0.2	1.4 ± 0.1	2.2
AF0451		uncharacterized conserved protein*	S	+				0.7 ± 0.1	0.5	0.8 ± 0.1	0.7 ± 0.1	0.9
AF0452		RecA-superfamily ATPase implicated in signal transduction*	T	+				2 ± 0.1	1.9	1.9 ± 0.1	1.7 ± 0.1	1.9 ± 0.1
AF0453		hypothetical protein	X	+	-1.27			0.6	0.5	0.6	0.4	0.4
AF0454		Signal transduction histidine kinase and PAS domains*	T	+				0.2	0.2	0.2	0.2	0.3
AF0455	noxB-1	NADH oxidase	C	-				2.2	2.5	2.1	1.8 ± 0.1	1.9 ± 0.1
AF0456		Putative redox-active protein*	R	+			-1.23	0.1	0	0.1	0.1	0.1
AF0457		hypothetical protein	X	+	**1.54**			0.1	0.1	0.1	0.2	0.2
AF0458	pmm	phosphomannomutase	G	-				0.6 ± 0.1	0.3 ± 0.1	0.6 ± 0.1	0.4 ± 0.1	0.4
AF0459		Predicted exporter of the RND superfamily*	R	+				0.1	0.1	0.1	0.1	0.1
AF0460		uncharacterized conserved protein*	S	-	-1.45			0.6	0.7	0.6	0.4	0.4
AF0461		S-adenosyl-l-methionine hydroxide adenosyltransferase*	F	-				0.4	0.3	0.3	0.3	0.3
AF0462		Predicted membrane protein*	S	-				0.4	0.4	0.4 ± 0.1	0.3	0.5
AF0463m	mvhB	polyferredoxin	C	+				0.6	0.8	0.7	0.6	0.6 ± 0.1
AF0464	chlP-1	bacteriochlorophyll synthase, 43 kDa subunit	C	+				1.5 ± 0.1	1.7	1.4 ± 0.1	1.4 ± 0.1	1.2 ± 0.1
AF0465	gyrA	DNA gyrase, subunit A	L	-				1.9 ± 0.1	2.3	1.8 ± 0.1	1.7 ± 0.1	1.6 ± 0.1
AF0466		TRAP-type uncharacterized transport system, fused permease component*	R	-	**2.02**			0.2	0.2	0.1	0.3 ± 0.1	0.3
AF0467	bcsp31-1	immunogenic protein	R	-				0.6	2.3	0.6	2.8	2.9
AF0468	korB	2-oxoglutarate ferredoxin oxidoreductase subunit beta	C	-				0.4	0.4	0.4	0.8 ± 0.2	1.3 ± 0.3
AF0469	korA	2-oxoglutarate ferredoxin oxidoreductase subunit alpha	C	-				0.4	0.6	0.5	0.9 ± 0.2	2 ± 0.7
AF0470	korD	2-ketoglutarate ferredoxin oxidoreductase, subunit delta	C	-				0.2	0.2	0.2	0.6 ± 0.2	1.3 ± 0.8
AF0471	korG	2-ketoglutarate ferredoxin oxidoreductase, subunit gamma	C	-				0	0	0	0.1	0.3 ± 0.3
AF0472		Phosphate uptake regulator*	P	+	**2.23**			0.1	0.1	0.1	0.1	0.2
AF0473	pacS	cation-transporting ATPase, P-type	P	-				0.4	0.4	0.3	0.3	0.3
AF0474		AsnC family transcriptional regulator	P	+				0.2	0.2	0.2	0.1	0.1
AF0475		Arsenite efflux pump ACR3 or related permease*	P	-				0.5 ± 0.1	0.5 ± 0.1	0.6 ± 0.1	0.6 ± 0.1	0.5 ± 0.1
AF0476		Transcriptional regulator AbrB*	K	-				0.3	0.3	0.3 ± 0.1	0.3 ± 0.1	0.3
AF0477		AAA ATPase family protein	O	+				1.3 ± 0.1	1.1 ± 0.1	1.3 ± 0.1	1.1 ± 0.1	1.1 ± 0.1
AF0478	phnP	ATP-binding protein PhnP	R	+				2.1 ± 0.2	1.8 ± 0.2	2.1 ± 0.2	1.9 ± 0.3	2.1 ± 0.2
AF0479		Predicted transcriptional regulator/sugar kinase*	K	+				1.1 ± 0.2	0.9 ± 0.1	1 ± 0.1	0.8 ± 0.1	0.9
AF0480	fucA	fuculose-1-phosphate aldolase	G	-				0.3	0.3	0.3	0.2	0.3
AF0481	psmB	proteasome, subunit beta	O	+				2.9	2.9	**3.1**	**3.1**	**3 ± 0.2**
AF0482		mRNA 3'-end processing factor, putative	R	+				2.1 ± 0.2	1.9 ± 0.1	2.2 ± 0.2	2 ± 0.2	1.9 ± 0.1
AF0483		uncharacterized conserved protein*	S	+				0.4 ± 0.1	0.3	0.4 ± 0.1	0.2 ± 0.1	0.3
AF0484	ribA-1	GTP cyclohydrolase II	H	+	-1.31			1.2 ± 0.1	1.3 ± 0.1	1.1 ± 0.1	0.8 ± 0.1	0.9
AF0485		Predicted metal-binding protein*	R	+				0.9 ± 0.1	0.7 ± 0.1	0.8 ± 0.1	0.6 ± 0.1	0.6
AF0486		Predicted Fe-S-cluster oxidoreductase*	R	+				1 ± 0.2	0.7 ± 0.1	0.9 ± 0.2	0.6 ± 0.2	0.7
AF0487		penicillin G acylase	R	+	**2.01**			0.9 ± 0.1	1.5 ± 0.1	0.9	2.2	2.3
AF0488		hypothetical protein	X	+	**3.11**			0.2	0.5	0.3	0.9 ± 0.1	1.2
AF0489		RNase P/RNase MRP subunit POP5*	J	+				1.1 ± 0.1	1	1.1 ± 0.1	1.4	1.3 ± 0.1
AF0490	psmA	proteasome subunit alpha	O	+				2 ± 0.2	2.2	2.2	2.3	2.4 ± 0.2
AF0491		putative RNA-associated protein	J	+				**3.1**	**3.1**	**3.2**	**3.3**	**3.2**
AF0492		exosome complex RNA-binding protein Rrp4	J	+				2.6 ± 0.2	2.6	2.7	2.8	2.7
AF0493	rph	exosome complex exonuclease Rrp41	J	+				2.9	2.8	2.9	2.9	2.9
AF0494		exosome complex RNA-binding protein Rrp42	J	+			1.21	1.4 ± 0.1	1.7 ± 0.1	1.3 ± 0.1	1.3	1.6 ± 0.1
AF0495		hypothetical protein	X	+		**-1.78**		0.1	0.1	0	0	0
AF0496		uncharacterized conserved protein*	S	+				0.1	0.1	0.1	0.1	0.1
AF0497	polB	DNA polymerase B1	L	+				0.7 ± 0.1	0.7	0.7	0.8	0.8
AF0498	acd-3	acyl-CoA dehydrogenase	I	+	**-1.54**	-1.49		2.9	**3.2**	2.4	1.9 ± 0.1	1.7 ± 0.2
AF0499		molybdopterin oxidoreductase, iron-sulfur binding subunit	C	+				**3.3**	**3.3**	**3.2**	**3.2**	**3.1**
AF0500		molybdopterin oxidoreductase, membrane subunit	C	+				**3.6**	**3.5**	**3.6**	**3.6**	**3.6**
AF0501		nitrate reductase, gamma subunit, putative	C	+				**3.6**	**3.5**	**3.6**	**3.6**	**3.6**
AF0502		heterodisulfide reductase, subunit D, putative	C	+				**3.4**	**3.4**	**3.4**	**3.4**	**3.3**
AF0503		uncharacterized conserved protein*	S	+				2.1	2.2	1.9 ± 0.1	1.7 ± 0.1	1.8
AF0504		Predicted hydrolase (metallo-beta-lactamase superfamily)*	R	-	-1.29			0.3	0.3	0.3	0.2	0.2
AF0505		Metal-dependent phosphoesterase (PHP family)*	R	+				0.1	0.1	0.1	0.1	0.1
AF0506		iron-sulfur binding reductase	C	-	**-2.00**			0.7 ± 0.1	0.5	0.7	0.3	0.3
AF0507		FAD/FMN-containing dehydrogenase fused to Heterodisulfide reductase, subunit B*	C	-	**-1.69**			1.4 ± 0.1	1.2 ± 0.1	1.5 ± 0.1	0.7 ± 0.1	0.8 ± 0.1
AF0508		pyridoxine biosynthesis protein	H	+				2.4	2.4	2.4	2.2	2.4
AF0509		glutamine amidotransferase subunit PdxT	H	+				2 ± 0.3	1.7 ± 0.1	2.2	1.9 ± 0.2	2 ± 0.2
AF0510		Permease of the drug/metabolite transporter (DMT) superfamily*	G	+				0.7 ± 0.2	0.4 ± 0.1	0.8 ± 0.1	0.6 ± 0.1	0.6
AF0511	rps6E	30S ribosomal protein S6e	J	+				**3.1**	**3.1**	2.8	2.9	2.7 ± 0.2
AF0512		chloroplast inner envelope membrane protein	Q	+				1.6 ± 0.3	1.3 ± 0.2	1.7 ± 0.1	1.4 ± 0.2	1 ± 0.1
AF0513		allene oxide synthase, putative	S	+				1.5	1.5 ± 0.1	1.5 ± 0.1	1.3 ± 0.1	1 ± 0.1
AF0514		Alpha/beta superfamily hydrolase*	R	-	-1.47			0.6	0.5	0.6	0.4	0.4
AF0515		NADH oxidase, putative	O	-	-1.35			0.7 ± 0.1	0.6	0.7	0.5	0.5
AF0516		Predicted ATPase involved in replication control, Cdc46/Mcm family*	L	+				1.1 ± 0.2	0.9 ± 0.1	1.2 ± 0.1	1.1 ± 0.1	0.9 ± 0.1
AF0517	cdc21	cell division control protein 21	L	+			-1.18	1.9 ± 0.2	1.7	2.1	2.1	1.7 ± 0.1
AF0518		RecA-superfamily ATPase implicated in signal transduction*	T	+				2.1	2	2.2	2.2	2.4
AF0519		Subunit of KEOPS complex, involved (Cgi121BuD32KAE1)*	J	+				2.2	2.5	2.3	2.4	**3**
AF0520		Archaeal shikimate kinase*	E	+				1.2 ± 0.1	1.1	1.2 ± 0.1	1.2 ± 0.1	1.4
AF0521		protease synthase and sporulation regulator Pai1, putative	K	-	**-1.74**			0.6 ± 0.1	0.6 ± 0.1	0.5 ± 0.1	0.3	0.3

AF0522	argE	acetylornithine deacetylase	E	+				0.2	0.2	0.1	0.1	0.1
AF0523		uncharacterized conserved protein*	S	+		**-1.51**		0.6	0.6	0.4	0.3	0.4
AF0524		Predicted membrane protein*	S	-				0.3	0.3	0.3	0.2	0.3
AF0525		Archaeal enzyme of ATP-grasp superfamily*	R	-				1.6 ± 0.2	1.6 ± 0.1	1.6 ± 0.1	1.6 ± 0.1	1.7
AF0526		Predicted Zn-ribbon RNA-binding protein*	J	-				2.2 ± 0.2	2.5	2.3	2.2	2.6
AF0527	eif2A	translation initiation factor IF-2 subunit alpha	J	-				2.7	2.4	2.7	2.7 ± 0.2	2.5
AF0528		cytochrome-c3 hydrogenase, subunit gamma	H	-				0.3	0.3	0.3	0.3	0.2
AF0529		Transcriptional regulator containing HTH domain, ArsR family*	K	+				0.1	0.1	0	0	0
AF0530	gyrB	DNA gyrase, subunit B	L	+				2.8	2.8	2.9	**3.2**	**3**
AF0531	moeB	molybdenum cofactor biosynthesis protein	H	+				0.9 ± 0.1	0.6	1 ± 0.1	1.2 ± 0.1	0.9
AF0532		mRNA 3'-end processing factor, putative	J	+				2.4	2.1	2.4	2.3 ± 0.2	2.2
AF0533		Icc family phosphoesterase*	R	+				0.5 ± 0.1	0.5	0.5 ± 0.1	0.4 ± 0.1	0.4
AF0534		Metal-dependent hydrolase of the beta-lactamase superfamily II*	R	-			-1.47	0.4	0.2	0.3	0.3	0.2
AF0535	ftsZ-1	cell division protein FtsZ	D	+				2.9	2.6	**3**	**3.1**	**3.1**
AF0536	secE	protein translocase, subunit SEC61 gamma	U	+				**3.1**	**3**	**3.1**	**3.2**	**3.2**
AF0537	nusG	transcription antitermination protein NusG	K	+			1.09	2.9	**3.2**	2.9	**3**	**3.2 ± 0.2**
AF0538	rpl11P	50S ribosomal protein L11P	J	+			1.43	1.7 ± 0.1	2.7	1.6 ± 0.1	1.7 ± 0.1	2.3
AF0539		GTPase	R	-				0.4	0.4	0.3	0.2	0.4
AF0540		uncharacterized conserved protein*	S	-				1.3 ± 0.1	1.2	1.2 ± 0.1	1.2 ± 0.1	1.6 ± 0.1
AF0541		hypothetical protein	X	-				0.5 ± 0.1	0.5	0.5	0.5 ± 0.1	0.5
AF0542m		Homolog of Wybutosine (yW) biosynthesis enzyme, Fe-S oxidoreductase*	J	+			**-1.68**	0.1	0.1	0.1	0.1	0.1
AF0543		Fe-S oxidoreductase*	C	-				**3.1**	2.8	2.9	2.7	2.6
AF0544		Fe-S oxidoreductase*	C	-				**3.6**	**3.3**	**3.5**	**3.4**	**3.5**
AF0545		Nitrate reductase gamma subunit*	C	-				**3.7**	**3.3**	**3.6**	**3.5**	**3.3 ± 0.2**
AF0546	narI	nitrate reductase, gamma subunit	C	+				1 ± 0.2	0.8 ± 0.1	0.9 ± 0.1	1.1 ± 0.2	0.6
AF0547		reductase, iron-sulfur binding subunit	C	+				0.8 ± 0.2	0.9 ± 0.1	0.8 ± 0.1	0.9 ± 0.2	0.6
AF0548	thrS	threonyl-tRNA synthetase	J	+				1.7 ± 0.1	2	1.4 ± 0.1	1.4 ± 0.1	1.2 ± 0.1
AF0549		uncharacterized conserved membrane protein*	S	+				0.6	0.8	0.5	0.4	0.4
AF0550	trzA-1	N-ethylammeline chlorohydrolase	F	-				0.8 ± 0.1	0.9 ± 0.1	0.8	0.6 ± 0.1	0.5
AF0551	thrC-1	threonine synthase	E	+				0.1	0.1	0.1	0	0.1
AF0552		Molybdopterin converting factor, small subunit*	H	+				0.1	0.1	0.1	0.1	0.1
AF0553	thiF	thiamine biosynthesis protein	H	+	-1.43			0.1	0.1	0.1	0.1	0.1
AF0554		Predicted redox protein, regulator of disulfide bond formation*	O	+				0.1	0.2	0.1	0.1	0.1
AF0555		Peroxiredoxin family protein*	R	+		**1.92**		0.7 ± 0.1	0.7	1.5 ± 0.2	1.4 ± 0.1	1.4 ± 0.1
AF0556		Predicted redox protein, regulator of disulfide bond formation*	O	+		**1.71**		1.2 ± 0.1	1.3 ± 0.1	2.2 ± 0.2	2.2 ± 0.2	2.3
AF0557		flavoprotein reductase	R	+		1.48		1.2 ± 0.1	1.4 ± 0.1	1.9 ± 0.1	2 ± 0.1	2
AF0558		uncharacterized conserved protein*	S	+				0.1	0.1	0.1	0.1	0.2
AF0559		ATP-utilizing enzyme of the PP-loop superfamily*	R	+				0.2	0.3	0.3	0.2	0.2
AF0562		hypothetical protein	X	-	-1.25			0.1	0.1	0.1	0.1	0.1
AF0563		Predicted peroxiredoxin*	R	+			-1.18	1 ± 0.1	0.9	1.1	1.1 ± 0.1	1
AF0564	nifS-2	nifS protein, class-V aminotransferase	E	+				2.2	1.8	2.4	2.5	2.3
AF0565	nifU-2	nifu protein	C	+		1.20		1.6 ± 0.1	1.4 ± 0.1	1.8 ± 0.1	1.8 ± 0.1	1.8 ± 0.1
AF0566		Predicted transcriptional regulator*	R	+			1.34	0.7 ± 0.1	0.9	0.7	0.5 ± 0.1	0.9
AF0567		Acyl-CoA synthetase (AMP-forming)/AMP-acid ligase II*	I	-				0.2 ± 0.1	0.2	0.2	0.1	0.1
AF0568		Acyl-CoA synthetase (AMP-forming)/AMP-acid ligase II*	I	-				0	0	0	0	0
AF0569		DR-beta chain MHC class II	V	+				2.3 ± 0.2	2.7	2.7 ± 0.2	2.5	2.5
AF0570	ftsZ-2	cell division protein FtsZ	D	+				**3.1**	**3.3**	**3.2**	**3.2**	**3.2**
AF0571		uncharacterized conserved protein*	S	+				0.8 ± 0.2	0.8 ± 0.1	1 ± 0.1	0.5 ± 0.1	0.4
AF0572		uncharacterized archaeal kinase related to aspartokinase, uridylate kinase*	R	+				0.5	0.4	0.5 ± 0.1	0.4 ± 0.1	0.3
AF0573		Predicted Zn-ribbon RNA-binding protein with a function in translation*	J	+				1.7 ± 0.1	2.3	1.8 ± 0.1	1.4 ± 0.1	1.7 ± 0.1
AF0574		translation elongation factor EF-1, subunit beta	J	+				2.3	2.9	2.2	1.9 ± 0.1	2
AF0575		Metal-dependent hydrolase of the beta-lactamase superfamily II*	R	+		-1.27		2.3	2.6	2.1	1.8 ± 0.1	1.8 ± 0.1
AF0576		hypothetical protein	X	+	**-1.68**			1.4 ± 0.2	1.5 ± 0.1	1.4 ± 0.1	0.9 ± 0.2	0.7
AF0577		Radical SAM superfamily enzyme*	R	+	**-2.06**			0.7 ± 0.1	0.7 ± 0.1	0.6	0.3 ± 0.1	0.2
AF0578		aminopeptidase, putative	R	-	**2.36**			0.2	0.3	0.1	0.5	0.4
AF0579		Signal transduction histidine kinase, contains PAS domain*	T	+				0.1	0.1	0.1	0.1	0.2
AF0580	xthA	exodeoxyribonuclease III	L	+				0.7	0.9	0.6 ± 0.1	0.7	0.7
AF0581		dolichol-P-glucose synthetase, putative	M	+				0.9 ± 0.1	0.8	0.8	0.7 ± 0.1	0.8
AF0582		uncharacterized membrane protein*	S	+				0.7 ± 0.1	0.6	0.7	0.5	0.5
AF0583		uncharacterized membrane protein*	S	+				0.2	0.3	0.2	0.2	0.2
AF0584		AsnC family transcriptional regulator	K	+				0.1	0.1	0.1	0.1	0.1
AF0585		uncharacterized conserved protein*	S	+				0.2	0.2	0.3	0.3 ± 0.1	0.3
AF0586		hypothetical protein	X	+				0.1	0.1	0.1	0.1	0.1
AF0587		Queuine tRNA-ribosyltransferase, contain PuA domain*	J	-				1.4	1.6	1.3	1.3 ± 0.1	1.2 ± 0.1
AF0588	tgtA	7-cyano-7-deazaguanine tRNA-ribosyltransferase	J	-				2.5	2	2.3 ± 0.2	2.3	1.5 ± 0.1
AF0589	prsA-1	ribose-phosphate pyrophosphokinase	F	+			**-1.74**	0.8 ± 0.1	0.5	0.8	0.7 ± 0.1	0.3
AF0590	hisG	ATP phosphoribosyltransferase	E	+				1.2 ± 0.1	1.1	1.1	1 ± 0.1	0.7 ± 0.1
AF0591		PIN domain containing protein*	R	-	**-1.57**			0.9	0.9 ± 0.1	0.9 ± 0.1	0.7	0.5
AF0592	eif2G	translation initiation factor IF-2 subunit gamma	J	-	-1.23			2.2	2.3	2	1.8	1.6 ± 0.1
AF0593		HEPN domain containing protein*	V	+				0.4	0.3	0.4	0.3	0.3
AF0594		Minimal nucleotidyltransferase*	V	+				0.5 ± 0.1	0.4	0.5 ± 0.1	0.3	0.3
AF0595		polysaccharide biosynthesis protein, putative	R	+				0.2	0.2	0.2	0.1	0.1
AF0596	ugd-2	uDP-glucose dehydrogenase	M	+				0.2	0.4	0.2	0.2	0.3
AF0597		Predicted antitoxins containing the HTH domain*	V	+				0.1	0.1	0.1	0.1	0.2
AF0598		PIN domain containing protein*	V	+				0.1	0.1	0.1	0.1	0.2
AF0599		dolichol phosphate mannose synthase, putative	M	-				0.1	0.1	0.1	0.1	0.1
AF0600		hypothetical protein	X	-				0.4	0.3	0.3	0.3	0.4
AF0601		SAM-dependent methyltransferase*	Q	-			**1.78**	0.1	0.2	0.1	0.1	0.2
AF0602		LPS biosynthesis protein, putative	M	-			**1.54**	0.1	0.1	0.1	0.1	0.1
AF0604		hypothetical protein	X	-				0	0	0	0	0
AF0605		hypothetical protein	X	-				0	0	0	0	0
AF0606	wbaZ-2	first mannosyl transferase	M	-			1.46	0.1	0.2	0.1	0.1	0.2
AF0607		LPS glycosyltransferase, putative	M	-			1.32	0.4	0.5	0.3	0.3	0.4
AF0608		RHH/CopG DNA binding protein*	V	-				0	0	0	0	0
AF0609		hypothetical protein	X	+				0	0	0	0	0
AF0610m		PIN domain containing protein*	V	-				0	0	0	0	0
AF0611		uncharacterized conserved protein*	S	+				0.1	0.1	0.1	0.1	0
AF0612		Pepsin-like aspartate protease*	E	-				0.2	0.4	0.3	0.3	0.3
AF0613		CopG family DNA-binding protein*	V	-			1.45	0.4	0.7	0.4	0.4	0.6
AF0614		Minimal nucleotidyltransferase*	V	-				0	0	0	0	0
AF0615		Predicted membrane protein*	S	-				0	0	0	0	0
AF0616		4-amino-4-deoxy-L-arabinose transferase or related glycosyltransferase of PMT family*	M	-				0.2	0.2	0.2	0.2	0.3
AF0617		LPS biosynthesis protein, putative	M	-				0	0	0	0	0
AF0618		PIN domain containing protein*	V	-				0	0	0	0	0
AF0619		hypothetical protein	X	-			1.20	0	0.1	0	0	0.1
AF0620	exoM	succinoglycan biosynthesis protein	M	+				0.1	0.1	0.1	0.1	0.1
AF0621	rnhB	ribonuclease HII	L	-				0.4	0.6	0.4	0.4	0.5
AF0622	srp54	signal recognition particle protein Srp54	U	-				2.3	2.4	2.3	2.3	2.2
AF0623	lig	DNA ligase	L	+			1.23	1.2 ± 0.1	1.6 ± 0.1	1.2	1.3	1.6 ± 0.1
AF0624		uncharacterized membrane protein*	S	+				1	1	1	1 ± 0.1	1.2
AF0625		D-aminoacyl-tRNA deacylase, involved in ethanol tolerance*	J	+	-1.39			0.5	0.5	0.5	0.4	0.4
AF0626		uncharacterized conserved protein*	S	-	**-1.52**			0.2	0.1	0.2	0.1	0.1
AF0627		iron-sulfur cluster binding protein	C	-				0.6	1.2 ± 0.1	0.6	0.8 ± 0.1	0.8

AF0628	leuB	3-isopropylmalate dehydrogenase	C	-			1.15	2.2	2.8	2.3	2.2	2.5
AF0629	leuD	3-isopropylmalate dehydratase small subunit	E	-				1.2 ± 0.1	1.6 ± 0.1	1.2 ± 0.1	1.2 ± 0.1	1.4 ± 0.1
AF0630		uncharacterized membrane protein*	S	-				1.2 ± 0.4	1.1 ± 0.2	1.5 ± 0.4	1.1 ± 0.4	1.5 ± 0.1
AF0631		hypothetical protein	X	-			1.22	1.5 ± 0.1	2 ± 0.1	1.8 ± 0.2	1.5 ± 0.1	1.9 ± 0.1
AF0632	nifU-3	nifu protein	O	-			1.17	2 ± 0.3	2.5	2.2 ± 0.2	2.1	2.6
AF0633	ileS	isoleucyl-tRNA synthetase	J	-				2.3	2.6	2.2	2.1	1.9 ± 0.1
AF0634		uncharacterized conserved protein related to dihydrodipicolinate reductase*	S	+				0.5	0.6	0.4	0.3 ± 0.1	0.3
AF0635	bcsp31-2	immunogenic protein	R	-				2.6	2.7	2.5	2.3	2.6
AF0636		TRAP-type uncharacterized transport system, fused permease component*	R	-				1 ± 0.1	0.8	0.9 ± 0.1	0.8 ± 0.1	0.6
AF0637		uncharacterized conserved protein*	S	-				0.2	0.3	0.2	0.2	0.2
AF0638	nrtC-2	nitrate ABC transporter, ATP-binding protein	P	-				0	0	0	0	0
AF0639	nrtB-2	nitrate ABC transporter, permease protein	P	-				0.2	0.1	0.2	0.1	0.1
AF0640		nitrate ABC transporter, ATP-binding protein, putative	P	-	**-1.50**			0.1	0.1	0.1	0.1	0.1
AF0643		Predicted transcriptional regulator with an HTH domain*	K	+			-1.31	0.2	0.1	0.2	0.2	0.1
AF0644		hypothetical protein	X	-				0.1	0.1	0.1	0.1	0.1
AF0645	eif5A	translation initiation factor IF-5A	J	+				0.6 ± 0.1	0.5 ± 0.1	0.6	0.4	0.5
AF0646	speB	agmatinase	E	+				0.6 ± 0.1	0.5	0.7 ± 0.1	0.4 ± 0.1	0.5 ± 0.1
AF0647	icd	isocitrate dehydrogenase, NADP	C	+				2.4	2.7	2.2	2.1	2
AF0648		Dehydrogenase (flavoprotein)*	C	+				1.5	1.9	1.3	1.2 ± 0.1	1.2
AF0649		Nucleoside-diphosphate-sugar pyrophosphorylase*	R	+				0.2	0.3	0.2	0.1	0.1
AF0650		hypothetical protein	X	+				0.2	0.2	0.3 ± 0.1	0.3 ± 0.1	0.3
AF0651		Lrp/AsnC family C-terminal domain*	K	+				0.5 ± 0.1	0.5	0.5 ± 0.1	0.7 ± 0.2	0.4
AF0652	top6B	DNA topoisomerase VI subunit B	L	+				1.3 ± 0.1	1.5	1.3	1.6 ± 0.1	1.4 ± 0.1
AF0653		Predicted transcriptional regulator*	K	-				0.1	0.2	0.1	0.1	0.2
AF0654		uncharacterized FAD-dependent dehydrogenase*	R	+				**3**	2.8	2.8	2.7	2.5
AF0655		Predicted Zn-dependent protease or their inactivated homolog*	R	+				**3**	**3**	**3**	2.8	**3**
AF0656	pmbA	antibiotic maturation protein	R	+				1.9 ± 0.1	2 ± 0.1	1.9 ± 0.2	1.5 ± 0.1	1.6 ± 0.1
AF0657		Cell suface protein*	S	-				0.5 ± 0.1	0.3	0.6 ± 0.1	0.4	0.3
AF0658		Flp pilus assembly protein TadC*	N	-				0.8 ± 0.1	0.6	0.6 ± 0.1	0.8 ± 0.1	1
AF0659	gspE-2	type II secretion system protein	N	-	1.26			2.1	1.7	1.8 ± 0.1	2.4	2.4
AF0660		Predicted pyrophosphatase*	R	-				2.5 ± 0.2	1.9 ± 0.1	2.3 ± 0.2	1.5 ± 0.2	1.5 ± 0.1
AF0661		heterodisulfide reductase, subunit E, putative	C	-	-1.09			**3.3**	**3.3**	**3.3**	2.9	**3 ± 0.2**
AF0662		heterodisulfide reductase, subunit A/methylviologen reducing hydrogenase, subunit delta	C	-				**3.4**	**3.4**	**3.4**	**3.2**	**3.2**
AF0663	hdrA-1	heterodisulfide reductase, subunit A	C	-	-1.11			**3.4**	**3.3**	**3.4**	**3**	**3**
AF0664		uncharacterized conserved protein*	S	+	**4.14**			0.1	0.5	0.1	0.9 ± 0.1	1.2 ± 0.1
AF0665		O-sialoglycoprotein endopeptidase, putative	T	+				0.3	0.4	0.2	0.3	0.3
AF0666		Predicted DNA-binding protein*	R	-				0.2	0.2	0.2	0.1	0.1
AF0667		uncharacterized conserved protein*	S	+				0.3	0.4	0.4 ± 0.1	0.5 ± 0.1	0.5 ± 0.1
AF0668		NifX family protein*	S	+				0.5 ± 0.1	0.6	0.7 ± 0.1	0.7 ± 0.1	0.8 ± 0.1
AF0669		4-oxalocrotonate tautomerase, putative	R	+				1.4 ± 0.1	1.3 ± 0.1	1.4 ± 0.1	1.2 ± 0.1	1.3 ± 0.1
AF0670	aroC	chorismate synthase	E	+				2.1	2.2	2.3	1.8 ± 0.1	1.9 ± 0.1
AF0671	acd-4	acyl-CoA dehydrogenase	I	-				0.9 ± 0.1	0.7	1.1 ± 0.1	0.8	0.9
AF0672	alkK-3	medium-chain acyl-CoA ligase	I	-				0.6	0.3	0.5	0.4	0.4
AF0673	merR	mercuric resistance operon regulatory protein	X	-				0.2	0.3	0.1	0.1	0.2
AF0674		Predicted transcriptional regulator*	K	+				1.6 ± 0.1	1.5 ± 0.1	1.5 ± 0.1	1.5 ± 0.1	1.7
AF0675	todF	2-hydroxy-6-oxohepta-2,4-dienoate hydrolase	R	+				0.4	0.5	0.4	0.3	0.4
AF0676	adk	adenylate kinase	F	-				2.5	2.5	2.5	2.2	1.7 ± 0.1
AF0677	acs-3	acetyl-CoA synthetase	I	-	**2.84**			0.5	0.6	0.5	1.4 ± 0.1	1.8 ± 0.3
AF0678		ISA1083-1 transposase	L	+				0.2	0.2	0.1	0.2	0.1
AF0679		ISA1083-1 transposase	L	+				0	0	0	0	0
AF0680	glnQ	glutamine ABC transporter, ATP-binding protein	E	-			1.31	0.6	0.7	0.5	0.5 ± 0.1	0.7
AF0681	sdhA	succinate dehydrogenase flavoprotein subunit	C	+				2.3 ± 0.2	1.6 ± 0.2	2.3 ± 0.2	**3.1**	**3**
AF0682	sdhB	succinate dehydrogenase iron-sulfur subunit	C	+	**2.03**			0.9 ± 0.1	0.8 ± 0.1	0.9 ± 0.1	1.7 ± 0.1	2.1
AF0683	sdhC	succinate dehydrogenase, subunit C	C	+	**1.88**			1.4 ± 0.1	1.2 ± 0.1	1.3 ± 0.2	2.3	2.8
AF0684	sdhD	succinate dehydrogenase, subunit D	C	+	**2.47**			0.9 ± 0.1	0.8 ± 0.1	0.8 ± 0.1	1.7 ± 0.1	2.6 ± 0.2
AF0685	fad-2	enoyl-CoA hydratase	I	+	**1.57**			0.1	0.2	0.2	0.3	0.3
AF0686		hypothetical protein	X	+				0.1	0.2	0.2	0.2	0.3
AF0687	fadD-3	acyl-CoA synthetase	I	-				0.4 ± 0.1	0.1	0.4 ± 0.1	0.8 ± 0.2	0.6 ± 0.1
AF0688		iron-sulfur cluster binding protein	C	+				0.4	0.4	0.5 ± 0.1	0.4	0.3
AF0689		uncharacterized conserved protein*	S	+	**1.60**			0.4	0.5	0.5	0.9	0.7
AF0690		Deacetylase, including yeast histone deacetylase and acetoin utilization protein*	R	-	**1.70**			0.1	0.2	0.1	0.2	0.2
AF0691		uncharacterized metal-binding protein*	R	+				1 ± 0.1	1 ± 0.1	1.1	1.2	1.1
AF0692		uncharacterized conserved protein*	S	+				0.1	0.1	0.1	0.1	0.1
AF0693m	boxA	DNA polymerase I	L	-				0.3	0.3	0.3	1 ± 0.3	0.4
AF0694		replication control protein A, putative	S	-				0.2	0.2	0.2	0.7 ± 0.3	0.3
AF0695	cdc6	cell division control protein 6	L	+				0.3	0.3	0.3	0.3	0.3
AF0696	minD-1	cell division inhibitor	N	+			**2.01**	0.9 ± 0.2	2.8	1.2 ± 0.2	2 ± 0.2	2.7 ± 0.2
AF0697		hypothetical protein	X	+			**2.42**	0.3	1.3 ± 0.1	0.4 ± 0.1	0.6 ± 0.2	1 ± 0.1
AF0698		Subunit of KEOPS complex (Cgi121BuD32KAE1)*	J	-				0.3 ± 0.1	0.5	0.4	0.3	0.4
AF0699		Single-stranded DNA-specific exonuclease RecJ*	L	-	**-1.60**			1.4 ± 0.1	1.3 ± 0.1	1.3 ± 0.1	0.7 ± 0.1	0.8
AF0700	lysC	aspartate kinase	E	-	-1.38			2.4	2.1	2.2	1.6 ± 0.1	1.5 ± 0.1
AF0701		uncharacterized conserved protein*	S	-				1.1 ± 0.1	1 ± 0.1	1.2 ± 0.1	1 ± 0.1	1.2
AF0702	thi1	ribulose-1,5-biphosphate synthetase	G	-			1.20	1.7 ± 0.1	2.2	1.8 ± 0.1	1.8 ± 0.1	2.1
AF0703		hypothetical protein	X	-	**1.64**			0	0	0	0.1	0.1
AF0704		Wybutosine (yW) biosynthesis enzyme, Trm5 methyltransferase*	J	+				0.2 ± 0.1	0.3	0.3	0.2	0.2
AF0705		Archaeal serine protease*	R	+			-1.30	1.3 ± 0.2	1.1 ± 0.1	1.4 ± 0.1	1.4 ± 0.2	0.9 ± 0.1
AF0706		Predicted membrane protein*	S	-				0.4 ± 0.1	0.3	0.5	0.3 ± 0.1	0.2
AF0707		undecaprenyl pyrophosphate synthase*	I	+			1.16	0.4	0.5	0.4	0.3	0.4
AF0708		tRNA modification protein, contains pre-PuA and PuA domains*	J	+				0.7	0.8	0.7	0.7	0.7
AF0709		uncharacterized conserved protein*	S	+				0.3	0.5	0.3	0.3 ± 0.1	0.3
AF0710	ppsA	phosphoenolpyruvate synthase	G	-		-1.20		2.5	2.7	2.2	2.1	2.1
AF0711	trx-1	thioredoxin	O	+		-1.27		0.2	0.3	0.2	0.2	0.2
AF0712		Predicted membrane protein*	S	+				0.4	0.3	0.4	0.3	0.3
AF0713	hisA-1	phosphoribosylformimino-5-aminoimidazole carboxamide ribotide isomerase	R	-				0.2	0.2	0.2	0.2	0.1
AF0714	mtd	F420-dependent methylenetetrahydromethanopterin dehydrogenase	C	+				**3.1**	**3.1**	**3**	**3**	2.9 ± 0.2
AF0715		potassium channel, putative	P	+				1.4 ± 0.1	1.1 ± 0.1	1.1 ± 0.1	1	0.9 ± 0.1
AF0716		K+ transport system, NAD-binding component fused to Ion channel*	P	+				1 ± 0.1	0.9 ± 0.1	0.8	0.7 ± 0.1	0.8 ± 0.1
AF0717		Kef-type K+ transport system, predicted NAD-binding component*	P	+				1 ± 0.2	0.9 ± 0.1	0.8 ± 0.1	0.6 ± 0.1	0.8 ± 0.1
AF0718		Metal-dependent hydrolase of the beta-lactamase superfamily II*	R	+				0.3 ± 0.1	0.2	0.2	0.2	0.2
AF0719		Metal-dependent hydrolase of the beta-lactamase superfamily II*	R	+				0.1	0.1	0.1	0.1	0.1
AF0720		Predicted ATPase of PP-loop superfamily*	R	-				1.3 ± 0.1	1.1	1.6 ± 0.1	1.2 ± 0.1	1.4 ± 0.1
AF0721		Sirohydrochlorin ferrochelatase*	P	-				1	0.8	1.2 ± 0.1	0.9 ± 0.1	0.9
AF0722	cbiE	cobalt-precorrin-6Y C(5)-methyltransferase	H	-				1.2 ± 0.1	0.9	1.4 ± 0.2	1.2	1.2
AF0723	cbiD	cobalt-precorrin-6A synthase	H	-				2	1.6 ± 0.1	2.3 ± 0.2	2.3	2.4
AF0724	cbiH	cobalamin biosynthesis precorrin-3 methylase	H	-		1.42		1.4 ± 0.1	1.1	1.8 ± 0.1	1.9 ± 0.1	1.9
AF0725	cbiG	cobalamin biosynthesis precorrin methylase	H	-		**1.53**		1.6 ± 0.1	1.4 ± 0.1	2.2 ± 0.2	2.3	2.6
AF0726	cbiF	cobalamin biosynthesis precorrin-3 methylase	H	-		**1.73**		1.2 ± 0.1	0.8	1.7 ± 0.2	1.8 ± 0.1	1.9 ± 0.1
AF0727	cbiL	cobalamin biosynthesis precorrin-2 methyltransferase	H	-		**1.88**		0.6 ± 0.1	0.4	0.9 ± 0.1	1 ± 0.1	1.2
AF0728	cbiM-1	cobalt transport protein CbiM	P	+				0.4 ± 0.1	0.3	0.7 ± 0.2	0.6 ± 0.2	1
AF0729	cbiN	cobalt transport protein CbiN	P	+				0.2 ± 0.1	0.1	0.4 ± 0.1	0.3 ± 0.1	0.7
AF0730	cbiQ-1	cobalt transport protein	P	+				0.3 ± 0.1	0.2	0.5 ± 0.2	0.5 ± 0.2	0.9 ± 0.1
AF0731	cbiO-1	cobalt transport ATP-binding protein	P	+				0.3 ± 0.1	0.2	0.5 ± 0.1	0.5 ± 0.1	0.7
AF0732	cbiT	cobalamin biosynthesis precorrin-8W decarboxylase	H	+				0.4	0.4	0.6 ± 0.1	0.5 ± 0.1	0.6

AF0733	thiL	thiamine monophosphate kinase	H	-				0.3	0.2	0.2	0.2	0.1
AF0734		ribosome biogenesis protein	J	-				0.1	0.1	0.1	0.1	0.1
AF0735		Single-stranded DNA-specific exonuclease RecJ*	L	+				1.6 ± 0.1	1.5	1.7 ± 0.2	1.5 ± 0.1	1.5 ± 0.1
AF0736		Predicted membrane protein*	S	+				0.5 ± 0.1	0.4 ± 0.1	0.5 ± 0.1	0.5 ± 0.1	0.4
AF0738		Predicted metal-binding protein*	R	+				2.1 ± 0.2	1.6	1.8 ± 0.2	2.3	2
AF0739		ribosomal protein S18 alanine acetyltransferase	R	-			**1.52**	0.4 ± 0.1	0.6 ± 0.1	0.3	0.5 ± 0.1	0.6
AF0740a		Predicted small methyltransferase*	S	+				0.6	0.5	0.5 ± 0.1	0.5	0.4
AF0741		uncharacterized conserved protein*	S	+				0.6 ± 0.1	0.5	0.6 ± 0.1	0.5 ± 0.1	0.4
AF0742		DNA primase, putative	L	+				0.6 ± 0.1	0.5	0.7	0.5 ± 0.1	0.4
AF0743		Transcriptional regulator, CopG/Arc/MetJ family (DNA-binding and a metal-binding domains)*	V	-			-1.36	0.4	0.3	0.4	0.3	0.2
AF0744		GTP-binding protein	R	-	**1.78**			0.8	1.3	0.8 ± 0.1	1.8 ± 0.1	1.8 ± 0.1
AF0745	pyrD	dihydroorotase dehydrogenase	F	+			**-1.94**	0.3	0.1	0.3	0.2	0.1
AF0746		tRNA-dihydrouridine synthase*	J	-				0.6 ± 0.1	0.4	0.8 ± 0.1	0.4 ± 0.1	0.2
AF0747	dapF	diaminopimelate epimerase	E	-	**-1.60**			1	0.9	0.9 ± 0.1	0.7	0.4
AF0748		NAD kinase*	H	+				1.8	1.7	1.8 ± 0.1	1.6 ± 0.1	1.5 ± 0.1
AF0749	orA	2-oxoacid ferredoxin oxidoreductase, subunit alpha	C	+				2.5	2.3	2.4	2.2	2.1
AF0750	orB	2-oxoacid ferredoxin oxidoreductase, subunit beta	C	+	-1.42			0.7	0.6	0.6	0.5	0.4
AF0751		Spou rRNA Methylase family enzyme*	R	+	**-1.61**			0.4	0.5	0.5	0.3	0.3
AF0752		uncharacterized conserved protein*	S	-	**-1.55**			0.3	0.3	0.4	0.2	0.2
AF0753		Predicted membrane-bound mannosyltransferase*	O	-			-1.46	0.2	0.1	0.2	0.2	0.1
AF0754		hypothetical protein	X	-	**2.97**			0.8 ± 0.1	0.9 ± 0.1	0.7	2.4	2.5
AF0755		heterodisulfide reductase, subunits E and D, putative	C	-	**4.69**			0.3 ± 0.1	0.4	0.6 ± 0.1	2.4	2.3
AF0756	ppx1	putative manganese-dependent inorganic pyrophosphatase	C	+	**-2.39**			**3.1**	**3.3**	**3.1**	1.4 ± 0.1	1.2
AF0757		transcription initiation factor IIE, subunit alpha, putative	K	+				0.9 ± 0.1	1.2 ± 0.1	1 ± 0.1	1 ± 0.1	1.8 ± 0.2
AF0758		uncharacterized conserved membrane protein*	S	-			**1.67**	0.4 ± 0.1	0.6	0.4	0.6 ± 0.1	0.8 ± 0.1
AF0759		uncharacterized conserved membrane protein*	S	-				2.2	2.7	2.3	2.7	**3**
AF0760		Protein comtaining Bacterial Ig-like domain and vWFA domain*	R	-	1.21			2 ± 0.2	2.2	2.2	2.6	2.7
AF0761		uncharacterized conserved protein*	S	-				0.5	0.5 ± 0.1	0.5	1 ± 0.2	1.7 ± 0.2
AF0762		uncharacterized conserved protein*	S	-	**2.43**			0.3 ± 0.1	0.3	0.3	0.7 ± 0.1	1 ± 0.1
AF0763		uncharacterized conserved protein*	S	-				0.6 ± 0.1	0.7 ± 0.1	0.6	1 ± 0.2	1.5 ± 0.1
AF0764	rpl7ae	50S ribosomal protein L7Ae	J	+				**3.2**	**3.3**	**3.2**	**3.1**	**3.1 ± 0.2**
AF0765	rps28e	30S ribosomal protein S28e	J	+				**3.4**	**3.5**	**3.4**	**3.4**	**3.4 ± 0.2**
AF0766	rpl24E	50S ribosomal protein L24E	J	+				**3**	**3.2**	2.9	2.8	2.9
AF0767	ndk	nucleoside diphosphate kinase	F	+				2.9	**3.1**	2.7	2.4	2.6
AF0768	infB	translation initiation factor IF-2	J	+	-1.29	-1.27		2.4	2.4	2.1	1.7 ± 0.1	1.7 ± 0.1
AF0769	trx-2	thioredoxin	O	+	**-2.03**			0.7 ± 0.1	0.6 ± 0.1	0.6 ± 0.1	0.3 ± 0.1	0.3
AF0770		signal-transducing histidine kinase	T	-				0.4	0.3	0.4	0.5	0.7 ± 0.1
AF0771		4-hydroxybenzoate polyprenyltransferase or related prenyltransferase*	H	+				0.1	0.1	0	0.1	0.1
AF0772		uncharacterized conserved protein*	S	-				0.1	0.1	0.1	0.1	0.2
AF0773		uncharacterized conserved protein*	S	-				0.3	0.2	0.3	0.3 ± 0.1	0.3 ± 0.1
AF0774		uncharacterized conserved protein*	S	-				1	0.7	1.1	1.1 ± 0.1	1.1 ± 0.1
AF0775		RecA-superfamily ATPase implicated in signal transduction*	T	-				1.6 ± 0.2	1.3 ± 0.1	1.7 ± 0.1	1.7 ± 0.1	1.7 ± 0.1
AF0776	tyrS	tyrosyl-tRNA synthetase	J	+		-1.26		1.9 ± 0.1	2.1	1.7	1.5 ± 0.1	1.4
AF0777	eif1A	translation initiation factor IF-1A	J	+				2 ± 0.2	2.5	2.1	1.8 ± 0.1	2.1
AF0778		uncharacterized conserved protein*	S	+				0.7 ± 0.1	0.8 ± 0.1	0.7 ± 0.1	0.6 ± 0.1	0.6
AF0779		RPA family protein, a subunit of RPA complex in P.uriosus*	R	-				1.1 ± 0.1	1.3 ± 0.1	1.1	1.2	1.3 ± 0.1
AF0780		replication factor A	L	-				1.5 ± 0.1	1.8 ± 0.1	1.6 ± 0.1	1.8 ± 0.1	1.7 ± 0.1
AF0781		Putative translation factor (SuA5)*	J	+				0.4	0.4	0.4	0.4	0.4
AF0782		Predicted cell division protein, SepF homolog*	D	+				0.8	0.7	0.9 ± 0.1	1 ± 0.1	1.1 ± 0.1
AF0783		C4-type Zn-finger protein*	R	+				0.9 ± 0.1	0.8	1.1	1	1.1
AF0784		hypothetical protein	X	-				0.2	0.2	0.3	0.1	0.1
AF0785		Predicted membrane protein*	S	-				0.1	0.1	0.1	0.1	0.1
AF0786	corA	magnesium and cobalt transporter	P	-				0.3	0.1	0.2	0.3	0.4
AF0787		Predicted cation transporter*	R	+				1	1	0.9 ± 0.1	0.9 ± 0.1	1.1
AF0788		Permease of the drug/metabolite transporter (DMT) superfamily*	G	+				0.9 ± 0.1	0.7	0.8 ± 0.1	0.8	0.6
AF0789		uncharacterized conserved protein*	S	+				0.5 ± 0.1	0.8	0.4	0.3	0.4
AF0790		SAM-dependent methyltransferase*	Q	-				0.2	0.2	0.2	0.2	0.1
AF0791		phosphate permease, putative	P	+			**-1.84**	0.2	0.1	0.2	0.2	0.1
AF0792		uncharacterized conserved protein*	S	-				0.3	0.3	0.4	0.3	0.2
AF0793		uncharacterized conserved protein*	S	+				1.2 ± 0.1	1.1	1 ± 0.1	0.7 ± 0.1	0.9 ± 0.1
AF0794		Phosphate uptake regulator*	P	-				0.9 ± 0.2	0.9 ± 0.1	0.9 ± 0.1	0.9 ± 0.1	0.7 ± 0.1
AF0795		RecA-superfamily ATPase implicated in signal transduction*	T	+				1.6 ± 0.3	1.5 ± 0.1	1.6 ± 0.2	1.7 ± 0.1	1.4 ± 0.2
AF0796		Predicted transcriptional regulator*	K	+				1.2 ± 0.2	1.4 ± 0.2	1.2 ± 0.1	1.2 ± 0.1	1.2 ± 0.2
AF0797	cofG	FO synthase subunit 1	H	+				1.3 ± 0.1	1.1 ± 0.1	1.3 ± 0.1	1.1	0.8
AF0798		Thiamine biosynthesis enzyme ThiH, FO synthase or related uncharacterized enzyme*	H	+				2.6	2.4	2.5	2.3	2
AF0799		Phosphoesterase*	R	-				0.9	0.9	0.9 ± 0.1	0.9	0.8
AF0800	lysA	diaminopimelate decarboxylase	E	-				1.7	2 ± 0.1	1.7 ± 0.1	1.7 ± 0.1	1.7
AF0801	rps15p	30S ribosomal protein S15P	J	-				1.6 ± 0.1	2 ± 0.2	1.5 ± 0.1	1.4 ± 0.1	1.4
AF0802		Predicted membrane protein*	S	+				0.3	0.4	0.2	0.2	0.2
AF0804		uncharacterized conserved small protein*	S	+				0.1	0.1	0.1	0.1	0.1
AF0805		Predicted transcriptional regulator, C-terminal HTH-like domain*	K	-				0.4 ± 0.1	1.3 ± 0.1	0.1	0.3	0.3
AF0806	lctP	L-lactate permease	C	+		**-5.81**		2.4 ± 0.2	**3**	0.7 ± 0.1	0.4 ± 0.1	0.2
AF0807	lldD	L-lactate dehydrogenase, cytochrome-type	H	-		**-4.57**		2.1 ± 0.3	**3.1**	0.8 ± 0.1	0.5 ± 0.1	0.4
AF0808	glcD	glycolate oxidase subunit	C	+		**-9.04**		2.4 ± 0.3	**3.1**	0.6 ± 0.1	0.1	0.1
AF0809		heterodisulfide reductase, subunit D, putative	C	+	**-5.69**	**-4.07**		**3.1 ± 0.2**	**3.4**	1.3 ± 0.2	0.5	0.4
AF0810		hypothetical protein	X	+		**-5.86**		2.4 ± 0.3	**3.1**	0.7 ± 0.1	0.3	0.3
AF0811		uncharacterized conserved protein containing ferredoxin-like domain*	C	+		**-5.75**		2.3 ± 0.4	**3.2**	0.7 ± 0.1	0.3	0.3
AF0812		hypothetical protein	X	+	**-2.58**	**-2.07**		0.6 ± 0.1	0.8	0.5	0.3	0.2
AF0813	serA	D-3-phosphoglycerate dehydrogenase	H	-				2.1	2.3	2.1	1.7 ± 0.1	1.8 ± 0.1
AF0814		Predicted nucleotide kinase*	F	+				0.8	1.3 ± 0.1	0.9 ± 0.1	0.9	1 ± 0.1
AF0815	trm1	N(2),N(2)-dimethylguanosine tRNA methyltransferase	J	+				0.3	0.6	0.3	0.3	0.4
AF0816		vtpJ-therm, putative	X	-	**4.27**			0.3 ± 0.1	0.2	0.3	1.4 ± 0.1	1.2 ± 0.1
AF0817		Secreted protein containing C-terminal beta-propeller domain distantly related to WD-40 repeats*	R	-		-1.42		1.5 ± 0.2	1.6 ± 0.1	1.2 ± 0.1	1 ± 0.1	0.9 ± 0.1
AF0818	acyP	acylphosphatase	C	+				0.5	0.4	0.3	0.4 ± 0.1	0.2
AF0819	hisF	imidazole glycerol phosphate synthase subunit HisF	E	+				1.9 ± 0.1	1.9 ± 0.1	1.7 ± 0.1	1.9 ± 0.2	1.3 ± 0.1
AF0820		Predicted pyrophosphatase*	R	+				2 ± 0.1	2.2	1.8 ± 0.2	2 ± 0.2	1.5 ± 0.1
AF0821		nicotinate phosphoribosyltransferase	H	+				1.8	2 ± 0.1	1.6 ± 0.1	1.6 ± 0.1	1.4
AF0822	braF-2	branched-chain amino acid ABC transporter, ATP-binding protein	E	-			**2.51**	0.1	0.3	0.1	0.2	0.2
AF0823	braG-2	branched-chain amino acid ABC transporter, ATP-binding protein	E	-			**2.31**	0.2	0.9	0.2	0.7 ± 0.1	0.8
AF0824	braE-2	branched-chain amino acid ABC transporter, permease protein	E	-			**2.36**	0.5	1.9 ± 0.1	0.5	1.5 ± 0.2	1.9 ± 0.1
AF0825	braD-2	branched-chain amino acid ABC transporter, permease protein	E	-				0.4 ± 0.1	1.3 ± 0.1	0.4	1.3 ± 0.2	1.2 ± 0.1
AF0826		Nucleotide-binding protein, uspA family*	T	-				0.3	0.6	0.2	0.3	0.3
AF0827	braC-2	branched-chain amino acid ABC transporter, periplasmic binding protein	E	-				0.8 ± 0.1	2 ± 0.1	0.7 ± 0.1	1.1 ± 0.1	1.1
AF0828		uncharacterized conserved protein*	S	+				0.1	0	0.1	0	0.1
AF0829		uncharacterized conserved protein*	S	-				0.2 ± 0.1	0.1	0.4 ± 0.1	0.1	0.2
AF0830		Conserved protein/domain typically associated with flavoprotein oxygenase, DIM6/NTAB family*	R	-				2.1	1.6	2.6	1.8 ± 0.1	1.9 ± 0.1
AF0831	rr2	rubrerythrin	C	-				**3.2**	2.9	**3.4**	**3.2**	**3.3**
AF0832	rr1	rubrerythrin	C	-				2.8 ± 0.2	2.4	**3.1**	2.9 ± 0.2	2.8
AF0833	dfx	desulfoferrodoxin	C	-				**3.3**	2.8	**3.4**	**3.3**	**3.2**
AF0834		ferritin, putative	P	-				2.2 ± 0.2	1.3 ± 0.1	2.7	2 ± 0.1	1.8 ± 0.1
AF0835		hypothetical protein	X	-				0	0	0	0	0
AF0836		hypothetical protein	X	+			1.11	0	0	0	0	0
AF0837	pelA	cell division protein pelota	J	+				2.2	2.2	2.1	2	1.9

AF0838	trkA	potassium transporter peripheral membrane component	P	+				2 ± 0.1	1.7 ± 0.2	1.9 ± 0.1	1.7 ± 0.2	1.6 ± 0.1
AF0839	trkH	TRK potassium uptake system protein	P	+				1.1 ± 0.1	0.9	0.9 ± 0.1	0.7	0.8
AF0840	fadD-4	long-chain-fatty-acid--CoA ligase	I	+	**3.51**			0.1	0.9	0.1	1.2 ± 0.1	1.5
AF0841	purA	adenylosuccinate synthetase	F	-		-1.30		2.1	2.5	2 ± 0.1	1.6 ± 0.1	1.7 ± 0.1
AF0842		hypothetical protein	X	-				0.8 ± 0.1	0.4	1.3 ± 0.4	0.1	0.1
AF0843		uncharacterized conserved protein*	S	+				0.3	0.3	0.2	0.2	0.2
AF0844		hypothetical protein	X	-			**-1.58**	0.5 ± 0.1	0.2	0.4	0.4	0.2
AF0845	acd-5	acyl-CoA dehydrogenase	I	-	1.47			0.1	0.1	0.1	0.2	0.2
AF0846	nhe2	Na+/H+ antiporter	P	-	**-5.15**			0.3	0.2	0.3	0	0
AF0847	guaB-1	inosine monophosphate dehydrogenase	R	+			-1.15	2.6	2.1	2.6	2.3	2.2
AF0848		CBS domain*	R	+			-1.23	1.8 ± 0.1	1.3 ± 0.1	1.8 ± 0.1	1.5 ± 0.1	1.4 ± 0.1
AF0849		ATP-dependent RNA ligase*	L	+				1.2 ± 0.3	0.7 ± 0.1	1.2 ± 0.2	0.8 ± 0.2	1
AF0850		Predicted butyrate kinase*	C	+				1.6 ± 0.2	1.4 ± 0.1	1.5 ± 0.2	1.2 ± 0.2	1.5
AF0851		Predicted sugar kinase*	G	-				0.7	0.8	0.7	0.6	0.6
AF0852	glyA	serine hydroxymethyltransferase	E	+				1 ± 0.1	0.8	0.9	0.7	0.5
AF0853		proliferating-cell nucleolar antigen P120, putative	J	+				1.9 ± 0.1	2.1	2	1.7 ± 0.1	1.7 ± 0.1
AF0854		Ribosome biogenesis protein, NOL1/NOP2/fmu family*	J	+				1.1 ± 0.2	0.9 ± 0.1	1.1 ± 0.1	0.7 ± 0.1	0.6
AF0855	mdhA	L-malate dehydrogenase, NAD+-dependent	C	+				1.9 ± 0.1	2	2	1.9 ± 0.1	2
AF0856		Periplasmic serine protease (ClpP class)*	O	+				2.7	2.2	2.7	3	2.7
AF0859		uncharacterized conserved protein*	S	-				0.6	0.5	0.6	0.5	0.7
AF0860		NMD protein affecting ribosome stability and mRNA decay*	J	-			-1.31	0.2	0.1	0.2	0.2	0.2
AF0861	hps-1	bifunctional hexulose-6-phosphate synthase/ribonuclease regulator	G	+			-1.21	2.5	1.8 ± 0.2	2.4	2.4	2.1
AF0862		RNA 3'-P ligase, RtcB family protein*	J	-				1.6 ± 0.1	1.6 ± 0.1	1.6 ± 0.1	1.8 ± 0.1	1.7 ± 0.1
AF0863		uncharacterized conserved protein*	S	-	**2.10**			1.1 ± 0.1	1.1	1.1	2.2 ± 0.2	2.5 ± 0.2
AF0864		Predicted membrane protein, DuF2068*	S	-	**2.25**			0.7 ± 0.1	0.9 ± 0.1	0.7	1.8 ± 0.1	1.9 ± 0.1
AF0865	est-1	carboxylesterase	R	-	**2.82**			0.5 ± 0.1	0.7	0.4	1.3 ± 0.1	1.7 ± 0.2
AF0866	glpK	glycerol kinase	C	-	1.34			1 ± 0.1	0.9	1	1.3 ± 0.1	1.3 ± 0.1
AF0867		reductase, putative	C	-	1.34			1.8 ± 0.1	1.7 ± 0.1	1.9 ± 0.1	2.4	2.5
AF0868		alkyldihydroxyacetonephosphate synthase	C	-	1.36			1.6 ± 0.1	1.5 ± 0.1	1.7 ± 0.1	2.1	2.3
AF0869		uncharacterized beta/alpha-propeller protein, likely forms oligomers*	S	-				1.9 ± 0.1	1.9 ± 0.2	2.1	2.3	2.9
AF0870		uncharacterized conserved protein*	S	-				1.2 ± 0.1	0.6 ± 0.1	1.2 ± 0.1	1.1 ± 0.1	1.3 ± 0.1
AF0871	gpsA	glycerol-3-phosphate dehydrogenase (NAD(P)+)	C	-				1.2 ± 0.1	0.7	1.2 ± 0.1	1.1 ± 0.1	1.2
AF0872		uncharacterized protein containing a Zn-ribbon*	S	+				0.7 ± 0.2	0.4 ± 0.1	0.7 ± 0.1	0.4 ± 0.1	0.4
AF0873	purF	amidophosphoribosyltransferase	F	-				2.6 ± 0.2	2.4 ± 0.2	2.7	2.3 ± 0.2	2.1
AF0874	rpl37e	50S ribosomal protein L37e	J	-				2.9	2.9	3	2.6	2.8
AF0875		small nuclear ribonucleoprotein	K	-				2	2.2	2 ± 0.1	1.7 ± 0.1	1.8 ± 0.1
AF0876	nt5	5'-nucleotidase	F	+				0.5	0.6	0.5 ± 0.1	0.5	0.7 ± 0.1
AF0877		hypothetical protein	X	+	**2.43**			0.4	1 ± 0.1	0.3	1.4 ± 0.2	1.5 ± 0.2
AF0878		uncharacterized Rossmann fold enzyme*	R	-				0.3 ± 0.1	0.3	0.2	0.2	0.2
AF0879		tRNA S(4)u 4-thiouridine synthase*	J	-				0.7 ± 0.1	0.7 ± 0.1	0.6	0.6	0.6
AF0880	rd-1	rubredoxin	C	+			**1.87**	0.1	0.3	0.1	0.1	0.2
AF0882	asnA	glutamyl-tRNA(Gln) amidotransferase subunit D	E	-				1.5	1.8 ± 0.1	1.3 ± 0.1	1.3	1.5 ± 0.1
AF0883	argH	argininosuccinate lyase	E	-	-1.34			1.3 ± 0.1	1.1	1.1	0.9	0.8
AF0884		uncharacterized conserved protein*	S	+			1.45	0.1	0.1	0.1	0.1	0.1
AF0885	hpaA-2	4-hydroxyphenylacetate-3-hydroxylase	Q	-	**1.54**			0.8 ± 0.1	0.6	0.9 ± 0.1	1.3 ± 0.1	1.3
AF0886	ahcY-1	S-adenosyl-L-homocysteine hydrolase	H	-				0.9 ± 0.1	0.9 ± 0.1	1	0.9 ± 0.1	0.9
AF0887	rbsA-1	ribose ABC transporter, ATP-binding protein	R	+				1.7 ± 0.1	1.8 ± 0.1	1.7 ± 0.2	1.6 ± 0.1	1.9 ± 0.1
AF0888	rbsC-1	ribose ABC transporter, permease protein	R	+				1.1 ± 0.1	0.9 ± 0.1	1 ± 0.1	1 ± 0.1	1.2 ± 0.1
AF0889	rbsC-2	ribose ABC transporter, permease protein	R	+				1.2 ± 0.1	1.1 ± 0.2	1.2 ± 0.2	1.1 ± 0.1	1.4 ± 0.1
AF0890		uncharacterized ABC-type transport system, periplasmic component/surface lipoprotein*	R	+				3.2	3.2	3.1	3	3
AF0891		uncharacterized conserved protein*	S	-	**-1.73**			0.4	0.3	0.4	0.2	0.2
AF0892		Predicted dinucleotide-binding enzyme*	R	+				0.2	0.3	0.2	0.3	0.3
AF0893		signal-transducing histidine kinase	T	-	**4.11**			0.1	0.2	0.1	0.4	0.6
AF0894	argS	arginyl-tRNA synthetase	J	+	-1.35			2 ± 0.1	1.8	1.8 ± 0.1	1.5	1.1 ± 0.1
AF0895		transposase IS240-A	L	+				0	0	0	0	0
AF0896		uncharacterized conserved protein*	S	+				0.2	0.1	0.2	0.2	0.2
AF0897		uncharacterized conserved protein*	S	+				0.1	0.1	0.1	0.1	0.1
AF0898		hypothetical protein	X	-			1.33	0.1	0.1	0.1	0.1	0.1
AF0899		Predicted metal-dependent hydrolase (urease superfamily)*	R	+				1.6	1.7 ± 0.1	1.6 ± 0.1	1.4 ± 0.1	1.1 ± 0.1
AF0900	endA	tRNA splicing endonuclease	J	+				0.7	0.7	0.7	0.6	0.5
AF0901		NTPase	F	+				0.8	0.7	0.8 ± 0.1	0.7 ± 0.1	0.5 ± 0.1
AF0902		DNA repair photolyase*	L	+				0.8 ± 0.2	0.9 ± 0.1	1 ± 0.1	0.8 ± 0.1	0.8
AF0903		Predicted transcriptional regulator*	K	+	-1.24			2.2	2.2	2.1	1.9 ± 0.1	1.6 ± 0.1
AF0904	dapE-2	succinyl-diaminopimelate desuccinylase	E	+	-1.27			2.3	2.2	2.2	1.9 ± 0.1	1.5
AF0905		Ribonuclease M5 (contains TOPRIM domain)*	L	-				0.2	0.1	0.2	0.1	0.1
AF0906	hyuA	hydantoin utilization protein A	E	-				0.4	0.2	0.4	0.5 ± 0.1	0.5
AF0907		Permease of the major facilitator superfamily*	G	-				0.3	0.2	0.3	0.2	0.2
AF0908		3-hydroxy-3-methylglutaryl CoA synthase family enzyme*	R	+	**3.98**			0.3	0.3	0.3	1.1 ± 0.1	1.4 ± 0.1
AF0909	dapB	dihydrodipicolinate reductase	E	-	-1.20			1.3	1.4 ± 0.1	1.3 ± 0.1	1.1	1 ± 0.1
AF0910	dapA	dihydrodipicolinate synthase	E	-				2.4	2.7	2.3	2.3	2.2
AF0911	rps17E	SSu ribosomal protein S17E	J	-				2	2.4	1.9 ± 0.1	2 ± 0.1	2 ± 0.1
AF0912		uncharacterized conserved protein*	S	-				0.6 ± 0.1	0.6 ± 0.1	0.7 ± 0.1	1.1 ± 0.5	1.8
AF0913		uncharacterized conserved protein*	S	-				0.1	0.1	0.1	0.1	0.1
AF0914		translation initiation factor Sui1	J	+				0.9 ± 0.1	1.3 ± 0.1	0.8 ± 0.1	0.8 ± 0.1	1.1 ± 0.1
AF0915		uncharacterized conserved protein*	S	-	-1.49			0.4	0.4	0.4 ± 0.1	0.2	0.2
AF0916	glyS	glycyl-tRNA synthetase	J	+				2.9	3.1	2.9	2.8	2.6
AF0917		LPPG:FO 2-phospho-L-lactate transferase	H	+				2.5	2.6	2.3	2.3 ± 0.2	2.1
AF0918	act-2	pyruvate formate-lyase activating enzyme	R	+				0.9 ± 0.1	0.8 ± 0.1	0.7 ± 0.1	0.6 ± 0.1	0.5
AF0919		Phosphoesterase*	R	+				1.1 ± 0.1	1.3 ± 0.1	1 ± 0.1	0.9	0.8
AF0920	aspC	aspartyl-tRNA synthetase	J	+				2.2	2.1	2	2.1	1.6 ± 0.1
AF0921		Imidazolonepropionase or related amidohydrolase*	Q	-				0.1	0.1	0.1	0.1	0
AF0922		Glutamate dehydrogenase/leucine dehydrogenase*	E	-				0.1	0.1	0.1	0.1	0.1
AF0923	fdx-5	ferredoxin	C	+				0	0	0.1	0.1	0.1
AF0924		hypothetical protein	X	-				0.1	0.1	0.1	0.1	0.1
AF0925		Zn-dependent protease with chaperone function*	O	-				0.1	0.1	0.1	0.1	0.1
AF0926		HGG motif-containing thioesterase, possibly involved in aromatic compounds catabolism*	Q	+				0.1	0.1	0.1	0.1	0.1
AF0927		Phosphopantothenate synthetase*	H	-	**-1.58**			0.6	0.7	0.6	0.4	0.4
AF0928		HAD superfamily hydrolase*	R	-	**-1.67**			0.4	0.3	0.4	0.2	0.2
AF0929		Orotidine-5'-phosphate decarboxylase*	F	-	**-1.59**			0.3	0.3	0.3	0.2	0.2
AF0930	moeA-2	molybdenum cofactor biosynthesis protein	H	+				2.6	2.8	2.9	2.7	2.8 ± 0.2
AF0931	moeA-1	molybdenum cofactor biosynthesis protein	H	+				2.7	2.9	2.9	2.7	2.8
AF0932		Acyl-CoA synthetase, ATP-grasp containing subunit*	C	+				0.8	1 ± 0.1	0.8 ± 0.1	0.8 ± 0.1	0.8
AF0933	ilvE	branched-chain amino acid aminotransferase	E	+				2.8	2.7	2.8	2.8	2.6
AF0934		ACT-domain-containing protein, predicted allosteric regulator of homoserine dehydrogenase*	E	+				3	3	3.2	3.2	3.2
AF0935	hom	homoserine dehydrogenase	E	+				2.7	2.9	3	3	3.1
AF0936		Peptidase A24A, prepilin type IV*	N	+				2.4	2.4	2.7	2.7	2.6
AF0937	tuf	elongation factor 1-alpha	J	+				3.6	3.6	3.7	3.7	3.6
AF0938	rps10p	30S ribosomal protein S10P	J	+				2.4 ± 0.2	2.7 ± 0.2	2.3	2.2 ± 0.2	2
AF0939		ribonuclease Z	R	+				1.4 ± 0.2	1.4 ± 0.2	1.5 ± 0.1	1.2 ± 0.2	1 ± 0.1
AF0940	top6A	DNA topoisomerase VI subunit A	L	-				0.6 ± 0.1	0.5	0.6	0.7 ± 0.1	0.5
AF0941		uncharacterized conserved protein*	S	+				0.5	0.5	0.5 ± 0.1	0.5 ± 0.1	0.4
AF0942	surE	stationary phase survival protein SurE	R	+			1.45	1 ± 0.1	1.7 ± 0.1	1.1 ± 0.1	1.1 ± 0.1	1.5 ± 0.1
AF0943	rpi	ribose 5-phosphate isomerase	G	+				1.3 ± 0.1	1.8	1.4 ± 0.1	1.1 ± 0.1	1.6 ± 0.1

AF0944		uncharacterized conserved protein*	S	-			**-1.57**	1.2 ± 0.1	0.7	1 ± 0.1	0.9 ± 0.1	0.6
AF0945		uncharacterized conserved protein*	S	-				3	3	2.9	2.9	2.8
AF0946		uncharacterized conserved protein*	S	-				3.3 ± 0.2	3.4	3.3 ± 0.2	3.3	3.4
AF0947		HEPN domain containing protein*	V	-				0.4	0.4	0.4 ± 0.1	0.4	0.5
AF0948		Minimal nucleotidyltransferase*	V	-				0.3	0.3	0.3	0.3	0.3
AF0949	glnA	glutamine synthetase	E	-				1 ± 0.2	1 ± 0.1	0.8 ± 0.1	0.9 ± 0.1	0.6
AF0950	cooF	carbon monoxide dehydrogenase, iron sulfur subunit	C	+			1.24	1.3 ± 0.1	1.8 ± 0.1	1.4 ± 0.2	1.6 ± 0.2	1.8
AF0951	noxA-4	NADH oxidase	R	+	1.20			1.8 ± 0.1	2	1.9 ± 0.1	2.3	2.4
AF0952		Glutamate synthase domain 1*	E	+				2.9	2.7	2.8	3.1	3
AF0953	gltB	glutamate synthase	E	+				3	2.8	2.9	3.2	3.2
AF0954		Glutamate synthase domain 3*	E	+				2.5	2.3	2.3	2.7 ± 0.2	2.5
AF0955		TPR repeats containing protein*	R	-			1.22	1.8 ± 0.1	2.5	1.9	1.8 ± 0.1	2.1
AF0956		uncharacterized conserved protein*	S	-				0.7	0.9 ± 0.1	0.7	0.6	0.6
AF0957	leuA-1	2-isopropylmalate synthase	E	+				1.8 ± 0.1	2	1.7 ± 0.1	1.6	1.3 ± 0.1
AF0958	braG-3	branched-chain amino acid ABC transporter, ATP-binding protein	E	-				0.1	0.1	0.1	0.1	0.1
AF0959	braF-3	branched-chain amino acid ABC transporter, ATP-binding protein	E	-	**1.83**			0.1	0.1	0.1	0.2	0.2
AF0960	braE-3	branched-chain amino acid ABC transporter, permease protein	E	-	**3.07**			0.1	0.1	0.1	0.2	0.3
AF0961	braD-3	branched-chain amino acid ABC transporter, permease protein	E	-	**3.97**			0.2	0.1	0.2	0.5 ± 0.1	1 ± 0.1
AF0962	braC-3	branched-chain amino acid ABC transporter, periplasmic binding protein	E	-	**3.35**			0.5	0.5	0.8	1.9 ± 0.1	2.3
AF0963	fad-3	enoyl-CoA hydratase	I	-	**2.00**	**2.04**		1.2	1.1 ± 0.1	1.7 ± 0.1	2.7	2.8 ± 0.2
AF0964	acd-6	acyl-CoA dehydrogenase	I	-	**1.57**	**1.60**		1.9 ± 0.1	1.6 ± 0.2	2.3	3.1	3.2 ± 0.2
AF0965		Predicted nucleic-acid-binding protein containing a Zn-ribbon*	R	-	**1.92**	**2.20**		1.5 ± 0.1	1 ± 0.1	2.2	3.2	3.1
AF0966		Predicted nucleic-acid-binding protein containing a Zn-ribbon*	R	-	**1.73**	**1.85**		1.7 ± 0.1	1.4 ± 0.1	2.4	3.2	3.3
AF0967	acaB-9	acetyl-CoA acetyltransferase	I	-		1.21		3	2.6	3.3	3.5	3.5
AF0968	acaB-10	acetyl-CoA acetyltransferase	I	-	**1.60**	**1.73**		1.6 ± 0.2	1.3 ± 0.1	2.2	3 ± 0.2	2.7 ± 0.2
AF0969	putP-2	proline permease	R	-	**9.47**			0.2	0.3	0.2	2.3 ± 0.2	2.8
AF0970		hypothetical protein	X	-	**22.85**			0.1	0.1	0.1	1.9 ± 0.3	2.3 ± 0.2
AF0971		CBS domain*	R	+	**4.62**			0.3	0.5 ± 0.1	0.3	1.7 ± 0.1	1.7 ± 0.1
AF0972	dnaQ	DNA polymerase III, subunit epsilon	L	+	**3.63**			0.1	0.1	0	0.2	0.2
AF0973	baiF-1	bile acid-inducible operon protein F	C	+	**3.74**			0.5 ± 0.1	1.3 ± 0.1	0.6 ± 0.1	3	3.2
AF0974	baiF-2	bile acid-inducible operon protein F	C	+	**2.15**			1 ± 0.1	2.1	1.2 ± 0.1	3.3	3.2 ± 0.2
AF0975	acs-4	acetyl-CoA synthetase	I	+	**3.28**			0.7 ± 0.1	1.4	0.8	3.3	3.3
AF0976	acs-5	acetyl-CoA synthetase	I	+	**2.46**			1.1 ± 0.1	1.9 ± 0.1	1.1	3.4	3.4
AF0977	amt-1	ammonium transporter	P	+				0.3	0.5 ± 0.1	0.2	0.2	0.2
AF0978	glnB-1	nitrogen regulatory protein P-II	E	+				0.7	0.9 ± 0.1	0.7 ± 0.1	0.6 ± 0.1	0.6 ± 0.1
AF0979	proW-1	osmoprotection protein	E	-				0.2	0.1	0.2	0.2	0.2
AF0980	proW-2	osmoprotection protein	E	-	**1.67**			0.5	0.6	0.6	0.9 ± 0.1	1
AF0981	proV	osmoprotection protein	E	-	**2.45**			0.4	0.4	0.4	1.1 ± 0.1	1 ± 0.1
AF0982	proX	osmoprotection protein	M	-	**3.38**			0.4	0.6	0.5	1.7 ± 0.1	1.8 ± 0.1
AF0983		ABC-type transport system involved in cytochrome c biogenesis, permease component*	O	-				0.2	0.1	0.2	0.1	0.1
AF0984		ABC transporter, ATP-binding protein	V	-				1 ± 0.1	0.8	1 ± 0.1	0.9 ± 0.1	1
AF0985	hisB	imidazoleglycerol-phosphate dehydrogenase/histidinol-phosphatase	E	-				1.4 ± 0.1	1.5 ± 0.1	1.4 ± 0.1	1.3 ± 0.1	1.8 ± 0.1
AF0986	hisA-2	phosphoribosylformimino-5-aminoimidazole carboxamide ribotide isomerase	E	-				0.6 ± 0.1	0.7	0.5 ± 0.1	0.4 ± 0.1	0.6
AF0987		Nucleotide-binding protein, uspA family*	T	-				0.1	0.2	0.1	0.2	0.2
AF0988	bcsp31-3	immunogenic protein	R	+				2.6	2.2	2.6	2.6	3.1
AF0989		TRAP-type uncharacterized transport system, fused permease component*	R	+				0.7 ± 0.1	0.2	0.6 ± 0.1	0.6 ± 0.1	0.5
AF0990	caiB-2	L-carnitine dehydratase	C	-	**2.36**			0.4	0.9 ± 0.1	0.4	1.4 ± 0.2	1.3 ± 0.1
AF0991	gcdH	glutaryl-CoA dehydrogenase	I	-	**1.77**			1.1 ± 0.1	1.8 ± 0.2	1.1 ± 0.1	2.7 ± 0.2	2.4
AF0992		uncharacterized conserved protein*	S	+				0.4	0.5	0.4	0.3	0.3
AF0993	radA	DNA repair and recombination protein RadA	L	-				1.1 ± 0.1	1.2 ± 0.1	1.2 ± 0.1	1.1 ± 0.1	1.3 ± 0.1
AF0994		Predicted HD superfamily hydrolase*	R	+			-1.40	0.3	0.2	0.3	0.2	0.2
AF0995		Flp pilus assembly protein TadC*	N	-				1.5 ± 0.1	0.8	1.3 ± 0.1	1.4 ± 0.1	1.3 ± 0.1
AF0996	gspE-3	type II secretion system protein	N	-				2.6	1.5 ± 0.1	2.4	2.6	2.4
AF0997	trzA-2	N-ethylammeline chlorohydrolase	F	-		-1.44		0.9 ± 0.1	1	0.8	0.6	0.6
AF0998		Predicted transcriptional regulator*	K	+				0.4	0.4	0.3	0.4	0.4
AF0999		Predicted membrane-associated trancriptional regulator*	K	-				0.7 ± 0.1	0.9	0.8	0.5	0.6
AF1000	nadE	NAD synthetase	H	-				0.8 ± 0.1	1	0.9 ± 0.1	0.7 ± 0.1	0.7
AF1001		hypothetical protein	X	-				0.2	0.3	0.2	0.4 ± 0.1	0.7 ± 0.1
AF1002		Phosphohistidine phosphatase SixA*	T	-				0.1	0.2	0.1	0.2	0.5 ± 0.1
AF1003		uncharacterized conserved protein*	S	+				0.4	0.5	0.4	0.4	0.3
AF1004		S-layer domain*	M	-			-1.23	0.2	0.2	0.2	0.2	0.2
AF1005		ABC transporter, ATP-binding protein, putative	V	-				0.1	0.1	0.1	0.1	0.1
AF1006		ABC transporter, ATP-binding protein	V	-				0.1	0.1	0.1	0.1	0.1
AF1007		Predicted ATP-dependent endonuclease of the OLD family*	L	+	**-2.44**	**-2.27**		0.2	0.2	0.1	0.1	0.1
AF1008		hypothetical protein	X	+				0	0	0	0	0
AF1009		Sugar-specific transcriptional regulator TrmB*	K	+			**1.55**	0.1	0.2	0.1	0.1	0.1
AF1010	fdx-6	ferredoxin	C	+		-1.23		0.1	0.1	0.1	0.1	0.1
AF1011		Membrane associated serine protease*	E	+				0.8 ± 0.1	0.5 ± 0.1	0.8 ± 0.1	0.7 ± 0.2	0.8 ± 0.1
AF1012		MiaB family, Radical SAM enzyme*	R	+				0.5 ± 0.1	0.3 ± 0.1	0.6 ± 0.1	0.4 ± 0.1	0.5 ± 0.1
AF1013		hypothetical protein	X	-				0.8 ± 0.1	0.3	0.8 ± 0.1	0.8 ± 0.1	0.7 ± 0.1
AF1014	ilvD	dihydroxy-acid dehydratase	E	-				1.7 ± 0.1	1.2	1.8 ± 0.1	1.9 ± 0.1	2 ± 0.1
AF1015		Predicted permease*	R	+	**1.84**			0.1	0.1	0.1	0.1	0.2
AF1016		uncharacterized conserved protein*	S	+				0.1	0.1	0.2	0.2	0.2
AF1017		ABC-type antimicrobial peptide transport system, permease component*	V	-				0.8 ± 0.1	0.8 ± 0.1	0.8 ± 0.1	0.6 ± 0.1	0.7 ± 0.1
AF1018		ABC transporter, ATP-binding protein	V	-				2 ± 0.1	2.2	2 ± 0.2	1.8 ± 0.1	2.3
AF1019		S-layer domain*	M	-				2.2	1.8 ± 0.1	2.2	2	1.9 ± 0.1
AF1020		Predicted regulator of amino acid metabolism, contains ACT domain*	R	-				0.2	0.1	0.3	0.2	0.2
AF1021		ABC transporter, ATP-binding protein	V	+				0.6	0.6	0.6	0.6 ± 0.1	0.7
AF1023	chlP-2	bacteriochlorophyll synthase, 43 kDa subunit	C	-				0.1	0.1	0.1	0.1	0.1
AF1024	top-RG	reverse gyrase	L	+				1.4 ± 0.2	1.7 ± 0.1	1.4 ± 0.1	1.3 ± 0.1	1.5
AF1025	hbd-4	3-hydroxyacyl-CoA dehydrogenase	I	-				0.7 ± 0.1	1.2 ± 0.2	0.5 ± 0.1	0.8 ± 0.2	1 ± 0.1
AF1026	acd-7	acyl-CoA dehydrogenase	I	+				0.3	0.8	0.3 ± 0.1	0.7 ± 0.1	0.5
AF1027	hpaA-3	4-hydroxyphenylacetate-3-hydroxylase	Q	-				0.2	0.5	0.2	0.5 ± 0.1	0.5
AF1028	fadA-1	acetyl-CoA acetyltransferase	I	-				0.2	0.4	0.2	0.5 ± 0.1	0.5
AF1029	fadD-5	long-chain-fatty-acid--CoA ligase	I	-	1.46			0.5 ± 0.1	0.6 ± 0.1	0.5 ± 0.1	1 ± 0.2	0.8
AF1030		HerA helicase*	L	+				2.5	2.7	2.7	2.8	3
AF1031	rad32	DNA repair protein RAD32	L	+				2	2	2.2	2.1	2.3 ± 0.2
AF1032		chromosome segregation protein	L	+				1.2 ± 0.1	1.1 ± 0.1	1.2 ± 0.2	1.1 ± 0.1	1.3 ± 0.1
AF1033		NurA 5'-3' nuclease*	L	+				0.6 ± 0.1	0.5 ± 0.1	0.5 ± 0.1	0.5 ± 0.1	0.6 ± 0.1
AF1034	tlpC-1	methyl-accepting chemotaxis protein	T	-				1 ± 0.1	0.3	0.9 ± 0.1	1.8 ± 0.7	2.4 ± 0.2
AF1035		chemotaxis histidine kinase, putative	T	-				0.1	0	0.1	0.1	0.1
AF1036		chemotaxis histidine kinase, putative	T	+				0.1	0	0.1	0.1	0.1
AF1037	cheR	chemotaxis protein methyltransferase	N	-				0.2	0.1	0.2	0.2	0.3
AF1038		Chemotaxis protein; stimulates methylation of MCP protein*	N	-				0.4	0.1	0.4	0.6 ± 0.2	1 ± 0.1
AF1039		Chemotaxis protein CheC, inhibitor of MCP methylation*	N	-				0.8	0.2	0.9	1.3 ± 0.4	1.8 ± 0.1
AF1040	cheA	chemotaxis histidine kinase	N	-				1 ± 0.1	0.3	1.1	1.6 ± 0.4	2.3 ± 0.2
AF1041	cheB	chemotaxis-specific methylesterase	N	-				1 ± 0.1	0.3	1.1 ± 0.1	1.6 ± 0.4	2.1 ± 0.2
AF1042	cheY	chemotaxis response regulator	T	-				0.5 ± 0.1	0.1	0.6	1.1 ± 0.4	1.3 ± 0.1
AF1043		uncharacterized conserved protein, containing HTH domain*	K	-				1 ± 0.1	0.2	1.1 ± 0.1	1.8 ± 0.5	2.4 ± 0.2
AF1044	cheW	purine-binding chemotaxis protein	N	-				1.2 ± 0.1	0.3	1.4 ± 0.1	2.4 ± 0.5	3
AF1045	tlpC-2	methyl-accepting chemotaxis protein	T	-	**2.86**			0.8 ± 0.1	0.5	0.9	2.1 ± 0.5	2.7 ± 0.2
AF1046		Signal transduction histidine kinase*	T	-				0.1	0.2	0.1	0.2 ± 0.1	0.5 ± 0.1
AF1047		hypothetical protein	X	-				0.3	0.5	0.5 ± 0.1	0.8 ± 0.3	1.4 ± 0.1

AF1048		flagellar assembly protein J	N	-				0.4	0.7 ± 0.1	0.6 ± 0.1	1.1 ± 0.5	2 ± 0.1
AF1049	gspE-4	type II secretion system protein	N	-				0.9 ± 0.1	1.3 ± 0.2	1.4 ± 0.2	2 ± 0.5	2.8
AF1050		flagellar accessory protein FlaH	N	-				1 ± 0.1	0.6	1.3 ± 0.1	1.7 ± 0.5	2 ± 0.2
AF1051		Putative archaeal flagellar protein F*	N	-				1.2 ± 0.1	0.8 ± 0.1	1.6 ± 0.1	2.1 ± 0.5	2.5
AF1052		Putative archaeal flagellar protein G*	N	-				1.2 ± 0.1	0.9	1.5 ± 0.1	2 ± 0.5	2.6
AF1053		uncharacterized conserved protein*	S	-				0.4 ± 0.1	0.3	0.5	1 ± 0.5	1.3 ± 0.1
AF1054	flaB1-1	flagellin	N	-	**1.94**			1.8 ± 0.1	1.3 ± 0.1	2.1	**3.4**	**3.5**
AF1055	flaB1-2	flagellin	N	-	**2.54**			1.3 ± 0.1	1.1 ± 0.2	1.7 ± 0.1	**3.5 ± 0.2**	**3.7**
AF1056		RNA base methyltransferase family enzyme*	J	-				0.2	0.1	0.2	0.1	0.1
AF1057	ccdA	cytochrome C-type biogenesis protein	O	-				0.2 ± 0.1	0.1	0.1	0.1	0.1
AF1058		Regulatory protein involved in competence development and sporulation*	L	-				0.3	0.4 ± 0.1	0.2 ± 0.1	0.2	0.4 ± 0.1
AF1059		hypothetical protein	X	+				0.1	0.1	0.1	0.1	0.1
AF1060		HerA helicase*	L	+				1.1 ± 0.1	1.2 ± 0.1	1.1	1.1	1.1 ± 0.1
AF1061		Predicted ATP-utilizing enzyme (ATP-grasp superfamily)*	R	-				0.6 ± 0.1	0.6	0.6 ± 0.1	0.5 ± 0.1	0.6
AF1062		uncharacterized conserved protein*	S	-			1.43	1 ± 0.1	1.3 ± 0.1	0.9 ± 0.1	0.9 ± 0.1	1.4 ± 0.1
AF1063		response regulator	T	-				0.2	0.3	0.2	0.2	0.2
AF1064		ABC transporter, ATP-binding protein, putative	R	-	**2.02**			0.3	0.3 ± 0.1	0.3	0.5	0.7 ± 0.1
AF1065		ASCH domain, predicted RNA-binding domain*	R	+				0.1	0.1	0.1	0.1	0.1
AF1066	mer-1	methylenetetrahydromethanopterin reductase	C	+	-1.10			2.7	2.8	2.8	2.5	2.4
AF1067		chromatin protein	K	+				1.8 ± 0.2	2.3	1.4 ± 0.1	1.4 ± 0.1	1.4 ± 0.1
AF1068		uncharacterized conserved protein*	S	+	-1.39			0.5 ± 0.1	0.4	0.4	0.3	0.3
AF1069	panF-1	pantothenate permease	H	-				2.8	2.8	2.8	2.8	2.7
AF1070	ftsA-1	coenzyme F390 synthetase	H	-				3.1	3.1	3	3.1	3
AF1071		Transposase*	L	+				0	0.1	0.1	0.1	0
AF1072		Predicted nuclease of the RNAse H fold, HicB family*	R	-			1.30	0	0	0	0	0
AF1075		Cytotoxic translational repressor of toxin-antitoxin stability system*	V	-				0.1	0.1	0.1	0.1	0.1
AF1076		hypothetical protein	X	-				0.1	0.2	0.1	0.1	0.2
AF1077		PIN domain containing protein*	V	-				0.3	0.2	0.3	0.3	0.3
AF1078		hypothetical protein	X	-				0.3	0.2	0.3	0.2	0.2
AF1079		DNA-binding protein, potential antitoxin AbrB/MazE fold*	K	-				0.2	0.2	0.2	0.2	0.3
AF1080		Cytotoxic translational repressor of toxin-antitoxin stability system*	V	-				0.1	0.1	0.1	0.1	0.1
AF1081		uncharacterized conserved protein*	S	-				0.1	0.1	0.1	0.1	0.2
AF1084		DNA-binding protein, potential antitoxin AbrB/MazE fold*	K	-				0.1	0.1	0.1	0.1	0.1
AF1086m		PIN domain containing protein*	V	-				0.3	0.4	0.4 ± 0.1	0.3	0.4
AF1087		DNA-binding protein, potential antitoxin AbrB/MazE fold*	K	-				0.2	0.3	0.2	0.2	0.4
AF1088		uncharacterized conserved protein*	S	-				0.1	0.1	0.1	0.1	0.1
AF1089		PIN domain containing protein*	V	-				0.4 ± 0.1	0.3	0.5 ± 0.1	0.5 ± 0.1	0.5
AF1090		DNA-binding protein, potential antitoxin AbrB/MazE fold*	K	-				0.3	0.3	0.3 ± 0.1	0.4 ± 0.1	0.4
AF1091		PIN domain containing protein*	V	-				0.5	0.9 ± 0.1	0.8 ± 0.1	1.1 ± 0.1	1.4 ± 0.1
AF1092		DNA-binding protein, potential antitoxin AbrB/MazE fold*	K	-				0.4 ± 0.1	0.3	0.6 ± 0.1	0.5 ± 0.1	0.6 ± 0.1
AF1094		hypothetical protein	X	-				0.2	0.2	0.3	0.3	0.3 ± 0.1
AF1096		Predicted flagellin FlaG*	N	-			1.23	2.3	**3.1**	2.5	**3**	**3.3**
AF1097	manC	mannose-6-phosphate isomerase/mannose-1-phosphate guanylyl transferase	M	-				0.3	0.3	0.3	0.2	0.2
AF1098	fum-1	fumarate hydratase	C	-				0.4 ± 0.1	1.5 ± 0.1	0.3	1.4 ± 0.1	1.9 ± 0.1
AF1099	fum-2	fumarate hydratase	C	-				0.4 ± 0.1	1.3	0.4	1.5 ± 0.1	1.6 ± 0.1
AF1100	cdhA-1	acetyl-CoA decarbonylase/synthase complex subunit alpha	C	+		**1.57**		2.3 ± 0.3	1.9 ± 0.1	**3.4**	**3.5**	**3.5**
AF1101	cdhB-1	acetyl-CoA decarbonylase/synthase complex subunit epsilon	C	+		**2.12**		1.5 ± 0.2	1.4 ± 0.1	**3**	**3**	**3.4**
AF1102		uncharacterized conserved protein*	S	+				0.2	0.2	1 ± 0.2	0.7 ± 0.1	1.6 ± 0.1
AF1103		Predicted nuclease (RNAse H fold)*	R	+				0.1	0.1	0.2 ± 0.1	0.2	0.3
AF1104		Predicted ATP-grasp domain fused to redox center*	R	+	-1.41			0.4	0.4	0.4	0.3	0.3
AF1105		uncharacterized protein containing a Zn-finger-like domain*	R	+				0.5 ± 0.1	0.6	0.5	0.3	0.4
AF1106		ribosomal biogenesis protein	J	+				0.1	0.1	0.1	0.1	0.1
AF1107		uncharacterized conserved protein*	S	-				0.4	0.2	0.3	0.3	0.4
AF1108		deoxyuridine 5'-triphosphate nucleotidohydrolase	F	-				0.2	0.3	0.2	0.2	0.2
AF1109		SWIM zinc finger*	S	-				1 ± 0.2	1	1.1 ± 0.1	1 ± 0.2	1
AF1110		uncharacterized conserved protein*	S	-				1 ± 0.1	1	1	0.9 ± 0.1	0.9
AF1111		uncharacterized conserved protein*	S	+				0.4 ± 0.1	0.3	0.4	0.3	0.2
AF1112	gcp	O-sialoglycoprotein endopeptidase	L	-				1.2 ± 0.2	1.1 ± 0.1	1.2 ± 0.1	1 ± 0.1	0.8
AF1113	rps27AE	SSu ribosomal protein S27AE	J	-				1.7 ± 0.2	1.7	1.8 ± 0.1	1.5 ± 0.1	1.2
AF1114	rps24E	SSu ribosomal protein S24E	J	-				2 ± 0.1	2.1	2	1.9 ± 0.1	1.7 ± 0.1
AF1115		uncharacterized conserved protein*	S	-				1.2	1.1 ± 0.1	1.3 ± 0.1	1.2 ± 0.1	1.2 ± 0.1
AF1116	rpoE2	DNA-directed RNA polymerase, subunit E''	K	-				1.5 ± 0.1	1.5	1.5 ± 0.1	1.5 ± 0.2	1.5
AF1117	rpoE1	DNA-directed RNA polymerase subunit E'	K	-				1.2 ± 0.1	1.5 ± 0.1	1.3 ± 0.1	1.4 ± 0.1	1.3 ± 0.1
AF1118		hypothetical protein	X	-				0.1	0.4	0.1	0.3 ± 0.1	0.3
AF1119		uncharacterized conserved protein*	S	+				0.9 ± 0.1	1.3 ± 0.1	0.3	0.7 ± 0.2	0.4 ± 0.1
AF1120		hypothetical protein	X	+				0.3	0.5 ± 0.1	0.2	0.3 ± 0.1	0.3
AF1121		AsnC family transcriptional regulator	K	+			1.30	0.4	0.5	0.3	0.4	0.5
AF1122	hbd-5	3-hydroxyacyl-CoA dehydrogenase	I	+	**2.43**			0.5	0.8 ± 0.1	0.4	1.6 ± 0.2	1.4 ± 0.1
AF1123		Poly(3-hydroxyalkanoate) synthetase*	I	+	1.29			0	0	0	0.1	0.1
AF1124		Acyl dehydratase*	I	+				0.1	0.1	0.1	0.1	0.1
AF1125	nirJ-1	heme biosynthesis protein	R	+	**-1.54**			0.4	0.4	0.4	0.3	0.2
AF1126		P450 cytochrome, putative	R	-	-1.46			0.2	0.2	0.2	0.1	0.1
AF1127	rpl18E	50S ribosomal protein L18e	J	+				3.3	3.3	3.3	3.3	3.3
AF1128	rpl13P	50S ribosomal protein L13P	J	+				3.1	3.2	3	3	3.1
AF1129	rps9p	30S ribosomal protein S9P	J	+				2.4	2.9	2.3	2.3	2.5
AF1130	rpoN	DNA-directed RNA polymerase subunit N	K	+	-1.43	-1.38		0.6	0.6	0.5 ± 0.1	0.4	0.4
AF1131	rpoK	DNA-directed RNA polymerase, subunit K	K	+			**-1.54**	0.7 ± 0.1	0.5	0.8 ± 0.1	0.6 ± 0.1	0.4
AF1132	eno	phosphopyruvate hydratase	G	+				3	2.9	3	2.9	2.8
AF1133	rps2P	30S ribosomal protein S2	J	+				3.2	3.3	3.2	3.2	3.2
AF1134		hypothetical protein	X	+				0.4 ± 0.1	0.4	0.3	0.6 ± 0.1	0.5
AF1135		uncharacterized conserved protein*	S	+		**-1.69**		0.1	0.1	0	0	0
AF1136		ABC transporter, ATP-binding protein	V	+	**-2.09**	**-1.75**		**3.2**	**3**	2.5	1.4 ± 0.2	1.2 ± 0.1
AF1137		ABC-type transport system involved in multi-copper enzyme maturation, permease component*	R	+	**-2.10**	**-1.77**		**3.4**	**3.1**	2.7 ± 0.2	1.4 ± 0.2	1.3 ± 0.2
AF1138		Predicted membrane protein*	S	+	**-2.46**	**-2.01**		**3**	2.7	2.2 ± 0.2	1.1 ± 0.2	0.9 ± 0.2
AF1139		ABC transporter, ATP-binding protein	V	+	**-3.70**			2.4 ± 0.2	1.5 ± 0.2	1.2 ± 0.1	0.5 ± 0.1	0.3 ± 0.1
AF1140		Predicted permease*	R	+	**-2.97**			2.1	1.3 ± 0.1	1.4 ± 0.1	0.6	0.4 ± 0.1
AF1141	acd-8	acyl-CoA dehydrogenase	I	-	-1.22			2.3	2.5	2.5	2.1	1.8 ± 0.1
AF1142	rfbF	glucose-1-phosphate cytidylyltransferase	M	-	**-1.53**			0.9 ± 0.1	1 ± 0.1	0.8 ± 0.1	0.6 ± 0.1	0.5
AF1143	pgsA-1	CDP-diacylglycerol--glycerol-3-phosphate-3-phosphatidyltransferase	I	+	-1.29			0.4	0.4	0.4	0.3	0.3
AF1144		multidrug resistance protein	G	-				0.6 ± 0.1	0.6	0.5	0.5 ± 0.1	0.5
AF1145	cat2-1	4-hydroxybutyrate CoA transferase	C	-				1.3 ± 0.1	2.5 ± 0.2	1.2 ± 0.2	1.4	0.8
AF1146	pgk	3-phosphoglycerate kinase	G	-				1.5 ± 0.1	1.5 ± 0.1	1.4 ± 0.1	1.6 ± 0.1	1.3 ± 0.1
AF1147	argJ	bifunctional ornithine acetyltransferase/N-acetylglutamate synthase protein	E	+				0.9 ± 0.1	1.2	0.9 ± 0.1	0.8 ± 0.1	0.8 ± 0.1
AF1148		AsnC family transcriptional regulator	K	-				0.3	0.3	0.3	0.3	0.3
AF1149	lhr-1	large helicase-related protein	R	+		-1.38		1.3 ± 0.1	1.4	1	1 ± 0.1	0.8
AF1149a		Subunit of KEOPS complex (Cgi121BuD32KAE1)*	J	+				1.3 ± 0.1	1.6 ± 0.1	1.1 ± 0.1	1 ± 0.1	1 ± 0.1
AF1150		erpK protein, putative	O	+				1 ± 0.1	1.5 ± 0.1	0.9 ± 0.1	0.9	0.8
AF1151		hypothetical protein	X	-				0.1	0.1	0.1	0	0.1
AF1152		Predicted pseudouridylate synthase*	J	+				0.4	0.5	0.4	0.3	0.3
AF1153		iron-sulfur cluster binding protein	R	-				1.8 ± 0.1	1.9	1.9 ± 0.1	2.2 ± 0.2	2.3
AF1154		KaiC-like protein*	R	-				1.2 ± 0.1	1.3 ± 0.1	1.1 ± 0.1	1.5 ± 0.2	2.1
AF1155		Metallophosphoesterase superfamily enzyme*	R	+				0.1	0.2	0.1	0.2	0.4
AF1156		uncharacterized conserved protein*	S	+				0.2	0.2	0.2	0.2	0.2

AF1157	purD	phosphoribosylamine--glycine ligase	F	-			1.1 ± 0.2	1.3 ± 0.1	1.1 ± 0.1	0.8 ± 0.1	0.6
AF1158		ATP synthase, subunit E, putative	C	+			**3.2**	**3.4**	**3.2**	**3.2**	**3.2**
AF1159	atpI	V-type ATP synthase subunit I	C	+			**3.5**	**3.6**	**3.5**	**3.5**	**3.5**
AF1160	atpK-1	H+-transporting ATP synthase, subunit K	C	+			**3.6**	**3.6**	**3.6**	**3.5**	**3.4**
AF1163	atpE	V-type ATP synthase subunit E	C	+	-1.07		**3.3**	**3.4**	**3.3**	**3.1**	**3.1**
AF1164	atpC	V-type ATP synthase subunit C	C	+			**3.5**	**3.6**	**3.5**	**3.3**	**3.4**
AF1165	atpF	V-type ATP synthase subunit F	C	+			**3.2 ± 0.2**	**3.2**	**3.2**	**3.1 ± 0.2**	**3.1 ± 0.2**
AF1166	atpA	V-type ATP synthase subunit A	C	+			**3.5**	**3.5**	**3.5**	**3.4**	**3.3**
AF1167	atpB	V-type ATP synthase subunit B	C	+			**3.5**	**3.6**	**3.5**	**3.5**	**3.4**
AF1168	atpD	V-type ATP synthase subunit D	C	+	-1.13		**3.2**	**3.2**	**3.1**	2.8	2.7 ± 0.2
AF1169		uncharacterized conserved protein*	R	+	**-2.02**		0.7	0.7	0.6	0.3 ± 0.1	0.3
AF1170	rbsA-2	ribose ABC transporter, ATP-binding protein	P	+	**-1.74**		0.3	0.3	0.3	0.2	0.1
AF1171		hypothetical protein	X	+			0.1	0.1	0.1	0.1	0.1
AF1172		RecA-superfamily ATPase implicated in signal transduction*	T	-			0.7 ± 0.1	0.9	0.7	1 ± 0.1	1 ± 0.1
AF1173		uncharacterized conserved protein*	S	+	**1.74**		0	0.1	0	0.1	0.1
AF1174		Putative sterol carrier protein*	I	+	**11.03**		0.1	0.5 ± 0.1	0.1	2.7	2.9
AF1175	acdS	acyl-CoA dehydrogenase, short chain-specific	I	+	**11.06**		0.1	0.4	0.1	2.5	2.5
AF1176		hypothetical protein	X	+	**7.51**		0.3	0.4	0.2	2.4	2.2
AF1177	hbd-6	3-hydroxyacyl-CoA dehydrogenase	I	+	**6.48**		0.2	0.2	0.2	1.2 ± 0.1	1.3 ± 0.1
AF1178		Predicted pyrophosphatase*	R	+			0.1	0.1	0.1	0.1	0.1
AF1179		GTP cyclohydrolase	H	+	-1.31		1.6 ± 0.1	1.7	1.6 ± 0.1	1.2 ± 0.1	1.2
AF1180		Phosphatidylserine/phosphatidylglycerophosphate/cardiolipin synthase or related enzyme*	I	+			1.1 ± 0.1	1 ± 0.1	1 ± 0.1	0.8 ± 0.1	0.7
AF1181		GTP-binding protein	R	+			1.6 ± 0.1	2.3	1.8 ± 0.1	2.2	2.2
AF1182		uncharacterized conserved protein*	S	+		1.19	2.3	2.9 ± 0.2	2.4	2.8	**3.1**
AF1183		Zn-ribbon containing protein*	R	+			1.2	1.6 ± 0.1	1.3	1.5 ± 0.1	1.6 ± 0.1
AF1184		signal-transducing histidine kinase	T	+			0.6	0.8	0.7	0.8 ± 0.1	0.7 ± 0.1
AF1185		iron-sulfur cluster binding protein	C	-			1.2 ± 0.1	1.3	1.4 ± 0.1	0.8 ± 0.1	1.1 ± 0.1
AF1186		uncharacterized conserved DuF39 domain fused to CBS domain*	S	-	-1.30		2.4	2.1	2.7	1.8 ± 0.1	1.7 ± 0.1
AF1187		Predicted nucleic acid binding protein containing the AN1-type Zn-finger*	R	-			0.6 ± 0.1	0.4	0.5 ± 0.1	0.4 ± 0.1	0.6 ± 0.1
AF1188		Membrane-bound metal-dependent hydrolase*	R	-			0.9 ± 0.1	0.6	0.6	0.6 ± 0.1	0.8 ± 0.1
AF1189		hypothetical protein	X	-			1.2 ± 0.1	0.9 ± 0.1	0.9 ± 0.1	0.9 ± 0.1	1.2 ± 0.1
AF1190	hbd-7	3-hydroxyacyl-CoA dehydrogenase	I	-	1.48		1.2 ± 0.1	1.6 ± 0.2	1.2 ± 0.1	2	2
AF1191	menB	dihydroxynaphthoic acid synthase	H	-	**1.79**		1 ± 0.1	1.3 ± 0.1	1.1 ± 0.1	2.1 ± 0.2	2.2
AF1192		Acyl-CoA synthetase, ATP-grasp containing subunit*	C	-	**2.43**		0.1	0.4	0.1	0.6 ± 0.1	0.6
AF1193		Metallophosphoesterase superfamily enzyme*	R	-			0.2 ± 0.1	0.3	0.2	0.2	0.2
AF1194		Predicted HTH domain, homologous to N-terminal domain of RPA1 protein family*	R	-			0.6 ± 0.1	0.8 ± 0.1	0.6 ± 0.1	0.5 ± 0.1	0.7
AF1195		replication factor C large subunit	L	-	1.25		1.1	1.2 ± 0.1	1.1 ± 0.1	1.6 ± 0.1	1.4
AF1196	mer-2	N5,N10-methylenetetrahydromethanopterin reductase	C	-			0.8 ± 0.1	1.9 ± 0.1	0.9 ± 0.1	2.1	1.8
AF1197	fadA-2	3-ketoacyl-CoA thiolase	I	-			1.9 ± 0.1	2.4	2.1	2	2.2
AF1199	gctA	glutaconate CoA-transferase, subunit A	I	-			0.3	0.3	0.3	0.2	0.1
AF1200		Kynurenine formamidase*	E	-			0.2	0.3	0.2	0.2	0.2
AF1201		uncharacterized conserved protein*	S	+			0.2	0.2	0.1	0.2	0.2
AF1202		molybdopterin oxidoreductase, iron-sulfur binding subunit	C	-			0.1	0.1	0.1	0.1	0.1
AF1203		molybdopterin oxidoreductase, molybdopterin binding subunit	C	-		**-1.56**	1 ± 0.1	0.6	0.8 ± 0.1	0.8 ± 0.1	0.4
AF1204		hypothetical protein	X	-		-1.47	0.4	0.2	0.4	0.3	0.2
AF1205	panF-2	pantothenate permease	E	-		-1.48	0.9 ± 0.1	0.5	0.8 ± 0.1	0.9 ± 0.1	0.6
AF1206	hbd-8	3-hydroxyacyl-CoA dehydrogenase	I	-			0.7 ± 0.1	0.4	0.6 ± 0.1	0.9 ± 0.1	0.5 ± 0.1
AF1207	kduD	2-deoxy-D-gluconate 3-dehydrogenase	I	-	1.40		0.8 ± 0.1	0.7 ± 0.1	0.8 ± 0.1	1.4 ± 0.2	1.1 ± 0.1
AF1208		uncharacterized conserved protein, contains double-stranded beta-helix domain*	S	-	**1.61**		0.1	0.1	0.1	0.2	0.2
AF1209		Predicted dinucleotide-binding enzyme*	R	-			0.1	0.1	0.1	0.1	0.1
AF1210		uncharacterized conserved protein*	S	-			0.1	0.2	0.1	0.1	0.1
AF1211		Acyl-CoA synthetase (NDP forming)*	C	+	**-1.60**		0.7 ± 0.1	0.9 ± 0.1	0.8	0.5	0.5
AF1212		BioD-like N-terminal domain of phosphotransacetylase*	R	+	-1.43		0.7	0.7	0.7	0.5	0.4
AF1213		uncharacterized conserved protein*	S	+			0.2	0.2	0.2	0.2	0.2
AF1214	pad1	phenylacrylic acid decarboxylase	H	-		1.24	0.9 ± 0.1	1.1 ± 0.1	0.9	1 ± 0.1	1.3 ± 0.1
AF1215		cell division protein, putative	D	-			2.3	2.2	2.2	2.4	2.4
AF1216	lysK	lysyl-tRNA synthetase	J	+	**-1.51**		2.4	2	2.2	1.6 ± 0.1	1.2 ± 0.1
AF1217		uncharacterized conserved protein related to pyruvate formate-lyase activating enzyme*	R	+	-1.40		1.6 ± 0.1	1.6 ± 0.1	1.6 ± 0.1	1.1 ± 0.1	1.1
AF1218		uncharacterized conserved protein*	S	+	-1.47		0.7 ± 0.1	0.7 ± 0.1	0.7 ± 0.1	0.5	0.4
AF1219		undecaprenyl pyrophosphate synthase*	I	+	-1.40		1.7 ± 0.1	1.6 ± 0.1	1.6 ± 0.1	1.2 ± 0.1	1
AF1220		peptide chain release factor 1	J	+	-1.39		1.8 ± 0.1	1.8 ± 0.1	1.7 ± 0.1	1.2 ± 0.1	1.2
AF1221		hypothetical protein	X	+			1.7 ± 0.2	1.2 ± 0.1	1.5 ± 0.2	0.9 ± 0.2	0.8
AF1222	putP-3	proline permease	E	+			2.2 ± 0.2	2 ± 0.1	2.2	2	2 ± 0.1
AF1223		Permease of the drug/metabolite transporter (DMT) superfamily*	G	-			0.6 ± 0.1	0.4	0.6 ± 0.1	0.4	0.4
AF1224		Predicted HD superfamily hydrolase*	R	-			0.6 ± 0.1	0.4	0.4	0.3	0.4
AF1225		uncharacterized conserved protein*	S	-			1 ± 0.2	0.8 ± 0.1	1 ± 0.1	1.5 ± 0.2	1.8 ± 0.1
AF1226		uncharacterized conserved protein*	S	-			2.2 ± 0.2	2.2 ± 0.3	2.4	2.8 ± 0.2	**3.1**
AF1227		hypothetical protein	X	-			0.5 ± 0.1	0.3 ± 0.1	0.6 ± 0.1	0.9 ± 0.1	0.9 ± 0.1
AF1228		hypothetical protein	X	-			0.2	0.1	0.2	0.2 ± 0.1	0.2
AF1229		Predicted exporter of the RND superfamily*	R	-	-1.46		1.2 ± 0.1	1.1	1 ± 0.1	0.8 ± 0.1	0.7
AF1230		S-layer domain*	M	-			2.1 ± 0.2	1.6	1.9 ± 0.1	1.5 ± 0.2	0.9
AF1231		S-layer domain*	M	-	-1.27		2.7	2.6	2.6	2.1	1.9
AF1232		Sugar-specific transcriptional regulator TrmB*	K	+	**-1.55**		0.6 ± 0.1	0.5	0.5 ± 0.1	0.4	0.3
AF1233		Histidinol phosphatase or related hydrolase of the PHP family*	E	+	-1.36		0.7	0.7	0.7	0.5	0.5
AF1234	mutT	mutator protein MutT	F	+	-1.48		0.3	0.3	0.3	0.2	0.1
AF1235		transcription-associated protein TFIIS	K	+	**-1.63**		0.3	0.2	0.3	0.2	0.1
AF1236		hypothetical protein	X	+		**1.71**	0.1	0.2	0.1	0.2	0.2
AF1237		Membrane-bound tetraheme cytochrome c subunit*	C	-			0.2	0.4	0.3	0.5 ± 0.1	0.4 ± 0.1
AF1238		heterodisulfide reductase, subunit A/methylviologen reducing hydrogenase, subunit delta	C	+			0.7	0.8	0.8	0.7	0.7
AF1239	fdx-7	ferredoxin	C	-			0.9 ± 0.1	0.7 ± 0.1	1.1 ± 0.1	1 ± 0.1	0.9 ± 0.1
AF1240	trpB-1	tryptophan synthase subunit beta	R	-			**3**	2.9	**3.1**	**3.2**	**3.2**
AF1241	hemL	glutamate-1-semialdehyde aminotransferase	H	+			1.2 ± 0.2	1.5	1.2 ± 0.1	1.1 ± 0.1	0.8
AF1242	hemC	porphobilinogen deaminase	H	+			2.2	2.5	2.3	2.1	1.7 ± 0.1
AF1243	cysG-2	uroporphyrin-III C-methyltransferase	H	+			1.1	1.2 ± 0.1	1.1	0.9 ± 0.1	0.7
AF1244		Putative regulatory, ligand-binding protein related to C-terminal domain of K+ channels*	P	+			1.3 ± 0.1	1.6	1.3	1.7 ± 0.1	1.7 ± 0.1
AF1245	napA-2	Na+/H+ antiporter	P	+	1.17		1.3 ± 0.1	1.4	1.3	1.6 ± 0.1	1.5
AF1246		Kef-type K+ transport system, predicted NAD-binding component*	P	-			0.1	0.1	0.1	0.1	0.1
AF1247		2-methylthioadenine synthetase*	J	+		-1.48	0.4	0.3	0.5	0.3	0.2
AF1248		uncharacterized membrane protein, possible Na+ channel or pump*	R	-	-1.43		0.3	0.3	0.3	0.2	0.2
AF1249		hypothetical protein	X	-			0.2	0.2	0.2	0.1	0.1
AF1250		uncharacterized conserved protein*	S	-		1.30	1.4 ± 0.1	2.2	1.5	1.5 ± 0.1	1.8
AF1251		Archaeal enzyme of ATP-grasp superfamily*	R	-			2.5	2.6	2.6 ± 0.2	2.5	2.4
AF1252m	oadA	oxaloacetate decarboxylase	C	-			1.8 ± 0.2	2	1.8 ± 0.1	1.5 ± 0.1	1.5 ± 0.1
AF1253		uncharacterized conserved protein*	S	+			0.4	0.3	0.5	0.6 ± 0.1	0.6
AF1254		Acetyltransferase (GNAT) family*	K	+	**-2.68**		1.8 ± 0.1	1.8 ± 0.1	1.6 ± 0.1	0.6	0.6
AF1255	argF	ornithine carbamoyltransferase	E	-		1.24	1.6 ± 0.1	2	1.6	1.5 ± 0.1	2 ± 0.1
AF1256		response regulator	T	-			0.4 ± 0.1	0.3	0.5	0.3	0.5
AF1257		Predicted DNA modification methylase*	L	-			0.5 ± 0.1	0.5 ± 0.1	0.5	0.5 ± 0.1	0.4
AF1258	srp19	signal recognition particle, subunit SRP19	U	-			1.2 ± 0.2	1 ± 0.1	1.3	1.3 ± 0.1	1.2
AF1259		inosine monophosphate dehydrogenase, putative	K	-			0.7 ± 0.1	0.5 ± 0.1	0.8	0.8 ± 0.1	0.8
AF1260	purQ	phosphoribosylformylglycinamidine synthase subunit I	F	-		1.35	0.7 ± 0.1	1	0.6	0.6	0.8
AF1261	alkK-4	medium-chain acyl-CoA ligase	I	-			0	0	0	0	0
AF1263		iron-sulfur cluster binding protein	C	-		-1.48	1.7 ± 0.1	1.2 ± 0.1	1.9 ± 0.2	1.6 ± 0.1	1.1 ± 0.1

AF1264		Transcriptional regulator containing HTH domain, ArsR family*	K	+				0.2	0.3	0.3	0.3	0.3
AF1265		metal-dependent hydrolase	R	-				0.8 ± 0.1	0.7 ± 0.1	1 ± 0.1	1.2 ± 0.1	1.2 ± 0.1
AF1266		hypothetical protein	X	+				0.1	0.1	0.1	0.1	0.2
AF1267		hypothetical protein	X	+				0	0	0	0	0
AF1268		hypothetical protein	X	+				0.1	0.1	0.1	0.1	0.1
AF1269		hypothetical protein	X	+				0	0	0	0	0
AF1270		ArsR family transcriptional regulator	K	+				0.2	0.2	0.2	0.1	0.1
AF1271	purE	phosphoribosylaminoimidazole carboxylase	F	+	-1.45			0.4	0.5	0.4	0.3	0.3
AF1272	hemH	phosphoribosylaminoimidazole-succinocarboxamide synthase	F	+			-1.35	1.6 ± 0.1	1.2 ± 0.1	1.5 ± 0.1	1.3 ± 0.1	0.9 ± 0.1
AF1273	carA	carbamoyl phosphate synthase small subunit	E	+			-1.17	2.7	2.3	2.6	2.4	2
AF1274	carB	carbamoyl-phosphate synthase, large (or ammonia) subunit	E	+	-1.29			2.2	2 ± 0.1	2	1.7 ± 0.1	1.4
AF1275		NCAIR mutase (PurE)-related protein*	R	+				1.1 ± 0.1	0.7	1 ± 0.1	0.7	0.5
AF1276		hypothetical protein	X	-			**-2.12**	0.7	0.4	0.7 ± 0.1	0.5 ± 0.1	0.2
AF1277		GMP synthase - Glutamine amidotransferase domain*	F	+				0.2	0.3	0.2	0.3	0.3
AF1278		Enzyme related to plant Lysine-oxoglutarate reductase/Saccharopine dehydrogenase*	R	-				1.9 ± 0.1	2 ± 0.1	1.9 ± 0.1	1.9 ± 0.1	1.7
AF1279		Sugar phosphate isomerase/epimerase*	G	-				**3**	2.9	2.9	2.9	**3.1 ± 0.2**
AF1280	argB	acetylglutamate kinase	E	-			-1.19	1.5 ± 0.1	1.2	1.4 ± 0.1	1.5 ± 0.1	1.2 ± 0.1
AF1281	pfpI	intracellular protease	R	-				0.3	0.2	0.3	0.3	0.1
AF1282		hypothetical protein	X	+			1.49	0.2	0.4	0.3	0.2	0.4
AF1283		uncharacterized conserved protein*	S	+				0.7 ± 0.1	1	0.7	0.6	0.8
AF1284	trx-3	thioredoxin	O	+				0.8 ± 0.1	1	0.9 ± 0.1	0.7	0.8
AF1285		cell division protein CDC48	R	-				0.7	0.7 ± 0.1	0.6 ± 0.1	0.6	0.7
AF1286		acetoin utilization protein, putative	R	-				0.3	0.2	0.3	0.3 ± 0.1	0.4
AF1287	acs-6	acetyl-CoA synthetase	I	-	1.39	1.36		2.1	2 ± 0.1	2.3	**3**	**3**
AF1288a		methylmalonyl-CoA mutase N-terminal domain-containing protein	I	+				2.9	2.2	**3.1**	2.6	2.8
AF1288b		methylmalonyl-CoA mutase C-terminal domain-containing protein	I	+				2.9	1.8 ± 0.2	**3.2**	2.5 ± 0.3	2.6 ± 0.3
AF1289		Putative periplasmic protein kinase ArgK or related GTPase of G3E family*	E	+				2.4	1.6	2.6	1.9 ± 0.1	2.1
AF1290		cob(I)alamin adenosyltransferase*	H	+				2	1.4 ± 0.1	2.3	1.6 ± 0.3	1.8 ± 0.2
AF1291	acaB-11	3-ketoacyl-CoA thiolase	I	+				2.6	1.8	2.7	2.1	2.1
AF1292		Predicted nucleic-acid-binding protein containing a Zn-ribbon*	R	+				2.4	1.4 ± 0.1	2.5	1.9 ± 0.1	1.9 ± 0.1
AF1293	acd-9	acyl-CoA dehydrogenase	I	+				1.5 ± 0.1	1	1.6 ± 0.1	1 ± 0.1	1.3 ± 0.1
AF1294		uncharacterized membrane protein*	S	-				0.5 ± 0.1	0.5	0.6 ± 0.1	0.3	0.4
AF1295		uncharacterized conserved protein*	S	-				0.2	0.2	0.2	0.1	0.1
AF1296	hsp20-1	small heat shock protein	O	-				**3**	**3.1**	**3.2**	2.7	2.7
AF1297	cdc48-1	cell division protein CDC48	O	-				**3.4**	**3.3**	**3.4**	**3.3**	**3.2**
AF1298		Predicted transcriptional regulator*	K	-				1.1 ± 0.2	1.1 ± 0.1	1.9 ± 0.4	0.7 ± 0.1	0.8 ± 0.2
AF1298a		RNA-binding protein involved in rRNA processing*	J	+				0.5	0.4	0.5 ± 0.1	0.5	0.6 ± 0.1
AF1299	tfb	transcription initiation factor IIB	K	+				2.4	2.5	2.4	2.4	2.9
AF1300		ABC transporter, ATP-binding protein	V	+	-1.34			0.5	0.6	0.5	0.4	0.4
AF1301		hypothetical protein	X	+				0.8 ± 0.1	0.7	0.8 ± 0.1	0.6 ± 0.1	0.4
AF1302		ABC-type multidrug transport system, permease component*	V	+				0.6 ± 0.1	0.5	0.6 ± 0.1	0.5	0.3
AF1303		hypothetical protein	X	+				0.4	0.4	0.4	0.3	0.3
AF1304	tpiA	triosephosphate isomerase	G	-	-1.20			1 ± 0.1	0.9	0.9	0.7	0.7
AF1305	hps-2	bifunctional formaldehyde-activating enzyme/3-hexulose-6-phosphate synthase	G	-				2.2	2.2	2.1	2 ± 0.1	1.8
AF1306		uncharacterized conserved protein*	S	-	**2.03**			0.1	0.1	0.1	0.3	0.2
AF1307		uncharacterized conserved protein*	S	+	**-1.55**			0.3	0.3	0.3	0.2	0.2
AF1308		thymidylate kinase, putative	F	-				0.2	0.1	0.2	0.2	0.2
AF1309		Dolichol kinase*	I	-				0.2	0.1	0.3	0.2	0.2
AF1310		ISA0963-3 transposase	L	+			1.26	0.5 ± 0.1	0.8 ± 0.1	0.7	0.7 ± 0.1	0.8
AF1311		oxygen-independent coproporphyrinogen III oxidase, putative	C	+				0.8	1	0.8	0.7	0.6
AF1312		Outer membrane lipoprotein-sorting protein*	M	-				1.9 ± 0.1	**3.1**	1 ± 0.1	2.6	2.8
AF1313		3-hydroxy-3-methylglutaryl CoA synthase family enzyme*	R	+				0.2	0.2	0.2	0.3	0.3
AF1314		Pyridine nucleotide-disulphide oxidoreductase*	R	-			-1.32	1.1 ± 0.1	0.8 ± 0.1	1.3 ± 0.1	1.3 ± 0.1	1 ± 0.1
AF1315	baiF-3	bile acid-inducible operon protein F	C	-				1.3 ± 0.1	1.5 ± 0.1	1.5 ± 0.1	1.6 ± 0.1	1.7 ± 0.1
AF1316	thrC-2	threonine synthase	E	+				1.1 ± 0.1	0.9 ± 0.1	1 ± 0.1	0.9	0.6
AF1317		hypothetical protein	X	-				0	0	0	0	0
AF1318		Argonaute homolog, implicated in RNA metabolism*	J	-				0	0	0	0	0
AF1319		uncharacterized conserved protein*	S	+			1.34	0.2	0.2	0.2	0.2	0.3
AF1320	guaA-2	GMP synthase subunit A	F	+				0.6	0.7	0.6 ± 0.1	0.5	0.6
AF1321		tRNA(Ile)-lysidine synthase MesJ*	D	-				0.7	0.8	0.7	0.7	0.7
AF1322		Predicted membrane-associated Zn-dependent protease*	M	-			1.25	0.9 ± 0.1	1.1	0.8	0.9	1.2
AF1323		group II decarboxylase	E	+				0.2	0.1	0.2	0.2	0.3
AF1324		carbohydrate kinase	G	-	**3.36**			0.2	0.1	0.2	0.6 ± 0.1	0.6 ± 0.1
AF1325		multidrug resistance protein	G	+	-1.43			0.1	0.1	0.1	0.1	0.1
AF1326		GTP-binding protein	R	-	**-1.95**			0.1	0.1	0.1	0	0
AF1327		hypothetical protein	X	-	**-1.70**			0.1	0.1	0.1	0.1	0.1
AF1328	glpA	glycerol-3-phosphate dehydrogenase	R	+				1.5 ± 0.1	1.5 ± 0.1	1.4 ± 0.1	1.5	1.4
AF1329		NAD(FAD)-dependent dehydrogenase*	R	+			-1.35	0.8 ± 0.1	0.6	0.8	0.7	0.5
AF1330	act-3	pyruvate formate-lyase activating enzyme	O	-				1.8	1.4 ± 0.1	2.1	2.1	2.3
AF1331		Galactose-1-phosphate uridylyltransferase*	C	-				1.2 ± 0.1	0.7	1.5 ± 0.1	1.4 ± 0.1	1.4 ± 0.1
AF1332		DNA replication initiation complex subunit, GINS15 family*	L	+				0.4	0.4	0.4 ± 0.1	0.4	0.4
AF1333	rpl44e	50S ribosomal protein L44e	J	+	-1.37			1.3 ± 0.1	1.2 ± 0.1	1.2 ± 0.1	0.9	0.8
AF1334	rps27e	30S ribosomal protein S27e	J	+	-1.44			1.8 ± 0.1	1.6 ± 0.1	1.7 ± 0.1	1.2 ± 0.1	1 ± 0.1
AF1335		hypothetical protein	X	-				0.1	0.1	0.1	0.1	0.1
AF1336	cobD	cobalamin biosynthesis protein	H	+				0.3 ± 0.1	0.3	0.2	0.2	0.3
AF1337		Archaea-specific RecJ-like exonuclease, contains DnaJ-type Zn finger domain*	L	+				1.1 ± 0.1	1.3 ± 0.1	1.2	1	1
AF1338	cbiP	cobyric acid synthase	H	-				0.7 ± 0.1	0.5	0.9 ± 0.1	0.6 ± 0.1	0.7
AF1339	rpl10e	50S ribosomal protein L10e	J	-				2.7	2.9	2.6 ± 0.2	2.6	2.5
AF1340	citZ	citrate synthase	C	-			-1.12	2.6	2.2	2.6	2.4	2.3
AF1341	deoA-1	thymidine phosphorylase	F	-			-1.42	2.8	1.7 ± 0.1	2.8	2.7	2.1
AF1342	deoA-2	thymidine phosphorylase	F	-			-1.33	2.4	1.5 ± 0.2	2.4	2.1	1.9 ± 0.1
AF1343		Metal-dependent hydrolase of the beta-lactamase superfamily II*	R	+				1.3 ± 0.1	1.2 ± 0.1	1.2 ± 0.1	1.4 ± 0.1	1.4 ± 0.1
AF1344		Sec-independent protein secretion pathway component TatC*	U	+	-1.29	-1.27		0.3	0.3	0.3	0.2	0.2
AF1345		uncharacterized conserved protein*	S	-	-1.41			0.3	0.3	0.3	0.2	0.2
AF1346m		Protein, predicted to be involved in DNA repair*	S	+		-1.42		0.3	0.3	0.2	0.2	0.2
AF1347m		Protein, predicted to be involved in DNA repair*	S	+				0.1	0.1	0.1	0.1	0.1
AF1348		Transcriptional regulator, ArsR family*	K	+				0.6 ± 0.1	1 ± 0.1	0.9 ± 0.1	0.7 ± 0.1	1.7 ± 0.1
AF1349	rd-2	rubredoxin	C	+				0.6 ± 0.1	1 ± 0.1	0.9 ± 0.1	0.6 ± 0.1	1.6 ± 0.2
AF1350		Metal-dependent hydrolase of the beta-lactamase superfamily II*	R	-				0.2	0.2	0.2	0.2	0.2
AF1351		ISA1083-2 transposase	L	+				0.1	0.1	0.1	0.1	0.1
AF1352		ISA1083-2 transposase	L	+				0.1	0.1	0.1	0.1	0.1
AF1353		uncharacterized conserved protein*	S	+				0.1	0.1	0.1	0.1	0.1
AF1354		Protein-disulfide isomerase*	O	+				1.3 ± 0.1	0.9 ± 0.1	1.2 ± 0.1	1.4 ± 0.1	1.5 ± 0.1
AF1355		Phosphate uptake regulator*	P	-				0.1	0.1	0.1	0.1	0.1
AF1356	phoX	phosphate ABC transporter, periplasmic phosphate-binding protein	P	+	**-3.37**			0.4 ± 0.1	0.6 ± 0.1	0.7 ± 0.1	0.1	0.2
AF1357	pstC	phosphate ABC transporter, permease protein	P	+	**-2.77**			0.4 ± 0.1	0.4 ± 0.1	0.6 ± 0.1	0.1	0.2
AF1358	pstA	phosphate ABC transporter, permease protein	P	+	**-2.60**			0.5 ± 0.1	0.7 ± 0.1	0.9 ± 0.1	0.2	0.3
AF1359	pstB	phosphate ABC transporter, ATP-binding protein	P	+	**-2.46**			0.3	0.4	0.5 ± 0.1	0.2	0.2
AF1360	phoU	phosphate ABC transporter, regulatory protein	P	+	**-1.90**			0.8	0.8	1	0.5	0.4
AF1361	arsC	arsenate reductase	T	+	-1.49			1	0.9	1.1 ± 0.1	0.7	0.6
AF1362		Predicted acyltransferase*	R	+				2.6 ± 0.2	2.5	2.6	2.5 ± 0.2	2.7
AF1363		hypothetical protein	X	+				2.2	2.3	2.2 ± 0.2	2.2 ± 0.2	2.4 ± 0.2
AF1364		translation-associated GTPase	J	+				1.6 ± 0.1	1.4 ± 0.1	1.5 ± 0.1	1.3 ± 0.1	1.3 ± 0.1

AF1365	hypE	hydrogenase expression/formation protein	O	-				0.6	0.5	0.6	0.6	0.6
AF1366	hypF	hydrogenase expression/formation regulatory protein	O	-				0.6	0.5	0.7	0.7	0.8
AF1367	hypA	hydrogenase expression/formation protein	R	+	**1.63**			0.5	0.3	0.5	0.8	0.8
AF1368	hypB	hydrogenase expression/formation protein	O	+	1.36			1.7 ± 0.1	1.6 ± 0.1	1.9	2.3	2.5
AF1369	hypC	hydrogenase expression/formation protein	O	+	1.37			1.5 ± 0.1	1.4 ± 0.1	1.6 ± 0.1	2.1	2.2
AF1370	hypD	hydrogenase expression/formation protein	O	+				1.2 ± 0.1	1.3 ± 0.1	1.3 ± 0.1	1.6 ± 0.1	1.7 ± 0.1
AF1371	vhtD-1	F420-nonreducing hydrogenase	C	-				0.2	0.1	0.2	0.3	0.3
AF1372	vhuA	methylviologen-reducing hydrogenase, subunit alpha	C	-	**2.61**			0.2	0.2	0.2	0.5 ± 0.1	0.6
AF1373	vhuG	methylviologen-reducing hydrogenase, subunit gamma	C	-	**3.55**			0.3	0.2	0.3	1 ± 0.1	1.1
AF1374	vhuD	methylviologen-reducing hydrogenase, subunit delta	C	-	**3.45**			0.1	0.1	0.1	0.3	0.3
AF1375	hdrB	heterodisulfide reductase, subunit B	C	-	**4.68**			0.1	0.2	0.1	0.6	0.9 ± 0.1
AF1376	hdrC	heterodisulfide reductase, subunit C	C	-	**6.30**			0.1	0.1	0.1	0.6	0.9 ± 0.1
AF1377	hdrA-2	heterodisulfide reductase, subunit A	C	-	**8.15**			0.1	0.1	0.1	0.5	0.7 ± 0.1
AF1378	vhtD-2	F420-nonreducing hydrogenase	C	-	**23.99**			0.1	0.1	0	1.4 ± 0.1	1.4 ± 0.1
AF1379	hydC	quinone-reactive Ni/Fe-hydrogenase B-type cytochrome subunit	C	-	**12.80**			0.2	0.3	0.2	**3.2**	**3.4**
AF1380	vhtA	F420-nonreducing hydrogenase	C	-	**18.66**			0.2	0.2	0.2	**3.4**	**3.3**
AF1381	vhtG	F420-nonreducing hydrogenase	C	-	**53.76**			0.1	0.1	0.1	**3.3**	**3.4**
AF1382		Predicted transcriptional regulator*	K	-	**1.69**			0	0	0	0	0.1
AF1383		ISA0963-4 transposase	L	-			1.27	0.5 ± 0.1	0.8	0.6	0.6	0.7
AF1384		response regulator	T	+				0.3	0.2	0.2	0.3	0.2
AF1385		PIN domain containing protein*	V	-				0.3	0.3	0.3	0.3	0.2
AF1386		hypothetical protein	X	+				0.2	0.2	0.2	0.2	0.2
AF1387		hypothetical protein	X	-				0.1	0	0.1	0.1	0.1
AF1388		DNA helicase, putative	L	+				0.6	0.7	0.6	0.6	0.5
AF1389	braG-4	branched-chain amino acid ABC transporter, ATP-binding protein	E	-				0.6	0.7	0.6	0.6	0.5
AF1390	braF-4	branched-chain amino acid ABC transporter, ATP-binding protein	E	-				2.6	2.7	2.8	2.9	2.9
AF1391	braC-4	branched-chain amino acid ABC transporter, periplasmic binding protein	E	-				**3.4**	**3.4**	**3.4**	**3.4**	**3.5**
AF1392	braD-4	branched-chain amino acid ABC transporter, permease protein	E	+			**-1.58**	0.9 ± 0.1	0.5	1	0.6 ± 0.1	0.5
AF1393	braE-4	branched-chain amino acid ABC transporter, permease protein	E	+				1.7 ± 0.1	1.3	1.9	1.5 ± 0.1	1.5 ± 0.1
AF1394		hypothetical protein	X	+				1.6 ± 0.2	1.6 ± 0.1	1.9 ± 0.1	1.5 ± 0.1	1.8 ± 0.1
AF1395		Dephospho-CoA kinase*	H	+				1.8 ± 0.2	1.8 ± 0.1	2.1	1.6 ± 0.1	1.5
AF1396		4-hydroxybenzoate synthetase (chorismate lyase)*	H	+				0.6 ± 0.1	0.5	0.7	0.4	0.3
AF1397m	potD	spermidine/putrescine ABC transporter, periplasmic spermidine/putrescine-binding protein	P	-				0.1	0.1	0.1	0.1	0.1
AF1398		PKD repeats containing protein*	S	-		**2.34**		0.8 ± 0.1	0.4	1.7 ± 0.4	1.8 ± 0.2	1.2 ± 0.1
AF1399		WD40-like repeat*	S	-		**2.66**		0.3 ± 0.1	0.2	0.8 ± 0.2	0.7 ± 0.1	0.8 ± 0.1
AF1400		Adenosylcobinamide amidohydrolase*	H	-				0.4 ± 0.1	0.4 ± 0.1	0.4 ± 0.1	0.3 ± 0.1	0.4
AF1401	hemV-3	iron ABC transporter, ATP-binding protein	P	-				1.2 ± 0.1	0.8 ± 0.1	1.3 ± 0.1	1.2 ± 0.1	1
AF1402	hemU-2	iron (III) ABC transporter, permease protein	P	-				0.7 ± 0.1	0.4 ± 0.1	0.9 ± 0.1	0.7 ± 0.1	0.6
AF1403		ACT domain-containing protein*	R	-				1.4 ± 0.2	1.4 ± 0.1	1.5 ± 0.1	1.1 ± 0.1	1.1
AF1404		AsnC family transcriptional regulator	K	-	-1.36			1.2 ± 0.2	1.2 ± 0.1	1.3 ± 0.1	0.9 ± 0.1	0.8
AF1405		Predicted RNA-binding protein (contains PuA domain)*	J	-	**-1.51**			1.3 ± 0.2	1.3 ± 0.1	1.4 ± 0.1	0.8 ± 0.1	0.8
AF1406		DEAD-box helicase, probable R-M system subunit*	V	-				0.5	0.7 ± 0.1	0.6 ± 0.1	0.6	0.7
AF1407		hypothetical protein	X	-				0	0	0	0	0.1
AF1408		hypothetical protein	X	-			**1.60**	0.1	0.1	0.1	0.1	0.1
AF1409		modification methylase, type III R/M system	L	-			1.19	0.8 ± 0.1	0.9	0.7 ± 0.1	0.7	0.8 ± 0.1
AF1411		Chromosome segregation ATPase*	D	-				1.7 ± 0.1	2	1.6 ± 0.1	1.7 ± 0.1	1.7
AF1412		S-layer protein*	S	-				0.3	0.2	0.2	0.2	0.1
AF1413	slgB-2	surface layer protein B	S	-			**-1.66**	0.9 ± 0.1	0.5	0.7	0.6	0.3
AF1414		dihydropteroate synthase	H	-				1.2 ± 0.1	1	1.2 ± 0.1	0.9 ± 0.1	0.8
AF1415		chloride channel, putative	P	-				1.5	1.2 ± 0.1	1.5 ± 0.1	1.2 ± 0.1	1
AF1416	ribC	riboflavin synthase	H	-		-1.34		1.1 ± 0.1	1.2 ± 0.1	0.9 ± 0.1	0.8 ± 0.1	0.8
AF1417	aspC	aspartate aminotransferase	E	-				2.6 ± 0.2	2.5	2.4	2.3	2.1
AF1418	taqD	glycerol-3-phosphate cytidyltransferase	M	-				2.8	2.8	2.8	2.6	2.6
AF1419	prsA-2	ribose-phosphate pyrophosphokinase	F	-				1.5 ± 0.1	1.4 ± 0.2	1.6 ± 0.1	1.2 ± 0.1	1.1 ± 0.1
AF1420		membrane protein	O	-		-1.17		2.1	2.1	1.8	1.7 ± 0.1	1.7 ± 0.1
AF1421		uncharacterized conserved protein*	S	-				1.7 ± 0.1	1.9 ± 0.1	1.5	1.6	1.8 ± 0.1
AF1422		aspartate racemase	M	-				1.2 ± 0.1	1 ± 0.1	1.3	1.7 ± 0.1	1.2
AF1423		HD superfamily phosphohydrolase*	R	-	-1.33			0.5	0.5	0.5	0.4	0.3
AF1424	pheT	phenylalanyl-tRNA synthetase subunit beta	J	+	-1.26			2.3	2.3	2.4	1.9 ± 0.1	1.6 ± 0.1
AF1425	bcpC-1	cofactor-independent phosphoglycerate mutase	G	+				2.6	2.3	2.7	2.3	1.8 ± 0.1
AF1426	glpF	glycerol uptake facilitator, MIP channel	G	-	**3.44**			0.3	0.4	0.5 ± 0.1	1.1 ± 0.1	1.6 ± 0.1
AF1427		uncharacterized conserved protein*	S	-	**13.84**			0.1	0.1	0	0.7 ± 0.1	1 ± 0.1
AF1428		uncharacterized conserved protein*	S	-	**10.02**			0.2	0.3	0.1	2.1	2.2
AF1429		Predicted transcriptional regulator, C-terminal HTH-like domain*	K	-	**3.34**			0.1	0.1	0.1	0.2	0.2
AF1430	rpl40e	50S ribosomal protein L40e	J	+	**-1.80**			0.4	0.4	0.4	0.3	0.2
AF1431		PIN-domain and Zn ribbon*	R	-				0.3	0.3	0.3	0.2	0.2
AF1432		Predicted hydrolase of HD superfamily*	R	-				0.1	0.1	0.1	0.1	0.1
AF1433		Endonuclease V homolog*	L	+			**1.51**	0.2	0.3	0.2	0.2	0.3
AF1434		hypothetical protein	X	+				0.5	0.4	0.4	0.4 ± 0.1	0.4
AF1435		RNA 3'-terminal-phosphate cyclase	J	+				0.4	0.4	0.4	0.4	0.2
AF1436	isf-1	iron-sulfur flavoprotein	R	-				0.7	1 ± 0.1	0.6 ± 0.1	0.8 ± 0.1	0.8 ± 0.1
AF1437		HAD superfamily hydrolase*	R	-				1	1.2	0.8 ± 0.1	0.7 ± 0.1	0.7
AF1438		Predicted Fe-S-cluster oxidoreductase*	R	-				0.3 ± 0.1	0.3 ± 0.1	0.3	0.2 ± 0.1	0.2
AF1439	asnB	asparagine synthetase	E	-				1 ± 0.2	0.9 ± 0.1	1 ± 0.1	0.7 ± 0.1	0.6
AF1440	gatB-1	glutamyl-tRNA(Gln) amidotransferase subunit E	J	-	-1.16			2	2.4	2.3	1.8 ± 0.2	1.9 ± 0.1
AF1441		hypothetical protein	X	-				2.9	**3**	**3.1**	2.9	**3**
AF1442		Bifunctional fructose-1,6-bisphosphate aldolase/phosphatase FBPA/FBPase*	G	-				2.8	2.9	**3**	2.8	2.6
AF1443		RecA-superfamily ATPase implicated in signal transduction*	T	+				1.6 ± 0.1	1.5	1.7 ± 0.1	1.7 ± 0.1	1.7 ± 0.1
AF1444		uncharacterized protein containing a von Willebrand factor type A (vWA) domain*	R	-	**1.63**			0.9 ± 0.1	0.9 ± 0.1	1	1.6	1.5 ± 0.1
AF1445		AAA family ATPase*	R	-	**1.74**			1.1 ± 0.1	1.3 ± 0.1	1.1 ± 0.1	2.1	2.1
AF1446		uncharacterized conserved protein*	S	+				0.2	0.3	0.3	0.2	0.4
AF1447		hypothetical protein	X	+				0.1	0.1	0.1	0.1	0.1
AF1448		AsnC family transcriptional regulator	P	+				0.2	0.1	0.2	0.1	0.1
AF1449	pflD	pyruvate formate-lyase 2	C	+				0.4 ± 0.1	0.3	0.5 ± 0.1	0.3	0.4
AF1450	pflC	pyruvate formate-lyase 2 activating enzyme	O	+				0.2	0.1	0.2	0.1	0.1
AF1451	thsB	thermosome, subunit beta	O	+	-1.25			**3.1**	**3**	**3.2**	2.5	2.4 ± 0.2
AF1452		signal-transducing histidine kinase	T	+	-1.40			0.6	0.7	0.6	0.4	0.4
AF1453	metG	methionyl-tRNA synthetase	J	+				2.1	2 ± 0.1	1.9 ± 0.1	1.7 ± 0.1	1.2
AF1454		Predicted nucleic acid binding protein containing the AN1-type Zn-finger*	R	-				0.5 ± 0.1	0.3	0.4 ± 0.1	0.4 ± 0.1	0.5 ± 0.1
AF1455		hypothetical protein	X	-				1.3 ± 0.2	0.8 ± 0.1	1.1 ± 0.2	1.1 ± 0.2	1.4 ± 0.2
AF1456		hypothetical protein	X	-				0.2	0.1	0.2	0.1	0.1
AF1457		uncharacterized conserved protein*	S	-			**1.57**	0.5 ± 0.1	0.8	0.4	0.6 ± 0.1	0.9 ± 0.1
AF1458		Hef nuclease	L	+				0.7 ± 0.1	0.5	0.7 ± 0.1	0.8	0.6
AF1459		Predicted transcriptional regulator*	K	-		-1.40		0.3	0.3	0.2	0.2	0.2
AF1460		hypothetical protein	X	-	-1.45	-1.47		0.2	0.2	0.1	0.1	0.1
AF1461		iron-sulfur cluster binding protein, putative	C	+				0.1	0.1	0	0	0
AF1462		hypothetical protein	X	+				0.1	0.1	0.1	0.1	0
AF1463	fdrA	fumarate reductase, flavoprotein subunit	C	+	-1.38			0.1	0.1	0.1	0.1	0
AF1464		uncharacterized conserved protein*	S	+				0.8 ± 0.1	1 ± 0.1	0.7 ± 0.1	0.5	0.3
AF1465		uncharacterized conserved protein*	S	+				0.4	0.6 ± 0.1	0.3	0.2	0.2
AF1466		hypothetical protein	X	+				0.1	0.1	0.1	0.1	0.1
AF1467		signal-transducing histidine kinase	T	+	-1.43			0.1	0.1	0.1	0.1	0.1
AF1468		S-layer domain*	M	+		**-2.51**		0.2	0.2	0.1	0.1	0.1

AF1469		ABC transporter, ATP-binding protein	V	+		**-2.77**		0.3	0.4 ± 0.1	0.2	0.1	0.1
AF1470		ABC-type antimicrobial peptide transport system, permease component*	V	+	**-2.25**	**-2.00**		0.2	0.3	0.2	0.1	0.1
AF1471		Na+-driven multidrug efflux pump*	V	+				0.4	0.4	0.4	0.4	0.3
AF1472		signal-transducing histidine kinase	T	-	**2.12**			0.4	0.4	0.5	0.9 ± 0.1	1.1
AF1473		response regulator	T	-	**2.18**			0.1	0.1	0.1	0.2	0.3
AF1474		uncharacterized conserved protein*	S	-				0.2	0.3	0.3	0.3	0.6
AF1475		mitochondrial benzodiazepine receptor/sensory transduction protein	T	-				0.2	0.2	0.2	0.3	0.3
AF1476		Lactoylglutathione lyase or related enzyme*	E	+				0.1	0.1	0.1	0.1	0.1
AF1477		uncharacterized conserved protein*	S	+				0.1	0.1	0.1	0.1	0.1
AF1478		Predicted antitoxin, copG family*	V	+				0.1	0.2	0.1	0.1	0.2
AF1479		PIN domain containing protein*	V	+				0.1	0.1	0.1	0.1	0.1
AF1480		PIN domain containing protein*	V	-	1.24			0.1	0.1	0.1	0.1	0.1
AF1481		Predicted antitoxin, copG family*	V	-				0	0	0	0	0
AF1482		hypothetical protein	X	-				0.1	0.1	0.1	0.1	0.1
AF1483		signal-transducing histidine kinase	T	-	**3.01**			0.1	0.2	0.2	0.5	0.6
AF1484		RecA-superfamily ATPase implicated in signal transduction*	T	-	**2.71**			0	0	0	0.1	0.1
AF1485	tgtB	queuine tRNA-ribosyltransferase	J	+				0.3	0.4	0.3	0.3	0.2
AF1486		phosphoenolpyruvate carboxylase	G	-	-1.41			0.1	0.1	0.1	0.1	0.1
AF1487		uncharacterized conserved protein*	S	-				0.5 ± 0.1	0.4	0.4 ± 0.1	0.3	0.2
AF1488		nicotinamide-nucleotide adenylyltransferase	H	-	**-1.56**			1.2 ± 0.1	0.9	1.2 ± 0.1	0.8 ± 0.1	0.5
AF1489	iorA	indolepyruvate ferredoxin oxidoreductase, subunit alpha	C	-	-1.17			2.9	3.1	2.9	2.5	2.5
AF1490	rpl1P	50S ribosomal protein L1P	J	+				3.3	3.5	3.4	3.4	3.3
AF1491	rplP0	acidic ribosomal protein P0	J	+				3.2	3.4	3.2	3.1	3.2
AF1492	rpl12p	50S ribosomal protein L12P	J	+				3.1	3.2	2.9 ± 0.2	2.7	3.1
AF1493	hpyA1-2	histone A1	L	+				1.2 ± 0.1	1	1 ± 0.2	1.1 ± 0.1	1
AF1494		Thermophilic glucose-6-phosphate isomerase or related metalloenzyme*	G	-				0.8 ± 0.1	0.8 ± 0.1	0.9 ± 0.1	1.1 ± 0.1	0.8 ± 0.1
AF1495		SHS2 domain protein implicated in nucleic acid metabolism*	R	+				0.7	0.7	0.7	0.7	0.7
AF1496		uncharacterized conserved protein*	S	+				1.6 ± 0.1	1.7	1.7 ± 0.1	1.7 ± 0.1	1.6
AF1497	aroA	3-phosphoshikimate 1-carboxyvinyltransferase	E	+				0.4	0.5	0.4	0.4	0.4
AF1498		dehydrase, putative	R	-				0.1	0.4	0.1	0.5	0.4
AF1499		hypothetical protein	X	+				0.1	0.1	0.1	0.1	0
AF1500		Roadblock/LC7 domain*	R	+				0.5 ± 0.1	0.3	0.6 ± 0.1	0.6	0.5
AF1501		uncharacterized conserved protein*	S	+				1.2 ± 0.1	1.1 ± 0.1	1.3 ± 0.1	1.3 ± 0.1	1.3
AF1502		hypothetical protein	X	-	1.35			1.3 ± 0.1	1.5 ± 0.2	1.4 ± 0.2	2.2 ± 0.3	2 ± 0.3
AF1503		HAMP domain*	T	-				3	3.1	3.1 ± 0.2	3.4	3.4
AF1504		hypothetical protein	X	-				1.8 ± 0.3	1.8 ± 0.1	2 ± 0.3	2.4 ± 0.2	2.2
AF1505		Permease, similar to cation transporter*	P	-			-1.31	0.7	0.5	0.6	0.6 ± 0.1	0.5
AF1506	asd	aspartate-semialdehyde dehydrogenase	E	-				2.3	2.4	2.1	2	1.8 ± 0.1
AF1507		uncharacterized conserved protein*	S	-				0.9	1.2 ± 0.1	1.1 ± 0.1	1.1 ± 0.1	1.4 ± 0.1
AF1508		Predicted permease*	R	-	**8.26**			0.1	0.2	0.1	1 ± 0.1	1.5 ± 0.1
AF1509		Metal-dependent hydrolase of the beta-lactamase superfamily II*	R	-				1.1 ± 0.1	2.2	1 ± 0.1	2 ± 0.1	2.1 ± 0.2
AF1510	fadD-6	acyl-CoA synthetase	I	-				1.9 ± 0.1	2.9	1.9 ± 0.1	2.9	3
AF1511		Acetyltransferase (GNAT) family*	K	-				2.6	3.4	2.6	3.3	3.3
AF1512		Sec-independent protein secretion pathway component TatC*	U	-				0.3	0.2	0.2	0.2	0.2
AF1513		uncharacterized distant relative of cell wall-associated hydrolase*	S	-	**6.35**			0.1	0.3	0.1	0.9 ± 0.1	1.1
AF1514		uncharacterized conserved protein*	S	-				0	0.1	0.1	0.1	0.1
AF1515		signal-transducing histidine kinase	T	+	**-1.53**	-1.47		0.1	0.1	0.1	0.1	0.1
AF1516	sfsA	sugar fermentation stimulation protein A	R	-				0.1	0.1	0.2	0.1	0.2
AF1517		Acetyltransferase (GNAT) family*	K	-				0.3 ± 0.1	0.3	0.5 ± 0.1	0.3	0.5
AF1518		DNA/pantothenate metabolism flavoprotein, putative	C	-				1.9 ± 0.2	1.7 ± 0.1	2.4	2 ± 0.2	2.2 ± 0.2
AF1519	isf-2	iron-sulfur flavoprotein	R	-				2.8	2.6	3.2	3	3.1
AF1520	fprA-2	flavoprotein	C	-				1.8 ± 0.2	1.5	2.3	1.8 ± 0.1	1.8 ± 0.1
AF1521		Predicted phosphatase homologous to the C-terminal domain of histone macroH2A1*	R	+	-1.36			0.9 ± 0.1	0.8 ± 0.1	0.9 ± 0.1	0.7	0.6
AF1522		Predicted P-loop ATPase fused to an acetyltransferase*	R	+				1.4 ± 0.1	1.5 ± 0.1	1.5	1.2 ± 0.1	1.1 ± 0.1
AF1523		ASCH domain, predicted RNA-binding domain*	S	+			**-1.64**	0.9 ± 0.1	0.6	1.1	0.7 ± 0.1	0.5
AF1524		hypothetical protein	X	-				0.8 ± 0.1	0.4	1 ± 0.1	0.8	0.7
AF1525		Predicted permease*	R	-	**2.57**			0.3	0.3	0.4	0.8 ± 0.1	1 ± 0.1
AF1526		Nucleotide-binding protein, uspA family*	T	-	**5.33**			0.1	0.3	0.2	1	1.2 ± 0.1
AF1527		uncharacterized conserved protein*	S	-	**5.75**			0.1	0.5 ± 0.1	0.1	1.3 ± 0.1	1.5
AF1528		uncharacterized conserved protein*	S	-				0.2	0.2	0.2	0.2	0.3 ± 0.1
AF1529	rpl21E	50S ribosomal protein L21e	J	+	-1.18			1.6 ± 0.1	1.7 ± 0.1	1.7 ± 0.1	1.4 ± 0.1	1.4
AF1530		DNA-directed RNA polymerase, subunit F (rpoF)*	K	+				1.4 ± 0.1	1.2	1.6 ± 0.1	1.1 ± 0.1	0.9
AF1531		Predicted RNA-binding protein*	J	+				1.4 ± 0.1	1.3 ± 0.1	1.5 ± 0.1	1.1 ± 0.1	1.2
AF1532		Protein implicated in RNA metabolism, contains PRC-barrel domain*	R	+				1 ± 0.1	0.7	1.2	1.1	0.9
AF1533		permease of the drug/metabolite transporter (DMT) superfamily*	G	-			**-1.51**	0.2	0.1	0.2	0.2	0.1
AF1534		Putative sterol carrier protein*	I	+			**1.76**	0.3	0.7 ± 0.1	0.4	0.5 ± 0.1	0.8 ± 0.1
AF1535	ftrB	ferredoxin-thioredoxin reductase, catalytic subunit	C	-				0.5 ± 0.1	0.2	1.4 ± 0.1	0.7 ± 0.1	0.7
AF1536	grx-1	glutaredoxin	O	-				0.5 ± 0.1	0.2	1.1 ± 0.1	0.5 ± 0.1	0.6
AF1537	est-2	carboxylesterase	R	-	**1.83**			0.1	0.1	0.1	0.2	0.2
AF1538		Short chain fatty acids transporter*	I	-	**8.01**			0.1	0.1	0.1	0.6 ± 0.1	0.7 ± 0.1
AF1539	sucD-1	succinyl-CoA synthetase, alpha subunit	C	-				0.4 ± 0.1	0.6	0.5 ± 0.1	0.5 ± 0.1	0.3
AF1540	sucC	succinyl-CoA synthetase, beta subunit	C	-				0.6 ± 0.1	1.1	0.9 ± 0.1	0.9 ± 0.1	0.7
AF1541		Permease of the major facilitator superfamily*	G	-				0.6 ± 0.1	0.5	0.7 ± 0.2	0.4 ± 0.1	0.5 ± 0.1
AF1542		Predicted transcriptional regulator, PadR family*	K	-				0.2	0.2	0.3 ± 0.1	0.2	0.2
AF1543		Class II terpene cyclase family protein*	I	+				0.1	0.2	0.2	0.1	0.1
AF1544		ArsR family transcriptional regulator	K	+				0.1	0.1	0.1	0.1	0.1
AF1545		uncharacterized conserved protein*	S	+				1 ± 0.1	0.9 ± 0.1	1 ± 0.1	1 ± 0.1	1.2 ± 0.1
AF1546		Small-conductance mechanosensitive channel*	M	+				1 ± 0.1	0.9	1	1.1 ± 0.1	1.1 ± 0.1
AF1547		hypothetical protein	X	+				0.8 ± 0.1	0.7	0.8 ± 0.1	0.9 ± 0.1	1.1
AF1548		Restriction endonuclease*	V	+				0.3	0.3	0.3	0.4	0.5
AF1549		uncharacterized conserved protein*	S	-				0.2	0.3	0.2	0.2	0.3 ± 0.1
AF1550		Histidinol phosphatase or related hydrolase of the PHP family*	E	+				0.2	0.3	0.2	0.2	0.2
AF1551	ispB	octaprenyl-diphosphate synthase	H	-				0.3	0.3	0.2	0.2	0.3
AF1552		Permease of the drug/metabolite transporter (DMT) superfamily*	G	-				0.1	0.1	0.1	0.1	0.1
AF1553		RNA:NAD 2'-phosphotransferase*	J	+			1.25	0.8 ± 0.1	1.1 ± 0.1	0.9	0.8 ± 0.1	1.1 ± 0.1
AF1554	trxB	thioredoxin reductase	O	+				1.2	1.6 ± 0.1	1.5	1.2 ± 0.1	1.5
AF1555		hypothetical protein	X	+				0.1	0.1	0.1	0.1	0.1
AF1556		ATP-utilizing enzyme of the PP-loop superfamily*	R	+				0.1	0.1	0.1	0.1	0.1
AF1557		uncharacterized conserved protein*	S	+			1.14	2.1	2.4	2	2.1	2.5
AF1558	smc1	chromosome segregation protein	D	+			1.11	2 ± 0.1	2.1	1.9 ± 0.1	1.9 ± 0.1	2.2
AF1559		Rec8/ScpA/Scc1-like protein (kleisin family)*	L	+			**1.76**	0.4 ± 0.1	0.6	0.3 ± 0.1	0.3 ± 0.1	0.6
AF1560		hypothetical protein	X	+				0.2	0.1	0.2	0.1	0.1
AF1561		Predicted permease*	R	+				3.4	3.3 ± 0.2	3.3	3.1	3.2 ± 0.2
AF1562		uncharacterized conserved protein*	S	+	-1.17			3.2	3	3	2.5	2.7
AF1563		Alpha/beta superfamily hydrolase*	R	-				0.8	0.4	0.8 ± 0.1	0.4	0.4
AF1564		Predicted transcriptional regulator, containd two HTH domains*	K	-				0.3 ± 0.1	0.2	0.4	0.3	0.3
AF1565		uncharacterized conserved protein*	S	-				0.3	0.2	0.3	0.3	0.2
AF1566		uncharacterized conserved protein*	S	+				2.9	3	2.9	2.8	2.8
AF1567		uncharacterized conserved protein*	S	+				2.1	2	2.1	1.8 ± 0.1	1.6 ± 0.1
AF1568		Predicted metal-binding protein*	S	+				1.5	2	1.4 ± 0.1	1.2 ± 0.1	1.6 ± 0.1
AF1569		hypothetical protein	X	+				0.6 ± 0.1	0.7	0.4 ± 0.1	0.4 ± 0.1	0.7 ± 0.1
AF1570		Predicted metal-binding protein*	S	+			**1.57**	0.5 ± 0.1	0.7	0.3	0.3 ± 0.1	0.6 ± 0.1
AF1571		hypothetical protein	X	+				0.5 ± 0.1	0.5 ± 0.1	0.4	0.3	0.4 ± 0.1

AF1572		hypothetical protein	X	+				0.1	0.2	0.1	0.1	0.1
AF1573		uncharacterized conserved protein*	S	+	-1.48			1.6 ± 0.1	1.4	1.5 ± 0.1	1.1 ± 0.2	0.8 ± 0.1
AF1574		uncharacterized conserved protein*	S	+	-1.17			2.8	2.8	2.7	2.3 ± 0.2	2.2
AF1575		uncharacterized conserved protein*	S	+				1.5 ± 0.2	1.9 ± 0.1	1.4 ± 0.1	0.9 ± 0.1	1.1 ± 0.1
AF1576		hypothetical protein	X	+				0.5 ± 0.1	0.3	0.6 ± 0.1	0.2	0.1
AF1577		hypothetical protein	X	+				0.2	0.1	0.2	0.1	0.1
AF1578		hypothetical protein	X	+				0.2	0.1	0.1	0.1	0.1
AF1579		hypothetical protein	X	+				0.6 ± 0.1	0.4	0.6 ± 0.1	0.4 ± 0.1	0.2
AF1580		Chromosome segregation and condensation protein B*	K	-				0.1	0.1	0.1	0.1	0.1
AF1581		hypothetical protein	X	+			-1.21	0	0	0	0	0
AF1582		hypothetical protein	X	-			-1.26	0.1	0.1	0.1	0.1	0.1
AF1583		DNA repair photolyase*	L	-				0.1	0.1	0.1	0.1	0.1
AF1584		uncharacterized conserved protein*	S	-				0	0	0	0	0
AF1585		HEPN domain containing protein*	V	+			-1.21	0.2	0.2	0.2	0.2	0.2
AF1586		Minimal nucleotidyltransferase*	V	+			1.43	0	0.1	0	0	0.1
AF1587	rbcL-1	ribulose bisphosphate carboxylase, large subunit	G	-	**6.09**			0.2	0.4	0.2	1.7 ± 0.2	1.6 ± 0.1
AF1588		Saccharopine dehydrogenase or related enzyme*	E	-	**5.04**			0.2	0.4	0.2	1.3 ± 0.2	1.4 ± 0.1
AF1589		DNA repair photolyase*	L	-				0.4	0.3	0.4	0.4 ± 0.1	0.3
AF1590		Protein, predicted to be involved in DNA repair*	S	-				0.9	1	0.9	1 ± 0.1	0.9 ± 0.1
AF1591		Predicted antitoxin, copG family*	V	+				0.2	0.2	0.2	0.2	0.2
AF1592		Siroheme synthase (precorrin-2 oxidase/ferrochelatase domain)*	H	+				0.6 ± 0.1	0.7	0.6 ± 0.1	0.6	0.8
AF1593m	nirD	heme d1 biosynthesis protein	K	+			1.38	0.9 ± 0.1	1.3 ± 0.1	0.9 ± 0.1	1 ± 0.1	1.4
AF1594m	nirH	heme biosynthesis protein	K	+			1.24	0.8 ± 0.1	1.2	0.9	0.9 ± 0.1	1
AF1595		tRNA(Ile)-lysidine synthase MesJ*	D	+				0.4 ± 0.1	0.5	0.4	0.4 ± 0.1	0.5
AF1596		Transcriptional regulator, MarR family*	K	-				0.1	0.1	0.1	0.2	0.1
AF1597		hypothetical protein	X	-				0.4 ± 0.1	0.3	0.4 ± 0.1	0.3 ± 0.1	0.2
AF1598		uncharacterized conserved protein*	S	-	-1.49	-1.44		0.9 ± 0.1	0.8	0.6 ± 0.1	0.5 ± 0.1	0.5 ± 0.1
AF1599	trpA	tryptophan synthase subunit alpha	E	-	1.36			1.6 ± 0.2	1.9 ± 0.1	1.6 ± 0.2	2.6 ± 0.3	2.4 ± 0.2
AF1600	trpB-2	tryptophan synthase subunit beta	E	-				2.1	2.1	2.1 ± 0.2	2.8 ± 0.2	2.6 ± 0.3
AF1601	trpF	N-(5'-phosphoribosyl)anthranilate isomerase	E	-				**3**	2.8	**3.1**	**3.4**	**3.3**
AF1602	trpG	anthranilate synthase component II	E	-				2.1	2	2.3 ± 0.2	2.7 ± 0.2	2.6 ± 0.2
AF1603	trpE	anthranilate synthase component I	E	-	1.14			2.4	2.6	2.5	**3 ± 0.2**	**3**
AF1604	trpD	anthranilate synthase component II	E	-	1.10			2.8 ± 0.2	2.9	2.9	**3.3**	**3.2**
AF1605m	potD	spermidine/putrescine ABC transporter, periplasmic spermidine/putrescine-binding protein	E	-				1.7 ± 0.2	1.9 ± 0.1	1.7 ± 0.3	1.5 ± 0.2	1.1 ± 0.1
AF1606	potC	spermidine/putrescine ABC transporter, permease protein	E	-				0.3	0.4 ± 0.1	0.3	0.4	0.3
AF1607	potB	spermidine/putrescine ABC transporter, permease protein	E	-				1.3 ± 0.1	1.4 ± 0.2	1.3 ± 0.1	1.7 ± 0.1	1.5 ± 0.1
AF1608	potA	spermidine/putrescine ABC transporter, ATP-binding protein	E	-				1.5 ± 0.1	1.7 ± 0.1	1.3 ± 0.1	1.6 ± 0.1	1.2 ± 0.1
AF1609	proS	prolyl-tRNA synthetase	J	+				2.2	2	2.1	2.1	1.4 ± 0.1
AF1610		S-adenosylmethionine decarboxylase proenzyme	E	+				1.9 ± 0.4	2.2 ± 0.2	1.8 ± 0.3	1.5 ± 0.2	1.1 ± 0.1
AF1611		Predicted methyltransferase*	R	+				1.6 ± 0.3	1.9 ± 0.1	1.5 ± 0.1	1.3 ± 0.1	1.1
AF1612	cat-1	cationic amino acid transporter	E	+			-1.34	1.1 ± 0.1	0.8	1	0.9	0.6
AF1613		Minimal nucleotidyltransferase*	V	-				0.3	0.4	0.4	0.4	0.5 ± 0.1
AF1614		HEPN domain containing protein*	V	-				0.2	0.2	0.2	0.2	0.3
AF1615	cmo	cofactor modifying protein	R	+				0.1	0.2	0.2	0.1	0.1
AF1616		uncharacterized conserved protein*	S	-		**1.62**		0.2	0.4	0.5	0.5	0.5
AF1617		hypothetical protein	X	-				2.2 ± 0.3	2.9 ± 0.2	**3**	**3.3**	**3.3**
AF1618		Predicted membrane protein*	S	-		1.07		**3.3**	**3.4**	**3.6**	**3.7**	**3.6**
AF1619		Predicted membrane protein*	S	-				**3.1**	**3.4**	**3.5**	**3.6**	**3.6**
AF1620		signal-transducing histidine kinase, putative	T	-				2	2.8	2.1	2.3	2.4 ± 0.2
AF1621		Predicted membrane protein*	S	-				0.5 ± 0.1	0.8	0.4	0.6 ± 0.1	1.2 ± 0.2
AF1622	lrp	leucine responsive regulatory protein	K	+			-1.29	1.6 ± 0.1	1.1	1.6 ± 0.1	1.4 ± 0.1	1.3
AF1623	aspB-3	aspartate aminotransferase	E	+				1.6 ± 0.1	1.1	1.5 ± 0.1	1.4 ± 0.1	1.4
AF1624	moaD	molybdopterin converting factor, subunit 1	H	+			**-1.67**	0.5 ± 0.2	0.2	0.4 ± 0.1	0.4 ± 0.1	0.2
AF1625		uncharacterized conserved protein*	S	+				0.2	0.2	0.2	0.2	0.5 ± 0.1
AF1626		Predicted membrane protein*	S	+				0.3 ± 0.1	0.3	0.3	0.2	0.2
AF1627		repressor protein	K	+				0.3 ± 0.1	0.4 ± 0.1	0.4 ± 0.1	0.3	0.2
AF1628		transposase, putative	L	+				0	0	0	0	0
AF1629		uncharacterized conserved protein*	S	+				0.1	0.1	0.1	0.1	0.1
AF1630		pyruvoyl-dependent arginine decarboxylase	E	-				0.4 ± 0.1	0.3	0.4	0.3	0.2
AF1631		hypothetical protein	X	-				0.4 ± 0.1	0.3	0.4	0.2	0.3
AF1632		Predicted permease*	R	+			-1.12	0	0	0	0.1	0
AF1633		hypothetical protein	X	+				0.4 ± 0.1	0.3	0.4	0.3	0.2
AF1634m		Fe-S oxidoreductase	C	-				2	2.3	2 ± 0.1	1.9 ± 0.1	2
AF1636		uncharacterized conserved protein*	S	-				1.4 ± 0.1	1.5 ± 0.1	1.3 ± 0.1	1.2 ± 0.1	1.1 ± 0.1
AF1637	chlP-3	bacteriochlorophyll synthase, 43 kDa subunit	C	+				0.4	0.3	0.4	0.3	0.3
AF1638	rbcL	ribulose bisphosphate carboxylase	G	+				2.1	2.5	1.9 ± 0.1	1.8 ± 0.1	1.8
AF1639		signal-transducing histidine kinase	T	+				0.3	0.2	0.3	0.3	0.3
AF1640	rr3	rubrerythrin	C	+				0.1	0.2	0.1	0.3	0.2
AF1641	fad-4	enoyl-CoA hydratase	I	-	**4.36**			0.1	0.1	0.1	0.4	0.4
AF1642	hisS	histidyl-tRNA synthetase	J	-				0.7 ± 0.1	1.1	0.7	0.6 ± 0.1	0.6
AF1643		3-polyprenyl-4-hydroxybenzoate decarboxylase or related decarboxylase*	H	+			**-1.52**	2.2	1.2 ± 0.1	2.1	2	1.5 ± 0.1
AF1644	fwdF	tungsten formylmethanofuran dehydrogenase, subunit F	C	+				2.1	1.7 ± 0.1	2	2	2 ± 0.1
AF1645	dfp	bifunctional phosphopantothenoylcysteine decarboxylase/phosphopantothenate synthase	H	+				2.2	2.3	2.1	2 ± 0.1	2.1
AF1646		Pantoate kinase*	H	+				1.5 ± 0.2	1.6 ± 0.1	1.5 ± 0.1	1.3 ± 0.1	1.5 ± 0.1
AF1647		Predicted amidohydrolase*	R	+				0.8 ± 0.1	0.9 ± 0.1	0.8 ± 0.1	0.6 ± 0.1	0.7
AF1648		bacteriochlorophyll synthase, 33 kDa subunit	H	+	**1.75**			0.2	0.2	0.2	0.5	0.3
AF1649	fwdG	tungsten formylmethanofuran dehydrogenase, subunit G	C	+				1.2 ± 0.2	1.4 ± 0.2	2.1	1.6 ± 0.1	2.1 ± 0.2
AF1650	fwdB-1	tungsten formylmethanofuran dehydrogenase, subunit B	C	+				1.6 ± 0.2	2 ± 0.2	2.5	2.1 ± 0.2	2.7
AF1651	fwdD-1	tungsten formylmethanofuran dehydrogenase, subunit D	C	+				0.5 ± 0.1	0.7 ± 0.1	1 ± 0.1	0.7 ± 0.1	0.9 ± 0.1
AF1652		prepro-subtilisin sendai, putative	O	-				0.6 ± 0.1	1.4 ± 0.1	0.6 ± 0.1	1 ± 0.2	0.8 ± 0.1
AF1653	aprM	alkaline serine protease	O	-			**4.90**	0.2	1.6 ± 0.2	0.2	0.6 ± 0.3	1.1 ± 0.1
AF1654		uncharacterized conserved protein*	S	-	1.44			0.1	0.1	0.1	0.1	0.1
AF1655		signal sequence peptidase, putative	U	-				0.1	0.1	0.1	0.2	0.1
AF1656		hypothetical protein	X	-	**1.65**			0	0	0	0.1	0.1
AF1657	spc21	signal sequence peptidase	U	-			-1.25	0.1	0	0	0.1	0
AF1658		uncharacterized conserved protein*	S	-				0.1	0.1	0.1	0.1	0.1
AF1659		hypothetical protein	X	-				1.5 ± 0.2	1.6 ± 0.1	1.7 ± 0.1	1.6 ± 0.1	1.1 ± 0.1
AF1660		uncharacterized conserved protein*	S	-				0.3 ± 0.1	0.3	0.3	0.3	0.1
AF1661		uncharacterized conserved protein*	S	-				0.5 ± 0.1	0.6	0.5	0.5	0.3
AF1662		hypothetical protein	X	-				0	0	0	0	0
AF1663		Predicted transcriptional regulator*	K	-			1.43	0	0.1	0	0	0.1
AF1664	nrd	ribonucleotide-diphosphate reductase subunit alpha	F	-				1.7 ± 0.1	2	1.8 ± 0.1	1.9	1.8 ± 0.1
AF1665	arcB	alanine dehydrogenase	E	+				1.6 ± 0.2	2.1	1.9 ± 0.1	1.8 ± 0.2	1.7 ± 0.1
AF1666		Predicted ATPase of PP-loop superfamily*	R	+			1.50	0.7 ± 0.1	1.4 ± 0.2	0.9	0.9 ± 0.1	1.1 ± 0.1
AF1667	sat	sulfate adenylyltransferase	P	+	-1.16			**3.6**	**3.6**	**3.6**	**3.2**	**3 ± 0.2**
AF1668		uncharacterized conserved protein*	S	+	**-1.51**			**3.1**	**3.2**	**3.1**	2.2	1.7
AF1669	aprB	adenylylsulfate reductase, subunit B	C	+				**3.5**	**3.6**	**3.5**	**3.4**	**3.2**
AF1670	aprA	adenylylsulfate reductase	C	+	-1.40			**3.6**	**3.5**	**3.6**	2.8	2.3
AF1671	ftsA-2	coenzyme F390 synthetase	H	+				**3.3**	**3.3**	**3.2**	**3.1**	2.9
AF1672		acetolactate synthase, small subunit, putative	R	+				1.4 ± 0.2	1.7 ± 0.1	1.3 ± 0.1	1.2 ± 0.1	1.2 ± 0.1
AF1673		potassium channel, putative	P	+				0.8 ± 0.1	0.7 ± 0.1	0.8	0.6 ± 0.1	0.6 ± 0.1
AF1674	gldA	sn-glycerol-1-phosphate dehydrogenase	C	-			-1.25	1.7 ± 0.1	1.3	1.8 ± 0.1	1.6 ± 0.1	1.3 ± 0.1
AF1675		hypothetical protein	X	-				0.2	0.1	0.3	0.3	0.2

AF1676		NAD-dependent deacetylase	K	+				0.6 ± 0.1	0.2	0.6 ± 0.1	0.9 ± 0.1	0.6 ± 0.1
AF1677		tRNA pseudouridine synthase D*	J	-				0.5	0.6	0.5	0.5	0.4
AF1678		Putative sterol carrier protein*	I	-	**1.99**			0.3	0.4	0.3	0.5	0.7
AF1679		vtpJ-therm, putative	X	+	1.30			0.2	0.2	0.2	0.2	0.2
AF1680		uncharacterized conserved protein*	S	-				0.2	0.1	0.2	0.2	0.2
AF1681		uncharacterized conserved protein*	S	-			-1.35	0.2	0.1	0.2	0.2	0.2
AF1682		hypothetical protein	X	+				0.1	0.1	0.1	0.1	0.1
AF1683		PIN domain containing protein*	V	+				0.3	0.2	0.2	0.2	0.2
AF1684		Radical SAM superfamily enzyme*	C	+				0.9 ± 0.1	0.8 ± 0.1	0.9 ± 0.1	0.9 ± 0.1	0.7 ± 0.1
AF1685	cooC-2	CODH nickel-insertion accessory protein	D	+	-1.35			1.1 ± 0.1	1.2 ± 0.1	1.3 ± 0.1	0.8	0.9
AF1686		Minimal nucleotidyltransferase*	V	+				1 ± 0.2	0.9	1.2 ± 0.1	0.7 ± 0.1	0.9 ± 0.1
AF1687		uncharacterized conserved protein*	S	+				1.8 ± 0.3	1.8 ± 0.1	2.3 ± 0.2	1.6 ± 0.2	2.2
AF1688		Predicted permease*	R	-				0.1	0.1	0.2	0.1	0.1
AF1689		Predicted antitoxin, copG family*	V	+				0.1	0.1	0.1	0.1	0.1
AF1690	vapC-2	virulence associated protein C	V	+				0.2	0.2	0.2	0.1	0.2
AF1691		permease of the drug/metabolite transporter (DMT) superfamily*	G	-				0.4	0.3	0.5	0.5	0.5
AF1692	nth	endonuclease III	L	-				0.4 ± 0.1	0.4	0.4	0.4 ± 0.1	0.4
AF1693	purM	phosphoribosylaminoimidazole synthetase	F	+				0.9 ± 0.1	0.8	0.8 ± 0.1	0.8	0.6
AF1694	trpS	tryptophanyl-tRNA synthetase	J	-				0.6	0.8	0.6 ± 0.1	0.5	0.5
AF1695	apbA	2-dehydropantoate 2-reductase	H	+				1.2 ± 0.1	1.7 ± 0.1	1.2 ± 0.1	1.1	1.1 ± 0.1
AF1696	qacE	small multidrug export protein	S	+				0.1	0.2	0.2	0.1	0.1
AF1697		Transcriptional regulator containing HTH domain, ArsR family*	K	+				0.1	0.1	0.1	0.1	0.1
AF1698		Phospholipid-binding protein*	R	+	**1.61**			0.1	0.1	0.1	0.1	0.1
AF1699	porG	pyruvate ferredoxin oxidoreductase subunit gamma	C	+	-1.18			**3.2**	**3.1**	**3.2**	2.7	2.5
AF1700	porD	pyruvate ferredoxin oxidoreductase, subunit delta	C	+				**3.3**	**3.1**	**3.2**	2.9	2.7
AF1701	porA	pyruvate ferredoxin oxidoreductase, subunit alpha	C	+				**3.5**	**3.4**	**3.5**	**3.4**	**3.4**
AF1702	porB	pyruvate ferredoxin oxidoreductase, subunit beta	C	+				**3.4**	**3.4**	**3.3**	**3.2**	**3.2 ± 0.2**
AF1703		Putative threonine efflux protein*	E	+				0.9 ± 0.1	0.8	0.9 ± 0.1	0.6 ± 0.1	0.7
AF1704		Predicted periplasmic solute-binding protein*	R	-	**-1.56**	-1.44		0.8	0.8	0.7	0.5	0.5
AF1706	pcbD	2-hydroxy-6-oxo-6-phenylhexa-2,4-dienoic acid hydrolase	R	-	**1.73**			0.1	0.1	0.1	0.2	0.1
AF1707		Predicted metal-dependent hydrolase*	R	-				0.2	0.2	0.2	0.1	0.2
AF1708		type I restriction-modification enzyme, R subunit	V	-				0.7 ± 0.1	0.7	0.7	0.5 ± 0.1	0.7
AF1709		HEPN domain containing protein*	V	-				0	0.1	0	0	0.1
AF1710		type I restriction-modification enzyme, S subunit	V	-				0.1	0.1	0.1	0.2	0.2
AF1711		PIN domain containing protein*	V	-				0.2	0.2	0.2	0.2	0.4 ± 0.1
AF1712		DNA-binding protein, potential antitoxin AbrB/MazE fold*	K	-			**2.19**	0.1	0.2	0.1	0.1	0.3
AF1713		hypothetical protein	X	-			**1.73**	0.1	0.2	0.1	0.1	0.2
AF1714		uncharacterized conserved protein*	S	-			1.48	0.1	0.1	0.1	0	0.1
AF1715m		type I restriction-modification enzyme, M subunit	V	-				0.2	0.3	0.2	0.2	0.2
AF1716	estA	carboxylesterase	I	+				2 ± 0.1	2.2	1.8 ± 0.2	1.9 ± 0.1	1.4 ± 0.1
AF1717		uncharacterized conserved protein*	S	-				0.4	0.4	0.5	0.3	0.4
AF1718		uncharacterized conserved protein*	S	-			-1.20	1.7 ± 0.1	1.3	1.7 ± 0.1	1.7 ± 0.1	1.5
AF1719	ilvH	acetolactate synthase 3 regulatory subunit	E	-				1.9 ± 0.1	1.4 ± 0.1	1.7 ± 0.1	1.7 ± 0.1	1.5 ± 0.1
AF1720	ilvB-1	acetolactate synthase 3 catalytic subunit	E	-				2.8	2.5	2.8	2.7	2.4
AF1721		signal-transducing histidine kinase	T	+				0.2	0.2	0.2	0.2	0.3
AF1722		DNA polymerase II large subunit	L	+	-1.32			1.2	1.2 ± 0.1	1.3	0.9	0.9 ± 0.1
AF1723		AsnC family transcriptional regulator	K	+				0.8 ± 0.1	1	0.8 ± 0.1	0.8 ± 0.1	1.1 ± 0.1
AF1724	draG	dinitrogenase reductase activating glycohydrolase	O	+				0.3	0.4	0.4	0.3	0.3
AF1725		DNA ligase, putative	L	-	**1.93**			0.2	0.4	0.2	0.5	0.6
AF1726		uncharacterized conserved protein*	S	-	**2.13**			0.2	0.3 ± 0.1	0.2	0.5 ± 0.1	0.5
AF1727	mae	malate oxidoreductase	C	+	**2.62**			0.7 ± 0.1	0.9 ± 0.1	0.3	1.6 ± 0.2	1.9 ± 0.1
AF1728		galactosyltransferase	M	+				0.1	0.1	0.1	0.1	0.1
AF1729		Cell division GTPase*	D	+				0.4	0.4	0.4	0.3	0.3
AF1730	truA	tRNA pseudouridine synthase A	J	-				1.2 ± 0.1	0.7	1.3 ± 0.1	0.9 ± 0.1	1 ± 0.1
AF1731		hypothetical protein	X	-				1.5	1	1.5	1.1	1
AF1732	gap	glyceraldehyde-3-phosphate dehydrogenase	G	-	-1.40			1.7 ± 0.1	1.9 ± 0.1	1.7 ± 0.1	1.3 ± 0.1	1.1
AF1733		hypothetical protein	X	-				1.4 ± 0.2	1.6 ± 0.1	1.5 ± 0.2	1.3 ± 0.1	1 ± 0.1
AF1734		ABC-type Na+ efflux pump, permease component*	C	+				0.4 ± 0.1	0.3	0.3	0.3	0.3
AF1735		ABC-type Na+ efflux pump, permease component*	C	+				0.4	0.3	0.2	0.3	0.2
AF1736	mvaA	3-hydroxy-3-methylglutaryl-coenzyme A reductase	I	-				0.9 ± 0.1	0.6	0.5	0.9 ± 0.1	0.6
AF1737		Highly conserved protein containing a thioredoxin domain*	O	-				0.2	0.1	0.4 ± 0.1	0.2	0.2
AF1738		Protoporphyrinogen oxidase*	H	+				0.3	0.3	0.3	0.3	0.3
AF1739		uncharacterized conserved protein*	S	-				0.5	0.2	0.5	0.3	0.2
AF1740		CDP-diglyceride synthetase*	I	+				0.2	0.2	0.2	0.2	0.1
AF1741	pyrE	orotate phosphoribosyl transferase	F	+				0.3	0.3	0.3	0.3	0.2
AF1742		Predicted transcriptional regulator*	K	-				0.4	0.6 ± 0.1	0.5	0.6	0.6
AF1743		AsnC family transcriptional regulator	P	+				1.1 ± 0.1	1.2	1.3 ± 0.1	1.2 ± 0.1	1.6
AF1744	pgsA-2	CDP-diacylglycerol--glycerol-3-phosphate 3-phosphatidyltransferase	I	+				0.4 ± 0.1	0.5	0.6 ± 0.1	0.4 ± 0.1	0.6
AF1745		Predicted antitoxin, copG family*	V	-				0.1	0.1	0.1	0	0
AF1746	amt-2	ammonium transporter	P	+				0.2	0.1	0.2	0.1	0.1
AF1747	glnB-2	nitrogen regulatory protein P-II	E	+				0.1	0.1	0.1	0.1	0.1
AF1748		Metal-dependent hydrolase of the beta-lactamase superfamily II*	R	+				0.1	0	0.1	0	0
AF1749	amt-3	ammonium transporter	P	+	-1.30			0.1	0.1	0.1	0.1	0.1
AF1750	glnB-3	nitrogen regulatory protein P-II	E	+				0.6	0.7 ± 0.1	0.6 ± 0.1	0.5	0.4
AF1751	bcpC-2	cofactor-independent phosphoglycerate mutase	G	-	**-2.35**			1.7 ± 0.1	1.5 ± 0.1	1.8 ± 0.1	0.8	0.6 ± 0.1
AF1752		carbohydrate kinase	G	-	**-2.05**			2 ± 0.1	1.4	2	0.9 ± 0.1	0.7
AF1753		lysophospholipase	I	-				1 ± 0.1	0.4	1.1 ± 0.1	0.5	0.4
AF1754		Branched-chain amino acid transport protein*	E	-				1.3 ± 0.2	0.7 ± 0.1	1.6 ± 0.2	0.7 ± 0.1	0.5
AF1755		Predicted branched-chain amino acid permease (azaleucine resistance)*	E	-				1.9 ± 0.1	1.4 ± 0.1	2.3 ± 0.2	1.4 ± 0.2	1 ± 0.1
AF1756		Predicted metal-binding protein*	S	-	**-1.61**			1.3 ± 0.1	1.2 ± 0.1	1.5 ± 0.1	0.9 ± 0.1	0.7
AF1757		hypothetical protein	X	-				0	0	0	0	0
AF1758		uncharacterized conserved protein*	S	-				0.1	0.1	0.1	0.1	0.1
AF1759		Nucleotide-binding protein containing DisA_N domain*	S	-				0.2	0.2	0.2	0.2	0.2
AF1760		Nucleotide-binding protein, uspA family*	T	+				1.7 ± 0.1	1.6 ± 0.2	1.7 ± 0.1	1.6 ± 0.1	1.4 ± 0.1
AF1761	leuD	3-isopropylmalate dehydratase small subunit	E	+				0.5 ± 0.1	0.6 ± 0.1	0.5	0.5 ± 0.1	0.5 ± 0.1
AF1762		hypothetical protein	X	-				0	0	0	0	0.1
AF1763		lipase, putative	R	+				0.2	0.2	0.3	0.2	0.1
AF1764		dCMP deaminase, putative	F	-				1	1.1 ± 0.1	1.4 ± 0.1	1 ± 0.1	0.9 ± 0.1
AF1765		Predicted metal-dependent hydrolase with the TIM-barrel fold*	R	-				0.8 ± 0.1	0.7	0.9 ± 0.1	0.7	0.5
AF1766		amino-acid ABC transporter, periplasmic binding protein/protein kinase	E	+				0.7	0.7	0.7 ± 0.1	0.8 ± 0.1	1.1 ± 0.1
AF1767	dppA	dipeptide ABC transporter, dipeptide-binding protein	E	-				**3.4**	**3.2**	**3.3**	**3.4**	**3.2**
AF1768	dppB	dipeptide ABC transporter, permease protein	E	+	**-1.79**	**-1.65**		2.2	2.1	1.7 ± 0.1	1 ± 0.1	1.1
AF1769	dppC	dipeptide ABC transporter, permease protein	E	+	**-1.77**			2 ± 0.2	1.6	1.5 ± 0.1	0.9 ± 0.1	0.9 ± 0.1
AF1770	dppD	dipeptide ABC transporter, ATP-binding protein	E	+	**-1.78**	**-1.58**		2.4	2.3	2	1.2 ± 0.1	1.2
AF1771	dppF	dipeptide ABC transporter, ATP-binding protein	E	+	**-2.07**	**-1.89**		1.2 ± 0.1	1.3	0.9 ± 0.1	0.5 ± 0.1	0.5
AF1772	fadD-7	long-chain-fatty-acid--CoA ligase	I	+	**2.66**			0.8 ± 0.1	1.4 ± 0.1	0.9 ± 0.1	**3**	**3**
AF1773		iron-sulfur binding reductase	C	+				2.6	2.8	2.4	2.2	1.7 ± 0.1
AF1774	cat-2	cationic amino acid transporter	E	+				0.2	0.1	0.2	0.1	0.1
AF1775		atrazine chlorohydrolase, putative	F	-				0.3	0.4 ± 0.1	0.3	0.3	0.3
AF1776		Preprotein translocase subunit Sec61beta*	U	+				1.2 ± 0.1	1.3 ± 0.1	1.6 ± 0.2	1.3 ± 0.2	1.5 ± 0.3
AF1777		uncharacterized conserved protein, NIF3 homolog*	S	-				0.5	0.7	0.6	0.5	0.6 ± 0.1
AF1778	spoVG	stage V sporulation protein	S	+			**1.89**	1 ± 0.3	1.6 ± 0.2	0.8 ± 0.2	0.7 ± 0.2	1.7 ± 0.2
AF1779		2-hydroxyacid dehydrogenase, putative	H	+				0.7 ± 0.1	0.5 ± 0.1	0.6 ± 0.1	0.6 ± 0.1	0.7 ± 0.1

AF1780	ilvB-2	acetolactate synthase, large subunit	E	+				0.8 ± 0.1	0.6 ± 0.1	0.7 ± 0.1	0.7 ± 0.1	0.7
AF1781	nfeD	nodulation protein NfeD	O	+				1.6 ± 0.2	1.8 ± 0.1	1.6 ± 0.1	1.4 ± 0.1	1.5 ± 0.1
AF1782		uncharacterized conserved protein*	S	+				2.8	3.1	2.8	2.6 ± 0.2	2.9
AF1783	ksgA	dimethyladenosine transferase	J	+				1.6 ± 0.1	1.8 ± 0.1	1.6 ± 0.1	1.4 ± 0.1	1.3 ± 0.1
AF1784	hemK	protoporphyrinogen oxidase	J	+	-1.36			0.4	0.4	0.4	0.3	0.3
AF1785		iron-dependent repressor	K	-				0.9 ± 0.1	1 ± 0.1	1 ± 0.1	0.9	0.9 ± 0.1
AF1786		Conserved protein/domain typically associated with flavoprotein oxygenase, DIM6/NTAB family*	R	-			-1.29	2.6	2.1	2.8 ± 0.2	2.4	1.9 ± 0.1
AF1787		Predicted transcriptional regulator*	K	-				1.4 ± 0.1	1.2	1.7 ± 0.1	1.4 ± 0.1	1.2 ± 0.1
AF1788	mtaP	methylthioadenosine phosphorylase	F	-			-1.15	2.5	2.2	2.7	2.8	2.4
AF1789	gptA-2	xanthine-guanine phosphoribosyltransferase	R	-		1.10		2.5	2.5	2.8	2.9	2.7
AF1790		DNA polymerase II small subunit	L	-				2.1 ± 0.2	2.2	2.3	2.4	2.4
AF1791	sec11	signal sequence peptidase	U	+				0.2	0.2	0.2	0.2	0.2
AF1792		Predicted membrane protein*	S	+				0.3	0.3	0.3	0.3	0.3
AF1793		repressor protein	K	+				0.3	0.4 ± 0.1	0.4	0.4	0.4
AF1794	ino1	myo-inositol-1-phosphate synthase	I	-				2.5	2.4	2.6	2.2	1.8 ± 0.1
AF1795	celM	endoglucanase	G	-				2.8 ± 0.2	2.6	2.7	2.6 ± 0.2	2.3
AF1796		Predicted sugar phosphate isomerase involved in capsule formation*	M	-				1.9 ± 0.1	1.6 ± 0.1	2 ± 0.2	1.9 ± 0.2	1.6
AF1797		phosphate regulatory protein, putative	P	-	**-2.10**			0.4 ± 0.1	0.3	0.4	0.2	0.1
AF1798		phosphate permease, putative	P	+				1.6 ± 0.3	2.1	1.7 ± 0.1	1.1 ± 0.2	1.4 ± 0.1
AF1799		Phosphate transport regulator (distant homolog of Phou)*	P	+	-1.39			1.4 ± 0.1	1.6 ± 0.1	1.6 ± 0.1	1.1 ± 0.1	1
AF1800		Predicted permease, member of the PurR regulon*	R	-				0.5	0.4	0.5	0.5	0.5
AF1801		rRNA methylase*	J	-				0.2	0.2	0.2	0.2	0.1
AF1803		Diphthamide synthase subunit DPH2*	J	-				0.1	0.1	0.1	0.1	0.1
AF1804		Serine/threonine protein kinase involved in cell cycle control*	T	+				0.8 ± 0.1	0.7	0.8 ± 0.1	0.8 ± 0.1	0.8 ± 0.1
AF1805		putative RNA-processing protein	R	+				2.3	1.9 ± 0.1	2.2	2.1	2.1
AF1806	topA	DNA topoisomerase I	L	+				1.6 ± 0.1	1.4	1.5 ± 0.1	1.3 ± 0.1	1.2
AF1807		Predicted transcriptional regulator*	K	-				0.2	0.2	0.2	0.2	0.3
AF1808		Transcriptional regulator containing HTH domain, ArsR family*	K	+				0.4	0.4	0.5	0.4	0.3
AF1809		hypothetical protein	X	-				0.8	0.7	1 ± 0.1	0.8 ± 0.1	0.5
AF1810		Radical SAM superfamily enzyme*	R	+				1 ± 0.1	1.3	1.2 ± 0.1	1	0.9 ± 0.1
AF1811		inosine monophosphate cyclohydrolase	F	+				0.9 ± 0.1	1.1	1	0.8	0.7 ± 0.1
AF1812		ASCH domain, predicted RNA-binding domain*	S	+				0.3	0.4	0.3	0.2	0.2
AF1813		TBP-interacting protein TIP49	K	-				1.3 ± 0.3	1.6 ± 0.1	1.8 ± 0.3	0.9 ± 0.2	0.9 ± 0.4
AF1814		uncharacterized conserved predicted metal-binding protein*	S	+			**1.51**	0.1	0.1	0.1	0.1	0.1
AF1815	argD-2	acetylornithine aminotransferase	E	+	**4.63**			0.1	0.4	0.2	1.1 ± 0.1	1.1 ± 0.1
AF1816		hypothetical protein	X	+	**3.82**			0.1	0.2	0.1	0.6 ± 0.1	0.7
AF1817		TetR family transcriptional regulator	K	+				0.5 ± 0.1	0.4	0.4	0.3 ± 0.1	0.3
AF1818		S-layer domain*	M	+				1.4 ± 0.2	1.1 ± 0.1	1.3 ± 0.1	1 ± 0.1	1 ± 0.1
AF1819		ABC transporter, ATP-binding protein	V	+				1.3 ± 0.2	1 ± 0.1	1.2 ± 0.1	1 ± 0.1	1 ± 0.1
AF1820		ABC-type antimicrobial peptide transport system, permease component*	V	+				0.3	0.3	0.3	0.2	0.2
AF1821		uncharacterized conserved protein*	S	-	**-1.53**	-1.50		0.2	0.2	0.1	0.1	0.1
AF1822	ppa	serine/threonine phosphatase	T	+		-1.18		0.2	0.2	0.2	0.2	0.1
AF1823		F420H2:quinone oxidoreductase, 16.5 kDa subunit, putative	S	+				3.3	2.6	2.7	2.9 ± 0.3	2.7
AF1824		F420H2:quinone oxidoreductase, 11.2 kDa subunit, putative	C	+				3.6	3.4	3.5	3.5	3.4
AF1825	nuoM	F420H2:quinone oxidoreductase, 53.9 kDa subunit	C	+				3.6	3.4	3.5	3.5	3.5
AF1826	nuoL	F420H2:quinone oxidoreductase, 72.4 kDa subunit.	C	+				3.3	2.9	3	3.1	3
AF1827		F420H2:quinone oxidoreductase, 43.2 kDa subunit, putative	C	+				3.6	3.4	3.5	3.5	3.5
AF1828		NADH dehydrogenase subunit A	C	+				3.5	3.4	3.4	3.4	3.4
AF1829		F420H2:quinone oxidoreductase, 39.7 kDa subunit, putative	C	+				3.3	3	3.3	3.2	3.2
AF1830	nuoD	NADH dehydrogenase subunit D	C	+				3.4	3.2	3.3	3.3	3.2
AF1831		NADH dehydrogenase subunit H	C	+				3.6	3.4	3.5	3.5	3.3
AF1832a		NADH dehydrogenase subunit I	C	+				3.5	3.4	3.4	3.4	3.2
AF1833		F420H2:quinone oxidoreductase, 39 kDa subunit, putative	C	+				3.2	3	3	3	2.9
AF1834		uncharacterized protein of DIM6/NTAB family*	R	-				0.4	0.4	0.4	0.5	0.4
AF1835		Triphosphoribosyl-dephospho-CoA synthetase*	H	-			-1.35	0.5	0.4	0.5 ± 0.1	0.6 ± 0.1	0.4
AF1837m	nadA	quinolinate synthetase	H	+	**-1.53**			0.1	0.1	0.1	0	0
AF1838		Predicted dinucleotide-utilizing enzyme*	R	+	**-1.73**			0.2	0.2	0.2	0.1	0.1
AF1839	nadC	nicotinate-nucleotide pyrophosphorylase	H	+	**-1.52**			0.2	0.1	0.2	0.1	0.1
AF1840	map	methionine aminopeptidase	J	+			**1.53**	0.7	1.3 ± 0.1	0.8 ± 0.1	0.7	1 ± 0.1
AF1841	cbiO-2	cobalt transport ATP-binding protein	P	-				0.4	0.4	0.5	0.3	0.3
AF1842	cbiQ-2	cobalt transport protein	P	-				0.2	0.2	0.2	0.3 ± 0.1	0.5 ± 0.1
AF1843	cbiM-2	cobalamin biosynthesis protein	P	-		**1.63**		1.1 ± 0.2	1.1 ± 0.1	1.7 ± 0.1	2 ± 0.3	2.2 ± 0.2
AF1844		uncharacterized conserved protein*	S	-			-1.49	0.5	0.3	0.5	0.4 ± 0.1	0.3
AF1845		uncharacterized conserved protein*	S	-				1.9 ± 0.1	1.7 ± 0.1	2 ± 0.1	1.9 ± 0.1	1.8 ± 0.1
AF1846		Transcriptional regulator, contains HTH domain*	K	-	-1.37			0.4	0.4	0.4	0.3	0.3
AF1847		hypothetical protein	X	-				0.3	0.2	0.3	0.2	0.2
AF1848		hypothetical protein	X	-				0.2	0.1	0.2	0.1	0.1
AF1849	cooS	carbon monoxide dehydrogenase, catalytic subunit	C	+				0.9 ± 0.1	2.1	1 ± 0.1	1.8 ± 0.1	1.6
AF1850		Metal-dependent hydrolase of the beta-lactamase superfamily II*	R	+	**2.26**			0.2	0.3	0.2	0.4 ± 0.1	0.6
AF1851		Fe-S oxidoreductase*	C	+				0.2	0.3	0.2	0.4	0.6
AF1852		hypothetical protein	X	-				0.5	0.6	0.4	0.4	0.5
AF1853		ArsR family transcriptional regulator	K	+		**-1.86**		0.1	0.1	0.1	0.1	0
AF1854	cat2-2	4-hydroxybutyrate CoA transferase	C	-	**-1.62**			2.7	2.5	2.6	1.5 ± 0.1	1.6 ± 0.1
AF1855	entE	2,3-dihydrosybenzoate-AMP ligase	I	+	**17.87**			0	0	0	0.8 ± 0.1	0.9
AF1856		uncharacterized conserved protein*	S	+	**-1.59**			0.8 ± 0.1	0.7	0.9 ± 0.1	0.5	0.4
AF1858	noxA-5	NADH oxidase	P	-				0.2	0.3	0.2	0.1	0.1
AF1859		CRISPR-associated endoribonuclease Cas6*	V	-				0.2	0.2	0.1	0.1	0.1
AF1860		hypothetical protein	X	-	-1.47			1 ± 0.1	0.9 ± 0.1	1	0.7 ± 0.1	0.6
AF1861		CRISPR system related protein, RAMP superfamily Cas7 group*	V	-	**-1.94**	**-1.83**		1.9 ± 0.1	2 ± 0.1	1.3	0.9 ± 0.1	0.8 ± 0.1
AF1862		CRISPR system related protein, small subunit of CASCADE complex*	V	-		**-2.15**		1.6 ± 0.1	1.9 ± 0.1	1 ± 0.1	0.7 ± 0.1	0.7 ± 0.1
AF1863		CRISPR system related protein, RAMP superfamily Cas7 group*	V	-	**-2.32**	**-2.20**		1.3 ± 0.1	1.6 ± 0.1	0.8 ± 0.1	0.5 ± 0.1	0.5
AF1864		CRISPR-associated protein Csx3*	V	-		**-2.30**		1.2 ± 0.1	1.4	0.7 ± 0.1	0.4 ± 0.1	0.5 ± 0.1
AF1865		hypothetical protein	X	-		**-2.42**		1 ± 0.1	1	0.5 ± 0.1	0.3	0.3
AF1866		CRISPR system related protein, RAMP superfamily Cas5 group*	V	-		**-2.25**		0.7 ± 0.1	0.6	0.3	0.2	0.2
AF1867		CRISPR associated protein, polymerase-related domain and HD family nuclease*	V	-	**-2.31**	**-2.19**		1.1 ± 0.2	1 ± 0.1	0.6 ± 0.1	0.4 ± 0.1	0.3 ± 0.1
AF1868		CRISPR system related protein, RAMP superfamily Cas7 group*	V	-		**-3.29**		0.5 ± 0.1	0.7 ± 0.1	0.2	0.1	0.1
AF1869		Transcriptional regulator containing HTH domain, ArsR family*	K	-				0.1	0.1	0.1	0.1	0.1
AF1870		CRISPR system associated protein, predicted small subunit of CASCADE complex*	V	+				1.2 ± 0.3	1 ± 0.1	0.8 ± 0.1	0.7 ± 0.1	0.3
AF1871		CRISPR system related protein Cas7, RAMP superfamily*	V	+				2.3 ± 0.2	2.5	1.9 ± 0.1	1.6 ± 0.2	1.2 ± 0.1
AF1872		CRISPR system related protein Cas5, RAMP superfamily*	V	+				2.3 ± 0.2	2.4	1.9	1.8 ± 0.1	1.4 ± 0.1
AF1873		CRISPR system related protein, large subunit of CASCADE complex*	V	+	-1.33	-1.31		2.4	2.5	2	1.9 ± 0.1	1.5 ± 0.1
AF1874		CRISPR-associated helicase Cas3*	V	+		-1.33		1.7 ± 0.2	1.8 ± 0.1	1.3 ± 0.1	1.1 ± 0.1	1.2 ± 0.1
AF1875		HD superfamily nuclease*	L	+	**-1.76**	**-1.66**		0.5 ± 0.1	0.5	0.3	0.2	0.2
AF1876		CRISPR-associated protein Cas2*	V	+				0.1	0.1	0.1	0.1	0.1
AF1877		CRISPR-associated protein Cas4, RecB family exonuclease*	V	-			**1.53**	0.1	0.2	0.1	0.2	0.2
AF1878		CRISPR-associated protein Cas1*	V	-				0.4	0.6	0.3	0.6 ± 0.1	0.7
AF1879		CRISPR-associated protein, RecB family exonuclease*	V	-				0.3 ± 0.1	0.4	0.2	0.4 ± 0.1	0.5
AF1880		hypothetical protein	X	+				0.5 ± 0.1	0.9 ± 0.1	0.5 ± 0.1	0.4	0.5 ± 0.1
AF1881		hypothetical protein	X	-				0.3 ± 0.1	0.3	0.2	0.2	0.2
AF1882		hypothetical protein	X	-	**-1.65**			0.2	0.2	0.2	0.1	0.1
AF1883	drrB	daunorubicin resistance membrane protein	V	-				0.5 ± 0.1	0.5 ± 0.1	0.5	0.4	0.4
AF1884	drrA	daunorubicin resistance ATP-binding protein	V	-				0.6	0.6	0.5	0.5	0.4
AF1885	rpoH	DNA-directed RNA polymerase subunit H	K	+			-1.34	1.3 ± 0.1	0.9 ± 0.1	1.3 ± 0.1	1.3 ± 0.1	1 ± 0.1

AF1886	rpoB2	DNA-directed RNA polymerase subunit beta''	K	+				2.8	2.5	2.9	2.9	2.9
AF1887	rpoB1	DNA-directed RNA polymerase subunit B'	K	+				**3.3**	2.9	**3.3**	**3.3**	**3.1 ± 0.2**
AF1888	rpoA1	DNA-directed RNA polymerase subunit alpha	K	+				**3.3**	**3.2**	**3.3**	**3.4**	**3.4**
AF1889	rpoA2	DNA-directed RNA polymerase subunit A''	K	+				**3**	2.9	2.9	**3**	**3**
AF1890	rpl30E	50S ribosomal protein L30E	J	+				**3.2**	**3.3**	**3.3**	**3.3**	**3.4**
AF1891	nusA	transcription elongation factor NusA-like protein	K	+				**3**	**3**	**3**	**3**	**3.2**
AF1892	rps12P	30S ribosomal protein S12P	J	+				**3.2**	**3.2**	**3.3**	**3.3**	**3.3**
AF1893	rps7P	30S ribosomal protein S7P	J	+				**3.5**	**3.5**	**3.5**	**3.5**	**3.6**
AF1894	fus	elongation factor EF-2	J	+				**3**	2.8	2.9	2.9	2.8
AF1895		Predicted Fe-S-cluster oxidoreductase*	R	+				1 ± 0.2	0.8 ± 0.2	1.1 ± 0.1	0.8 ± 0.2	0.9 ± 0.1
AF1896	isf-3	iron-sulfur flavoprotein	R	+	1.47			0.8 ± 0.1	1	1 ± 0.1	1.5 ± 0.1	1.4 ± 0.1
AF1897		Glycosyltransferase*	M	-				0.8	1 ± 0.1	0.8	0.8 ± 0.1	0.6 ± 0.1
AF1898		response regulator	T	+				0.7 ± 0.1	0.9 ± 0.1	0.7 ± 0.1	0.7	0.8 ± 0.1
AF1899		DNA primase	L	+				2.4	2.6	2.4	2.5	2.6 ± 0.2
AF1900	cmk	cytidylate kinase	F	-		-1.32		1.2 ± 0.1	1.2	1	0.8 ± 0.1	0.9 ± 0.1
AF1901		OxaA/SpoJ/YigC translocase/secretase, sec-independent integration of nascent memrane proteins into membrane*	U	-		-1.33		1.5 ± 0.1	1.5	1.1 ± 0.1	1 ± 0.1	1.1 ± 0.1
AF1902	secY	preprotein translocase subunit SecY	U	-				2.9	2.7	2.5	2.4	2.5 ± 0.2
AF1903	rpl15P	50S ribosomal protein L15P	J	-				2.9	2.7	2.6	2.5 ± 0.2	2.2
AF1904	rpl30p	50S ribosomal protein L30P	J	-				**3.2**	**3.2**	**3**	**3**	**3**
AF1905	rps5p	30S ribosomal protein S5P	J	-				**3.3**	**3.3**	**3.3**	**3.2**	**3.2**
AF1906	rpl18p	50S ribosomal protein L18P	J	-				**3.2**	**3.2**	**3.1**	**3.1**	**3.1**
AF1907	rpl19e	50S ribosomal protein L19e	J	-				**3.4**	**3.2**	**3.4**	**3.3**	**3**
AF1908	rpl32e	50S ribosomal protein L32e	J	-				**3.2**	**3.1**	**3.1**	**3.1**	2.9
AF1909	rpl6P	50S ribosomal protein L6P	J	-				**3.2**	**3.2**	**3.3**	**3.2**	**3 ± 0.2**
AF1910	rps8p	30S ribosomal protein S8P	J	-				**3.3**	**3.2**	**3.2**	**3.3**	**3.1**
AF1911	rps14P	30S ribosomal protein S14P	J	-				2.9	**3**	2.8	2.9	2.8
AF1912	rpl5p	50S ribosomal protein L5P	J	-				**3.3**	**3.2**	**3.2**	**3.2**	**3.2**
AF1913	rps4E	30S ribosomal protein S4e	J	-				**3.5**	**3.4**	**3.4**	**3.4**	**3.3**
AF1914	rpl24p	50S ribosomal protein L24P	J	-				**3.4**	**3.4**	**3.4**	**3.4**	**3.3**
AF1915	rpl14p	50S ribosomal protein L14P	J	-				**3.3**	**3.3**	**3.3**	**3.3**	**3.2**
AF1916	rps17p	30S ribosomal protein S17P	J	-				**3.4**	**3.3**	**3.3**	**3.4**	**3.2**
AF1917		RNase P/RNase MRP subunit p29*	J	-				2.9 ± 0.2	**3.1**	2.9	2.9	2.9
AF1918c	#N/A	50S ribosomal protein L29P	0.1	-				**3.3**	**3.3**	**3.3**	**3.3**	**3.2**
AF1919	rps3p	30S ribosomal protein S3P	J	-				**3.2**	**3.3**	**3.2**	**3.3**	**3 ± 0.3**
AF1920	rpl22p	50S ribosomal protein L22P	J	-				**3.4**	**3.4**	**3.4**	**3.4**	**3.3**
AF1921	rps19p	30S ribosomal protein S19P	J	-				**3.3**	**3.2**	**3.2**	**3.3**	**3.1 ± 0.2**
AF1922	rpl2p	50S ribosomal protein L2P	J	-				**3.3**	**3.4**	**3.3**	**3.3**	**3.3**
AF1923	rpl23P	50S ribosomal protein L23P	J	-				**3.5**	**3.5**	**3.4**	**3.5**	**3.3 ± 0.2**
AF1924	rpl4lp	50S ribosomal protein L4P	J	-				**3.4**	**3.4**	**3.3**	**3.4**	**3.3**
AF1925	rpl3p	50S ribosomal protein L3P	J	-				**3**	**3**	2.9	**3**	2.8
AF1926		RNA methyltransferase, SPOuT superfamily*	R	-				0.7 ± 0.1	0.5	0.6	0.6 ± 0.1	0.4
AF1927		Outer membrane lipoprotein-sorting protein*	M	-				0.5 ± 0.1	0.4	0.6	0.6 ± 0.1	0.4
AF1928	fwdD-2	tungsten formylmethanofuran dehydrogenase, subunit D	C	+				**3.4**	**3.4**	**3.5**	**3.3**	**3.3**
AF1929	fwdB-2	tungsten formylmethanofuran dehydrogenase, subunit B	C	+				**3.5**	**3.5**	**3.5**	**3.5**	**3.5**
AF1930	fwdA	tungsten formylmethanofuran dehydrogenase, subunit A	C	+				**3.5**	**3.5**	**3.5**	**3.5**	**3.5**
AF1931	fwdC	tungsten formylmethanofuran dehydrogenase, subunit C	C	+				**3.3**	**3.4**	**3.2**	**3.3**	**3.4**
AF1932	fadD-8	long-chain-fatty-acid--CoA ligase	I	+	**2.12**			0.4	0.5	0.3	0.7	1
AF1933		monoamine oxidase regulatory protein, putative	I	-				0.2	0.1	0.1	0.1	0.1
AF1934		Nucleotide-binding protein, uspA family*	T	-	**-1.77**			0.4 ± 0.1	0.2	0.3	0.2	0.1
AF1935	mch	N(5),N(10)-methenyltetrahydromethanopterin cyclohydrolase	H	-				**3.4**	**3.4**	**3.5**	**3.4**	**3.3**
AF1936		PIN domain containing protein*	V	-				0.6	0.4	0.7	0.8 ± 0.1	1
AF1937	minD-2	cell division inhibitor	N	-	**3.65**			0	0	0	0.1	0.2
AF1938		Acyl-CoA synthetase, ATP-grasp containing subunit*	C	+	-1.36			0.8	1	0.8	0.7	0.6
AF1939		hypothetical protein	X	-				0	0	0	0	0
AF1940	purL	phosphoribosylformylglycinamidine synthase II	F	-				1.2 ± 0.1	1.3	1.1	1.1	1
AF1941		Phosphoribosylformylglycinamidine (FGAM) synthase, PurS component*	F	-	-1.36			0.3	0.3	0.3	0.2	0.2
AF1942m		beta-propeller repeat protein fused to CARDB-like adhesion domain*	S	+				0.2	0.2	0.1	0.1	0.1
AF1944		hypothetical protein	X	+				0.5 ± 0.1	0.6	0.4	0.4	0.3
AF1945		hypothetical protein	X	+				0.1	0.2	0.1	0.1	0.1
AF1946		cysteine proteinase, putative	S	+	-1.30			0.5	0.5	0.5	0.4	0.4
AF1947		hypothetical protein	X	+				0.1	0.1	0.1	0.1	0.1
AF1948		hypothetical protein	X	+	**2.66**			0.7 ± 0.1	0.9 ± 0.1	0.6	2.1 ± 0.2	2 ± 0.2
AF1949		beta-propeller repeat protein*	S	+	**3.61**			0.1	0.2	0.1	0.4 ± 0.1	0.6 ± 0.1
AF1950	hisIE	phosphoribosyl-AMP cyclohydrolase/phosphoribosyl-ATP pyrophosphohydrolase	E	+				0.4	0.5	0.4	0.4	0.3
AF1951		ATPase	R	+			1.28	1.7	2.5	1.7 ± 0.1	1.8 ± 0.1	2
AF1952		Presenilin-like membrane protease, A22 family*	R	+				0.2	0.4	0.2	0.2	0.3
AF1953		Serine/threonine protein phosphatase PP2A family*	T	+				0.8 ± 0.1	1.1	0.8 ± 0.1	0.6	0.6
AF1954	gatA-1	Glu-tRNA amidotransferase, subunit A	J	-				0.3	0.9 ± 0.1	0.3	1 ± 0.1	1.4 ± 0.1
AF1955	pheS	phenylalanyl-tRNA synthetase subunit alpha	J	+				1.6 ± 0.1	2	1.6 ± 0.1	1.4 ± 0.1	1.3 ± 0.1
AF1956		chromatin protein	K	+	**-1.92**			1.9 ± 0.1	1.7 ± 0.1	1.7 ± 0.1	0.8 ± 0.1	1 ± 0.1
AF1957	hgdB	2-hydroxyglutaryl-CoA dehydratase, subunit beta	E	+			-1.24	1.5 ± 0.1	1.1	1.6 ± 0.1	1.3 ± 0.1	1.2 ± 0.1
AF1958	hgdA	2-hydroxyglutaryl-CoA dehydratase, subunit alpha	E	+				1.6 ± 0.1	1.4	1.7 ± 0.1	1.9 ± 0.1	1.9 ± 0.1
AF1959	hgdC	(R)-hydroxyglutaryl-CoA dehydratase activator	I	+				1.3 ± 0.1	0.8	1.2 ± 0.1	1.5 ± 0.1	1.2 ± 0.1
AF1960		DNA helicase, putative	L	-				0.7 ± 0.1	0.4	0.6 ± 0.1	0.5 ± 0.1	0.5 ± 0.1
AF1961	pflX	pyruvate formate-lyase activating enzyme	C	-				1.3 ± 0.1	0.9	1.2 ± 0.1	1.2 ± 0.1	1.4 ± 0.1
AF1962		hypothetical protein	X	+				0.1	0.2	0.2	0.1	0.1
AF1963	acn	aconitase	E	+				**3**	2.8	**3**	2.7	2.1
AF1964		uncharacterized conserved protein*	S	-				1.4	1.1	1.5 ± 0.1	1.1 ± 0.1	0.7
AF1965		Holliday junction resolvase*	L	+	**-1.75**			0.3	0.3	0.4	0.2	0.2
AF1966		uncharacterized archaeal Zn-finger protein*	R	+				2.1	1.9 ± 0.1	2.1	1.7 ± 0.1	1.6 ± 0.1
AF1967		Predicted nucleotidyltransferase*	R	+				1.8 ± 0.1	1.5 ± 0.1	1.8 ± 0.2	1.3 ± 0.1	1.3 ± 0.1
AF1968		ROK family transcriptional regulator	K	-			-1.34	0.3	0.3	0.4	0.3	0.2
AF1969		uncharacterized conserved protein*	S	+			**1.58**	0.2	0.4	0.2	0.3	0.3
AF1970		TPR domain-containing protein	R	-				1.2	1 ± 0.1	1.1 ± 0.1	0.9 ± 0.1	0.8 ± 0.1
AF1971	hsp20-2	small heat shock protein	O	-				1.5 ± 0.3	1.5 ± 0.1	2.3 ± 0.2	1 ± 0.1	1 ± 0.2
AF1972		NMD protein affecting ribosome stability and mRNA decay*	J	+				0.4	0.6	0.5	0.5	0.5
AF1973		Wybutosine (yW) biosynthesis enzyme, TYW2 transferase*	J	+				0.3	0.4	0.4	0.4	0.3
AF1974	hemB	delta-aminolevulinic acid dehydratase	H	-				2.4	2.2	2.2	2.2	2.1
AF1975	hemA	glutamyl-tRNA reductase	H	-				0.9 ± 0.1	1	0.9 ± 0.1	0.8	0.9 ± 0.1
AF1976		proteasome-activating nucleotidase	O	-				**3.5**	**3.6**	**3.4**	**3.5**	**3.5**
AF1977		Predicted transcription factor, homolog of eukaryotic MBF1*	K	+				1.4 ± 0.1	1.2	1.4 ± 0.1	1.1 ± 0.1	1
AF1978	cutA	periplasmic divalent cation tolerance protein	P	+				2.1 ± 0.2	2	2.3	1.8 ± 0.1	1.9 ± 0.1
AF1979		Minimal nucleotidyltransferase*	V	+				1.5 ± 0.1	1.7	1.7 ± 0.1	1.3 ± 0.1	1.5
AF1980	helC	heme exporter protein C	O	-				0.3	0.2	0.3	0.2	0.3
AF1981		ABC transporter, permease protein	P	-				0.9 ± 0.2	0.6 ± 0.1	1 ± 0.1	0.8 ± 0.1	0.9 ± 0.1
AF1982		ABC transporter, ATP-binding protein	P	-				1.9 ± 0.3	1.6 ± 0.1	2.3	1.9 ± 0.2	2.3
AF1983		ABC transporter, periplasmic binding protein	P	-				2.5 ± 0.3	2.1 ± 0.2	2.8	2.5	2.7 ± 0.2
AF1984	troR	iron-dependent repressor	K	-				2 ± 0.4	1.3 ± 0.3	2.4	2.1 ± 0.2	2.1
AF1985	ilvC	ketol-acid reductoisomerase	E	+				2.1	2.6	2 ± 0.1	2	2.1
AF1986		hypothetical protein	X	-				0.7	0.8 ± 0.1	0.7 ± 0.1	0.5 ± 0.1	0.6 ± 0.1
AF1987		Transcriptional regulator containing HTH domain, ArsR family*	K	-				2.7	2.4	2.6	2.5	2.1 ± 0.2
AF1988		Adenylate cyclase, class 2 (thermophilic)*	F	-				0.4	0.3	0.4	0.4	0.4
AF1989	slyD	peptidyl-prolyl cis-trans isomerase	O	+	**-1.53**			2.3	2.7	2.3	1.6 ± 0.1	1.5 ± 0.1

AF1990		uncharacterized conserved protein*	S	+				1.2 ± 0.1	0.4	1.1 ± 0.2	0.8 ± 0.1	1 ± 0.1
AF1991		hypothetical protein	X	+				1.4 ± 0.2	0.6	1.2 ± 0.2	1 ± 0.1	1.4 ± 0.2
AF1992		calcium-binding protein, putative	S	+				0.9 ± 0.2	0.4	0.8 ± 0.2	0.6	0.6 ± 0.1
AF1993		uncharacterized conserved protein*	S	+				0.5 ± 0.1	0.2	0.4 ± 0.1	0.3	0.3
AF1994		uncharacterized conserved protein*	S	+				0.2	0.1	0.1	0.1	0.1
AF1995		sodium- and chloride-dependent transporter	R	+	**-1.86**	**-1.74**		0.6 ± 0.1	0.5	0.4	0.2	0.3
AF1996	vapC-1	virulence associated protein C	V	-			1.29	0	0	0	0	0
AF1997		Predicted antitoxin, copG family*	V	-				0.1	0.1	0.1	0.1	0.1
AF1998		iron-sulfur binding reductase	C	-				0.8	1.1 ± 0.1	0.7	0.7	0.8
AF1999		uncharacterized conserved protein*	S	-				2 ± 0.3	2.3	1.8 ± 0.2	1.3 ± 0.1	2 ± 0.1
AF2000	ahcY-2	S-adenosyl-L-homocysteine hydrolase	H	-	-1.12	-1.12		2.7	2.6	2.5	2.3	2.3
AF2001		Predicted nucleotide kinase (CMP/AMP kinase related)*	F	-				**3**	**3**	**3**	2.8	**3 ± 0.2**
AF2002	hisC-1	histidinol-phosphate aminotransferase	E	-				1.6 ± 0.1	1.5 ± 0.1	1.4 ± 0.1	1.2 ± 0.1	1.3 ± 0.1
AF2003		uncharacterized conserved protein*	S	-				1.5 ± 0.1	1.5 ± 0.1	1.4 ± 0.1	1.3 ± 0.1	1.5 ± 0.1
AF2004		L-tyrosine decarboxylase	E	-				1.5 ± 0.1	1.4 ± 0.1	1.3 ± 0.1	1.3 ± 0.1	1.6 ± 0.1
AF2005	mobA	molybdopterin-guanine dinucleotide biosynthesis protein A	H	+				0.6	0.4	0.6	0.4	0.4
AF2006	moaA	molybdenum cofactor biosynthesis protein A	H	+				0.5	0.5	0.5	0.5 ± 0.1	0.5
AF2007	ribG	riboflavin-specific deaminase	H	+				0.8 ± 0.1	0.7	0.9	0.9 ± 0.1	0.7 ± 0.1
AF2008		Predicted transcription regulator containing HTH domain*	K	-				0.1	0.1	0.1	0.1	0.1
AF2009	nirJ-2	heme biosynthesis protein	R	+				0.6 ± 0.1	1 ± 0.1	0.4 ± 0.1	0.2	0.2
AF2010		hypothetical protein	X	+		**-1.79**		1.3 ± 0.1	0.9	0.6	0.6 ± 0.1	0.5 ± 0.1
AF2011		Transcriptional regulator AbrB*	K	+				0.2	0.2	0.2	0.1	0.2
AF2012		PIN domain containing protein*	V	+				0.1	0.1	0.1	0.1	0.1
AF2013	ftsA-3	coenzyme F390 synthetase	H	+				2.7	**3.3 ± 0.2**	2.6	2.6	2.7
AF2014		sugar transporter, putative	G	+				0.1	0.1	0.1	0.1	0.1
AF2015	ilvB-3	acetolactate synthase, large subunit	E	+				0.3	0.2	0.2	0.3	0.3
AF2016	galE-2	uDP-glucose 4-epimerase	M	+				0.3	0.2	0.2	0.2	0.2
AF2017	hbd-9	3-hydroxyacyl-CoA dehydrogenase	I	+				0.3	0.2	0.2	0.2	0.2
AF2018		Predicted permease*	R	+				0.3	0.2	0.2	0.2	0.2
AF2019		alcohol dehydrogenase, iron-containing	C	+				0.2	0.1	0.2	0.1	0.1
AF2020		uncharacterized conserved protein*	S	-				0.1	0.1	0.1	0.1	0.1
AF2021	mreB	rod shape-determining protein	D	+				1.9 ± 0.1	2.1	1.9 ± 0.1	2 ± 0.1	1.8 ± 0.1
AF2022		uncharacterized conserved protein*	S	+			1.13	2.1	2.6	2.3	2.5	2.6
AF2023		Coenzyme F420-dependent N5,N10-methylene tetrahydromethanopterin reductase or related flavin-dependent oxidoreductase*	C	+				1.1	1.4	1.3	1.3 ± 0.1	1.2
AF2024	hisC-2	histidinol-phosphate aminotransferase	E	+				0.5 ± 0.1	0.8 ± 0.1	0.6	0.5 ± 0.1	0.7
AF2025		hypothetical protein	X	-				0.9 ± 0.2	0.6 ± 0.1	0.9 ± 0.1	0.7 ± 0.1	0.7 ± 0.1
AF2026		uncharacterized conserved protein*	S	-				1.5 ± 0.2	1.1 ± 0.2	1.3 ± 0.1	1.3 ± 0.1	1.5 ± 0.1
AF2027		uncharacterized conserved protein*	S	-				1.8 ± 0.2	1.2 ± 0.1	1.8 ± 0.1	1.9 ± 0.1	1.6 ± 0.1
AF2028		uncharacterized conserved protein*	S	-				**3**	**3.2**	**3**	**3.2**	**3.2 ± 0.2**
AF2029		Exopolyphosphatase-related protein fused to TrkA-N domain*	R	+				2.7	2.8	2.7	2.9	2.7
AF2030	iorB	indolepyruvate ferredoxin oxidoreductase, subunit beta	C	+				1.4 ± 0.1	1.6	1.3 ± 0.1	1.3 ± 0.1	1.4 ± 0.1
AF2031		uncharacterized archaeal coiled-coil protein*	S	-				2.9	**3.2**	2.9	2.8	2.7
AF2032		signal-transducing histidine kinase, putative	T	+				0.5 ± 0.1	1 ± 0.1	0.7 ± 0.1	0.9 ± 0.1	1.1 ± 0.1
AF2033	alkK-5	acyl-CoA synthetase	I	-				**3.1**	**3.1**	**3.3**	**3.1**	**3.2**
AF2034	pepQ	X-pro aminopeptidase	E	+				0.8 ± 0.1	1.3	0.8 ± 0.1	0.9	0.9
AF2035	serS	seryl-tRNA synthetase	J	-				1.7 ± 0.1	1.7 ± 0.1	1.7 ± 0.1	1.6 ± 0.1	1.6
AF2036	coxD	cytochrome C oxidase folding protein	O	-				0.8 ± 0.1	0.6	0.7	0.6 ± 0.1	0.6
AF2037	eif2BD	translation initiation factor IF-2B subunit delta	J	-				0.8	1	0.7	0.7	0.7
AF2038		Predicted RNA-binding protein homologous to eukaryotic snRNP*	K	+				1.8 ± 0.1	2.1	1.7	1.7 ± 0.1	1.7
AF2039		proliferating-cell nucleolar antigen P120, putative	J	+				1 ± 0.1	1	0.9	0.9 ± 0.1	0.7
AF2040		Predicted membrane protein*	S	-				0.4 ± 0.1	0.3	0.2	0.2	0.2
AF2041		uncharacterized conserved protein*	S	+				0.2	0.1	0.1	0.2	0.2
AF2042	pyrH	uridylate kinase	F	+				0.8 ± 0.1	0.6	0.9 ± 0.1	0.7 ± 0.1	0.4
AF2043		uncharacterized conserved protein*	S	-			**-1.56**	0.5	0.4	0.6	0.5	0.3
AF2044	pssA	CDP-diacylglycerol--serine O-phosphatidyltransferase	I	-				0.4	0.3	0.4	0.3	0.4
AF2045	psd2	phosphatidylserine decarboxylase	I	-				0.7 ± 0.1	0.6	0.6	0.6	0.7
AF2046		cytochrome oxidase, subunit I, putative	U	-				0.7	0.5	0.6	0.6	0.6
AF2047		thymidylate synthase, putative	F	-			-1.26	1.6	1.3 ± 0.1	1.6	1.5	1.1
AF2048		uncharacterized conserved protein*	S	+				1.5	1.6 ± 0.1	1.5 ± 0.1	1.4 ± 0.1	1.2 ± 0.1
AF2049		Predicted GTPase or GTP-binding protein*	R	+				1.5 ± 0.2	1.4 ± 0.1	1.7 ± 0.1	1.5 ± 0.1	1.2 ± 0.1
AF2050		uncharacterized conserved protein*	S	+				0.8 ± 0.1	0.7 ± 0.1	0.8	0.7 ± 0.1	0.6
AF2051	ftsJ	cell division protein	J	+	-1.38			1.1 ± 0.1	1	1.1 ± 0.1	0.8 ± 0.1	0.6
AF2052	vorB	2-ketoisovalerate ferredoxin oxidoreductase, subunit beta	C	-				2 ± 0.2	2.1	2 ± 0.1	1.6 ± 0.1	1.6 ± 0.1
AF2053	vorA	2-ketoisovalerate ferredoxin oxidoreductase, subunit alpha	C	-				2.4	2.4	2.2	2.1	2
AF2054	vorD	2-ketoisovalerate ferredoxin oxidoreductase, subunit delta	C	-				1.2 ± 0.1	1.2	1.2 ± 0.1	1	1
AF2055	vorG	2-ketoisovalerate ferredoxin oxidoreductase, subunit gamma	C	-				0.6 ± 0.1	0.5 ± 0.1	0.6	0.5 ± 0.1	0.4
AF2056		Sec-independent protein secretion pathway component*	U	-				0.8 ± 0.1	0.8	0.8 ± 0.1	0.6	0.7 ± 0.1
AF2057	acd-10	acyl-CoA dehydrogenase	I	+				2.5	2.5	2.3	2.2 ± 0.2	1.9 ± 0.1
AF2058		ABC-type multidrug transport system, permease component*	R	+				0.8 ± 0.1	0.6	0.6	0.7 ± 0.1	0.7
AF2059		Wybutosine (yW) biosynthesis enzyme*	J	-				0.2	0.2	0.2	0.2	0.3
AF2060	rfc	replication factor C small subunit	L	-				0.9 ± 0.1	1	0.9	1 ± 0.1	1.1
AF2061		2-phospho-L-lactate guanylyltransferase, coenzyme F420 biosynthesis enzyme, CobY/MobA/RfbA family*	H	-				0.6 ± 0.2	0.3 ± 0.1	0.7 ± 0.2	0.5 ± 0.2	0.5 ± 0.1
AF2062	dpa	signal recognition particle receptor	U	-				1.8 ± 0.2	1.8 ± 0.2	2 ± 0.2	1.7 ± 0.3	1.7 ± 0.2
AF2063		prefoldin subunit alpha	O	-				1.3 ± 0.1	1.2 ± 0.1	1.4 ± 0.2	1 ± 0.1	0.8 ± 0.1
AF2064	rplX	50S ribosomal protein LX	J	-				2.1 ± 0.2	2.1	2 ± 0.1	1.7 ± 0.1	1.8
AF2065		translation initiation factor IF-6	J	-				2.9	**3.1**	**3**	2.8	2.9
AF2066	rpl31E	50S ribosomal protein L31E	J	-				**3.3**	**3.5**	**3.3**	**3.3**	**3.3**
AF2067	rpl39e	50S ribosomal protein L39e	J	-				**3.1**	**3.3**	**3.1**	**3.1**	**3.2 ± 0.2**
AF2068		DNA-binding protein*	R	-				2.6	2.7	2.6	2.4	2.6
AF2069	rps19E	30S ribosomal protein S19e	J	-				2.7	2.7	2.7	2.5	2.5
AF2070		Predicted RNA-binding protein containing KH domain, possibly ribosomal protein*	J	-				1	0.9	0.9 ± 0.1	0.8 ± 0.1	0.8
AF2071	argC	N-acetyl-gamma-glutamyl-phosphate reductase	E	-				2.1	2.1	1.9 ± 0.1	1.7 ± 0.1	1.3 ± 0.1
AF2072		uncharacterized conserved protein*	S	+				0.2	0.3	0.3	0.2	0.2
AF2073	ftr-1	tetrahydromethanopterin formyltransferase	C	-				2.4	2.7	2.5	2.4	2.3
AF2074	thiE	thiamine phosphate pyrophosphorylase	H	-				1 ± 0.1	1.3 ± 0.1	1 ± 0.1	0.9 ± 0.1	0.8
AF2075	thiM	hydroxyethylthiazole kinase	H	-				0.8	0.8	0.7	0.7 ± 0.1	0.5
AF2076		uncharacterized conserved protein*	S	-	**-1.50**			0.2	0.2	0.2	0.2	0.1
AF2077		uncharacterized conserved protein, DuF1102 family*	S	-				0.1	0.1	0.1	0.1	0.1
AF2078		Signal peptidase I*	U	-				0.1	0.1	0.1	0.1	0.1
AF2079		uncharacterized conserved protein, DuF1102 family*	S	-				0.1	0.1	0.1	0.1	0.1
AF2080		uncharacterized conserved protein, DuF1102 family*	S	-				0.2	0.2	0.2	0.2	0.1
AF2081		uncharacterized conserved protein, DuF1102 family*	S	-				0.1	0.1	0.1	0.1	0.1
AF2082		hypothetical protein	X	-			1.44	0.1	0.1	0.1	0.1	0.1
AF2083		Predicted transcriptional regulator*	K	-				0	0	0	0	0
AF2084	oadB	oxaloacetate decarboxylase, sodium ion pump subunit	C	-				1.5 ± 0.1	1.3 ± 0.1	1.3 ± 0.2	1.3 ± 0.1	1 ± 0.1
AF2085		oxaloacetate decarboxylase, biotin carboxyl carrier subunit, putative	I	-				2.5	2.4	2.4	2.3	2.2
AF2086		protease inhibitor, putative	X	-				1 ± 0.1	0.9	0.9 ± 0.1	0.9	0.8
AF2087	fib	fibrillarin	J	-				0.7	0.8	0.6	0.5	0.5
AF2088		Protein implicated in ribosomal biogenesis, Nop56p homolog*	J	-				1.7	1.8	1.6 ± 0.1	1.5 ± 0.1	1.3 ± 0.1
AF2089		Predicted sugar kinase*	R	-				0.8 ± 0.1	0.8	0.6	0.6	0.6
AF2090-C	AF2090-N	ISA1214-5 transposase	M	+			-1.48	1.8 ± 0.1	1	1.6 ± 0.1	1.4 ± 0.1	1.1
AF2090-N	AF2091	Halomucin*	M	+				2.7	1.9	2.4	2.3	2.2
AF2093		uncharacterized conserved protein*	S	+			**-1.61**	0.4	0.2	0.4	0.3	0.2

AF2094		uncharacterized conserved protein*	S	+				0.2	0.2	0.2	0.1	0.2
AF2095		peptidyl-tRNA hydrolase	J	+				0.4 ± 0.1	0.4	0.4	0.4	0.4
AF2096	radB	DNA repair and recombination protein RadB	L	-				0.3	0.3	0.3	0.2	0.2
AF2097		uncharacterized conserved protein*	S	-				0.5	0.4	0.4	0.4	0.3
AF2098	cdc48-2	cell division protein CDC48	O	+				2.5 ± 0.2	2.6	2.5	2.5	2.4
AF2099	clcB	muconate cycloisomerase II	M	-	1.41			0.1	0.1	0.1	0.2	0.2
AF2100	ilvB-4	acetolactate synthase, large subunit	E	+	**5.72**			0.2	0.2	0.2	1.1 ± 0.2	1.1
AF2101		alcohol dehydrogenase, zinc-dependent	E	+	**4.08**			0.2	0.2	0.2	0.9 ± 0.1	0.9
AF2102		Permease of the major facilitator superfamily*	G	-				0.3	0.2	0.3	0.5 ± 0.1	0.4
AF2103		Permease of the major facilitator superfamily*	G	-				0.1	0.1	0.1	0.1	0.2
AF2104		Radical SAM superfamily enzyme*	R	-	**2.11**			0.3	0.5	0.3	0.7	1
AF2105		Molybdopterin converting factor, small subunit*	H	-	**1.79**			0.2	0.4	0.2	0.4	0.5
AF2106		CTP-dependent Riboflavin kinase*	H	+			-1.37	1.5 ± 0.2	1.1	1.3 ± 0.2	1.6 ± 0.1	1 ± 0.2
AF2107	ribB	3,4-dihydroxy-2-butanone 4-phosphate synthase	H	+				1.1 ± 0.1	1.1	1	1 ± 0.1	0.9
AF2108		uncharacterized conserved protein*	S	+				0.6	0.5	0.7	0.4	0.4
AF2109		signal-transducing histidine kinase	T	-			**2.64**	0.2	0.6 ± 0.1	0.2	0.6 ± 0.3	1 ± 0.1
AF2110		uncharacterized member of the PurR regulon*	R	+				0.8	0.8	0.8	0.7 ± 0.1	0.5
AF2111		Multiple antibiotic transporter*	U	-				0.9 ± 0.1	0.8 ± 0.1	0.9 ± 0.1	1 ± 0.1	1.1 ± 0.1
AF2112	metE	5-methyltetrahydropteroyltriglutamate--homocysteine methyltransferase	E	-				1	0.9	1	1.1 ± 0.1	1.2 ± 0.1
AF2113		hypothetical protein	X	-				1.1	0.9	1.1 ± 0.1	1.2 ± 0.1	1.1
AF2114		hypothetical protein	X	-				0.7 ± 0.1	0.5	0.7 ± 0.1	0.6 ± 0.1	0.5
AF2115		Glycosyltransferase*	M	-				1.8 ± 0.1	1.8 ± 0.1	1.8 ± 0.1	1.6 ± 0.1	1.6 ± 0.1
AF2116	gatB	aspartyl/glutamyl-tRNA amidotransferase subunit B	J	-	-1.26			1.8 ± 0.1	1.9	1.8	1.6	1.3
AF2117	alkA	3-methyladenine DNA glycosylase	L	+				0.3	0.5	0.4	0.3	0.3
AF2118	guaB-2	inosine monophosphate dehydrogenase	K	+			**1.99**	0.2	0.4	0.2	0.3	0.5
AF2119		hypothetical protein	X	-	**5.78**			0.2	0.2	0.2	1 ± 0.1	0.9
AF2120		hypothetical protein	X	-				0.1	0.3	0.1	0.3	0.4
AF2121		hypothetical protein	X	-	**3.06**			0.2	0.7	0.2	1.1 ± 0.1	1.3 ± 0.1
AF2122		hypothetical protein	X	-	**3.44**			0.3	0.6	0.3 ± 0.1	1.5 ± 0.1	1.6
AF2123		hypothetical protein	X	-	**2.98**			0.1	0.3	0.2	0.6 ± 0.2	0.7
AF2124		hypothetical protein	X	-				0.4	0.1	0.6 ± 0.1	0.4 ± 0.1	0.5
AF2125		Predicted permease*	R	-				**3.1**	1.5 ± 0.1	**3.2**	**3.1**	**3.3**
AF2126		3-hydroxy-3-methylglutaryl CoA synthase family enzyme*	R	-				2.6	0.6 ± 0.1	2.9	2.6	2.5
AF2127		LysR family transcriptional regulator	K	-				0.8 ± 0.1	0.2	0.4	0.2	0.2
AF2128	ribH	riboflavin synthase subunit beta	H	+				2.9	2.8	2.7	2.6	2.2
AF2129	aspB-2	aspartate aminotransferase	E	+				2.7	2.9	2.6	2.4	2.3
AF2130		hypothetical protein	X	-				0.2	0.1	0.2	0.1	0.1
AF2131		hypothetical protein	X	-		**-1.54**		0.3	0.3	0.2	0.2	0.2
AF2132		uncharacterized conserved protein*	S	-		-1.30		0.1	0.1	0.1	0.1	0.1
AF2133		hypothetical protein	X	-			-1.19	0	0	0	0	0
AF2134		hypothetical protein	X	+			-1.29	0.1	0	0.1	0	0
AF2135		hypothetical protein	X	+			**-1.61**	0.1	0.1	0.1	0.1	0.1
AF2136		ArsR family transcriptional regulator	K	+				0.1	0.1	0.1	0.1	0.1
AF2137		Predicted Co/Zn/Cd cation transporter*	P	-				0.5 ± 0.1	0.5 ± 0.1	0.5 ± 0.1	0.5 ± 0.1	0.5
AF2138	serB	phosphoserine phosphatase	E	-				1.5 ± 0.1	1.7 ± 0.1	1.5 ± 0.1	1.6 ± 0.2	1.5 ± 0.1
AF2141		hypothetical protein	X	-				0.7 ± 0.1	0.9 ± 0.1	1.1 ± 0.2	0.6 ± 0.1	1 ± 0.2
AF2142	fdx-8	ferredoxin	C	-				1.1 ± 0.1	1.2 ± 0.1	1.5 ± 0.2	1 ± 0.1	1.5 ± 0.2
AF2143		Transcriptional regulator, contains HTH domain*	K	-				0.9 ± 0.1	1.1	1.4 ± 0.1	0.9 ± 0.1	1.2 ± 0.1
AF2144	trx-4	thioredoxin	O	-				1	1	1.5 ± 0.2	1 ± 0.1	1.2 ± 0.2
AF2145	grx-2	glutaredoxin	O	-				0.7 ± 0.1	0.7	1.1 ± 0.1	0.8 ± 0.1	0.7 ± 0.2
AF2146		GTP-binding protein	R	-				0.7	1	0.7 ± 0.1	0.7 ± 0.1	0.7 ± 0.1
AF2147		hypothetical protein	X	-				0.1	0.1	0.1	0.1 ± 0.1	0.2
AF2148		Metal-dependent hydrolase of the beta-lactamase superfamily II*	R	+				0.6	0.6 ± 0.1	0.5	0.4	0.3
AF2149		Phosphate transport regulator (distant homolog of Phou)*	P	-				0.6	1	0.7	0.6	0.8
AF2150	moaC	putative molybdenum cofactor biosynthesis protein C	H	-			1.40	0.8 ± 0.1	1.4 ± 0.1	0.9	0.9 ± 0.1	1.1 ± 0.1
AF2151	entB	isochorismatase	Q	+				0.3	0.6	0.3	0.3 ± 0.1	0.4
AF2152	rps8E	30S ribosomal protein S8e	J	-				2	2.4	1.7 ± 0.1	1.8 ± 0.1	1.9 ± 0.1
AF2153		Zn-dependent hydrolase of the beta-lactamase fold*	R	+	**1.79**			0.4	0.5 ± 0.1	0.5	1.1 ± 0.1	0.7 ± 0.1
AF2154		uncharacterized conserved protein*	S	-				0.7 ± 0.1	0.7	0.6	0.5	0.6
AF2155		Metal-dependent hydrolase of the beta-lactamase superfamily*	R	-				1.4 ± 0.1	1	1.1 ± 0.1	1	0.9
AF2156	cca	tRNA nucleotidyltransferase	J	-				2.2	1.7 ± 0.1	1.9 ± 0.1	1.8 ± 0.1	1.8 ± 0.1
AF2157		2'-5' RNA ligase*	J	-				1.3 ± 0.1	1 ± 0.1	1	0.9	0.9
AF2158		hypothetical protein	X	-		**-2.04**		0.7 ± 0.1	0.5	0.3	0.2	0.3
AF2159		uncharacterized conserved protein*	S	-		**-1.60**		2.4	2.7	1.5 ± 0.1	1.4 ± 0.2	1.7 ± 0.2
AF2160		hypothetical protein	X	-				0	0	0.1	0	0
AF2161		hypothetical protein	X	-				0.1	0.1	0.1	0.1	0.2
AF2162		uncharacterized conserved protein*	S	-				0.2	0.1	0.1	0.2	0.1
AF2163		uncharacterized conserved protein*	S	-				0.1	0	0.1	0.1	0.1
AF2164		hypothetical protein	X	-				0.5 ± 0.1	0.3 ± 0.1	0.3 ± 0.1	0.2	0.3 ± 0.1
AF2165		hypothetical protein	X	-				1.1 ± 0.1	1 ± 0.1	0.7 ± 0.2	0.6 ± 0.1	0.8 ± 0.1
AF2166		hypothetical protein	X	-				1.2 ± 0.2	0.9 ± 0.2	0.7 ± 0.2	0.6 ± 0.1	0.7 ± 0.1
AF2167		hypothetical protein	X	-				1.8 ± 0.2	1.4 ± 0.1	1.2 ± 0.1	1.1 ± 0.1	1.1 ± 0.1
AF2168		Minimal nucleotidyltransferase*	V	-				0.4 ± 0.1	0.4	0.4 ± 0.1	0.3 ± 0.1	0.6 ± 0.1
AF2169		HEPN domain containing protein*	V	-				0.6 ± 0.1	0.7	0.6 ± 0.1	0.5 ± 0.1	0.8
AF2170		uncharacterized conserved protein*	S	-				1.5 ± 0.2	0.7 ± 0.1	0.9 ± 0.2	0.5	0.3
AF2171		hypothetical protein	X	-	**-1.84**	**-1.73**		2.4	1.7 ± 0.1	1.5 ± 0.1	1 ± 0.1	0.9 ± 0.1
AF2172		hypothetical protein	X	-				**3.4**	**3.1**	**3.1**	2.6 ± 0.2	2.2 ± 0.2
AF2173	ncd2	2-nitropropane dioxygenase	R	+	-1.20			0.1	0	0	0	0
AF2175		CRISPR associated protein cas4, RecB family nuclease*	V	-		**-1.98**		0.2	0.3	0.1	0.1	0.1
AF2176	ubiA	4-hydroxybenzoate octaprenyltransferase	H	-	-1.38			1.4 ± 0.1	1.1 ± 0.1	1.3 ± 0.1	1	0.8
AF2177m	lhr-2	ATP-dependent helicase	R	-				1.5	1.4 ± 0.1	1.5 ± 0.1	1.4 ± 0.1	1.1
AF2178		Predicted N6-adenine-specific DNA methylase*	L	+				0.2	0.2	0.2	0.2	0.1
AF2179	moaE	molybdopterin converting factor, subunit 2	H	-			**-1.53**	1.9 ± 0.2	1.1 ± 0.1	1.9 ± 0.2	2	1.3 ± 0.1
AF2180		Phosphoesterase*	R	-				0.6 ± 0.1	0.3	0.6 ± 0.1	0.7 ± 0.1	0.5
AF2181		PD-(D/E)XK superfamily nuclease*	R	+	-1.34	-1.35		0.2	0.1	0.1	0.1	0.1
AF2182		Predicted transcriptional regulator containing an HTH domain fused to a Zn-ribbon*	K	-				0.1	0.1	0.1	0.1	0.1
AF2183		hypothetical protein	X	-	**3.16**			0.2	0.2	0.1	0.6 ± 0.1	0.6
AF2184		Predicted rRNA methylase*	J	-				0.3	0.5	0.3 ± 0.1	0.5 ± 0.1	0.3
AF2185	sucD-2	succinyl-CoA synthetase, alpha subunit	C	-				2 ± 0.1	2.7	2.2	2.9	2.4
AF2186	sucC	succinyl-CoA synthetase, beta subunit	C	-				1.4 ± 0.1	2.5	1.6 ± 0.1	2.2	2.1
AF2187		hypothetical protein	X	-				0	0	0	0	0
AF2188		hypothetical protein	X	+				0	0	0	0	0
AF2189		Transcriptional regulator AbrB*	K	+				0.2	0.3	0.2	0.1	0.2
AF2190		PIN domain containing protein*	V	+				0.1	0.1	0.2	0.1	0.1
AF2191		uncharacterized conserved protein*	S	+				0.3	0.2	0.3	0.2	0.2
AF2192	nrfE	cytochrome C-type biogenesis protein	O	+			1.27	0.5	0.6	0.5	0.4	0.6
AF2193		uncharacterized conserved protein*	S	+				0.8 ± 0.1	1 ± 0.1	0.7 ± 0.1	0.9 ± 0.1	0.8
AF2194		rRNA-methyltransferase, putative	Q	-				0.1	0.2	0.1	0.1	0.1
AF2195	dys1-1	deoxyhypusine synthase	O	-				0.8	1 ± 0.1	0.7	0.7 ± 0.1	0.6 ± 0.1
AF2196		uncharacterized conserved protein*	S	+	**-1.79**	**-1.62**		1.6	1.9 ± 0.1	1.4 ± 0.1	1 ± 0.1	0.8
AF2197		potassium channel, putative	R	-				1.5 ± 0.1	1.7 ± 0.2	1.5 ± 0.2	1.7 ± 0.2	2
AF2198		Predicted metal-dependent protease of the PAD1/JAB1 superfamily*	R	-				1.5 ± 0.2	1.5 ± 0.2	1.5 ± 0.1	1.2 ± 0.2	1.2
AF2199	leuC	3-isopropylmalate dehydratase large subunit	E	-				1.7	1.8 ± 0.1	1.7	1.5 ± 0.1	1.3

AF2200		mutator protein MutT, putative	L	+			1.37	0.4	0.6	0.4	0.4	0.6
AF2201		uncharacterized protein containing a ferredoxin domain*	S	+				0.8	0.5	0.9 ± 0.1	1 ± 0.1	0.8
AF2202		tryptophan-specific permease, putative	S	-				0.5	0.5	0.6	0.5 ± 0.1	0.5
AF2203		Predicted transcriptional regulator, PadR family*	K	-			-1.24	0.3	0.3	0.3	0.3	0.2
AF2204		arylsulfatase regulatory protein, putative	R	+				2.6	2.5	2.8	2.7	2.8
AF2205		hypothetical protein	X	+				0.1	0.1	0.1	0.1	0.1
AF2206		phosphopantetheine adenylyltransferase	R	+				0.6 ± 0.1	1	0.6 ± 0.1	0.8 ± 0.1	0.8 ± 0.1
AF2207	ftr-2	tetrahydromethanopterin formyltransferase	C	+				**3.1**	**3.3**	**3.2**	**3**	**3**
AF2208	thiD	hydroxymethylpyrimidine phosphate kinase	H	+				1.3 ± 0.2	1.6 ± 0.1	1.3 ± 0.1	1 ± 0.1	1 ± 0.1
AF2209		GTPase	R	-				0.3	0.4	0.3	0.4	0.4
AF2210		uncharacterized conserved protein*	S	-	**1.50**			0.3	0.4	0.3	0.6	0.5
AF2211	hit	HIT family protein	F	-			**-1.87**	0.5 ± 0.1	0.3	0.5 ± 0.1	0.6 ± 0.1	0.2
AF2213		PIN domain containing protein*	V	+				0	0	0	0	0.1
AF2214		hypothetical protein	X	+				0	0	0	0	0.1
AF2215	mcmA1	methylmalonyl-CoA mutase, subunit alpha, N-terminus	I	-				2.7	2.4	2.7	2.8	2.8
AF2216	mmdC	methylmalonyl-CoA decarboxylase, biotin carboxyl carrier subunit	I	-			-1.26	2.7	1.9 ± 0.1	2.7	2.6 ± 0.2	2.2
AF2217	mmdA	methylmalonyl-CoA decarboxylase, subunit alpha	I	-				2.6	2.1	2.7	2.6	2.5
AF2218		Lactoylglutathione lyase or related enzyme*	E	-			-1.41	2.4 ± 0.2	1.4 ± 0.1	2.6	2.5	2
AF2219	mcmA2	methylmalonyl-CoA mutase, subunit alpha, C-terminus	I	-				1.4 ± 0.2	0.7 ± 0.1	1.6 ± 0.2	1.4 ± 0.2	1.1 ± 0.1
AF2220		Pyruvate/2-oxoglutarate dehydrogenase complex, dihydrolipoamide dehydrogenase (E3) component or related enzyme*	C	+	**1.54**			0	0	0	0.1	0.1
AF2221		uncharacterized conserved protein*	S	-				0.2	0.2	0.2	0.2	0.3
AF2222		ISA1214-6 transposase	L	-			1.34	0	0	0	0	0.1
AF2223		ISA1214-6 transposase	S	+	-1.24			0	0.1	0.1	0	0
AF2224	valS	valyl-tRNA synthetase	J	-				2	2.3	1.7 ± 0.2	1.7 ± 0.1	1.8 ± 0.1
AF2225	hpcE-2	2-hydroxyhepta-2,4-diene-1,7-dioate isomerase	Q	-	-1.32	-1.29		1.5	1.3	1.3	1 ± 0.1	1 ± 0.1
AF2226		Predicted RNA-binding protein, contains THuMP domain*	R	+		-1.46		0.2	0.3	0.2	0.2	0.1
AF2227		Repressor of nif and glnA expression*	K	-				0.3	0.3	0.3	0.3	0.3
AF2228	dsvC	sulfite reductase, desulfoviridin-type subunit gamma	P	+				1.1 ± 0.1	1.6 ± 0.1	0.8	0.9 ± 0.2	1.1
AF2229	cbiA	cobyrinic acid a,c-diamide synthase	H	-				0.7	0.7	0.6	0.5	0.4
AF2230		uncharacterized conserved protein*	S	+				0.2	0.2	0.3	0.2	0.2
AF2231		uncharacterized conserved membrane protein*	S	-				0.6	0.5	0.5	0.4	0.5
AF2232	fur	ferric uptake regulation protein	P	-				0.3	0.2	0.4	0.3	0.3
AF2233	perA	peroxidase / catalase	P	+				0.5 ± 0.1	0.3	0.5	0.4 ± 0.1	0.5
AF2234		uncharacterized conserved protein*	S	+			-1.46	0.4	0.3	0.4	0.3	0.2
AF2235		uncharacterized conserved protein*	S	+			**-1.89**	0.3	0.1	0.3	0.2	0.1
AF2236		hypothetical protein	X	-				0.2	0.1	0.2	0.2	0.1
AF2237		HAM1 protein	F	+				0.5	0.7 ± 0.1	0.5 ± 0.1	0.4	0.4
AF2238	thsA	thermosome, subunit alpha	O	-				**3.4**	**3.5**	**3.5**	**3.3 ± 0.2**	**3.3**
AF2239		uncharacterized conserved protein*	S	+				0.4	0.3	0.5	0.3	0.2
AF2240		uncharacterized conserved protein*	S	-	**-1.61**			0.7	0.6	0.8	0.5	0.3
AF2241		uncharacterized conserved protein*	S	-	-1.50			1.1	1.3	1.1 ± 0.1	0.8	0.6
AF2242	purB	adenylosuccinate lyase	F	-				1.6 ± 0.2	1.9 ± 0.2	1.6 ± 0.1	1.3 ± 0.1	1 ± 0.1
AF2243	fadA-3	acetyl-CoA acetyltransferase	I	-				1.8 ± 0.1	2.4	1.8 ± 0.1	2.5	2.3
AF2244	acd-11	acyl-CoA dehydrogenase	I	-				1.8 ± 0.2	2.4	1.8 ± 0.1	2.6	2.2
AF2245m		SKI2-family helicase	L	+	-1.42			0.9 ± 0.1	0.9	0.8 ± 0.1	0.6 ± 0.1	0.6
AF2246		Metal-binding trascriptional regulator, contains putative Fe-S cluster and ArsR family DNA binding domain*	K	-				0.1	0.1	0.1	0.1	0.1
AF2247		Predicted permease*	R	+				0.3	0.2	0.3	0.3	0.2
AF2248		Thiol-disulfide isomerase or thioredoxin*	O	+				0.5	0.7	0.6 ± 0.1	0.6 ± 0.1	0.9 ± 0.5
AF2249		response regulator	T	-				0.1	0.1	0.2	0.3 ± 0.1	0.6 ± 0.1
AF2250	pyrC	dihydroorotase	F	-				0.9 ± 0.1	0.7 ± 0.1	1.1 ± 0.1	1 ± 0.1	0.9 ± 0.1
AF2251		competence-damage protein, putative	R	-				1.1 ± 0.1	1 ± 0.1	1.2	1.2 ± 0.1	1.1 ± 0.1
AF2252	argG	argininosuccinate synthase	E	-				2 ± 0.1	2.1	2	1.8 ± 0.1	1.5
AF2253	mobB	molybdopterin-guanine dinucleotide biosynthesis protein B	H	+				0.2	0.1	0.2	0.1	0.1
AF2254	deaD	DEAD-box ATP dependent DNA helicase	L	+				0.3 ± 0.1	0.2	0.3 ± 0.1	0.1	0.1
AF2255	alaS	alanyl-tRNA synthetase	J	+				2.9	**3**	2.9	2.9	**3**
AF2256		F(420)-0:gamma-glutamyl ligase, F420 coenzyme biosynthesis enzyme*	H	+				1.7	1.9	1.7	1.6	1.5
AF2257		Predicted ATP-dependent carboligase related to biotin carboxylase*	R	+				0.2	0.3	0.2	0.1	0.2
AF2258		multidrug resistance protein	G	-		-1.20		1.4	1.3	1.2 ± 0.1	1.1	1.1 ± 0.1
AF2259		tRNA(Ile2) 2-agmatinylcytidine synthetase; containing Zn-ribbon domain and OB-fold domain*	J	-				1.1 ± 0.2	0.8 ± 0.1	1 ± 0.1	0.8 ± 0.2	0.7
AF2260		Predicted RNA-binding protein of the translin family*	J	-				1.8 ± 0.2	1.7	1.8 ± 0.1	1.5 ± 0.2	1.7 ± 0.1
AF2261		tRNA-modifying enzyme	J	-				1.6 ± 0.1	1.7	1.5 ± 0.1	1.3 ± 0.1	1.6
AF2262		uncharacterized conserved protein*	S	+				0.7 ± 0.1	0.7	0.8 ± 0.1	0.8	0.5
AF2263		hypothetical protein	X	+				0.6	0.8	0.7	0.7	0.6
AF2264		HGG motif-containing thioesterase, possibly involved in aromatic compounds catabolism*	Q	+				0	0.1	0	0	0
AF2265	hisH	imidazoleglycerol-phosphate synthase, subunit H	E	-				0.2	0.3	0.2	0.2	0.2
AF2266		Metal-dependent hydrolase of the beta-lactamase superfamily II*	R	+				0.1	0.2	0.1	0.1	0.1
AF2267		NAD(P)H-flavin oxidoreductase	C	-				0.2	0.4	0.2	0.2	0.2
AF2268		Acetoacetate decarboxylase related enzyme*	Q	+				0.1	0.2	0.2	0.2	0.2
AF2269		nucleotide-binding protein	D	-	-1.43			0.3	0.3	0.4	0.2	0.2
AF2270		hypothetical protein	X	-				0	0	0	0	0
AF2271		Predicted transcriptional regulator*	K	+				0.3	0.3	0.3	0.3	0.3
AF2272		NAD(FAD)-dependent dehydrogenase*	R	+				0.3	0.4	0.4	0.4	0.6 ± 0.1
AF2273	hbd-10	3-hydroxyacyl-CoA dehydrogenase	I	+				1.8 ± 0.1	2.3	1.6 ± 0.1	1.8 ± 0.1	1.9 ± 0.1
AF2274		uncharacterized conserved protein*	S	-				0.1	0.1	0.1	0.1	0.1
AF2275	acd-12	acyl-CoA dehydrogenase	I	-				0.3	0.5 ± 0.1	0.2	0.2	0.2
AF2276		uncharacterized conserved protein*	S	-				0.1	0.1	0.1	0.1	0.1
AF2277		DNA polymerase	L	-				0.6 ± 0.1	0.5	0.5	0.5	0.4
AF2278	act-4	pyruvate formate-lyase activating enzyme	O	+			1.15	0.4	0.4	0.3	0.3	0.4
AF2279		Predicted membrane protein*	S	+				0.1	0.1	0.1	0.1	0.1
AF2280		uncharacterized conserved protein*	S	+				0.1	0.1	0.1	0.1	0.1
AF2281	aor-4	aldehyde ferredoxin oxidoreductase	C	+				0.7 ± 0.1	0.9 ± 0.1	0.8	0.8 ± 0.1	0.8
AF2282	rpoD	DNA-directed RNA polymerase subunit D	K	-				2.1 ± 0.2	2.1	1.9	1.8 ± 0.1	1.5 ± 0.1
AF2283	rps11p	30S ribosomal protein S11P	J	-				**3.1**	2.9	**3**	**3**	2.6
AF2284	rps4p	30S ribosomal protein S4	J	-				**3.1**	**3.1**	**3.1**	**3.1**	2.8
AF2285	rps13p	30S ribosomal protein S13P	J	-				2.9	2.9	2.8	2.6	2.5
AF2286	idsA	bifunctional short chain isoprenyl diphosphate synthase	H	-				2.9	2.8	2.8	2.6	2.5
AF2287		isopentenyl pyrophosphate isomerase	H	-				2.6	2.6	2.6	2.5	2.3 ± 0.2
AF2288		acetylglutamate kinase, putative	I	-				1.9 ± 0.3	2.3	2 ± 0.1	2	2.1
AF2289	mvk	mevalonate kinase	I	-				1.8 ± 0.2	2.2	1.9 ± 0.1	1.9 ± 0.1	1.9
AF2290		acetylpolyamine aminohydrolase, putative	R	+				0.8 ± 0.1	0.7	0.9 ± 0.1	0.9 ± 0.1	0.7
AF2291		hypothetical protein	X	+	1.46			0	0	0	0.1	0.1
AF2292		hypothetical protein	X	-				0.8 ± 0.2	0.8 ± 0.1	0.8	0.5 ± 0.1	0.7 ± 0.1
AF2293		hypothetical protein	X	-				0.4	0.5	0.4	0.3	0.4
AF2294		Permease of the major facilitator superfamily*	G	-	**2.49**			0.2	0.2	0.2	0.4	0.6
AF2295		group II decarboxylase	E	-	**2.36**			0.3	0.4	0.3	0.8 ± 0.1	0.9
AF2296	cydA-1	cytochrome oxidase,subunit I	C	-				2.6	2.1	2.4	2.4	2.3
AF2297	cydA-2	cytochrome oxidase, subunit I	C	-				2.9	2.4	2.9	**3 ± 0.2**	**3**
AF2298		Eukaryotic-type DNA primase, catalytic (small) subunit*	L	-				0.2	0.1	0.2	0.3	0.2
AF2299		Phosphatidylglycerophosphate synthase*	I	-				0.5	0.3	0.3	0.4	0.2
AF2300	dys1-2	putative deoxyhypusine synthase	O	-				0.7 ± 0.1	0.6 ± 0.1	0.4 ± 0.1	0.6 ± 0.1	0.4
AF2301		pyruvoyl-dependent arginine decarboxylase	E	-				0.6 ± 0.1	0.4	0.4 ± 0.1	0.5 ± 0.1	0.2
AF2302		uncharacterized alpha+beta fold domain often fused to a RimK-like ATP-grasp enzyme*	S	+				0.4 ± 0.1	0.6 ± 0.1	0.5	0.3 ± 0.1	0.3
AF2303	rimK	ribosomal protein S6 modification protein	H	+				0.5 ± 0.1	0.7 ± 0.1	0.7 ± 0.1	0.3 ± 0.1	0.4

AF2304		Minimal nucleotidyltransferase*	V	+				0.2	0.1	0.2	0.1	0.1
AF2305		hypothetical protein	X	+				0	0	0.1	0	0
AF2306		Predicted Fe-S-cluster oxidoreductase*	R	-				0.1	0.1	0.1	0	0
AF2307		Glutamate-cysteine ligase family 2*	H	-				0.1	0.1	0.1	0.1	0.1
AF2308	arsB	arsenite transport protein	P	-				0.2 ± 0.1	0.1	0.1	0.1	0.1
AF2309		Permease of the major facilitator superfamily*	G	-				0.4 ± 0.2	0.2 ± 0.1	0.3 ± 0.1	0.1 ± 0.1	0.1
AF2310		Predicted dioxygenase*	R	+				0.5 ± 0.1	0.5	0.5 ± 0.1	0.5 ± 0.1	0.5
AF2311		Predicted transcriptional regulator*	K	+			**-1.55**	1.2	0.8 ± 0.1	1.1 ± 0.2	0.9 ± 0.1	0.6
AF2312	rr4	rubrerythrin	C	+	-1.28			1.1	1.1	1.1 ± 0.1	0.8 ± 0.1	0.8
AF2313	maoC	maoC protein	I	+				0.1	0.1	0.2	0.1	0.1
AF2314	ogt	methylated-DNA-protein-cysteine methyltransferase	L	-				0.5 ± 0.1	0.4	0.8 ± 0.1	0.5 ± 0.1	0.4
AF2315		Nicotinamide mononucleotide adenylyltransferase*	H	-				2.1	2.4	2.3	2.3	2.6 ± 0.2
AF2316		Radical SAM superfamily enzyme*	R	-				2.6	2.5	2.7	2.7	2.4
AF2317		RNase P/RNase MRP subunit p30*	J	-				0.7 ± 0.2	0.5 ± 0.1	0.8 ± 0.1	0.4 ± 0.1	0.4
AF2318		Exosome subunit, RNA binding protein with dsRBD fold*	J	-				0.5 ± 0.2	0.4 ± 0.1	0.6 ± 0.1	0.3 ± 0.1	0.3
AF2319	rpl15E	50S ribosomal protein L15e	J	-				3.2	3.2	3.2	3.2	3.1
AF2320	rps3AE	30S ribosomal protein S3Ae	J	-				3.3	3.3	3.3	3.3	3.3
AF2321		GTP:adenosylcobinamide-phosphate guanylyltransferase*	H	+	-1.43			0.2	0.2	0.2	0.2	0.1
AF2322	pcm-2	L-isoaspartyl protein carboxyl methyltransferase	O	+		-1.30		0.4	0.4	0.3	0.3	0.3
AF2323	cobS-2	cobalamin synthase	H	+				0.2	0.2	0.2	0.2	0.2
AF2324		fmu and fmv protein	J	-				0.9 ± 0.1	0.8 ± 0.1	0.9 ± 0.1	0.9 ± 0.1	0.8
AF2325		uncharacterized conserved protein*	S	-				2.1 ± 0.2	2.3	2.3	2.2	2.3
AF2326		translation initiation factor IF-2 subunit beta	J	-				1.2	1.3 ± 0.1	1.1 ± 0.1	1.1 ± 0.1	1.2 ± 0.1
AF2327	aroE	shikimate 5-dehydrogenase	E	+	-1.43			0.5	0.6	0.6 ± 0.1	0.4	0.3
AF2328	gatC	aspartyl/glutamyl-tRNA amidotransferase subunit C	J	+				0.4 ± 0.1	0.5	0.4	0.3	0.3
AF2329	gatA-2	Glu-tRNA amidotransferase, subunit A	J	+				1.1 ± 0.1	1.5	1.1	1 ± 0.1	1
AF2330		Restriction endonuclease family enzyme*	L	-				0.3	0.2	0.3	0.2	0.2
AF2331		uncharacterized conserved protein*	S	-	**-1.52**			0.3	0.2	0.3	0.2	0.2
AF2332		flavodoxin, putative	X	+				0.1	0.1	0.1	0.1	0.1
AF2333		Predicted aconitase*	R	-				0.9	0.9 ± 0.1	0.7 ± 0.1	0.7 ± 0.1	0.6
AF2334	speE	spermidine synthase	E	-	-1.23	-1.25		0.9	0.9	0.8 ± 0.1	0.7	0.7
AF2335		Amidase related to nicotinamidase*	Q	+				0.1	0.2	0.1	0.1	0.2
AF2336	est-3	carboxylesterase	R	-	**1.91**			0.1	0.1	0.1	0.2	0.2
AF2337		hypothetical protein	X	-				0.2	0.1	0.1	0.1	0.1
AF2338		Predicted ATPase (AAA+ superfamily)*	R	+				0.2	0.1	0.2	0.1	0.1
AF2339		hypothetical protein	X	+				0.3	0.3	0.4	0.3	0.4
AF2340		Predicted ATPase (AAA+ superfamily)*	R	+				0.2	0.2	0.3	0.2	0.3
AF2341		hypothetical protein	X	+				0.1	0.1	0.1	0.1	0.1
AF2342		Cytotoxic translational repressor of toxin-antitoxin stability system*	V	-				0.2	0.2	0.2	0.2	0.2
AF2343		hypothetical protein	X	-				0.2 ± 0.1	0.4	0.3	0.2	0.3 ± 0.1
AF2344		uncharacterized conserved protein*	S	+	1.33			0.1	0.1	0.1	0.1	0.1
AF2345m		DNA methylase containing a Zn-ribbon module	L	+				0.1	0.1	0.1	0.1	0.1
AF2348		hypothetical protein	X	+				0	0	0	0	0
AF2350		ATP-dependent RNA helicase HepA, putative	K	+				0.1	0	0.1	0.1	0.1
AF2351		uncharacterized conserved protein*	S	+				0.1	0.1	0.1	0.1	0.1
AF2352		NADH dehydrogenase, subunit 1, putative	X	+				0	0	0	0	0
AF2353		hypothetical protein	X	+				0.1	0.2	0.1	0.1	0.1
AF2354		hypothetical protein	X	+				0	0	0	0	0
AF2355		hypothetical protein	X	+				0	0	0	0	0
AF2356		Predicted RNA-binding protein containing PuA-domain*	R	+			**1.63**	0.1	0.1	0.1	0.1	0.2
AF2357		Transglutaminase-like enzyme, putative cysteine protease*	E	+				0.1	0.1	0	0.1	0.1
AF2358		Phage integrase family protein*	L	-				0	0	0	0	0
AF2359		Transcriptional regulator AbrB*	K	+				0.1	0.2	0.1	0.1	0.1
AF2360		PIN domain containing protein*	V	+				0.2	0.2	0.1	0.1	0.1
AF2361		mRNA 3'-end processing factor, putative	J	-				1.5 ± 0.1	1.7 ± 0.1	1.2	1.1 ± 0.1	1.1 ± 0.1
AF2362		uncharacterized conserved protein*	S	+				0.8 ± 0.1	0.9	0.8	0.7	0.6
AF2363		Hydrogenase maturation factor*	O	-				0.8 ± 0.1	1.3	0.8	0.7	0.9
AF2364		ABC transporter, ATP-binding protein	O	+				1.8 ± 0.1	1.9 ± 0.1	2 ± 0.2	1.7 ± 0.1	1.5 ± 0.1
AF2365		Cysteine desulfurase activator SufB*	O	+				1.7 ± 0.1	1.7 ± 0.1	1.8 ± 0.1	1.7 ± 0.1	1.3 ± 0.1
AF2366	aspB-1	aspartate aminotransferase	E	+				1.3 ± 0.1	1.3	1.1 ± 0.1	1.1 ± 0.1	0.7
AF2367		Acetyltransferase (GNAT) family*	R	+	-1.48			0.6	0.7	0.6	0.5	0.4
AF2368	fadD-9	long-chain-fatty-acid--CoA ligase	I	-	1.43			0.8 ± 0.1	0.9 ± 0.1	0.6 ± 0.1	1.3 ± 0.1	1.1 ± 0.1
AF2369		TPR repeats containing protein*	R	-				1.7 ± 0.1	1.6	1.4 ± 0.1	1.4 ± 0.1	1.3
AF2370		uncharacterized Zn-finger containing protein*	R	-				0.2	0.3	0.2	0.2	0.2
AF2370a		uncharacterized conserved protein*	S	+				0.1	0.2	0.1	0.1	0.1
AF2371		Dolichol kinase*	I	+				0.2	0.1	0.1	0.1	0.1
AF2372	suhB	extragenic suppressor	G	+	-1.25			0.9	0.9	0.8	0.6	0.7
AF2373		NAD kinase*	H	+				0.5	0.5	0.5	0.3	0.4
AF2374		Periplasmic component of the Tol biopolymer transport system*	U	+				0.1	0.1	0.1	0.1	0.1
AF2375		uncharacterized conserved protein*	S	+				0.5	0.6	0.5 ± 0.1	0.5 ± 0.1	0.7 ± 0.1
AF2376		uncharacterized conserved protein*	S	+			**1.84**	0.1	0.2	0.1	0.1	0.2
AF2377		NifX family protein*	S	+	-1.27			2.1	2	2.1	1.8 ± 0.1	1.4
AF2378		NifX family protein*	S	+				3.2	3	3.2	3.1	3
AF2379		hypothetical protein	X	+				3.5	3.4	3.5	3.5	3.5
AF2380		iron-sulfur cluster binding protein	C	+				3.2	3.1	3.2	3.2	3.3
AF2381		iron-sulfur cluster binding protein	C	+				2.7	2.6	2.8	2.7	3
AF2382		nucleotide-binding protein	D	+				1.7 ± 0.2	1.5 ± 0.2	1.7 ± 0.1	1.5 ± 0.1	1.5
AF2383		Metal-dependent hydrolase of the beta-lactamase superfamily II*	R	+				0.3 ± 0.1	0.3 ± 0.1	0.3 ± 0.1	0.2	0.3
AF2384		molybdopterin oxidoreductase, molybdopterin binding subunit	C	+		1.28		2.8	2.4	**3.5**	**3.3**	**3.4**
AF2385		molybdopterin oxidoreductase, iron-sulfur binding subunit	C	+		**2.08**		1.4 ± 0.1	1	**3**	2.3	2.6
AF2386		molybdopterin oxidoreductase, membrane subunit	P	+		**1.95**		1.2 ± 0.1	1.1 ± 0.1	2.6	1.9 ± 0.1	2.5
AF2387		Putative protein-S-isoprenylcysteine methyltransferase*	O	+				0.1	0.1	0.3	0.1	0.2
AF2388		Predicted Co/Zn/Cd cation transporter*	P	+				0.3	0.2	0.3	0.2	0.2
AF2389-C	AF2389-N	acetyl-CoA synthetase, putative	I	+				0	0	0	0	0
AF2389-N	AF2389-C	Acyl-coenzyme A synthetase/AMP-(fatty) acid ligase*	I	+				0.3	0.2	0.5	0.3	0.3
AF2391		uncharacterized conserved protein*	S	+				0.5	0.8 ± 0.1	0.4	0.4	0.6 ± 0.1
AF2392		uncharacterized conserved protein*	S	+		1.34		1.4 ± 0.1	2.1 ± 0.2	1.5 ± 0.1	1.3 ± 0.1	1.8 ± 0.2
AF2393		hypothetical protein	X	-				1 ± 0.1	0.7 ± 0.1	1.2 ± 0.1	0.9 ± 0.1	1 ± 0.1
AF2394	feoB-2	iron (II) transporter	P	-				2.9 ± 0.4	1.2 ± 0.5	3.1	2.9 ± 0.2	3.1 ± 0.2
AF2395		iron-dependent repressor	K	-				3.2 ± 0.2	2.3 ± 0.5	3.4	3.3	3.3
AF2396		Fe^{2+} transport system protein A*	P	-				2.4 ± 0.4	1.1 ± 0.4	2.9	2.5 ± 0.2	2.1 ± 0.2
AF2397	cdhA-2	acetyl-CoA decarbonylase/synthase complex subunit alpha	C	+				3.6	3.5	3.5	3.6	3.6
AF2398	cdhB-2	acetyl-CoA decarbonylase/synthase complex subunit epsilon	C	+				3.4	3.4	3.3	3.3	3.3
AF2399		rRNA methylase, putative	J	+	-1.31			0.1	0.1	0.1	0.1	0.1
AF2400		Transglutaminase-like enzyme, putative cysteine protease*	E	+		-1.31		0.7 ± 0.1	0.6	0.4	0.4	0.5
AF2401		uncharacterized conserved membrane protein*	S	+		-1.42		0.6 ± 0.1	0.5	0.4	0.3	0.4
AF2402		Histone acetyltransferase*	K	+				1.4 ± 0.1	1.3	1.1	1 ± 0.1	0.8 ± 0.1
AF2403		uncharacterized conserved protein*	S	-				0.1	0.1	0.1	0.1	0.1
AF2404		Predicted transcriptional regulator*	K	+				0.2	0.3 ± 0.1	0.2 ± 0.1	0.6 ± 0.4	0.6 ± 0.1
AF2405		Predicted metal-binding protein*	R	+				0.8 ± 0.1	0.5	0.6 ± 0.1	0.4 ± 0.1	0.3
AF2406		ATP-dependent RNA helicase, putative	R	-				1.6 ± 0.1	1.4 ± 0.1	1.4 ± 0.1	1.3	0.9
AF2407		Metal-dependent hydrolase of the beta-lactamase superfamily II*	R	-				0.6	0.4	0.6	0.7 ± 0.1	0.6
AF2408		Alkylhydroperoxidase family enzyme*	R	-			**-1.97**	0.6 ± 0.1	0.3	0.5	0.4 ± 0.1	0.2

AF2409		iron-sulfur cluster binding protein	C	+	**2.48**				0.2	0.1	0.2	0.5 ± 0.1	0.5
AF2410		uncharacterized conserved protein*	S	+					0.5	0.5	0.4	0.5	0.5
AF2411		Coenzyme F420-dependent N5,N10-methylene tetrahydromethanopterin reductase or related flavin-dependent oxidoreductase*	C	+					1.4 ± 0.1	1.4	1.3 ± 0.1	1.2 ± 0.1	1.1 ± 0.1
AF2412	thiC	thiamine biosynthesis protein ThiC	H	+					1.4 ± 0.1	1.4	1.4	1.3 ± 0.1	1.1
AF2413	pqqE	coenzyme PQQ synthesis protein	R	+					0.8	0.8	0.8	0.8 ± 0.1	0.7
AF2414		Predicted transcriptional regulator*	K	+	1.39				1.8 ± 0.2	1.7 ± 0.1	1.5 ± 0.1	2.4 ± 0.2	2.6
AF2415	acaA-2	3-hydroxy-3-methylglutaryl CoA synthase*	I	+	1.31				2 ± 0.2	1.8	1.8 ± 0.1	2.6 ± 0.2	2.7
AF2416	acaB-12	acetyl-CoA acetyltransferase	I	+	**1.58**				1.7 ± 0.1	1.5	1.4 ± 0.1	2.3 ± 0.2	2.8
AF2417		Predicted nucleic-acid-binding protein containing a Zn-ribbon*	R	+					0.2	0.2	0.2	0.3 ± 0.1	0.4
AF2418		DNA repair protein, putative	L	-					0.3	0.4	0.3	0.2	0.3
AF2419		response regulator	T	-					0.4	0.5	0.5	0.5	0.7
AF2420		signal-transducing histidine kinase, putative	T	-	**3.46**				0.3	0.2	0.2	0.7 ± 0.1	1
AF2421	leuS	leucyl-tRNA synthetase	J	-					2.3	2.2	2.2	2.1	1.6
AF2422		Predicted adenosine-specific kinase*	L	-					1 ± 0.1	1.3	1.1	0.9 ± 0.1	1.1 ± 0.1
AF2423		uncharacterized conserved protein*	S	-					1.5 ± 0.1	1.2 ± 0.1	1.3 ± 0.1	1.1	1.1 ± 0.1
AF2424		uncharacterized conserved protein, DuF58 family, contains vWF domain*	R	-		-1.15			1.9 ± 0.1	1.8 ± 0.1	1.7 ± 0.1	1.5 ± 0.1	1.6 ± 0.1
AF2425	moxR	methanol dehydrogenase regulatory protein	R	-					1.1 ± 0.1	1	0.9	0.8 ± 0.1	0.7
AF2426		RIO-like serine/threonine protein kinase fused to N-terminal HTH domain*	T	+					0.3	0.3	0.3	0.3	0.2
AF2427		Possible nuclease of RNase H fold, RuvC/YqgF family*	R	+					1.3 ± 0.2	1.4 ± 0.1	1.4 ± 0.1	1.4 ± 0.2	1.7 ± 0.1
AF2428		Sugar phosphate isomerase/epimerase*	G	+					0.5 ± 0.1	0.5 ± 0.1	0.5 ± 0.1	0.4 ± 0.1	0.7 ± 0.1
AF2429	fad-5	enoyl-CoA hydratase	I	+	-1.45				1.4 ± 0.1	1.6 ± 0.1	1.4 ± 0.1	1.1 ± 0.1	0.9
AF2430	icc	lacZ expression regulatory protein	R	+	-1.35				1.4 ± 0.1	1.5	1.6 ± 0.1	1.2 ± 0.1	0.9
AF2431		pyruvoyl-dependent arginine decarboxylase	E	-					0.7	0.6	0.9	0.5	0.4
AF2432		Minimal nucleotidyltransferase*	V	-			-1.15		0.3	0.2	0.3	0.3	0.2
AF2433		HEPN domain containing protein*	V	-					0.2	0.2	0.2	0.3	0.3
AF2434		CRISPR-associated protein Cas2*	V	-					0.1	0.1	0.1	0.1	0.1
AF2435		CRISPR-associated protein Cas1*	V	-					0.3	0.2	0.2	0.3	0.4 ± 0.1
AF2436		CRISPR-associated protein Cas4, RecB family exonuclease*	V	-					0.3	0.2	0.3	0.5	0.5 ± 0.1
