# Supplementary figures and images for: Identification of key components in the energy metabolism of the hyperthermophilic sulfate-reducing archaeon Archaeoglobus fulgidus by transcriptome analyses
Source: Front Microbiol. 2014 Mar 11;5:95. doi: 10.3389/fmicb.2014.00095 (PMC3949148; doi:10.3389/fmicb.2014.00095)

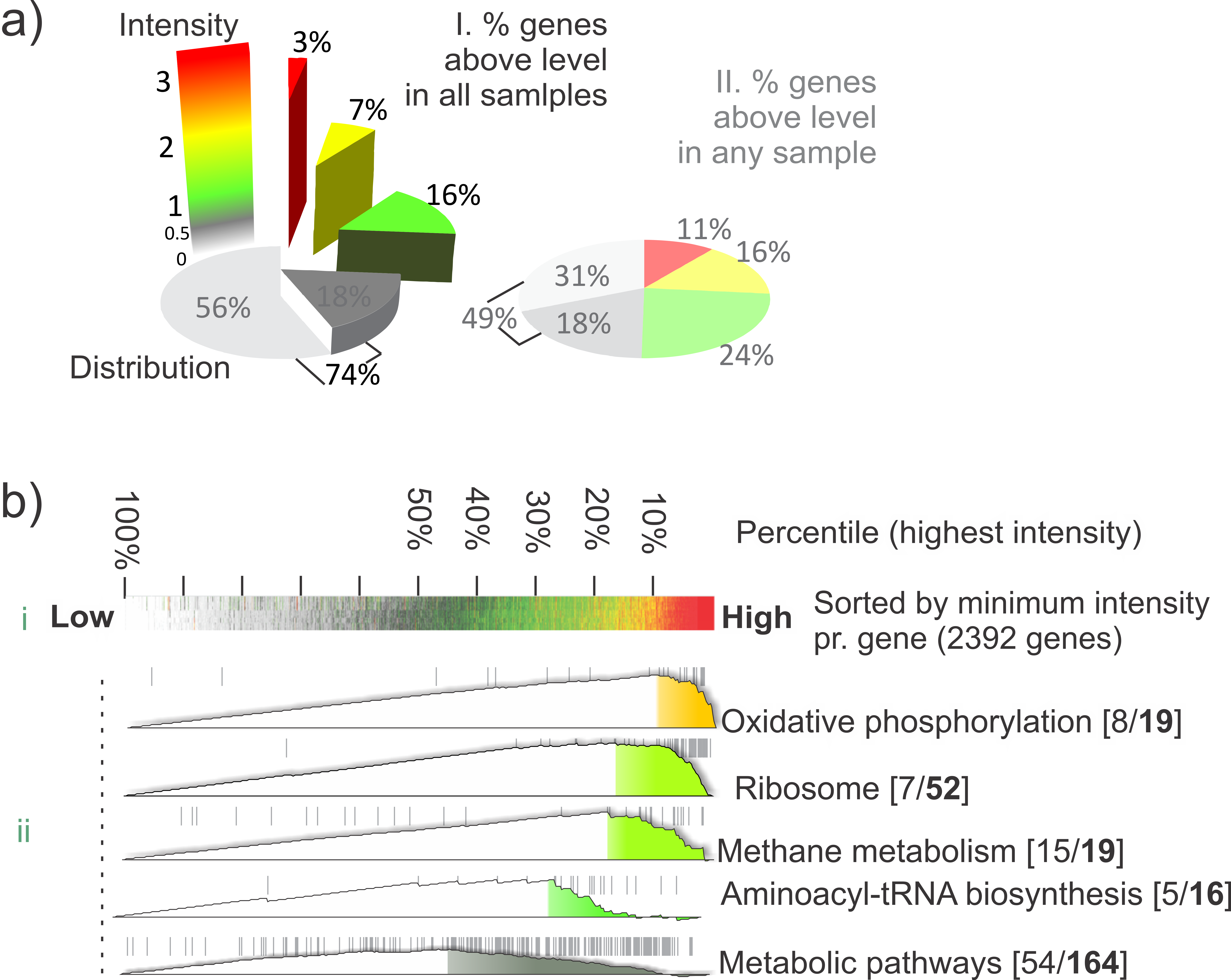

Supplement: Figure S1 — Distribution of array abundance and correlation with and central metabolic processes. (A) Distribution of intensity of quantile-normalized data; (A-I) distribution of values corresponding to minimum expression per gene and (A-II) maximum expression per gene. (B) Gene set enrichment analysis (GSEA) (Subramanian et al., 2005) on the distribution of selected genes on a list of genes sorted by minimum intensity values (i, corresponding to pie chart A-I), i.e., deviation from a random distribution, is displayed for identified peptides and genes corresponding to KEGG pathways. The maximum enrichment score (ES) corresponds to the largest deviance from random distribution, the region above the maximum enrichment score corresponds to the leading edge (LE), this region is colored corresponding to the intensity at point of ES. The numbers denote—[values below LE/above LE]. [file Presentation1.ZIP › 58387_Steen_Suppl_Figure_1.TIF]
